# How Fe(II)/2-Oxoglutarate Oxygenase Chooses Chlorination over Hydroxylation: Electric Field-Driven Ligand Exchange Governs C–Cl Formation

**DOI:** 10.1021/jacs.5c22369

**Published:** 2026-05-23

**Authors:** Simahudeen Bathir Jaber Sathik Rifayee, Midhun George Thomas, Anandhu Krishnan, Kritika Gupta, Carter Davis, Tatyana Karabencheva-Christova, Christo Z. Christov

**Affiliations:** † Department of Chemistry, 3968 Michigan Technological University, Houghton, Michigan 49931, United States; ‡ Department of Chemical Engineering, 3968 Michigan Technological University, Houghton, Michigan 49931, United States

## Abstract

Non-heme Fe­(II)/2-oxoglutarate (2OG)-dependent halogenases catalyze highly selective C–H halogenation. BesD is a non-heme Fe­(II)/2OG halogenase that performs regio- and stereoselective chlorination of the l-lysine (l-Lys) substrate. Understanding the mechanism by which halogenation is favored over canonical hydroxylation is essential for guiding enzyme engineering efforts aimed at converting hydroxylases into halogenases. Here, we combine molecular dynamics (MD) and hybrid quantum mechanics/molecular mechanics (QM/MM) calculations to elucidate the origin of chlorination selectivity in BesD and variants derived from a homologous hydroxylase. Our results indicate that, although the initial inline Cl–Fe­(III)–OH intermediate is inherently predisposed toward hydroxylation, it undergoes a two-step isomerization in which the −Cl and −OH ligands exchange coordination positions. This rearrangement positions the chloride ligand *trans* to H207, where the protein’s intrinsic electric field (IEF) enhances Fe–Cl bond polarization and promotes C–Cl bond formation, ultimately enabling the selective chlorination of l-Lys. Comparative analysis of the homologous hydroxylase and two halogenation-competent variants (Hydrox-3R and Chimera14) reveals halogenase-specific correlated motions between substituted second coordination sphere, long-range residues with the active-site, particularly the coordinated succinate, and the substrate. Notably, these collective motions mirror those observed in the native halogenase BesD. Furthermore, the Hydrox-3R and Chimera14 variants employ a two-step isomerization strategy analogous to that of BesD, enabling efficient chlorination through IEF alignment along the Fe–Cl bond. These results highlight the critical roles of collective correlated motions, the second coordination sphere, and long-range interactions, as well as enzyme-generated electric fields, in determining halogenation selectivity in non-heme Fe­(II)/2OG-dependent oxygenases. Ultimately, halogenation is achieved not due to a new catalytic mechanism, but rather due to subtle electronic, geometric, and dynamic perturbations of a hydroxylase scaffold. These insights provide a mechanistic framework for engineering hydroxylases into halogenases with enhanced activity.

## Graphical abstract

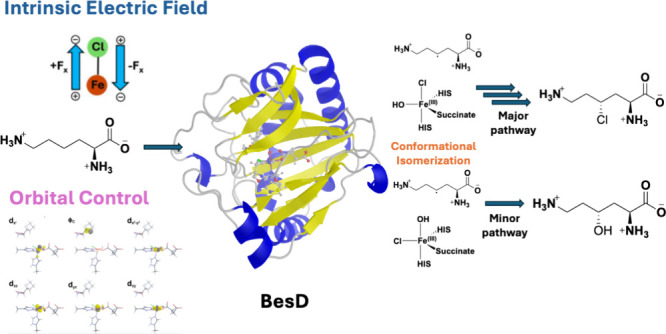

## 1 Introduction

Halogenation-the introduction of halogen atoms into organic molecules is a key chemical transformation that can profoundly influence molecular reactivity, polarity, and biological activity.^1,2^ In nature, halogenated natural products constitute a diverse and important class of bioactive compounds with broad applications in medicine and agriculture, including antibiotic, antifungal, and anticancer activities.^3^ These properties make halogenation an essential reaction both in natural biosynthetic pathways and in synthetic chemistry.^4–7^ Within biosynthetic pathways, enzymes play a critical role in catalyzing halogenation reactions. Among them, non-heme Fe­(II)/2-oxoglutarate (2OG)-dependent halogenases are particularly notable for their ability to carry out site-, regio- and stereoselective halogenation under mild physiological conditions.^7–13^

Enzymatic C–H activation has been an active area of research because of its high selectivity and specificity under physiological conditions, in contrast to the often harsh reaction conditions required by synthetic methods.^14–16^ Non-heme Fe­(II)/2OG-dependent enzymes constitute a large superfamily that catalyzes diverse oxidative transformations initiated by C–H activation, including hydroxylation, desaturation, ring formation, epimerization, and halogenation.^7,17–21^ The active site of these enzymes typically features a facial triad motif (His-X-His, where X = Asp/Glu or a halide) that coordinates the Fe­(II) center. In the Fe­(II)-bound resting state, the metal is further coordinated by three water molecules, completing an octahedral geometry.^10,17^ The catalytic cycle begins with 2OG binding to the Fe­(II) center in a bidentate fashion, displacing two coordinated water molecules. Subsequent substrate binding displaces the third water molecule, generating a catalytically competent enzyme–substrate complex with one vacant coordination site available for dioxygen binding. Two possible 2OG binding configurations have been proposed: one in which the C1 carboxylate is positioned *trans* to higher histidine (H207 in BesD), forming an offline 2OG configuration, and another in which the C1 carboxylate is *trans* to lower histidine (H140 in BesD, Figure 1). Dioxygen binds to the Fe­(II) center to form either an offline or an inline Fe­(III)-superoxo complex. This complex triggers the oxidative decarboxylation of 2OG, yielding succinate and CO_2_ and generating the corresponding offline or inline Fe­(IV)=O (ferryl) intermediate (Figure S1). The ferryl species abstracts a hydrogen from the substrate, forming a substrate radical and an Fe­(III)–OH intermediate. The subsequent radical rebound step determines the nature of the final product (Figure S1). In most non-heme Fe­(II)/2OG oxygenases, the hydroxyl group rebounds to the substrate radical, producing hydroxylated product. In halogenases, however, the metal coordination environment differs: one of the negatively charged residues (Asp/Glu) is replaced by glycine/alanine, creating an open coordination site for halide binding. As a result, at the Fe­(III)–OH intermediate state, competition arises between the −OH and halide ligands, and the outcome of this competition dictates whether halogenation or hydroxylation occurs (Figure S1).^8,9,22–28^ The protein environment plays a critical role in orienting both the halide and the substrate radical to favor halogen rebound over hydroxyl rebound, thereby enabling selective halogenation (Figure 1).^23,27,29^

**Figure 1:**
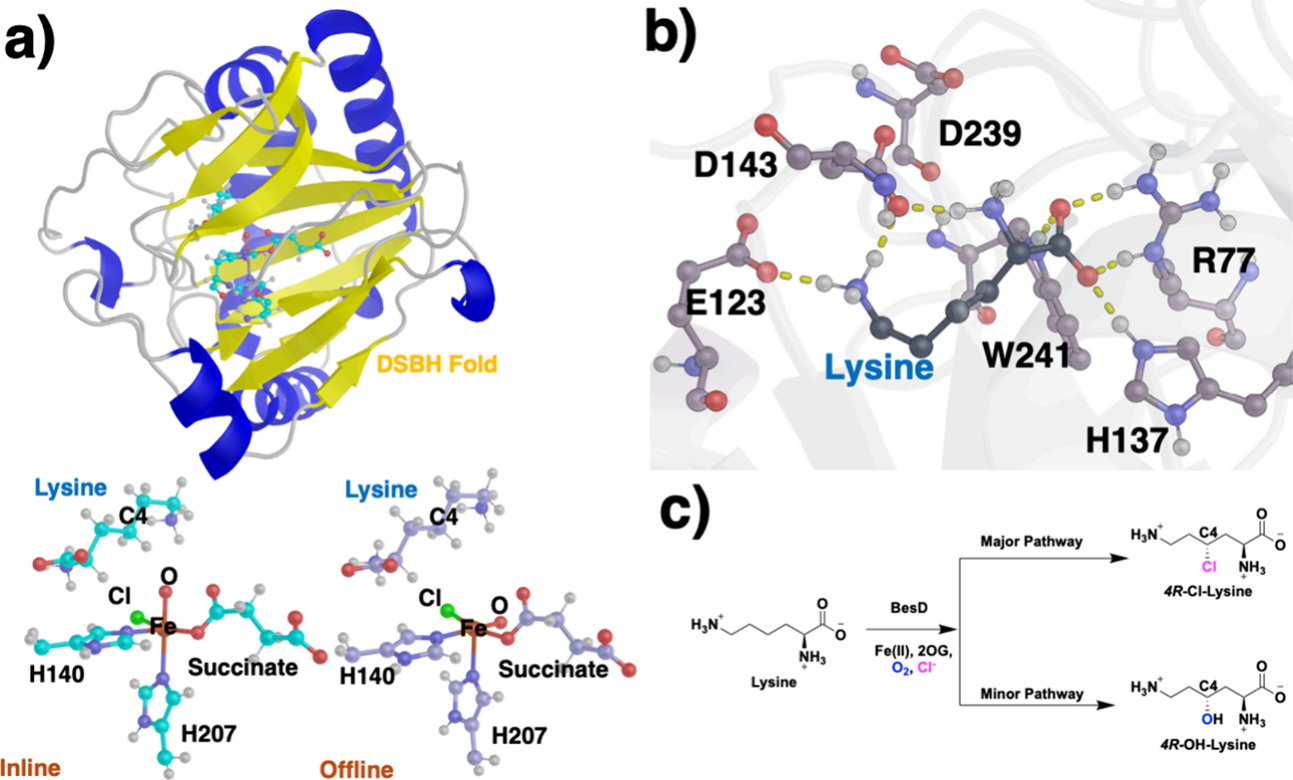
(a) Overall fold of BesD (PDB ID: 6NIE). (top) Yellow-colored regions represent the β-sheets of the DSBH fold, and blue-colored regions represent the α-helices. (bottom) 3D representation of the computationally modeled BesD active site, depicting Fe­(IV) bonded with a facial triad, oxygen, succinate, and chloride ligand in inline orientation and offline orientation. (b) Substrate binding interactions surrounding the Lys substrate. (c) Reactions catalyzed by the BesD enzyme.

SyrB2 from *Pseudomonas syringae* was the first identified non-heme Fe­(II)/2OG-dependent halogenase catalyzing the regioselective chlorination of threonine.^28,30,31^ However, efficient halogenation requires threonine to be tethered to the carrier protein SyrB1. Since this discovery, several non-heme Fe­(II)/2OG-dependent halogenases that act on free substrates have been identified, including WelO5,^25,26^ HalA,^32^ HalB,^33^ AmbO5,^34^ DAH,^35^ AdeV,^8^ and BesD.^9,36^ BesD was a radical halogenase obtained from *Streptomyces cattleya* involved in the biosynthetic pathway of a terminal alkyne-containing amino acid.^9,37^ It catalyzes the regio- and stereoselective chlorination of free l-lysine at the C4 position to form (*4R)*-chloro-l-Lysine. BesD is particularly intriguing because it shares very low sequence similarity with other halogenases-only (7%) with SyrB2 and (11%) with WelO5- yet displays relatively high sequence similarity (≤46%) to a putative hydroxylase homologue. Structurally, BesD adopts the double-stranded β-helix (DSBH) topology characteristic of the Fe­(II)/2OG enzyme family (Figure 1).^9,29^ Its active site contains the conserved facial triad that coordinates Fe­(II), which, in turn, binds 2OG and a chloride ligand (Figure 1). Motivated by its high sequence similarity to the hydroxylase, an experimental study characterized a hydroxylase homologue of BesD^29^ and demonstrated that it could be converted into a halogenase through targeted modifications (mutations) in the first and second coordination spheres (SCSs).^29^ The key alteration involved replacing the carboxylate ligand (Glu/Asp) with a noncoordinating residue (Gly), thereby freeing a coordination site for halide binding. However, this single substitution was insufficient to achieve efficient halogenation. To enhance halogenase activity, two variants were engineered. The first involved substitution in the facial triad along with two additional mutations (Hydrox D142G, N149I, V223N (Hydrox-3R)). The second replaced 13 residues with amino acids conserved among halogenases (Chimera14). These variants achieved chlorination selectivities of 84% (Hydrox-3R) and 90% (Chimera14), although both exhibited reduced substrate turnover relative to hydroxylase.^29^ Thus, elucidating the catalytic mechanism of these variants offers a powerful comparative framework for understanding how specific structural features of individual and collective changes, and more subtle contributions such as protein dynamics, substrate positioning, and electric field effects, drive the transformation of a hydroxylase into a halogenase.

Computational studies have been widely used in synergy with experimental efforts, providing novel insights into the effects of protein environment, including the SCS and long-range (LR) interactions, correlated motions, and the electric field, on the catalytic mechanism of non-heme Fe­(II)/2OG enzymes.^22,31,38–47^ In particular, computational investigations of halogenases,^48,49^ including SyrB2,^27,31,38^ WelO5,^26^ and BesD,^22,50,51^ have proposed different mechanistic strategies underlying their chlorination selectivity. For example, two interconverting ferryl species were identified experimentally in SyrB2,^52^ and computational studies identified only one of them as responsible for effective chlorination of threonine substrate.^31^ The same study, further reported that isomerization of the Cl–Fe­(III)–OH complex proceeds with high energy barriers.^31^ Experimental kinetics, Mössbauer spectroscopy, and product analysis have revealed that AdeV forms two ferryl intermediates sequentially, but only the early species enables C–H activation and subsequent C–Cl formation. Furthermore, molecular dynamics (MD)-based Quantum Mechanics/Molecular Mechanics (QM/MM) calculations identified the offline ferryl configuration as the active intermediate that undergoes σ-channel hydrogen atom transfer (HAT) and favors -Cl over −OH rebound, whereas the latter, inline ferryl state is unreactive.^53^ A QM/MM study on WelO5 showed that the equatorial Fe­(IV)=O (offline) configuration is responsible for HAT and chlorination preference, as the SCS residue S189 forms a hydrogen bond with the −OH group formed after HAT, thereby preventing rebound hydroxylation.^26^ A recent study on the HalA enzyme demonstrated isomerizations of the Cl–Fe­(IV)=O intermediate, involving a positional switch between the chloride and oxo ligand using experimental techniques. The same work also proposed, based on DFT calculations, that the isomerization of the Cl–Fe­(III)–OH complex, entailing a switch between the −Cl and −OH ligand positions, may facilitate selective halogenation.^32^ A QM/MM study on BesD also proposed a conformational isomerization of the Cl–Fe­(III)–OH complex as a strategy for achieving chlorination, involving a flip of the −OH group from an inline to an offline orientation.^51^ However, that study did not explore in detail the conformational dynamics of the BesD enzyme, particularly with respect to effects of SCS and LR interactions, correlated motions, and electric field.^51,54–56^ BesD has also been shown to bind fluoride, activate O_2_, form an F–Fe­(IV)=O intermediate, and perform H-abstraction from l-Lys, but it yields exclusively hydroxylated product, establishing that the failure of fluorination arises only at the final radical-coupling step. Spectroscopic data indicate that F^–^ remains coordinated throughout all catalytic states, while QM/MM calculations reveal that F• rebound has a substantially higher barrier than HO• rebound-even after a two-step isomerization in which the −OH and −F ligands exchange positions.^57^ Notably, an analogous isomerization pathway has not been assessed for chlorination in BesD. In the present study, we employed MD-based QM/MM calculations to examine the structural and mechanistic basis of chlorination preference in BesD, Chimera14, and Hydrox-3R, with particular focus on correlated motions, SCS and LR interactions, and electric fields. Additionally, we investigated the conformational dynamics and catalytic mechanism of the Hydrox enzyme to enable a rigorous comparison with the halogenase variants obtained from it, as well as with BesD. We propose an alternative strategy for the chlorination preference in BesD and in the halogenase variants obtained from Hydrox, supported by quantitative analysis of energetics and electric field. The insights obtained from this study may guide future enzyme redesign efforts aimed at converting a hydroxylase into a halogenase with enhanced efficiency.

## 2 Methods

### 2.1 System Preparation

Initially, we obtained the crystal structures of wild-type BesD (PDB ID: 6NIE) and lysine hydroxylase (PDB ID: 7JSD).^9,29,58^ The BesD crystal structure has the 2OG in the offline orientation with the C1 carboxylate *trans* to H207. For modeling the Fe­(III)-superoxo complex of the BesD, the dioxygen (O_2_) was added to the Fe *trans* to H140, along with bidentate 2OG, a chloride ion (Cl-), and two histidines (H140, H207). The initial structure of the inline ferryl intermediate for the BesD was prepared by modifying the active site to obtain Fe coordinated to oxo ligand (Fe­(IV)=O) (*trans* to H207 for inline configuration), monodentate succinate, a chloride ion, H140, and H207. Hydrogens were added to the protein based on the p*K*_a_ values obtained from the PROPKA program.^59^ The parameters for the lysine substrate, 2OG, and succinate were obtained using the generalized Amber force field (GAFF)^60^ implemented in the antechamber suite in AmberTools20.^61,62^ The metal center parameters, including bond, angle, and torsion force constants at Fe­(III)-superoxo and Fe­(IV)=O states, were obtained at the high-spin quintet state using the Metal Center Parameter Builder (MCPB.py v3.0) of AmberTools20.^63^ Protein residues were modeled using the Amber ff14SB force field.^64^ To neutralize the system, Na+ counterions were added using the Leap module in AmberTools20.^62^ Following neutralization, the system is solvated using TIP3P^65^ water molecules with the upper limit of 10 Å from the farthest point on the protein surface. All these procedures are followed for the Hydrox enzyme as well, with the active site modeled with Fe coordinated to oxo, monodentate succinate, H140, D142, and H207.

### 2.2 MD Simulations

The modeled systems were minimized in two steps- first, the solvent molecules were minimized with a 500 kcal/mol restraint on the solute to remove bad contacts, followed by complete minimization of the system. Both minimizations were performed for 10,000 steps each (5000 steepest descent and 5000 conjugate gradient). Following minimizations, the systems were subjected to heating from 0 to 300 K under the NVT ensemble using a Langevin thermostat^66,67^ (collision frequency of 1 ps^–1^) over 250 ps, with a mild harmonic restraint of 50 kcal mol^–1^ Å^–2^ on the solute atoms. Periodic boundary conditions were applied in all simulations. Long-range electrostatic interactions were computed using the particle mesh Ewald (PME)^68,69^ method with a 10 Å cutoff for both van der Waals and electrostatic interactions. Hydrogen atoms were subjected to SHAKE^70^ constraints, allowing a 2 fs integration time step. Following heating, the system was equilibrated in multiple stages. Initially, a 1 ns density equilibration was performed under NPT conditions at 300 K with a weak restraint of 5 kcal mol^–1^ Å^–2^ on the solute atoms to achieve uniform density. Following density equilibration, a 3 ns unrestrained equilibration under NPT ensemble at 300 K and 1 bar was performed. Berendsen barostat^71^ was used to maintain the pressure with a coupling constant of 2 ps. The production simulations were performed for a minimum of 1 μs with a time step of 2 fs at 300 K and 1 bar pressure. The GPU version of the Amber20 package was used for production simulations.^61^ The CPPTRAJ module was used for hydrogen bond analysis.^72^ The principal component analysis (PCA) and dynamic cross-correlation analysis (DCCA) were performed on the backbone atoms using the Bio3D^73,74^ module in the R programming environment, focusing on the equilibrated portions of the production trajectories.

### 2.3 QM/MM Calculations

Representative snapshots from the production MD simulations were obtained for QM/MM calculations. We retained the water solvation layer up to 12 Å from each atom on the protein surface. The QM/MM calculations were implemented in the ChemShell^75^ software package by using Turbomole^76,77^ for QM theory and DL_POLY^78,79^ for the MM calculations. The active site, including the Fe center coordinated by two histidines and one aspartate/chloride, succinate/2OG, oxygen/superoxo, along with the l-Lys substrate, was included in the QM region depending on the system under consideration. The overall charge on the QM region is neutral (0). The unrestricted B3LYP functional was used to model the QM region, as it has proven efficient in calculations involving Fe-containing enzymes.^80–84^ The region within 8 Å from the QM region was considered as the flexible MM region, while the rest of the system remained fixed. The Amber ff14SB^64^ force field was used for modeling the MM region. Hydrogen link atoms were used as a cap for the QM/MM boundary using the charge shift model.^85^ The effect of the MM region was accounted for in the QM region using an electrostatic embedding scheme.^85^ The geometry optimizations and frequency calculations were carried out using the def2-SVP,^86^ basis set (QM­(B1)/MM). Experimental studies demonstrated the quintet state as the ground state for non-heme Fe enzymes.^42,87–94^ Multiple computational studies on non-heme halogenases have shown that, despite the possibility of triplet, quintet, and septet spin states, the reaction happens through the quintet state.^22,26,88–90,95^ Hence, we considered a HS quintet state (Fe­(III)-OO^•–^, Fe­(IV)=O) for modeling the systems in this study. From the optimized reactant complexes (RCs), the reaction paths were modeled to search for transition states (TS) along a specific reaction coordinate appropriate for the modeled reaction with a step size of 0.1 Å, and finally to obtain the intermediates (IM) and product complexes (PC). Highest energy points along the potential energy surface (PES) were optimized without any restrictions using the dimer^96^ method implemented in the DL-FIND optimizer.^97^ Numerical frequency calculations were performed to confirm the minima and TS states. Single-point energy calculations were performed on the optimized geometries using a large def2-TZVP basis set (QM­(B2)/MM).^86^ The zero-point energy corrections obtained from frequency calculations were added to the B2 energy to get the zero-point corrected QM­(B3)/MM energy. All the discussions were based on the QM­(B3)/MM level of energies. The TITAN^98^ software package was used for the calculation of the intrinsic electric field (IEF) along different bonds. The coordinates and the absolute energies of the QM/MM optimized geometries are given in the SI.

### 2.4 QM/MM MD Simulations

QM/MM MD simulations were performed using the same starting structures employed for the QM/MM calculation in the BesD. QM/MM Born–Oppenheimer MD simulations were carried out using the CP2K 6.1 package, employing the QUICKSTEP module for the QM region and the FIST module for the MM region.^99–101^ The QM region was defined consistently as in the QM/MM calculations. The QM region was treated at UB3LYP with D3 dispersion correction, employing the dual basis set of Gaussian and plane-waves (GPW) formalism.^100^ The Gaussian double-ζ valence polarized (DZVP) basis set^102^ was used for the wave function expansion, and the auxiliary plane-wave basis set with a density cutoff of 360 Ry and GTH pseudopotentials.^103^ The hydrogen link atom method was used to complete the valences of the bonds at the QM-MM boundary.^85^ The remaining parts of the systems, other than the QM regions, were treated at the MM level using the same parameters as in the classical MD simulations. Hartree–Fock exchange calculations within UB3LYP were accelerated using the auxiliary density matrix method (ADMM).^104^ All QM/MM MD simulations were performed in the NVT ensemble with a time step of 0.5 fs and for a total of 4 ps.

## 3 Results and Discussion

### 3.1 Conformational Dynamics of Fe­(IV)=O Intermediate in the BesD Enzyme with Lysine Substrate

We explored the formation of the inline Fe­(IV)=O intermediate from the Cl–Fe­(III)-OO^•–^ complex, modeled based on the crystallographic structure of BesD. A detailed analysis of the Cl–Fe­(III)-OO^•–^ intermediate dynamics and the O_2_ activation reaction mechanism is provided in the SI (Figures S2–S4). To further investigate substrate binding and the conformational dynamics of BesD with the l-Lys substrate at the Fe­(IV)=O intermediate state, we performed MD simulations. These simulations yielded a well-equilibrated trajectory of the BesD–Fe­(IV)=O•l-Lys complex (Figure S5). Here, “•” is part of the nomenclature of the complex, while radical species are indicated in superscript. Analysis of the MD trajectory revealed that the protonated amino group of the l-Lys substrate form stable salt bridge interactions with residues D239, D143, and E123. Furthermore, the substrate’s carboxylate group engages in hydrogen bonding interactions with R77, H137, and W241 (Figure S6), stabilizing l-Lys in the active site. The 1 μs trajectory also revealed that the substrate adopts two conformations: one with the C4-carbon hydrogens (the halogenation target) oriented toward the Fe center (Conf A), and another with the hydrogens oriented away (Conf B). The average distance between the ferryl oxygen and the C4-carbon was 4.45 Å. Conf A was the most populated, occurring in 77.5% of the trajectory (Figure S7). In Conf A, the *pro-R* hydrogen of the C4-carbon is closer to Fe­(IV)=O, whereas in Conf B, both *pro-R* and *pro-S* hydrogens are further from the Fe­(IV)=O. The average O–H distance for the *pro-R* hydrogen was 3.86 Å, while that for the *pro-S* hydrogen was 4.94 Å. Based on this conformational analysis, we suggest that Conf A represents the more stable conformation, which also predicts a favorable *pro-R* hydrogen atom abstraction consistent with experimental observations.^9^ Succinate forms hydrogen-bonding interactions with R218 via its noncoordinating carboxylate, while the Fe-bound carboxylate establishes hydrogen bonds with N222 through its nonbonded oxygen (Figure S8), stabilizing the monodentate coordination mode of succinate within the BesD active site.^51^ Additionally, the iron-binding histidines, H207 and H140, form strong hydrogen bonds with each other, as well as with G138 and T205, respectively, further supporting the conformational sampling of the catalytic center to facilitate efficient catalysis (Figure S8).

We further performed PCA to investigate the flexible motions of the BesD enzyme. The PCA revealed that the region spanning residues 226–255 (region (i)) exhibited the greatest flexibility during the MD simulation (Figure 2a). The residues involved in substrate stabilization, such as D239 and W241, are part of this region (Figure S6). Furthermore, the N-terminal region comprising residues 1–10 (region (ii)) also exhibits high flexibility, which contributes to the interdependent motions observed in the enzyme. DCCA revealed that regions (i) and (ii) are anticorrelated with each other (Figure 2b). Region (i) also shows anticorrelated motion with residues 162–185 (region A). Additionally, the l-Lys substrate is highly correlated with the region (i), highlighting the role of this region in efficient substrate binding. Region A surrounds the DSBH fold, which contains the active site. Region (ii) exhibits negatively correlated motion with residues 80–130 (region B) (Figure 2b). Region B includes the substrate-stabilizing residue (E123) (Figure S6) and also encompasses part of the active site from the N-terminal side. Overall, the observed correlated and anticorrelated motions suggest how different regions of the protein, including active-site residues, the SCS, and LR residues, dynamically influence the catalytic mechanism. In particular, the strong correlation between region (i) and the l-Lys substrate may help stabilize the substrate orientation required for efficient catalysis. Similarly, the observed anticorrelated motions between regions (i), (ii), and the active-site surrounding regions (A and B) may also help stabilize the Fe­(IV)-coordinating residues and substrate binding, thereby facilitating catalysis (Figure 2).

**Figure 2:**
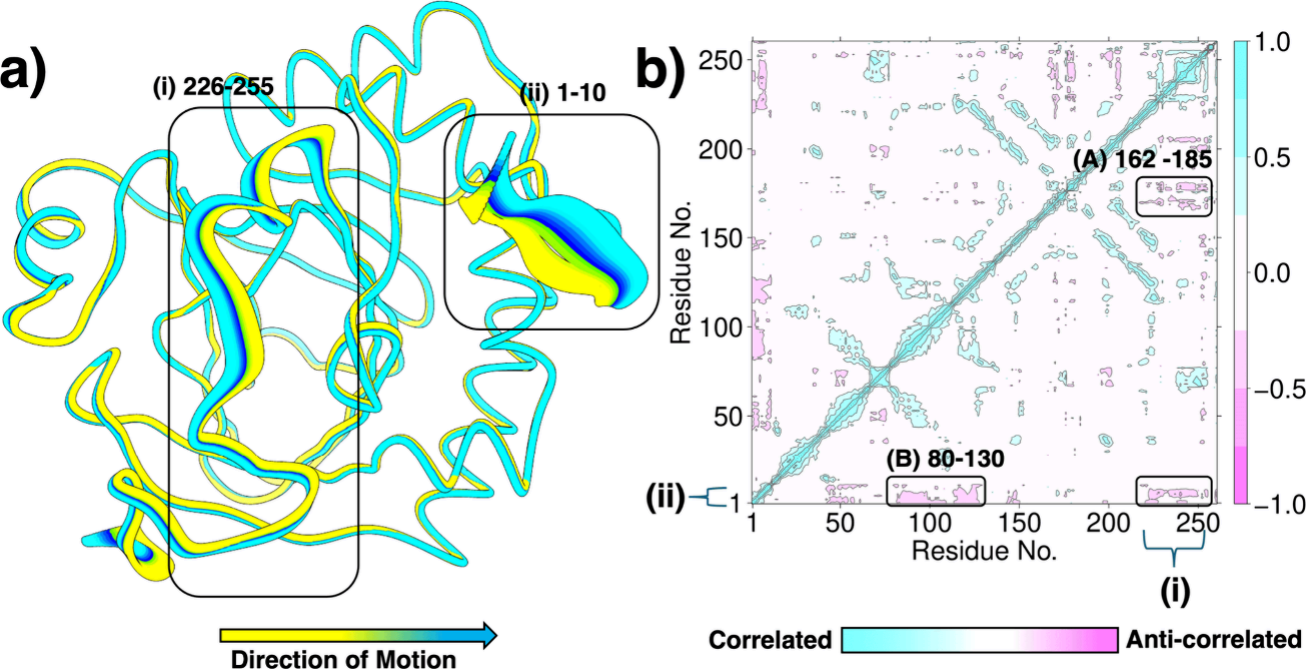
Conformational analysis of BesD-Fe­(IV)=O-l-Lys system. (a) PCA showing the flexible regions of the enzyme with boxed regions highlighting the most flexible regions. (b) DCCA plot showing the correlated and anticorrelated motions existing between different regions of the enzyme. Boxed regions show regions that exhibit high anticorrelated motion.

### 3.2 Reaction Mechanism of BesD Enzyme

Multiple experimental and computational studies have proposed different mechanistic pathways for the halogenation of substrate by non-heme Fe­(II)/2OG halogenases.^22,27,31,50,51,105^ However, a consensus mechanism has not yet been established. To investigate the halogenation mechanism in BesD, we performed QM/MM calculations on snapshots extracted from MD simulations of the BesD-Fe­(IV)=O•l-Lys system. Snapshots were obtained from different times of the MD trajectory to ensure statistical significance.

#### 3.2.1 Catalytic Mechanism of Hydrogen Atom Transfer in BesD

To investigate the HAT mechanism, we optimized five MD snapshots using the QM/MM, yielding five RCs designated as BesD1-RC, BesD2-RC, BesD3-RC, BesD4-RC, and BesD5-RC. The distance between the ferryl oxygen (O) and substrate *pro-R* C4 hydrogen (H) ranges from 2.92 to 3.25 Å, while the distance between Cl and the C4 carbon ranges from 3.71 to 4.37 Å across the five RCs. The Fe–O bond length was 1.61 Å for all five RCs, consistent with previous computational and experimental studies on ferryl complexes of non-heme Fe­(II)/2OG halogenases.^22,26,27,39^ Experimental studies on non-heme Fe­(II)/2OG oxygenases, including halogenases, have shown that substrate hydroxylation or halogenation is initiated by hydrogen abstraction from the target atom by the Fe­(IV)=O complex.^52,106^ To investigate HAT, we performed QM/MM PES scans starting from the five RCs. The activation energies for HAT ranged from 17.5 to 25.0 kcal/mol, with a Boltzmann-averaged value of 18.4 kcal/mol, consistent with previous experimental and computational studies (Figure 3).^51,52,87,105,107^ To analyze whether the initial conformations used for the QM/MM calculations, obtained from well-equilibrated classical MD simulations, can interconvert, we performed five independent QM/MM MD simulations of 4 ps each, utilizing the snapshots used for the QM/MM calculations in the BesD system. Further, analysis of the QM/MM MD trajectories, together with classical MD simulations, was performed using clustering based on the distance between the ferryl oxygen and the C4 carbon of l-lysine (Figure S9). The clustering analysis employed the distance between the ferryl oxygen and the C4 carbon of l-lysine as the structural descriptor, which captures the key reactive coordinate for the HAT step. The cluster analysis and FeO–C distance plots (Figures S9 and S10) show a tendency of trajectories to move toward shared regions, suggesting an emerging trend of interconversion among the initial structures. At the same time, longer QM/MM MD simulations would be required to determine whether full interconversion occurs. Among the RCs, BesD4-RC exhibited the lowest activation energy barrier of 17.5 kcal/mol, with an O–H distance of 2.96 Å and a Cl–C4 distance of 3.83 Å. In BesD4-RC, HAT proceeds through **a TS**, BesD4-TS1, characterized by an O–H distance of 1.29 Å, a Fe–O bond length increase to 1.76 Å, and an Fe–O–H angle of 135.1°. Following HAT, the Fe center forms the Cl–Fe­(III)–OH complex (BesD4-IM1) with an energy of 1.7 kcal/mol relative to BesD4-RC, where the O–H distance decreases to 0.96 Å, the O–C4 distance is 3.29 Å, and the Cl–C4 distance is 4.07 Å (Figure 3). Across all five RCs, the O–H distance at the HAT TSs varies from 1.29 to 1.35 Å, while the Fe–O–H angle ranges from 126.2° to 139.9°. Notably, the TSs with larger Fe–O–H angles, specifically BesD1-TS1 (139.9°) and BesD2-TS1 (139.6°), exhibit higher activation barriers, with calculated HAT barriers of 25.0 kcal/mol for both TSs. Although some studies have suggested that larger Fe–O–H angles facilitate lower HAT barriers, other computational studies have reported only minor differences in the activation energy.^22,52,108–112^ Additionally, previous studies have shown that SCS and LR residues can significantly influence the activation barrier for the HAT reaction.^40,113^ Analysis of HAT TSs indicates that the steric interactions of SCS residues R77, H137, D143, and W241 influence the Fe–O–H angle and thereby the activation barrier. Furthermore, energy decomposition analysis (EDA) reveals that the SCS residue E123, which forms a salt-bridge interaction with the substrate, and the LR residue R218 contribute to the destabilization of the HAT TS in BesD1 and BesD2. The EDA also shows that the SCS residue H137 contributes to the destabilization of TS1 in most snapshots, except for BesD4, where H137 stabilizes the TS (Figure S11). Differences in steric interactions within the active site, together with the energetic contributions of specific SCS and LR residues that stabilize or destabilize the TS and their correlated motions, may therefore contribute to the higher activation energies observed for BesD1 and BesD2 despite their relatively larger Fe–O–H angles. Spin density analyses indicate that the HAT reaction proceeds via the σ-pathway in all five snapshots. For example, in BesD4-RC, the spin density on Fe and O of the Fe­(IV)=O intermediate was 3.07 and 0.69, respectively (Figure S12). Upon HAT, the spin density on Fe increases to 3.96, while a negative spin density appears on the C4 carbon of the l-Lys substrate (−0.37) at BesD4-TS1, consistent with α-electron transfer from σ_C–H_ to the d_*z*_2. In BesD4-IM1, the spin density on Fe is 4.18, and that on the C4 carbon is −0.96, indicating the formation of an Fe­(III) center and substrate-based radical (Figure S12). These observations are in agreement with previous reports on non-heme Fe­(II)/2OG-dependent halogenases.^22,26,31,39^ Additionally, further test calculations utilizing the lowest-energy snapshot of the BesD system (BesD4) were performed for the HAT reaction to evaluate (Table S1): (i) an increased QM region including two Arg residues (R218 and R77), which form salt-bridge interactions with the C5 carboxylate group of succinate and the substrate, and N222, which forms a hydrogen-bonding interaction with the noncoordinating carboxylate oxygen of succinate; (ii) a 12 Å MM region; (iii) inclusion of dispersion corrections; and (iv) the use of the def2-TZVP basis set.

**Figure 3:**
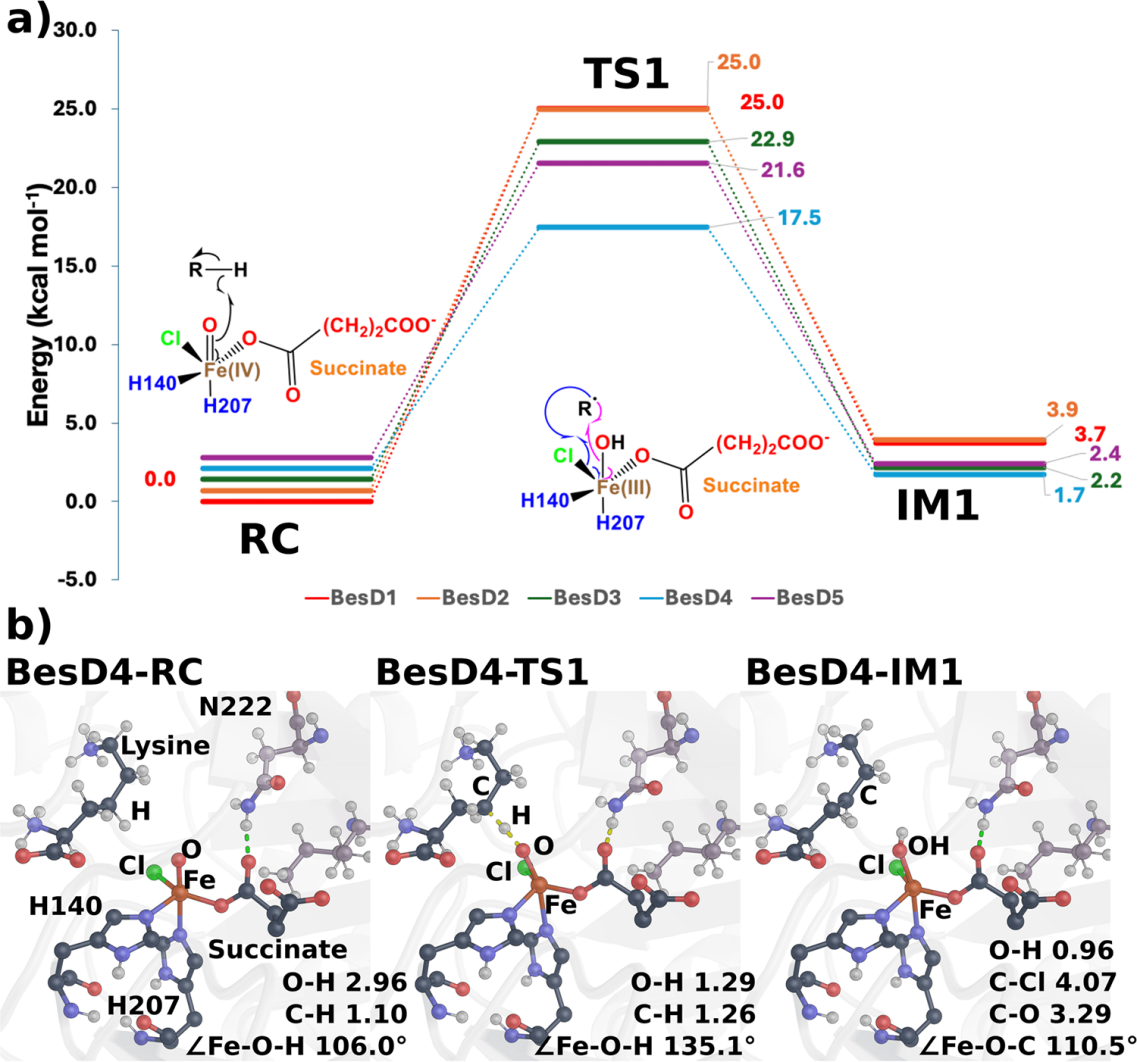
(a) QM/MM reaction profiles of HAT reactions in BesD, (b) QM/MM optimized structures obtained from HAT reaction starting from BesD4-RC. Distances are mentioned in Å, and energies are mentioned in kcal/mol at the QM­(B3)/MM level.

The results reveal that inclusion of N222, both individually and in combination with the two Arg residues (R77 and R218), increases the activation energy by approximately 4 kcal/mol, highlighting some influence of the choice of the QM region on the reaction energetics; however, the mechanistic conclusions of the study remain unchanged (Table S1). The other test calculations show comparable trends in the activation and reaction energies relative to the standard system (Table S1). Specifically, for the calculations involving the def2-TZVP basis set, the resulting activation and reaction energies (22.2/2.0 kcal/mol) closely resemble the corresponding energies obtained at the B2 level (22.2/1.5 kcal/mol), suggesting that the use of the def2-TZVP basis set minimally influences the energetics of the reaction (Table S1).

#### 3.2.2 Reaction Mechanism of Post-HAT Step

After formation of the Cl–Fe­(III)–OH intermediate, the system can follow two possible reaction pathways: rebound hydroxylation, in which the hydroxo group binds to the substrate C4 carbon radical (C4) to produce a hydroxylated product, or halogenation, in which the −Cl ligand binds to the carbon radical to yield a halogenated substrate.^10,17^ At the IM1 states of the five RCs, the distance between the substrate carbon radical and the hydroxo oxygen (O) ranges from 3.29 to 3.66 Å, whereas the C4–Cl distance ranges from 3.77 to 4.50 Å. Due to difference in the spatial orientation of the −OH and −Cl groups relative to C4, the corresponding activation energies for hydroxylation are lower, ranging from 8.4 to 19.8 kcal/mol, with a Boltzmann averaged value of 9.1 kcal/mol, compared to chlorination, which requires activation energies of 11.1–23.7 kcal/mol, with a Boltzmann averaged value of 12.0 kcal/mol. For the snapshot with low HAT barrier, BesD4-RC, the hydroxylation barrier was 8.4 kcal/mol, corresponding to a C4–O distance of 3.29 Å at BesD4-IM1, whereas the chlorination barrier was 15.6 kcal/mol, with a C4–Cl distance of 4.07 Å at BesD4-IM1 (Figure 4). These calculations indicate that, from the as-formed Cl–Fe­(III)–OH complex after HAT, hydroxylation is favored over chlorination, which is contrary to the experimental observation.^9^ Hence, we hypothesize that additional conformational changes are required to facilitate halogenation, as suggested by several computational studies on non-heme Fe­(II)/2OG-dependent halogenases, including SyrB2 and BesD.^31,51^

**Figure 4:**
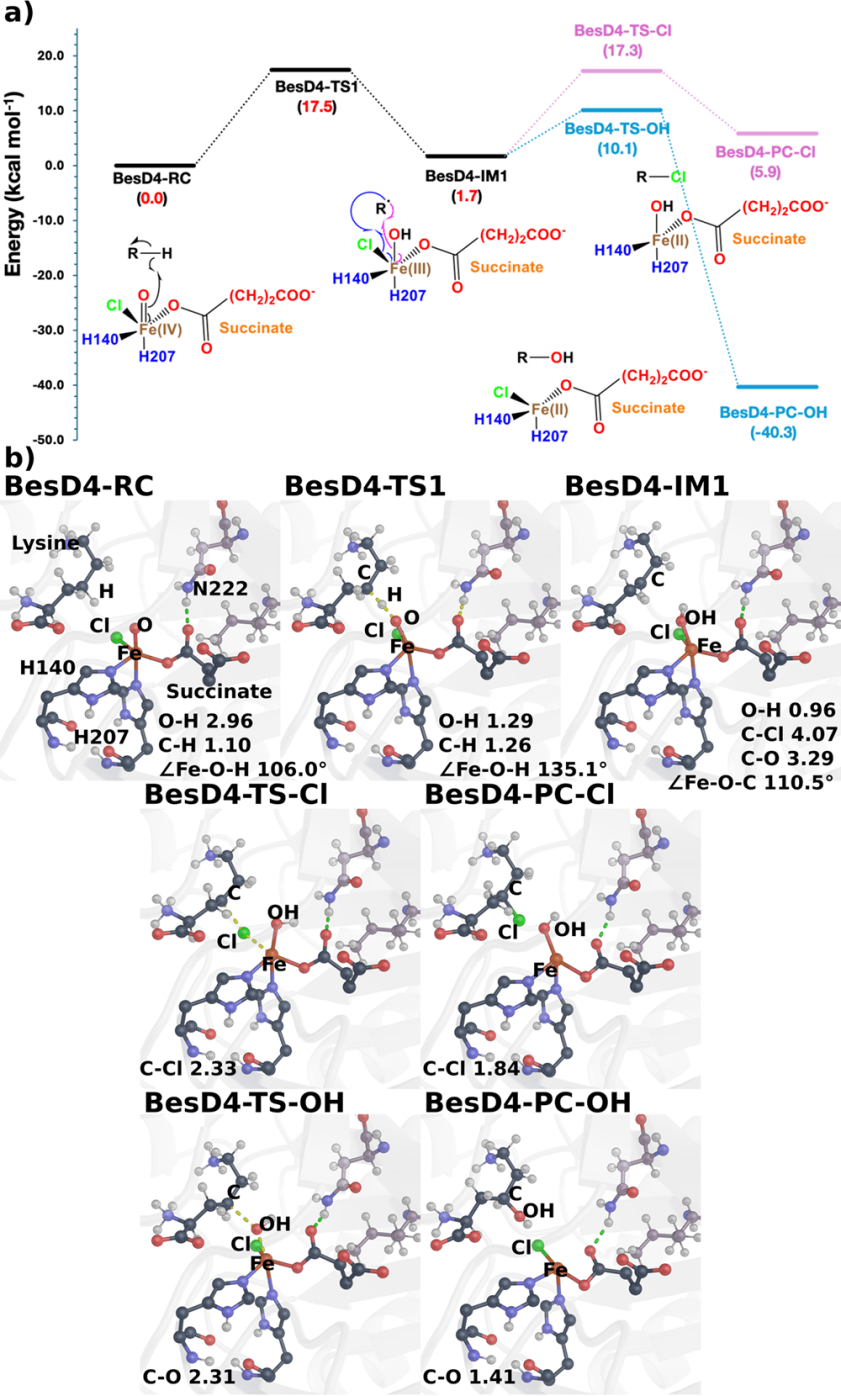
(a) Reaction profile of hydroxylation and chlorination reactions obtained from BesD4-RC, (b) QM/MM optimized structures of stationary points obtained from BesD4-RC. Distances are mentioned in Å, and energies are mentioned in kcal/mol at the QM­(B3)/MM level.

#### 3.2.3 Conformational Isomerization of Cl–Fe­(III)–OH Complex

We explored the conformational isomerization of the Cl–Fe­(III)–OH complex. First, we examined the isomerization of the Fe­(III)–OH species, in which the −OH group shifts from being *trans* to H207 to *trans* to H140. The conformational isomerization required activation barriers ranging from 1.6 to 10.8 kcal/mol, with a Boltzmann-averaged value of 2.5 kcal/mol across the five RCs. These barriers are comparatively lower than the hydroxylation barrier observed prior to isomerization. Additionally, the resulting IM1 intermediate formed after the catalytically important HAT reaction is nearly thermoneutral or only slightly endothermic, with reaction energies ranging from 1.7 to 3.9 kcal/mol across the five snapshots (Figure 3). The observed reaction energies suggest that the formed IM1 intermediate is sufficiently stable to selectively undergo either the canonical rebound hydroxylation or the subsequent isomerization reaction. Specifically, in BesD4-IM1, isomerization proceeds with a barrier of 6.7 kcal/mol through BesD4-Iso1-TS to form BesD4-Iso1-IM, which is lower than the 8.4 kcal/mol barrier for hydroxylation before isomerization (Figure 5). We further explored chlorination and hydroxylation from BesD4-Iso1-IM. During hydroxylation, the −OH group returns to its original *trans* conformation relative to H207, whereas during chlorination, the −Cl group shifts from *trans* to succinate (∠N–Fe–Cl (N-nitrogen of H207 = 88.8°) to a conformation nearly *trans* to H207 (∠N–Fe–Cl = 135.2°) at BesD4-Iso1-TS-Cl, with an activation barrier of 6.7 kcal/mol (Figure 5). We hypothesize that the isomerization reaction may involve an additional step, and therefore we performed PES scans for the second isomerization, in which the -Cl group moves from *trans* to succinate to a configuration *trans* to H207. The activation barriers for this second isomerization ranged from 3.1 to 4.3 kcal/mol, with a Boltzmann average of 3.5 kcal/mol, which is lower than the Boltzmann-averaged barrier of 5.6 kcal/mol for chlorination from the intermediates (Iso1-IMs) obtained after the first isomerization across the five RCs. Specifically, in BesD4-Iso1-IM, the second isomerization proceeded with a small barrier of 2.7 kcal/mol through BesD4-Iso2-TS to form BesD4-Iso2-IM (Figure 5). Notably, during the second isomerization, the −OH group shifts to a position *trans* to succinate (∠N–Fe–O of 80.9°). Overall, the isomerization involves two sequential steps that switch the positions of -Cl and −OH. At BesD4-Iso2-IM, the C4–Cl distance decreases to 3.28 Å, while the C4-OH distance increases to 5.10 Å. Consistent with these distances, chlorination from BesD4-Iso2-IM requires a low barrier of 4.5 kcal/mol, whereas hydroxylation requires 25.6 kcal/mol (Figure 5). Spin density analysis revealed that the spin densities on the −OH and −Cl groups change upon isomerization. At BesD4-IM1, the spin densities on O and Cl were 0.31 and 0.14, respectively. After two isomerization steps, these values changed to 0.25 and 0.20, respectively. The increase in spin density on the −Cl group (0.14–0.20) upon isomerization may contribute to the low barrier for chlorination from BesD4-Iso2-IM (Figure S13). Similarly, in BesD3-RC, HAT required a barrier of 22.9 kcal/mol to form BesD3-IM1, from which the first isomerization step required 6.7 kcal/mol to form BesD3-Iso1-IM (Figure S14). Chlorination from BesD3-Iso1-IM required a 5.5 kcal/mol barrier, whereas the second isomerization step required only 4.1 kcal/mol to form BesD3-Iso2-IM, from which chlorination required only 4.8 kcal/mol (Figure S14). The spin density changes on −OH and −Cl during isomerizations in BesD3-RC were consistent with those observed in BesD4-RC (Figure S15). In BesD5-RC, the initial HAT required 21.6 kcal/mol to form BesD5-IM1, from which hydroxylation was preferred over chlorination. The first isomerization step required only 1.6 kcal/mol to form BesD5-Iso1-IM. During subsequent chlorination and hydroxylation attempts, the system underwent a complete second isomerization to form BesD5-Iso2-IM, from which chlorination required only 3.3 kcal/mol, while hydroxylation required 21.7 kcal/mol (Figure S16). Based on comprehensive calculations across multiple snapshots, we propose that the isomerization of the Cl–Fe­(III)–OH complex proceeds via two consecutive steps, leading to a complete exchange of the −Cl and −OH coordination positions, rather than a single-step process as previously proposed, in which only the −OH group undergoes a single-step isomerization. In agreement with the previous study,^51^ the activation barrier for chlorination is substantially reduced after the first isomerization. Notably, our calculations show that the subsequent isomerization proceeds with a lower barrier than chlorination from the first isomer, thereby further reducing the overall barrier for the chlorination. However, further experimental validation might be crucial to validate the mechanistic proposal.

**Figure 5:**
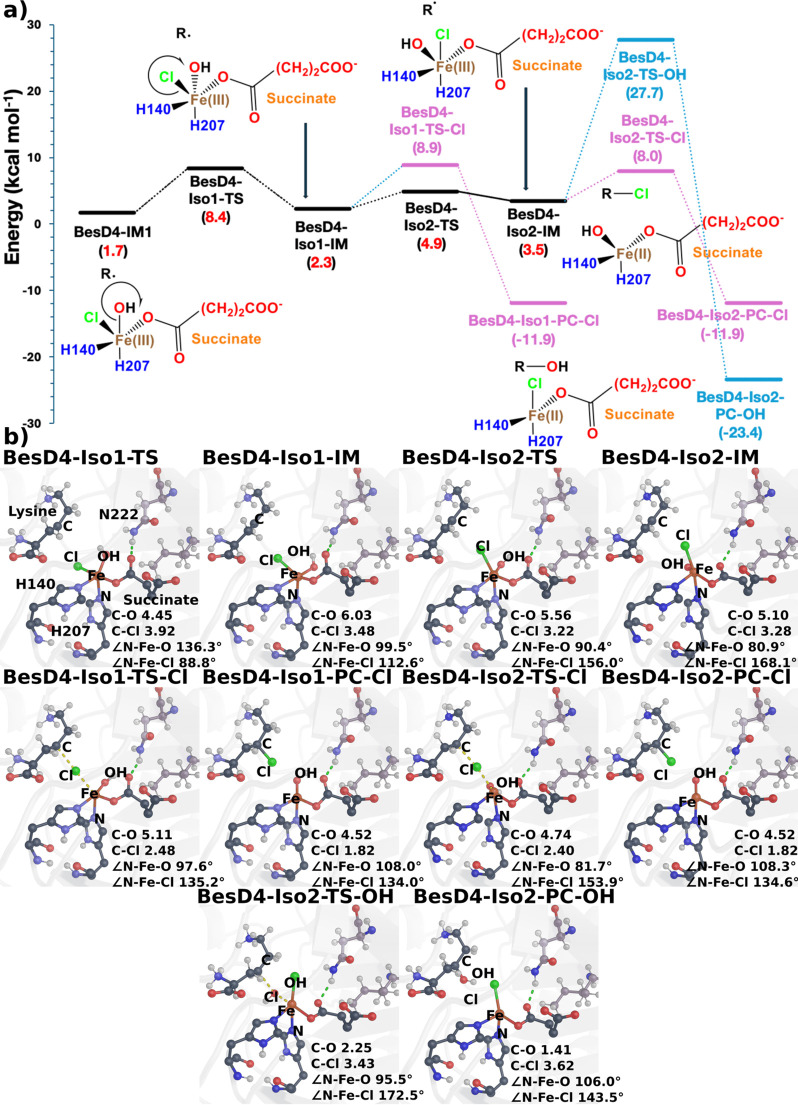
(a) Reaction profile of isomerization, hydroxylation, and chlorination reactions obtained from BesD4-RC, (b) QM/MM optimized structures of stationary points obtained from BesD4-RC. Distances are mentioned in Å, and energies are mentioned in kcal/mol at the QM­(B3)/MM level. The competing hydroxylation pathway from the IM1 intermediate is shown in Figure 4.

#### 3.2.4 Role of the Enzyme Environment in Catalysis

##### 3.2.4.1 Role of Intrinsic Electric Field

IEFs are recognized as a key contributor to the enhanced reactivity and selectivity of enzymatic active sites.^55,114–117^ Therefore, we calculated the IEFs along the Fe-X bonds (X = Cl or O) across multiple catalytic states, including the RC, IM1, Iso1-IM, and Iso2-IM, using representative snapshots for each state. In the five RCs, the IEF along the Fe–O bond ranged from −0.0346 to −0.0359 a.u., whereas the fields along the Fe–Cl bond varied from 0.0037 to 0.0065 a.u. However, the IEF along the Fe–O bond of the Fe­(III)–OH moiety in IM1, Iso1-IM, and Iso2-IM undergoes a substantial change, which may influence the rebound step. For example, in BesD4-RC, the electric field along the Fe–O bond is −0.0289 a.u. in BesD4-IM1, where the −OH group is positioned *trans* to H207. After the first isomerization, the field increases to −0.0227 a.u. in BesD4-Iso1-IM, and following the second isomerization, it increases further to 0.0077 a.u. In contrast, the IEF along the Fe–Cl bond in BesD4-IM1 is 0.0024 a.u., but it decreases upon isomerization to −0.0011 a.u. in BesD4-Iso1-IM and to −0.0236 a.u. in BesD4-Iso2-IM, where the −Cl ligand becomes *trans* to H207 (Figure 6).

**Figure 6:**
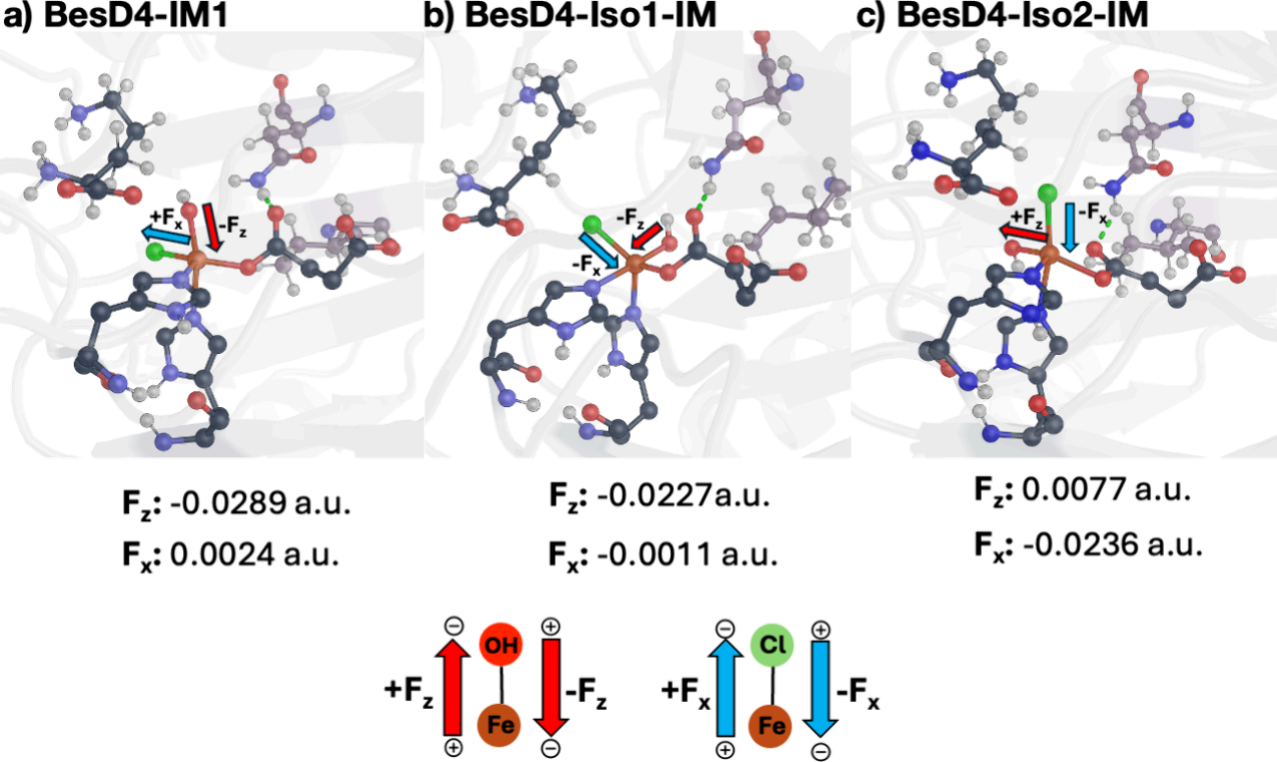
IEF variation along the Fe–Cl and Fe–O bonds in Cl–Fe­(III)–OH isomers of BesD4-RC. The IEF vectors are defined from positive to negative according to the TITAN convention. Red arrows represent the field components, with the *z*-axis aligned along the Fe–O bond and the *x*-axis along the Fe–Cl bond (shown in blue).

These results clearly indicate that the ligand positioned *trans* to H207 consistently exhibits a more negative IEF along the Fe-ligand bond. This change in IEF is also mirrored in the rebound hydroxylation and chlorination barriers. For example, in BesD4-RC, chlorination from BesD4-IM1 (IEF of Fe–Cl 0.0067 a.u.) requires a barrier of 15.6 kcal/mol, whereas chlorination from BesD4-Iso2-IM (IEF of Fe–Cl −0.0236 a.u.) requires only 4.5 kcal/mol. The shift in the IEF along the Fe–Cl bond in BesD4-Iso2-IM also correlates with corresponding changes in spin density on the −Cl ligand, as discussed in the previous section. This redistribution of spin density may facilitate C–Cl bond formation during the chlorination step. Similarly, for hydroxylation from BesD4-IM1, the reaction barrier is only 8.4 kcal/mol when the IEF along the Fe–O bond is −0.0289 a.u., compared with a barrier of 24.2 kcal/mol from BesD4-Iso2-IM, where the corresponding IEF is 0.0024 a.u. Consistent with the changes in the IEF along the Fe–O bond in BesD4-Iso2-IM, the spin density on the O atom decreases from 0.31 in BesD4-IM to 0.25 in BesD4-Iso2-IM. A similar trend is observed in the other BesD RCs (Figure S17). The observed trend in the IEF is consistent with previous computational studies on non-heme Fe­(II)/2OG-dependent halogenases, such as the hectochlorin biosynthesis enzyme HctB,^39^ where protein-induced electric field effects were shown to modulate the charge distribution along the Fe–Cl bond, thereby promoting selective halogenation over hydroxylation.

These results support the conclusion that the IEF plays a major role in governing reaction selectivity, defining the *trans* position to H207 as the preferred orientation of the −Cl ligand for efficient chlorination.

##### 3.2.4.2 Role of SCS and LR Residues

To assess the influence of the enzyme environment on the HAT reaction catalyzed by BesD, we performed EDA.^118^ EDA allowed us to identify residues in the SCS and LR regions that energetically stabilize or destabilize the HAT TSs across the five representative snapshots. In this analysis, we focused on the three residues with the strongest stabilizing contributions and the three with the strongest destabilizing contributions. EDA based on the optimized structures of BesD4-TS1 and BesD4-RC indicated that the residues H137, K173, and R240 contribute to stabilization of BesD4-TS1, whereas N222, D239, and E123 contribute to destabilization (Figures 7 and S18). EDAs based on BesD3-TS1 and BesD3-RC showed that the residues K120, K173, and R240 stabilize BesD3-TS1, while E123, H137, and R218 destabilize it (Figures 7 and S19). Overall, the positively charged residues R240, K173, and K120 consistently stabilize the HAT TS across the five snapshots, whereas residues such as H137, E123, and D239 contribute to destabilization. Positively charged residues surrounding the active site play a major role in the HAT reaction catalyzed by BesD. Consistent with this, all of these residues were predicted to exhibit dynamic correlations with the l-Lys substrate in our MD simulations, as revealed by the DCCA analysis. Thus, we hypothesize that substrate binding and the accompanying conformational adjustments in the BesD protein play a key role in catalysis.

**Figure 7:**
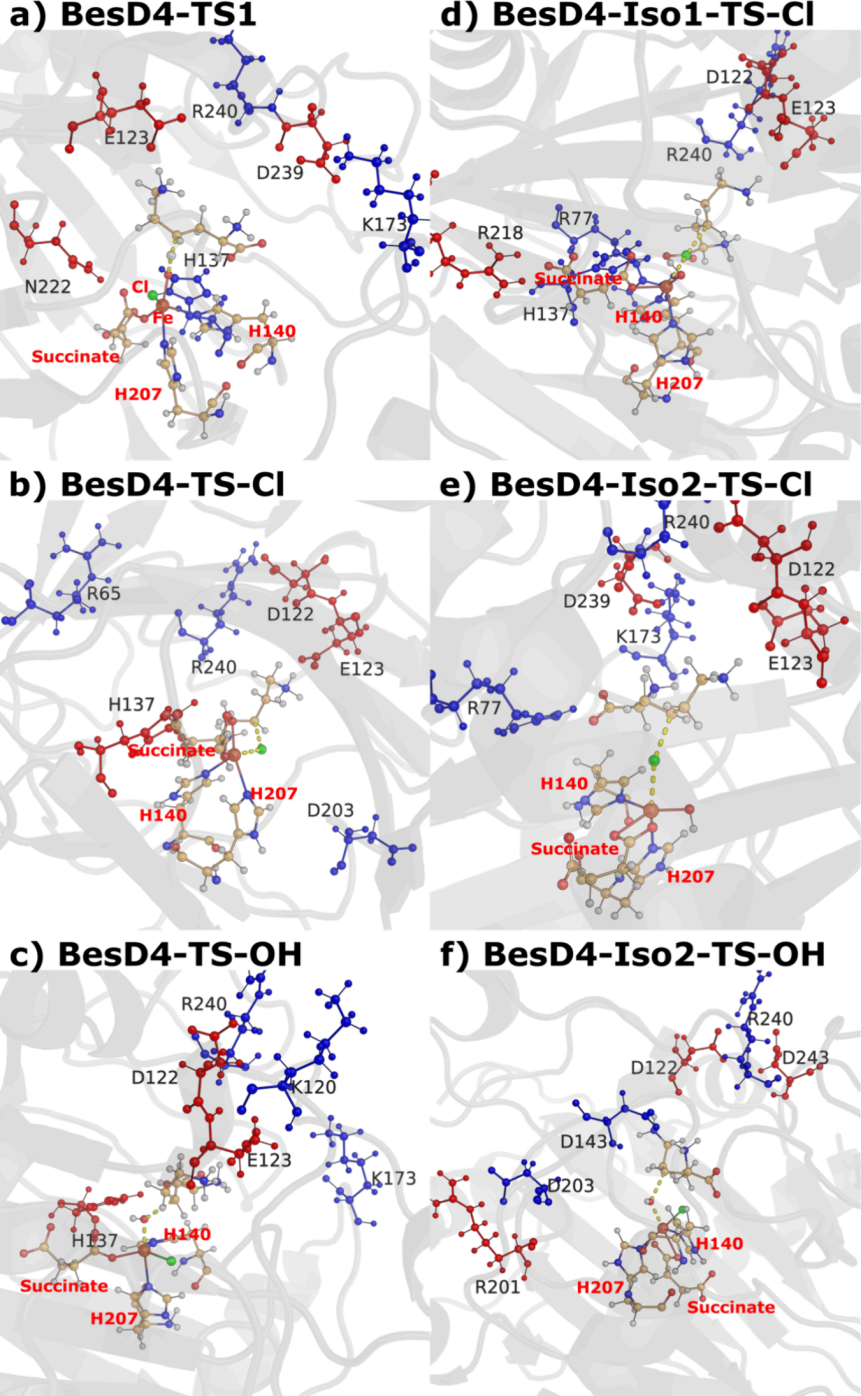
EDA analysis shows the TS stabilizing contributions of SCS and LR residues in HAT (a) and chlorination and hydroxylation processes, initiated from BesD4-IM1 (b,c), BesD4-Iso1-IM (d), and BesD4-Iso2-IM (e,f). The active site residues are labeled in red.

To further probe the roles of active-site, SCS, and LR residues, we performed EDA for the chlorination and hydroxylation reactions catalyzed by BesD. Based on the optimized structures of BesD4-TS-Cl and BesD4-TS–OH relative to BesD4-IM1, the EDA indicates that similar residues influencing the HAT also contribute to the TSs of chlorination and hydroxylation. The destabilizing contributions are larger for BesD4-TS-Cl (H137 (3.28), D122 (2.43), E123 (1.92)) than for BesD4-TS–OH (H137 (2.36), D122(1.86), E123 (1.67)) (Figures 7 and S18). After the first isomerization, however, these destabilizing contributions decrease or even shift to stabilizing effects in BesD4-Iso1-TS-Cl (H137 (−2.22), D122 (1.66), and E123 (2.08)). A similar trend, characterized by a further reduction in destabilization, is observed in BesD4-Iso2-TS-Cl. During isomerization, succinate undergoes a subtle reorientation (Figure 6), which likely alters the positions and interactions of SCS and LR residues. Indeed, the residues contributing to TS stabilization or destabilization display correlated motions with succinate. Thus, the combined EDA and DCCA analyses suggest that the observed correlated motions between the EDA residues and the substrate, succinate, and the other SCS/LR residues likely contribute to TS stabilization by enabling LR dynamic coupling.

##### 3.2.4.3 Effects of Isomerization in the Electronic Structure of BesD

To elucidate the electronic factors governing chlorination selectivity during the isomerization of the Cl–Fe­(III)–OH intermediate in BesD, we performed Frontier Molecular Orbital (FMO) analysis. We analyzed three isomeric intermediates derived from BesD4-RC: BesD4-IM1, BesD4-Iso1-IM, and BesD4-Iso2-IM. FMO analysis of BesD4-IM1 indicated that the electronic configuration is 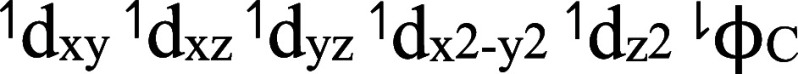 as HAT proceeded through the **σ-**pathway via α-electron transfer from the substrate σ_CH_ orbital to the d_*z*_^2^ orbital of the Fe­(IV)=O center (Figure 8). The SNO and NO analyses also support the proposed electron transfer pathways during HAT (Figure S20). The FMO analysis of the as-formed BesD4-IM1 intermediate shows that the LUMO corresponds to the β orbital of the d_*xy*,_ with the LUMO+1 and LUMO+2 corresponding to the β orbitals of the d_*xz*_ and d_*yz*_, respectively (Figure S20). The FMO energy gap between d_*xy*_ and d_*yz*_ is −0.251 eV (−5.7 kcal/mol), indicating stabilization of the d_*xy*_ (Figure 8). Consistent with the SNO analysis, hydroxylation from BesD4-IM proceeds via electron transfer from ϕ_C_ to the LUMO (d_*xy*_)_,_ resulting in a low barrier of 8.4 kcal/mol (Figure S20). In contrast, chlorination requires electron transfer from ϕ_C_ to the d_*yz*_ orbital (LUMO+2), leading to a higher barrier of 15.6 kcal/mol (Figure S20, Note: During the chlorination step, the d_*yz*_ orbital becomes the LUMO along the chlorination pathway). After two isomerization steps, in the BesD4-Iso2-IM state, the ordering of the frontier orbitals changes: d_*xz*_ becomes the LUMO, d_*yz*_ becomes the LUMO+1, and d_*xy*_ becomes the LUMO+2 (Figure 8). The FMO energy difference between d_*xy*_ and d_*xz*_ shifts to +0.373 eV (8.57 kcal/mol), stabilizing the d_*xz*_ orbital (Figure 8). As a result, chlorination from BesD4-Iso2-IM involves electron transfer from ϕ_C_ to the d_*xz*_ LUMO and proceeds with a low barrier of 4.5 kcal/mol (Figure S20). Conversely, hydroxylation now requires transfer from ϕ_C_ to the d_*xy*_ (LUMO+2) orbital, which exhibits a substantially higher barrier of 24.2 kcal/mol (Figure S20). Repeating the FMO, NO, and SNO analysis on the isomers of the Cl–Fe­(III)–OH intermediate obtained from BesD3-RC (Figures S21 and S22) revealed the same qualitative trends: isomerization reorganizes the d-orbital manifold in a manner that promotes chlorination over hydroxylation. Overall, the MD-based QM/MM calculations show that, in addition to the steric effects demonstrated in previous studies,^51^ the combined influence of the protein environment, including correlated motions, electronic structure, and IEF, governs the selectivity between the canonical rebound hydroxylation and chlorination pathways.

**Figure 8:**
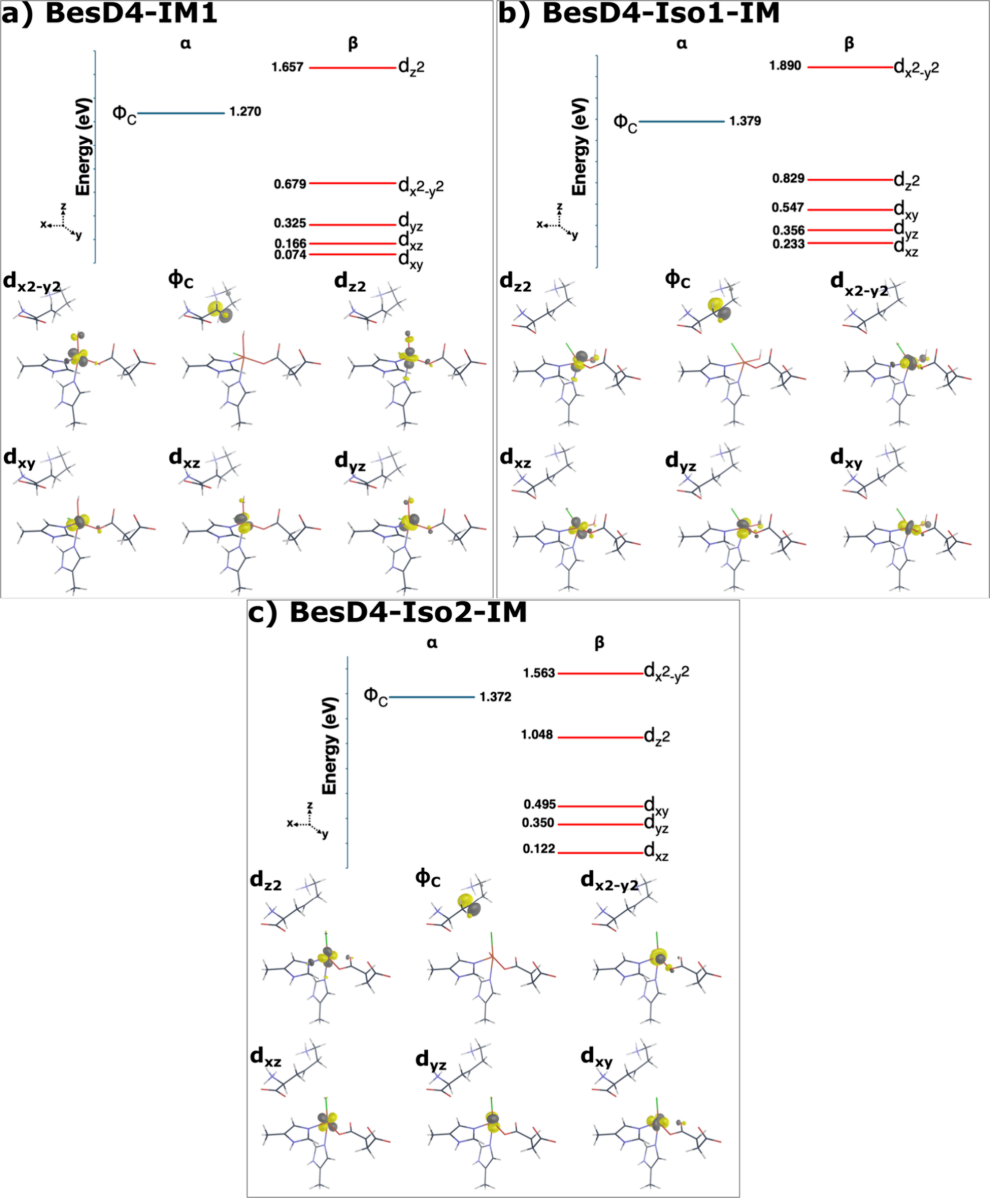
Frontier molecular orbital (FMO) analysis of the isomers of Cl–Fe­(III)–OH intermediates obtained from BesD4-RC, (a) BesD4-IM1, (b) BesD4-Iso1-IM, and (c) BesD4-Iso2-IM.

### 3.3 Insights into Hydroxylase to Halogenase Engineering

Recently, using BesD as a starting point, a homologous lysine hydroxylase (Hydrox) was identified through a phylogenetic analysis of the BesD family. This enzyme crystallized and was subsequently engineered into a halogenase by modifying key residues that govern reaction selectivity.^29^ In this study, we investigated Hydrox, along with two engineered variants, Hydrox-3R, which contains three residue substitutions (D142G, N149I, V223N), and Chimera14, which contains 14 substitutions, using MD-based hybrid QM/MM calculations. Our objective was to elucidate how SCS and LR residues modulate reaction selectivity and to define the molecular basis underlying the successful conversion of a hydroxylase into a halogenase.

#### 3.3.1 Conformational Dynamics of Fe­(IV)=O Intermediate in Hydrox, Hydrox-3R, and Chimera14

To gain insights into substrate binding and the conformational dynamics of the Fe­(IV)=O intermediate in Hydrox, Hydrox-3R, and Chimera14, we performed MD simulations. These simulations yield well-equilibrated trajectories for all three systems (Figures S23–S25). Overall, MD simulations show that substrate-stabilizing interactions are largely conserved across Hydrox, Hydrox-3R, and Chimera14. However, some differences arise in hydrogen-bonding interactions between the noncoordinating succinate carboxylate oxygen and SCS residues. Detailed analysis is provided in the SI (Page S28; Figures S26–S28).

Substitution in the SCS and LR regions influences both local and broader protein environment by perturbing correlated dynamics. To characterize these dynamic fluctuations among the three systems, we performed PCA to identify the predominant protein motions. In Hydrox, the principal motion primarily involves region (i) (residues 226–239), similar to the dominant motions observed in the native halogenase BesD (residues 226–255). However, region (ii) (residues 1–10) exhibits reduced flexibility relative to BesD, whereas region (A) (residues 170–176) displays increased flexibility, a feature unique to the Hydrox (Figure S29). In the Hydrox-3R variant, flexibility in region (A) is diminished relative to Hydrox, while region (ii) gains flexibility. Notably, the characteristic motions of region (i) are retained (Figure S29). Furthermore, PCA revealed that in Hydrox, regions (i) and (A) move toward each other, whereas in Hydrox-3R, these regions move apart (Figure S29). Because these two regions form a lid over the substrate, their relative motions are likely to influence substrate positioning. In Hydrox, the inward movement of the two loops creates a more closed conformation that stabilizes the orientation of l-Lys with respect to the Fe­(IV)=O (Figure S29). In contrast, the outward motion observed in Hydrox-3R correlates with the substrate adopting a position farther from the Fe­(IV)=O. Following this trend, the motions within region (i) are further enhanced in Chimera14, whereas region (A) exhibits no flexibility and region (ii) shows moderate flexibility relative to Hydrox (Figure S29). Overall, the flexibility patterns in Chimera14 most closely resemble those observed in the native halogenase BesD. To further investigate these dynamic differences, we analyzed correlated and anticorrelated motions using the DCCA method. Consistent with the PCA results, the DCCA revealed distinct correlated motions in Hydrox (Figure S29). Relative to BesD, Hydrox displays markedly reduced anticorrelated motion, with no anticorrelated motion between regions (i) and (ii) and only limited anticorrelation involving residues 170–176 in region (A). Moreover, Hydrox lacks the correlated motion between the l-Lys substrate and region (i) that is characteristic of BesD. In Hydrox-3R, anticorrelation between regions (i) and (A) increases, and a correlated motion emerges between the l-Lys substrate and W242. In Chimera14, anticorrelated motion between regions (i) and (A) becomes even more pronounced, closely mirroring BesD (Figure S29). However, unlike BesD, the l-Lys substrate displays anticorrelated motion with region (i). These differences in correlated and anticorrelated motions involving region (i) between Hydrox and the halogenase variants (Hydrox-3R and Chimera14) influence substrate binding. In the halogenase variants, the l-Lys substrate is positioned further from the Fe center than in Hydrox, consistent with the higher turnover of hydroxylated l-Lys observed experimentally in the Hydrox system.^29^ Thus, to improve the turnover of halogenation, region (i) should be a primary target in enzyme redesign efforts. We further examined the correlated and anticorrelated motions of the individual residues substituted during the conversion of Hydrox into Chimera14. Interestingly, the corresponding residues in Hydrox-F148, N149, G216, A217, V218, M221, V223, V224, S225, S227, and S229 showed no correlation with either succinate or the l-Lys substrate. Upon substitution, however, the analogous residues in Chimera14 (L148, I149, S216, T217, I220, N222, M223, T224, A226, and K231) exhibited clear correlated motions with both l-Lys and succinate. This behavior mirrors the correlated motions observed in the equivalent residues of the BesD. These results suggest that successful engineering of the reaction pathway from hydroxylation to halogenation appears to rely on enhancing correlated dynamics among the SCS and LR residues, the Fe center (including succinate), and the substrate.

#### 3.3.2 How is Hydroxylation Achieved in Hydrox?

A representative snapshot from the MD trajectory of Hydrox was optimized using the QM/MM method to obtain the Hydrox-RC structure. In Hydrox-RC, the distance between the ferryl oxygen (O) and the substrate *pro-R* hydrogen (H) was 2.93 Å, and the substrate C4–H bond length was 1.10 Å. We then performed a PES scan to model the HAT reaction, yielding Hydrox-IM1 via Hydrox-TS1 with an activation barrier of 17.2 kcal/mol (Figure 9). At Hydrox-TS1, the O–H distance was 1.29 Å, the C4–H was 1.25 Å, and the ∠Fe–O–H was 138.4°. In Hydrox-IM1, the O–H distance shortened to 0.96, the C4–H distance increased to 2.69 Å, and the C4–O distance was 3.41 Å. From this Fe­(III)–OH intermediate, we explored the rebound hydroxylation step, which proceeds with a barrier of 5.9 kcal/mol from Hydrox-IM1 through Hydrox-TS2 (Figure 9). At Hydrox-TS2, the C4–O distance is 2.34 Å, which further decreases to 1.42 Å in Hydrox-PC, yielding the hydroxylated l-Lys product (Figure S30). Previous computational studies^92,113^ and our calculations on BesD demonstrate how the conformational variability in the starting structures can influence the calculated activation energies. Therefore, we performed QM/MM calculations utilizing five different snapshots for the HAT reaction. The QM/MM calculations show that the Boltzmann-averaged activation energy for the HAT reaction is 13.0 kcal/mol, with the lowest and highest values being 12.2 and 19.4 kcal/mol, respectively (Figure S31). The low activation barrier for HAT, combined with the closer approach of the C4-carbon to the ferryl center observed throughout the MD trajectory, indicates a higher intrinsic reactivity of the Hydroxyl system, consistent with the experimentally reported high substrate turnover for the hydroxylase variant.^29^ Additional insights from IEF calculations, spin density analyses, and residue-level EDA for the HAT and rebound hydroxylation steps are presented in the SI (Page S33 and Figures S32 and S33).

**Figure 9:**
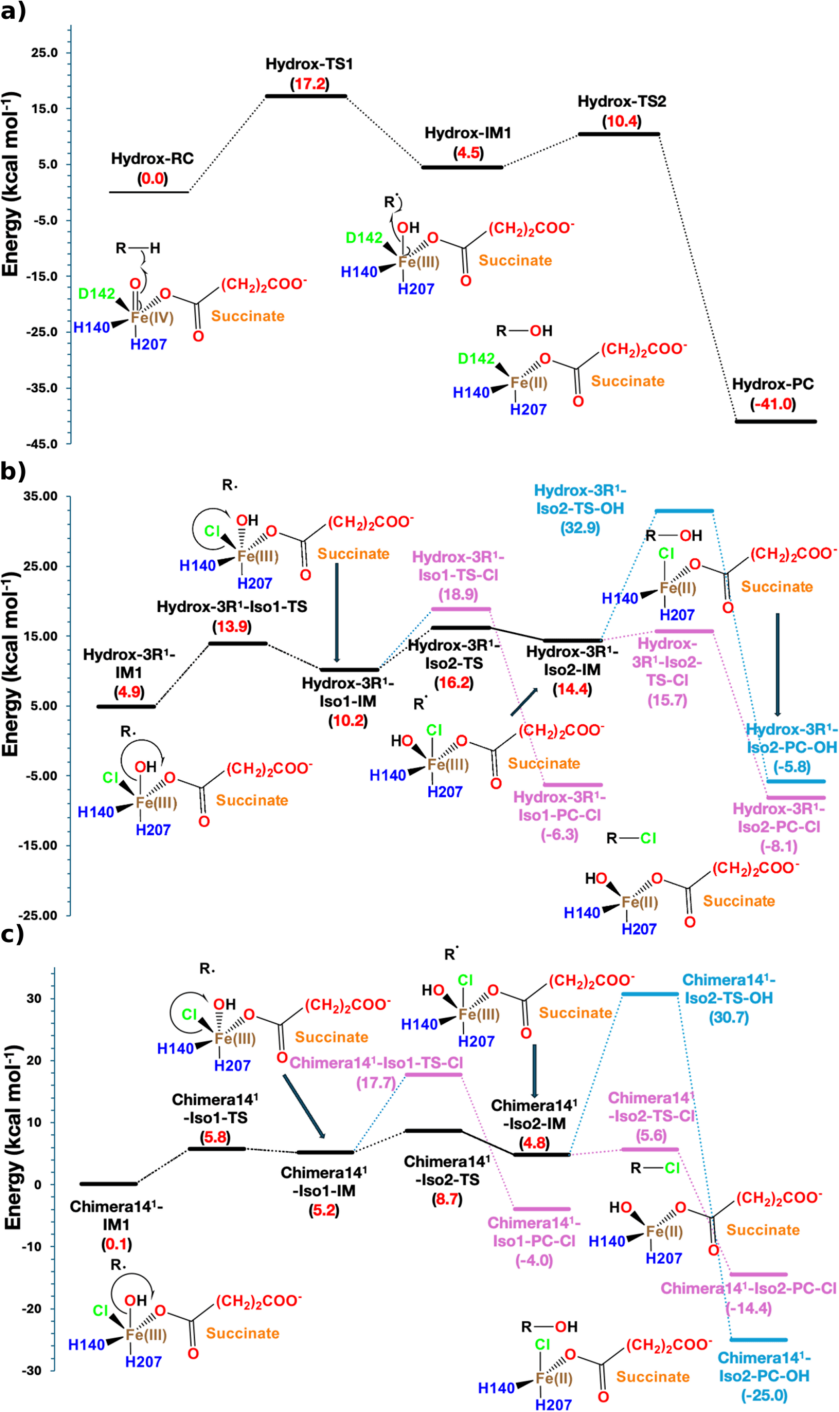
(a) Reaction profile of hydroxylation obtained from Hydrox-RC. Reaction profiles of isomerization, hydroxylation, and chlorination reactions obtained from (b) Hydrox-3R^1^-RC, (c) Chimera14^1^-RC. Energies are mentioned in kcal/mol at the QM­(B3)/MM level. The competing hydroxylation pathway from the IM1 intermediate of Hydrox-3R^1^ and Chimera14^1^ is shown in Figures S34 and S49, respectively.

#### 3.3.3 How is Halogenation Achieved in Hydrox-3R?

We explored the reaction mechanism of Hydrox-3R by extracting a snapshot from the MD trajectory and optimizing it using QM/MM to obtain Hydrox-3R^1^-RC. At this reactant complex, the distance between the ferryl oxygen (O) and the hydrogen (H) of the C4 carbon of l-Lys was 3.25 Å, with an ∠Fe–O–H angle of 119.8° (Figure S34). The HAT reaction proceeded via Hydrox-3R^1^-TS1 with a barrier of 19.0 kcal/mol. Spin density analysis indicated that HAT occurs through a σ-pathway (Figure S35). At Hydrox-3R^1^-TS1, the O–H and C4–H distances reduced to 1.29 and 1.27 Å, respectively. Upon HAT, Hydrox-3R^1^-IM1 is formed, with a C4–Cl distance of 4.50 Å and a C4–O distance of 3.43 Å. As expected, chlorination had a high barrier of 25.0 kcal/mol, whereas hydroxylation required only 11.2 kcal/mol (Figure S34). We then examined the first isomerization step, which required 9.0 kcal/mol to form Hydrox-3R^1^-Iso1-IM, followed by a second isomerization step with a barrier of 6.0 kcal/mol, yielding Hydrox-3R^1^-Iso2-IM (Figures 9 and S36). From Hydrox-3R^1^-Iso2-IM, chlorination proceeded with a low activation barrier of 1.3 kcal/mol, whereas hydroxylation required an activation energy of 18.5 kcal/mol. The TS for chlorination, Hydrox-3R^1^-Iso2-TS-Cl, had an energy of 15.7 kcal/mol relative to Hydrox-3R^1^-RC, which is lower than the hydroxylation TS energy of 16.1 kcal/mol (Figures 9 and S36). IEF calculations along the Fe–O bond in Hydrox-3R^1^-RC gave a negative value of −0.0317 a.u., whereas along the Fe–Cl bond it was positive at 0.0076 a.u. At Hydrox-3R^1^-IM1, the IEF along the Fe–O and Fe–Cl changes to −0.0275 and 0.0085 a.u., respectively (Figure S37). Upon isomerization, the IEF along Fe–O increases to −0.0236 a.u. at Hydrox-3R^1^-Iso1-IM and further to 0.0065 a.u. at Hydrox-3R^1^-Iso2-IM. Similar to BesD, the IEF along Fe–Cl changes to 0.0108 a.u., at Hydrox-3R^1^-Iso1-IM, which further reduces to −0.0260 a.u. at Hydrox-3R^1^-Iso2-IM (Figure S37). Due to changes in the IEF, the spin density of the −Cl ligand increases from 0.16 at Hydrox-3R^1^-IM1 to 0.21 at Hydrox-3R^1^-Iso2-IM (Figure S38), which leads to a lower barrier for chlorination initiated from Hydrox-3R^1^-Iso2-IM. EDA revealed that the residues K173, N223, and R241 are involved in HAT TS stabilization, while different LR residues, such as R66, H136, and D203, are involved in TS stabilization of chlorination, and G142, N223, and R241 are involved in TS stabilization of hydroxylation initiated from Hydrox-3R^1^-IM1 (Figure S39). Residues that destabilized the Hydrox-3R^1^-TS-Cl (D122, W141, and R201) contribute similarly in the Hydrox-3R^1^-Iso1-TS-Cl. However, their destabilizing effects (D122, E123, and W141) were minimized in Hydrox-3R^1^-Iso2-TS-Cl (Figure S40). In the case of Hydrox-3R^1^-Iso2-TS–OH, the residue H137 makes high destabilizing contributions (18.8 kcal/mol), which leads to a high barrier for hydroxylation initiated from Hydrox-3R^1^-Iso2-IM (Figure S40). The FMO and SNO analyses of the three isomeric Cl–Fe­(III)–OH intermediates revealed that from Hydrox-3R^1^-IM1, hydroxylation proceeds via electron transfer from ϕC to the d_*xy*_-based LUMO, giving a low barrier (11.2 kcal/mol), whereas chlorination requires transfer to the d_*xz*_ (LUMO+1), resulting in a higher barrier (25.0 kcal/mol) (Figures S41 and S42). After two isomerizations (Hydrox-3R^1^-Iso2-IM), the d_*yz*_ orbital becomes the LUMO, stabilizing the chlorination pathway and lowering its barrier to 1.3 kcal/mol, while hydroxylation requires transfer to the higher-lying d_*xy*_ (LUMO+2), leading to the barrier of 18.5 kcal/mol (Figures S41 and S42). Hence, like BesD, the Hydrox-3R also follows a two-step isomerization process, which helps in the rearrangement of the d-orbital manifold to help achieve chlorination preference. Furthermore, to evaluate the influence of conformational dynamics, additional QM/MM calculations for the HAT reaction were performed using four new snapshots obtained from MD simulations of the Hydrox-3R mutant. The Boltzmann-averaged activation barrier is 16.6 kcal/mol, with individual barriers ranging from 15.6 to 22.5 kcal/mol (Figure S43). To further understand how the starting geometries influence the subsequent competing hydroxylation and isomerization pathways, we performed additional QM/MM calculations for both reactions. The calculations show that in four out of the five snapshots, isomerization proceeds with a lower activation energy than the rebound hydroxylation. The Boltzmann-averaged activation barriers are 8.2 kcal/mol for hydroxylation (with individual values of 11.2, 7.7, 7.7, 8.6, and 11.2 kcal/mol) and 6.8 kcal/mol for isomerization (ranging from 5.9 to 11.0 kcal/mol, Figures S44–S48). These results are in good agreement with experimental observations, where the Hydrox-3R variant exhibits ∼ 84% selectivity toward chlorination while also forming hydroxylated products.^29^

#### 3.3.4 How Does Chimera14 Achieve Chlorination Selectivity?

To investigate the effects of residue substitutions in Chimera14, we extracted a representative snapshot (corresponding to the most populated MD conformation in the trajectory) and optimized it using QM/MM to generate Chimera14^1^-RC. In Chimera14^1^-RC, the distance between the ferryl oxygen (O) and the hydrogen (H) of the C4 carbon (C4) of l-Lys was 2.77 Å, with an ∠Fe–O–H angle of 123.8°. We first modeled the HAT reaction using a PES scan and obtained a barrier of 18.4 kcal/mol (Figure S49). At Chimera14^1^-TS1, the O–H and C4–H distances were 1.32 Å and 1.25 Å, respectively, prior to forming Chimera14^1^-IM1. In Chimera14^1^-IM1, the C4–O and C4–Cl distances were 3.08 and 4.16 Å. Spin density analysis indicated that HAT proceeds via a σ-pathway (Figure S50). Consistent with these distances, the subsequent hydroxylation from Chimera14^1^-IM1 was favored, with a barrier of 8.7 kcal/mol, whereas chlorination required a higher barrier of 24.5 kcal/mol (Figure S49). Notably, the chlorination product from this step corresponded to the S-isomer of 4-Cl-l-Lys, while experiments report the R-isomer.^29^ Thus, additional active site rearrangements are required to achieve the observed stereo- and chemo-selectivity. Similar to BesD, we performed isomerization from Chimera14^1^-IM1, which involves flipping the −OH group from *trans* to H207 to *trans* to H140, requiring a barrier of 5.7 kcal/mol to form Chimera14^1^-Iso1-IM compared to 8.7 kcal/mol required for hydroxylation (Figure 9). At Chimera14^1^-Iso1-IM, the ∠N–Fe–O angle decreased from 155.0° in Chimera14^1^-IM to 135.3° (Figure S51). From Chimera14^1^-Iso1-IM, chlorination still required a high barrier of 12.5 kcal/mol to form the *S*-isomer of 4-Cl-l-Lys. However, a second isomerization, involving the flip of the -Cl group from *trans* to succinate to *trans* to H207, proceeded with a low barrier of 3.5 kcal/mol, yielding Chimera14^1^-Iso2-IM. At Chimera14^1^-Iso2-IM, the ∠N–Fe–Cl angle increased to 167.9° (Figures 9 and S51). The subsequent chlorination then required a very low barrier of 0.8 kcal/mol, producing Chimera14^1^-Iso2-PC-Cl. Importantly, the product matched the experimentally observed stereoselectivity, yielding the *R*-isomer of 4-Cl-l-Lys.^29^ Thus, similar to BesD, the Chimera14 variant follows the same reaction mechanism, involving active site isomerization in which the −OH and −Cl groups switch positions. The Fe center conformation in which the −Cl group is *trans* to H207 is proposed to be the catalytically active state for chemo- and stereoselective chlorination. Consistent with BesD, this isomerization also alters the spin density on the −Cl group, increasing it from 0.17 in Chimera14^1^-IM1 to 0.22 at Chimera14^1^-Iso2-IM (Figure S52). To ensure robustness of the calculated mechanism, we repeated the reaction path calculations using another snapshot (Chimera14^2^-RC), extracted from the MD trajectory. The HAT reaction proceeded with a barrier of 24 kcal/mol, leading to the formation of Chimera14^2^-IM1. From this intermediate, chlorination occurred with a barrier of 12.7 kcal/mol, whereas hydroxylation required 13.8 kcal/mol (Figure S53), indicating a slight preference for chlorination. However, the first isomerization, involving the flip of the −OH group, required a lower barrier of 7.1 kcal/mol to form Chimera14^2^-Iso1-IM. This intermediate then underwent a second isomerization, involving the flip of the −Cl group, with a very low barrier of 0.3 kcal/mol, yielding Chimera14^2^-Iso2-IM (Figure S54). As observed in Chimera14^1^-RC, chlorination from Chimera14^2^-Iso2-IM proceeded with a low barrier of 3.9 kcal/mol, whereas hydroxylation required a much higher barrier of 25.9 kcal/mol. Additional QM/MM calculations using four more snapshots were performed to evaluate the influence of the conformational dynamics of the ferryl complex of Chimera14 on the activation energy. The calculations show that the Boltzmann-averaged activation energy for the HAT reaction is 16.7 kcal/mol, with individual barriers ranging from 15.6 to 24.0 kcal/mol (Figure S55).

##### 3.3.4.1 Role of SCS and LR Residues in the Catalytic Mechanism of Chimera14

To elucidate the role of SCS and LR residues in the reaction mechanism of Chimera14, we performed EDA. The EDA results indicate that, relative to Chimera14^1^-RC, residues K173, D196, and R240 contribute to stabilizing the HAT TS (Chimera14^1^-TS1), whereas residues D122, E123, and H137 contribute to destabilization (Figure S56). Notably, H137 acts as a stabilizing substrate-binding residue in BesD; however, in Chimera14, the LR residue D196 uniquely participates in stabilizing the HAT TS. For the chlorination and hydroxylation reactions proceeding from Chimera14^1^-IM1, residues D122 (2.82), E123 (4.40), and W141 (3.15) exert stronger destabilizing effects in the chlorination pathway; in contrast, during hydroxylation, the same residues exhibit reduced destabilizing contributions- D122 (2.52), E123 (2.90), and D239 (2.43). Although D122 and E123 contribute to both pathways, the differences in their magnitudes lead to a higher activation barrier for chlorination relative to hydroxylation from Chimera14^1^-IM (Figure S56). Residue R240, previously predicted to destabilize the TS in Hydrox, is predicted here to stabilize the TSs across all steps of the Chimera14 mechanism-HAT, chlorination, and hydroxylation, indicating its crucial catalytic role. Finally, we also performed EDA for the chlorination TSs arising from Chimera14^1^-Iso1-IM and Chimera14^1^-Iso2-IM. In contrast to Chimera14^1^-TS-Cl, the destabilizing contributions of residues D122, E123, and R218 are reduced in both Chimera14^1^-Iso1-TS-Cl and Chimera14^1^-Iso2-TS-Cl (Figure S57). Similar to observations in BesD, isomerization in the Chimera14 system alters the orientation of succinate, which in turn introduces subtle changes in the residues contributing to TS stabilization or destabilization. These shifts are consistent with the correlated motion predicted by DCCA analysis of the MD trajectory of the Chimera14 system. Therefore, the catalytic center isomerization induces changes in the active site environment that collectively enhance the chlorination pathway.

##### 3.3.4.2 Role of Electric Field in the Catalytic Mechanism of Chimera14

Similar to our analyses of BesD, we computed the IEF along the Fe–O and Fe–Cl bonds for the different intermediate states of Chimera14^1^-RC. In Chimera14^1^-RC, the IEF along the Fe–O bond is −0.0317 a.u., consistent with the value observed in BesD. This IEF becomes less negative at Chimera14^1^-IM1 (−0.0302 a.u.), decreases again at Chimera14^1^-Iso1-IM (−0.0343 a.u.), and then shifts to a slightly positive value at Chimera14^1^-Iso2-IM (0.0018 a.u.). Along the Fe–Cl bond, the IEF is 0.0063 a.u. in Chimera14^1^-IM1, increases to 0.0098 a.u. after the first isomerization, and upon second isomerization, reduces to −0.0243 a.u. (Figure 10). This pronounced shift in IEF correlates with the increased spin density on the −Cl ligand at Chimera14^1^-Iso2-IM, suggesting that the more negative IEF along Fe–Cl facilitates efficient chlorination. Accordingly, the more negative Fe–Cl IEF contributes to the favorable/low chlorination barrier observed after the second isomerization in Chimera14^1^-RC. In Chimera14^2^-RC, the calculated IEFs along the Fe–O and Fe–Cl are −0.0335 and 0.0091 a.u., respectively. The subtle difference in the Fe–O IEF may account for the variation in HAT barriers between Chimera14^1^-RC and Chimera14^2^-RC. Upon formation of Chimera14^2^-IM1, the Fe–O IEF becomes less negative (−0.0283 a.u.), while the Fe–Cl IEF increases to 0.010 a.u. Similar to Chimera14^1^-RC, the Fe–O IEF increases further to −0.0219 a.u. after the first isomerization, while the Fe–Cl IEF remains unchanged. However, after the second isomerization, the Fe–Cl IEF decreases to −0.0164 a.u. (Figure S58). Notably, the magnitude of the negative Fe–Cl IEF in Chimera14^1^-Iso2-IM is smaller than in Chimera14^2^-Iso2-IM. Thus, the chlorination barriers differ significantly between Chimera14^1^-Iso2-IM (0.8 kcal/mol) and Chimera14^2^-Iso2-IM (3.9 kcal/mol), demonstrating that chlorination efficiency is strongly dependent on the IEF along the Fe–Cl bond.

**Figure 10:**
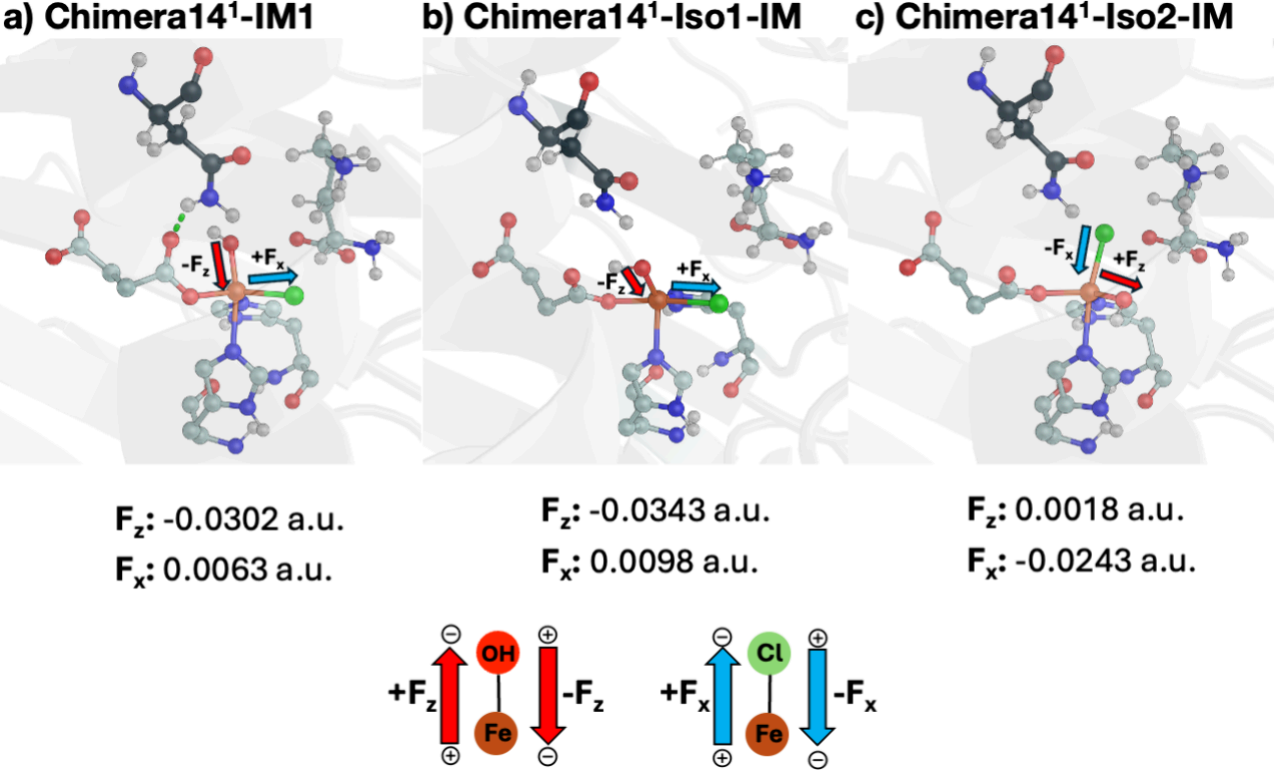
IEF variation along the Fe–Cl and Fe–O bonds in Cl–Fe­(III)–OH isomers of Chimera14^1^-RC. The IEF vectors are defined from positive to negative according to the TITAN convention. The red arrows indicate the field components, with the *z*-axis aligned along the Fe–O bond and the *x*-axis along the Fe–Cl bond (shown in blue).

##### 3.3.4.3 Effects of Isomerization in the Electronic Structure of Chimera14

FMO analysis of Chimera14^1^-IM1, Chimera14^1^-Iso1-IM, and Chimera14^1^-Iso2-IM indicates that the electronic configuration of the Fe center changes during isomerization. As in BesD, the electronic configuration of Chimera14^1^-IM1 is 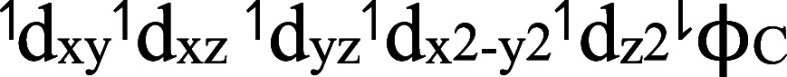 consistent with a σ-transfer HAT involving electron transfer from σ_C–H_ to d_*z*_2 orbital (Figure S59). The FMO energies show that in Chimera14^1^-IM1, the LUMO is d_*xy*_, while LUMO+1 and LUMO+2 correspond to d_*xz*_ and d_*yz*,_ respectively (Figure S59). SNO analysis of TSs for chlorination and hydroxylation further reveals that the hydroxylation from Chimera14^1^-IM1 proceeds via electron transfer from ϕ_C_ to d_*xy*_ (LUMO), whereas chlorination requires electron transfer from ϕ_C_ to the d_*yz*_ (LUMO+2) (Figure S60). Correspondingly, the hydroxylation barrier is lower than the chlorination barrier from Chimera14^1^-IM1. After the first isomerization at Chimera14^1^-Iso1-IM, the d_*xz*_ orbital becomes the LUMO, while the d_*yz*_ and d_*xy*_ orbitals shift to LUMO+1 and LUMO+2, respectively (Figure S59). Consequently, chlorination from Chimera14^1^-Iso1-IM involves electron transfer from ϕ_C_ to the d_*yz*_ orbital, which corresponds to LUMO+1 as shown by the SNO analysis (Figure S59), corresponding to a barrier of 17.7 kcal/mol. After the second isomerization, however, the d_*xz*_ orbital remains the LUMO in Chimera14^1^-Iso2-IM (Figure S59). Contrary to chlorination from Chimera14^1^-Iso1-IM, the SNO analysis of Chimera14^1^-Iso2-TS-Cl revealed that the electron transfer happens from ϕ_C_ to d_*xz*_ (Figure S60), which is the LUMO orbital at Chimera14^1^-Iso2-IM, thus leading to a lower barrier for chlorination originating from Chimera14^1^-Iso2-IM. Thus, the electronic structure changes during isomerization help enhance the chlorination selectivity of the Chimera14 variant, as observed in the native halogenase BesD.

## 4 Conclusions

In this study, we employed an integrated computational strategy that combines MD and hybrid QM/MM calculations to elucidate the catalytic mechanism by which the Fe­(II)/2OG halogenase BesD achieves the selective chlorination of its l-Lys substrate. To uncover the mechanistic principles underlying the conversion of a hydroxylase into a halogenase, we further examined a homologous hydroxylase and two engineered variants (Hydrox-3R and Chimera14). Our results reveal the critical effects of dynamics, correlated motions, and key trends on electronic structure and electric-field properties across the enzyme series. Together, these findings clarify how the evolution of geometric, electronic structure, and dynamical features enables halogenase reactivity and provide a mechanistic framework for future enzyme engineering efforts. Our calculations provide key mechanistic insight into BesD catalysis. Immediately following HAT, the resulting Cl–Fe­(III)–OH intermediate is geometrically and kinetically predisposed toward hydroxylation, indicating that chlorination is not inherently favored at this stage. We propose that BesD overcomes this hydroxylation bias through a two-step isomerization process that swaps the positions of the −OH and −Cl ligands, in contrast to the previously suggested one-step isomerization.^51^ This rearrangement places the – Cl ligand *trans* to H207, a conformation that supports a significantly lower barrier for chlorination.

IEF calculations indicate that this *trans*-H207 geometry produces a more negative electric field along the Fe–Cl bond, thereby increasing the electron density on the chloride ligand and facilitating efficient C–Cl bond formation. FMO, NO, and SNO analyses reveal that the as-formed Cl–Fe­(III)–OH intermediate in BesD electronically favors hydroxylation, with the d_*xy*_ orbital serving as the accessible LUMO. Two-step isomerization reorganizes the Fe d-orbital manifold, stabilizing the d_*xz*_ orbital as the new LUMO and shifting d_*xy*_ to a higher-lying state. This reordering lowers the barrier for electron transfer into d_*xz*_ during chlorination while simultaneously raising the barrier for hydroxylation. Together, these results clarify how BesD redirects its initially hydroxylation-biased intermediate toward productive halogenation.

To elucidate how hydroxylase activity can evolve into halogenase activity, we additionally performed MD and QM/MM calculations on the Hydrox, Hydrox-3R, and Chimera14 systems. Our results predict that chlorination selectivity emerges through the progressive crafting of essential correlated motions between second-sphere residues and the active site motions characteristic of the halogenase BesD, which are likely to facilitate the required isomerization process. Notably, in the Chimera14 variant, the introduced substitutions generated a new correlated motion involving the engineered residues, succinate, and the l-Lys substrate. This dynamic coupling was absent in the parent Hydrox enzyme, highlighting its functional importance. IEF calculations further revealed a stepwise strengthening of electrostatic control from Hydrox to Hydrox-3R and Chimera14, consistently favoring the coordination geometry in which the −Cl ligand occupies the position *trans* to H207, mirroring the most productive halogenation conformation in BesD. The FMO analysis demonstrated that isomerization in these variants similarly rearranges the electronic structure of the Cl–Fe­(III)–OH intermediate, lowering the energy of the key acceptor orbital and thereby promoting efficient C–Cl bond formation. Together, these findings provide a mechanistic rationale for how structural, dynamical, and electrostatic features can be crafted and tuned to shift hydroxylase reactivity toward selective halogenation.

Collectively, these results advance the fundamental science of Fe­(II)/2OG-dependent halogenation and establish a mechanistic blueprint for future enzyme redesign aimed at expanding and diversifying non-heme Fe­(II)/2OG oxygenase catalysis, particularly selective C–H halogenation.

## Supplementary Material



## Notes

Additional images, optimized QM/MM geometries, and absolute energies (PDF)
