# Supplementary material for: How Fe(II)/2-Oxoglutarate Oxygenase Chooses Chlorination over Hydroxylation: Electric Field-Driven Ligand Exchange Governs C–Cl Formation
Source: J Am Chem Soc. 2026 May 23;148(21):21344–64. doi: 10.1021/jacs.5c22369 (PMC13244439; doi:10.1021/jacs.5c22369)
Supplement: Supplementary file 1 [file ja5c22369_si_001.pdf]

How Fe(II)/2-Oxoglutarate Oxygenase Chooses Chlorination over Hydroxylation: Electric Field-Driven Ligand Exchange Governs C–Cl Formation

Simahudeen Bathir Jaber Sathik Rifayee,[a#] Midhun George Thomas,[a#] Anandhu Krishnan,[a] Kritika Gupta, [a] Carter Davis,[b] Tatyana Karabencheva-Christova,[a*] Christo Z. Christov [a*].

[a] Department of Chemistry, [b] Department of Chemical Engineering, Michigan Technological University, Houghton, MI 49931, USA.

* Corresponding Authors

Table of Contents

Table of Contents......2

TABLE OF FIGURES......2

LIST OF TABLES......5

1. Conformational Dynamics of Offline Fe(III)-OO•⁻ Complex and Reaction Mechanism of O_2 Activation in BesD.......7

2. MD simulation of the Fe(IV)=O Intermediate in Hydrox, Hydrox-3R and Chimera14......28

3. IEF, Spin Density, and EDA analysis of HAT and Rebound Hydroxylation in Hydrox......33

4. Coordinates......64

5. References......160

TABLE OF FIGURES

Figure S1. General mechanistic strategy of non-heme Fe(II)/2OG-dependent oxygenases/halogenases. 6

Figure S2. Conformational Dynamics of BesD-Fe(III)-OO•⁻•L-Lys system. a) Root mean square deviation (RMSD) of the dynamics suggests that the system is equilibrated, b) Root mean square fluctuation (RMSF) of the system identifies flexible regions. 7

Figure S3. Conformational analysis of BesD-Cl-Fe(III)-OO•⁻•L-Lys system. a) PCA showing the flexible regions of the enzyme with boxed regions highlighting the most flexible regions. b) DCCA plot showing the correlated and anticorrelated motions existing between different regions of the enzyme. Boxed regions show regions that exhibit high anti-correlated motion. 8

Figure S4. a) Reaction Profile of O_2 activation obtained from BesD-SO-RC, b) QM/MM optimized structures of stationary points obtained from BesD-SO-RC. Distances are mentioned in Å, and energies are mentioned in kcal/mol at the QM(B3)/MM level. 10

Figure S5. Conformational Dynamics of BesD-Fe(IV)=O•L-Lys system. a) RMSD of the dynamics suggests that the system is equilibrated, b) RMSF of the system identifies flexible regions. 11

Figure S6. Substrate binding interactions observed in the MD trajectory of BesD-Fe(IV)=O•L-Lys system. 11

Figure S7. Substrate flexibility in BesD-Fe(IV)=O•L-Lys. a) Histogram plot of distance between Ferryl oxygen and C4-carbon of the substrate and hydrogens attached to it, b) Evolution of distances over time, c) ChemDraw image showing the color-coded distances depicted in (a) and (b). 12

Figure S8. Interactions stabilizing the active site in BesD-Fe(IV)=O•L-Lys system. 12

Figure S9. Histogram plot showing the clustered FeO–C distances derived from classical MD (1 μs) and QM/MM MD (4 ps) simulations. The QM/MM MD simulations were performed using five representative snapshots selected for the QM/MM calculations. 13

Figure S10. Time-dependent fluctuations of the FeO–C distance observed during the 4 ps QM/MM MD simulation. 14

Figure S11. EDA analysis of HAT across the five snapshots used for the QM/MM calculations in BesD. 15

Figure S12. Spin density plots for the stationary points obtained during QM/MM calculations on the BesD4-RC snapshot before isomerization. 16

Figure S13. Spin density plots for the stationary points obtained during QM/MM calculations on the BesD4-RC snapshot during and after isomerization. 17

Figure S14. a) Reaction Profile of isomerization, hydroxylation, and chlorination reactions obtained from BesD3-RC, b) QM/MM optimized structures of stationary points obtained from BesD3-RC. Distances are mentioned in Å, and energies are mentioned in kcal/mol at the QM(B3)/MM level. 18
Figure S15. Spin density plots for the stationary points obtained during QM/MM calculations on the BesD3-RC snapshot during and after isomerization. 19
Figure S16. Reaction Profile of isomerization, hydroxylation, and chlorination reactions obtained from BesD5-RC. 20
Figure S17. IEF variation along the Fe–Cl and Fe–O bonds in Cl–Fe(III)–OH isomers of BesD3-RC. The IEF vectors are defined from positive to negative according to the TITAN convention. The red arrows indicate the field components, with the z-axis aligned along the Fe–O bond and the x-axis along the Fe–Cl bond (shown in blue). 21
Figure S18. EDA plots of TS states obtained during HAT, hydroxylation, and chlorination reactions from BesD4-RC snapshot. 22
Figure S19. EDA analysis of HAT, hydroxylation, chlorination reactions of BesD3-RC snapshot. 23
Figure S20. Spin Natural Orbital (SNO) and Natural Orbital (NO) analysis of the TS states of HAT, chlorination, and hydroxylation originating from different isomers of Cl-Fe(III)-OH intermediate obtained from BesD4-RC, a) BesD4-TS1, b) BesD4-TS-Cl, c) BesD4-TS-OH, d) BesD4-Iso1-TS-Cl, e) BesD4-Iso2-TS-Cl and f) BesD4-Iso2-TS-OH. 24
Figure S21. FMO analysis of the isomers of Cl-Fe(III)-OH intermediates obtained from BesD3-RC, a) BesD3-IM1, b) BesD3-Iso1-IM, and c) BesD3-Iso2-IM. 25
Figure S22. SNO and NO analysis of the TS states of HAT, chlorination, and hydroxylation originating from different isomers of Cl-Fe(III)-OH intermediate obtained from BesD3-RC, a) BesD3-TS1, b) BesD3-TS-Cl, c) BesD3-TS-OH, d) BesD3-Iso1-TS-Cl, e) BesD3-Iso2-TS-Cl, and f) BesD3-Iso2-TS-OH 26
Figure S23. Conformational Dynamics of Hydrox-Fe(IV)=O•L-Lys system. a) RMSD of the dynamics suggests that the system is equilibrated, b) RMSF of the system identifies flexible regions. 26
Figure S24. Conformational Dynamics of Hydrox-3R-Fe(IV)=O•L-Lys system. a) RMSD of the dynamics suggests that the system is equilibrated, b) RMSF of the system identifies flexible regions. .. 27
Figure S25. Conformational Dynamics of Chimera14-Fe(IV)=O•L-Lys system. a) RMSD of the dynamics suggests that the system is equilibrated, b) RMSF of the system identifies flexible regions. .. 27
Figure S26. Substrate flexibility in Hydrox-Fe(IV)=O•L-Lys. a) Histogram plot of distance between Ferryl oxygen and C4-carbon of the substrate and hydrogens attached to it, b) Evolution of distances over time, c) ChemDraw image showing the color-coded distances depicted in (a) and (b). 29
Figure S27. Substrate flexibility in Hydrox-3R-Fe(IV)=O•L-Lys. a) Histogram plot of distance between Ferryl oxygen and C4-carbon of the substrate and hydrogens attached to it, b) Evolution of distances over time, c) ChemDraw image showing the color-coded distances depicted in (a) and (b). 29
Figure S28. Substrate flexibility in Chimera14-Fe(IV)=O•L-Lys. a) Histogram plot of distance between Ferryl oxygen and C4-carbon of the substrate and hydrogens attached to it, b) Evolution of distances over time, c) ChemDraw image showing the color-coded distances depicted in (a) and (b). 30
Figure S29. Conformational behavior of Fe(IV)=O species of Hydrox, Hydrox-3R, and Chimera14 variants. a), c), and e) PCA showing the dominant motions of the enzyme. Boxed regions showed dominant motions. b), d), and f) DCCA plot showing the correlated/anticorrelated motions involved in the system. Boxed regions show correlated and anticorrelated motions of the flexible regions in (a), (c), and (e). 31
Figure S30. QM/MM optimized structures of stationary points obtained from Hydrox-RC. Distances are mentioned in Å. 32
Figure S31. QM/MM reaction profiles of HAT reactions in Hydrox. The relative energies are given in kcal/mol at the QM(B3)/MM level. 33
Figure S32. Spin density plots for the stationary points obtained during QM/MM calculations on the Hydrox-RC. 34

Figure S33. EDA analysis of chlorination and hydroxylation post-isomerization steps in Hydrox-RC snapshot. 35
Figure S34. a) Reaction Profile of hydroxylation and chlorination reactions obtained from Hydrox-3R[1]-RC, b) QM/MM optimized structures of stationary points obtained from Hydrox-3R[1]-RC. Distances are mentioned in Å, and energies are mentioned in kcal/mol at the QM(B3)/MM level. 36
Figure S35. Spin density plots for the stationary points obtained for hydroxylation and chlorination reactions during QM/MM calculations on the Hydrox-3R[1]-RC 37
Figure S36. QM/MM optimized structures of stationary points obtained from Hydrox-3R[1]-IM. Distances are mentioned in Å 38
Figure S37. IEF variation along the Fe–Cl and Fe–O bonds in Cl–Fe(III)–OH isomers of Hydrox-3R[1]-RC. The IEF vectors are defined from positive to negative according to the TITAN convention. The red arrows indicate the field components, with the z-axis aligned along the Fe–O bond and the x-axis along the Fe–Cl bond (shown in blue) 39
Figure S38. Spin density plots for the stationary points obtained for isomerization, hydroxylation, and chlorination reactions during QM/MM calculations on the Hydrox-3R[1]-RC. 40
Figure S39. EDA analysis of HAT, hydroxylation, and chlorination reactions of Hydrox-3R[1]-RC snapshot. 41
Figure S40. EDA analysis of chlorination and hydroxylation post-isomerization steps in Hydrox-3R[1]-RC snapshot. 42
Figure S41. FMO analysis of the isomers of Cl-Fe(III)-OH intermediates obtained from Hydrox-3R[1]-RC, a) Hydrox-3R[1]-IM1, b) Hydrox-3R[1]-Iso1-IM, and c) Hydrox-3R[1]-Iso2-IM. 43
Figure S42. SNO and NO analysis of the TS states of HAT, chlorination, and hydroxylation originating from different isomers of Cl-Fe(III)-OH intermediate obtained from Hydrox-3R[1]-RC, a) Hydrox-3R[1]-TS1, b) Hydrox-3R[1]-TS-Cl, c) Hydrox-3R[1]-TS-OH, d) Hydrox-3R[1]-Iso1-TS-Cl, e) Hydrox-3R[1]-Iso2-TS-Cl, and f) Hydrox-3R[1]-Iso2-TS-OH. 44
Figure S43. QM/MM reaction profiles of HAT reactions in Hydrox-3R. The relative energies are given in kcal/mol at the QM(B3)/MM level. 45
Figure S44. QM/MM reaction profile for HAT and the subsequent isomerization and rebound hydroxylation steps, starting from snapshot Hydrox-3R[1] 46
Figure S45. QM/MM reaction profile for HAT and the subsequent isomerization and rebound hydroxylation steps, starting from snapshot Hydrox-3R[2] 47
Figure S46. QM/MM reaction profile for HAT and the subsequent isomerization and rebound hydroxylation steps, starting from snapshot Hydrox-3R[3] 48
Figure S47. QM/MM reaction profile for HAT and the subsequent isomerization and rebound hydroxylation steps, starting from snapshot Hydrox-3R[4] 49
Figure S48. QM/MM reaction profile for HAT and the subsequent isomerization and rebound hydroxylation steps, starting from snapshot Hydrox-3R[5] 50
Figure S49. a) Reaction Profile of hydroxylation and chlorination reactions obtained from Chimera14[1]-RC, b) QM/MM optimized structures of stationary points obtained from Chimera14[1]-RC. Distances are mentioned in Å, and energies are mentioned in kcal/mol at the QM(B3)/MM level. 51
Figure S50. Spin density plots for the stationary points obtained for hydroxylation and chlorination reactions during QM/MM calculations on the Chimera14[1]-RC. 52
Figure S51. QM/MM optimized structures of stationary points obtained from Chimera14[1]-IM1. Distances are mentioned in Å 53
Figure S52. Spin density plots for the stationary points obtained for isomerization, hydroxylation, and chlorination reactions during QM/MM calculations on the Chimera14[1]-RC 54
Figure S53. a) Reaction Profile of hydroxylation and chlorination reactions obtained from Chimera14[2]-RC, b) QM/MM optimized structures of stationary points obtained from Chimera14[2]-RC. Distances are mentioned in Å, and energies are mentioned in kcal/mol at the QM(B3)/MM level. 55

Figure S54. a) Reaction Profile of isomerization, hydroxylation, and chlorination reactions obtained from Chimera14^2-RC, b) QM/MM optimized structures of stationary points obtained from Chimera14^2-RC. Distances are mentioned in Å, and energies are mentioned in kcal/mol at the QM(B3)/MM level. 56
Figure S55. QM/MM reaction profiles of HAT reactions in Chimera14. The relative energies are given in kcal/mol at the QM(B3)/MM level. 57
Figure S56. EDA analysis of HAT, chlorination and hydroxylation steps in Chimera14^1-RC snapshot. 58
Figure S57. EDA analysis of chlorination and hydroxylation post-isomerization steps in Chimera14^1-RC snapshot. 59
Figure S58. IEF variation along the Fe–Cl and Fe–O bonds in Cl–Fe(III)–OH isomers of Chimera14^2-RC. The IEF vectors are defined from positive to negative according to the TITAN convention. The red arrows indicate the field components, with the z-axis aligned along the Fe–O bond and the x-axis along the Fe–Cl bond (shown in blue). 60
Figure S59. FMO analysis of the isomers of Cl-Fe(III)-OH intermediates obtained from Chimera14^1-RC, a) Chimera14^1-IM1, b) Chimera14^1-Iso1-IM, and c) Chimera14^1-Iso2-IM. 61
Figure S60. SNO and NO analysis of the TS states of HAT, chlorination, and hydroxylation originating from different isomers of Cl-Fe(III)-OH intermediate obtained from Chimera14^1-RC, a) Chimera14^1-TS1, b) Chimera14^1-TS-Cl, c) Chimera14^1-TS-OH, d) Chimera14^1-Iso1-TS-Cl, e) Chimera14^1-Iso2-TS-Cl, and f) Chimera14^1-Iso2-TS-OH. 62

LIST OF TABLES

Table S1. Tabulated B1, B2, and B3 energy values for the HAT reaction computed with an expanded QM region and MM environment, D3 dispersion corrections, and the def2-TZVP basis set, for the lowest-energy snapshot of BesD (BesD4). 63

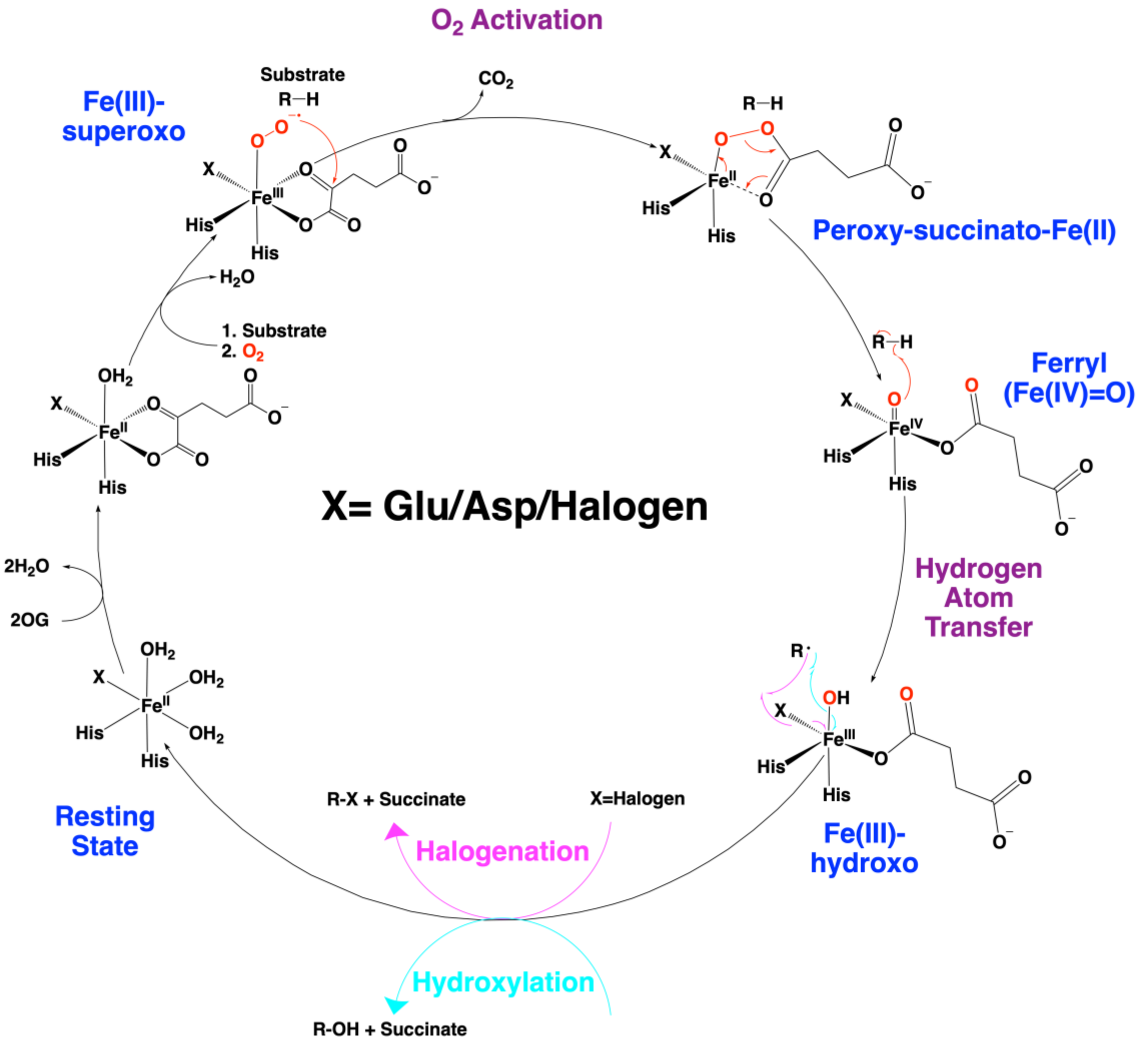

Figure S1. General mechanistic strategy of non-heme Fe(II)/2OG-dependent oxygenases/halogenases.

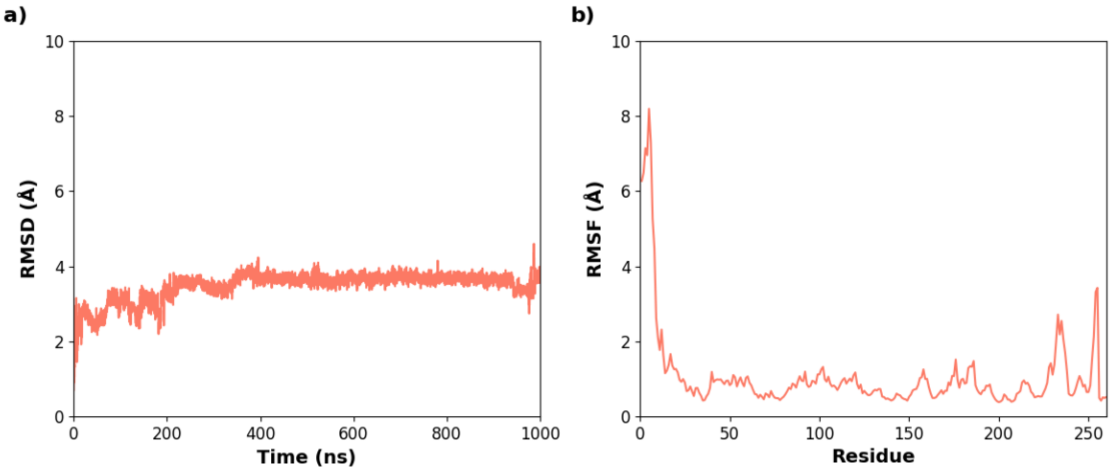

Figure S2. Conformational Dynamics of BesD-Fe(III)-OO$^{\bullet -}$•L-Lys system. a) Root mean square deviation (RMSD) of the dynamics suggests that the system is equilibrated, b) Root mean square fluctuation (RMSF) of the system identifies flexible regions.

1. Conformational Dynamics of Offline Fe(III)-OO$^{\bullet -}$ Complex and Reaction Mechanism of O_2 Activation in BesD.

We modelled the offline Cl-Fe(III)-OO$^{\cdot -}$ complex of BesD, as the crystal structure has the 2-oxoglutarate (2OG) in the offline orientation with the C1 carboxylate of 2OG trans to H207. Computational studies have proposed the O_2 activation mechanism of non-heme Fe(II)/2OG-dependent hydroxylases and halogenases. Initially, we performed MD simulations of offline Cl-Fe(III)-OO$^{\cdot -}$ systems to obtain a well-equilibrated trajectory (BesD-Cl-Fe(III)-OO$^{\bullet -}$•L-Lys) (**Figure S2**). The atomistic analysis revealed the formation of stable hydrogen bonds by R218, Y198, and H137 with the C5 carboxylate of 2OG, while the C1 carboxylate of 2OG forms a hydrogen bond with N222. Principal component analysis (PCA) revealed that the residues 226 to 255 showed dominant motions in the 1 μs trajectory (**Figure S3a**). Furthermore, we employed Dynamic Cross Correlation analysis (DCCA) to investigate long-range correlated motions in the offline Cl-Fe(III)-OO$^{\cdot -}$ complex. DCCA revealed that the residues 226-255 showed correlated motion with the Fe and Fe-binding residues (**Figure S3b**).

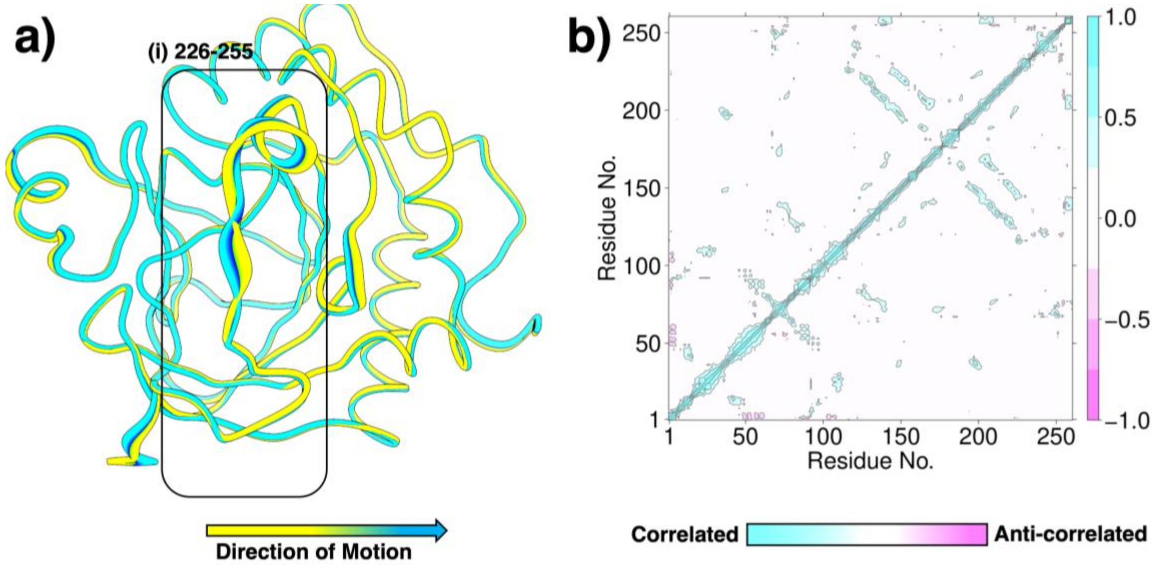

Figure S3. Conformational analysis of BesD-Cl-Fe(III)-$OO^{\bullet -}$•L-Lys system. a) PCA showing the flexible regions of the enzyme with boxed regions highlighting the most flexible regions. b) DCCA plot showing the correlated and anticorrelated motions existing between different regions of the enzyme. Boxed regions show regions that exhibit high anti-correlated motion.

To explore the O_2 activation reaction, we obtained a snapshot from the offline BesD-Cl-Fe(III)-$OO^{\cdot -}$ system and performed QM/MM optimization to obtain the reactant complex (BesD-SO-RC) for the offline Cl-Fe(III)-$OO^{\cdot -}$ system. The QM region definition used for these calculations is described in the "QM/MM Calculations" section of the Methods section. From BesD-SO-RC, the initial O_2 activation reaction involves the Od-C2 bond formation between O_2 and 2OG and C1-C2 bond breakage within 2OG, resulting in the decarboxylation to form the Cl-Fe(II)-peroxo-succinate complex (BesD-SO-IM1). The initial decarboxylation reaction required a reaction barrier of 10.9 kcal/mol (**Figure S4**). In the case of BesD-SO-RC, along the reaction path, the angle formed by nitrogen (N) of H207 with Fe-Op (∠N-Fe-Op) increased from 85.6° in the BesD-SO-RC to 157.6° in BesD-SO-IM1 (**Figure S4**). Subsequently, the Op-Od bond cleavage became barrierless in the inline system at the QM(B3)/MM level, leading to the formation of an Fe^{III}-O intermediate at BesD-SO-IM2, along with succinate. The Fe-Op and Op-Od distances were 1.76 Å and 2.14 Å at BesD-SO-IM2, indicating a partial bond between Op and Od. In addition, at BesD-SO-IM2, the ∠N-Fe-Op increased to 173.7°, reaching the inline orientation with Op

almost trans to H207 (**Figure S4**). However, in Off-SO-IM2, the ∠N-Fe-Op reduced to 95.9°, retaining the offline orientation (**Figure S4**). Further complete breakage of the Op-Od bond and rearrangements of ∠N-Fe-Op led to an inline chloro-Fe(IV)=O species with the oxo group trans to His207, characterized by Fe-Op distances of 1.61 Å in BesD-SO-PD (**Figure S4**). Hence, the calculations indicate that the O_2 activation reaction leads to the formation of inline chloro-Fe(IV)=O as proposed in a previous study.

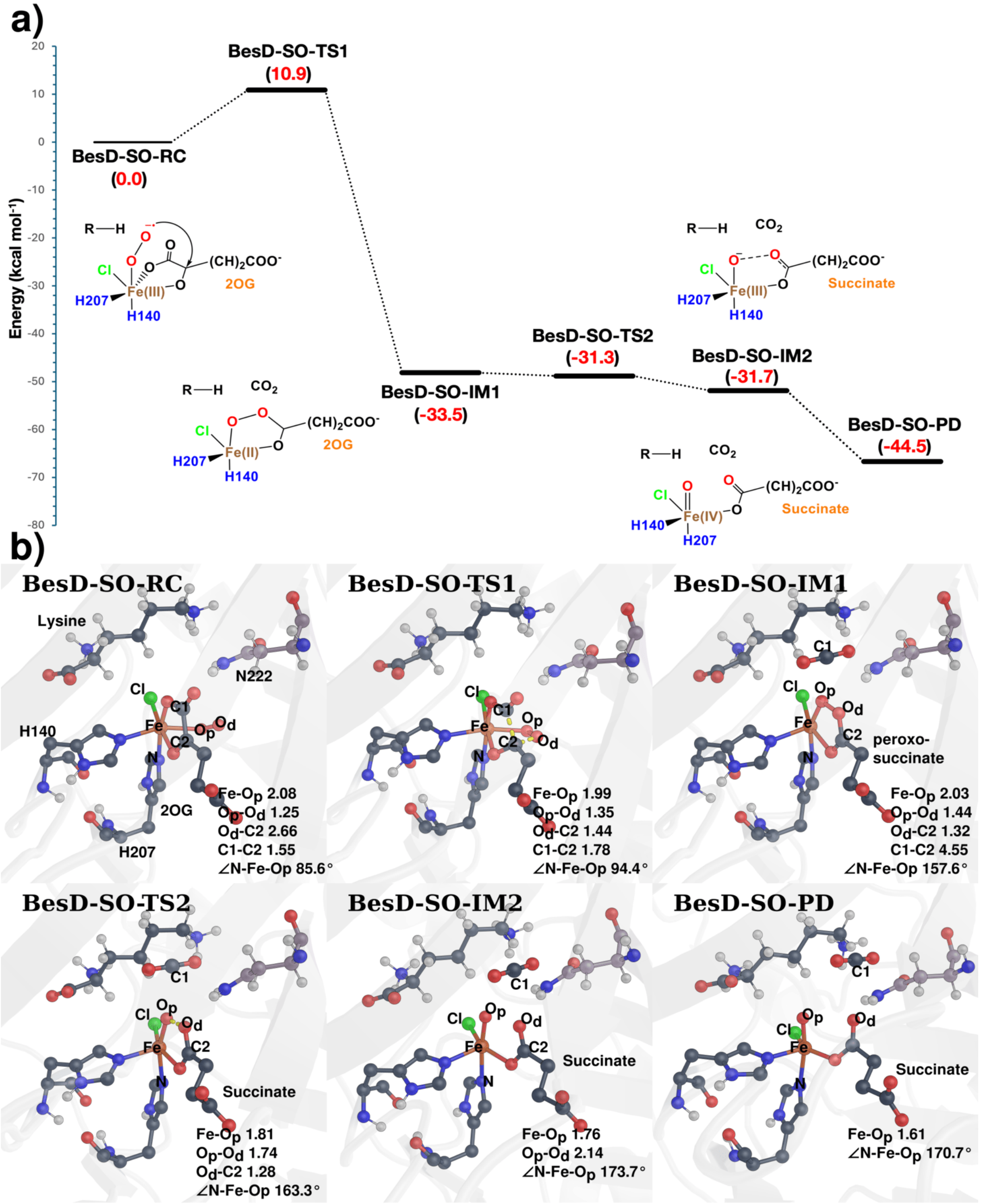

Figure S4. a) Reaction Profile of O_2 activation obtained from BesD-SO-RC, b) QM/MM optimized structures of stationary points obtained from BesD-SO-RC. Distances are mentioned in Å, and energies are mentioned in kcal/mol at the QM(B3)/MM level.

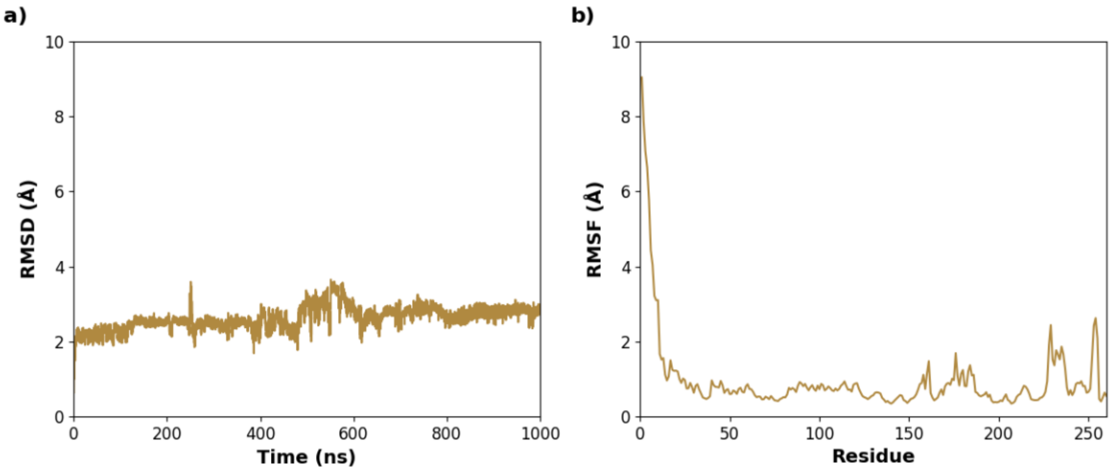

Figure S5. Conformational Dynamics of BesD-Fe(IV)=O•L-Lys system. a) RMSD of the dynamics suggests that the system is equilibrated, b) RMSF of the system identifies flexible regions.

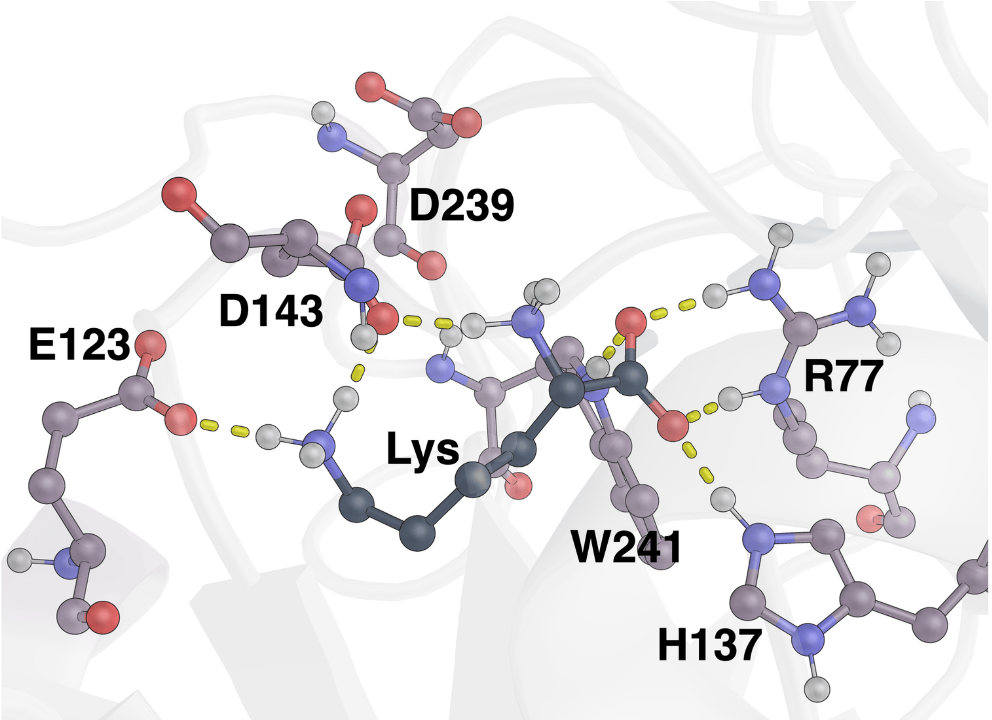

Figure S6. Substrate binding interactions observed in the MD trajectory of BesD-Fe(IV)=O•L-Lys system.

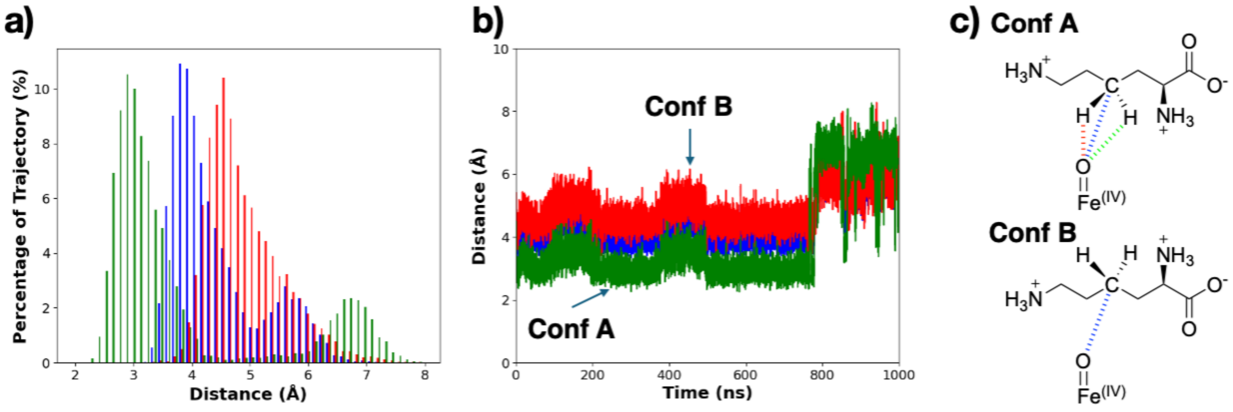

Figure S7. Substrate flexibility in BesD-Fe(IV)=O•L-Lys. a) Histogram plot of distance between Ferryl oxygen and C4-carbon of the substrate and hydrogens attached to it, b) Evolution of distances over time, c) ChemDraw image showing the color-coded distances depicted in (a) and (b).

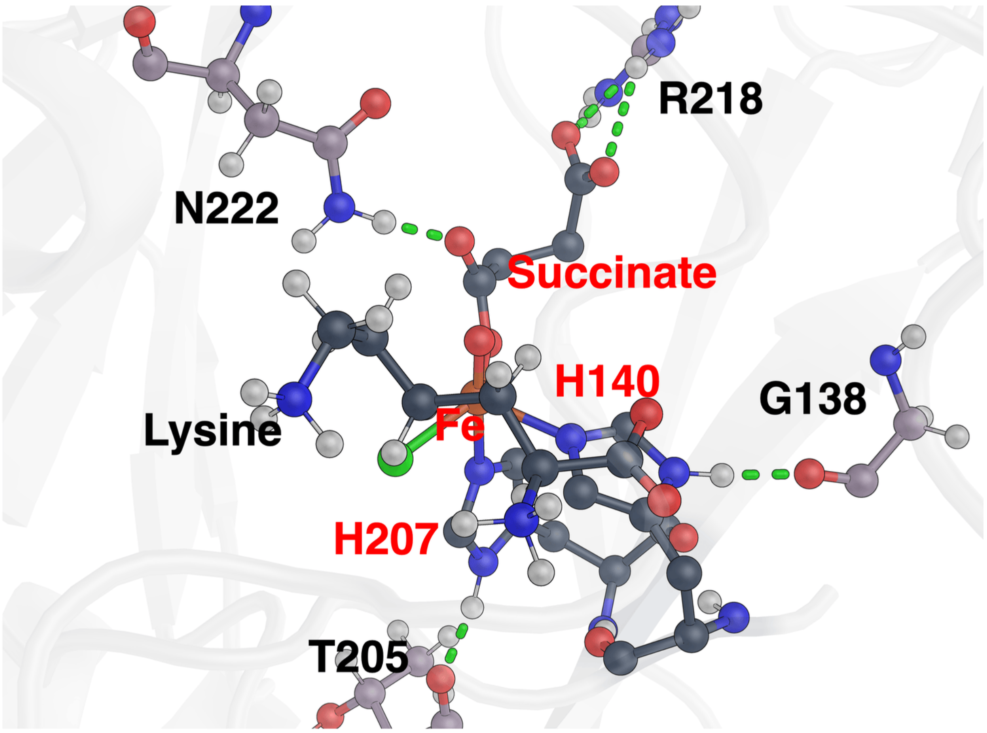

Figure S8. Interactions stabilizing the active site in BesD-Fe(IV)=O•L-Lys system.

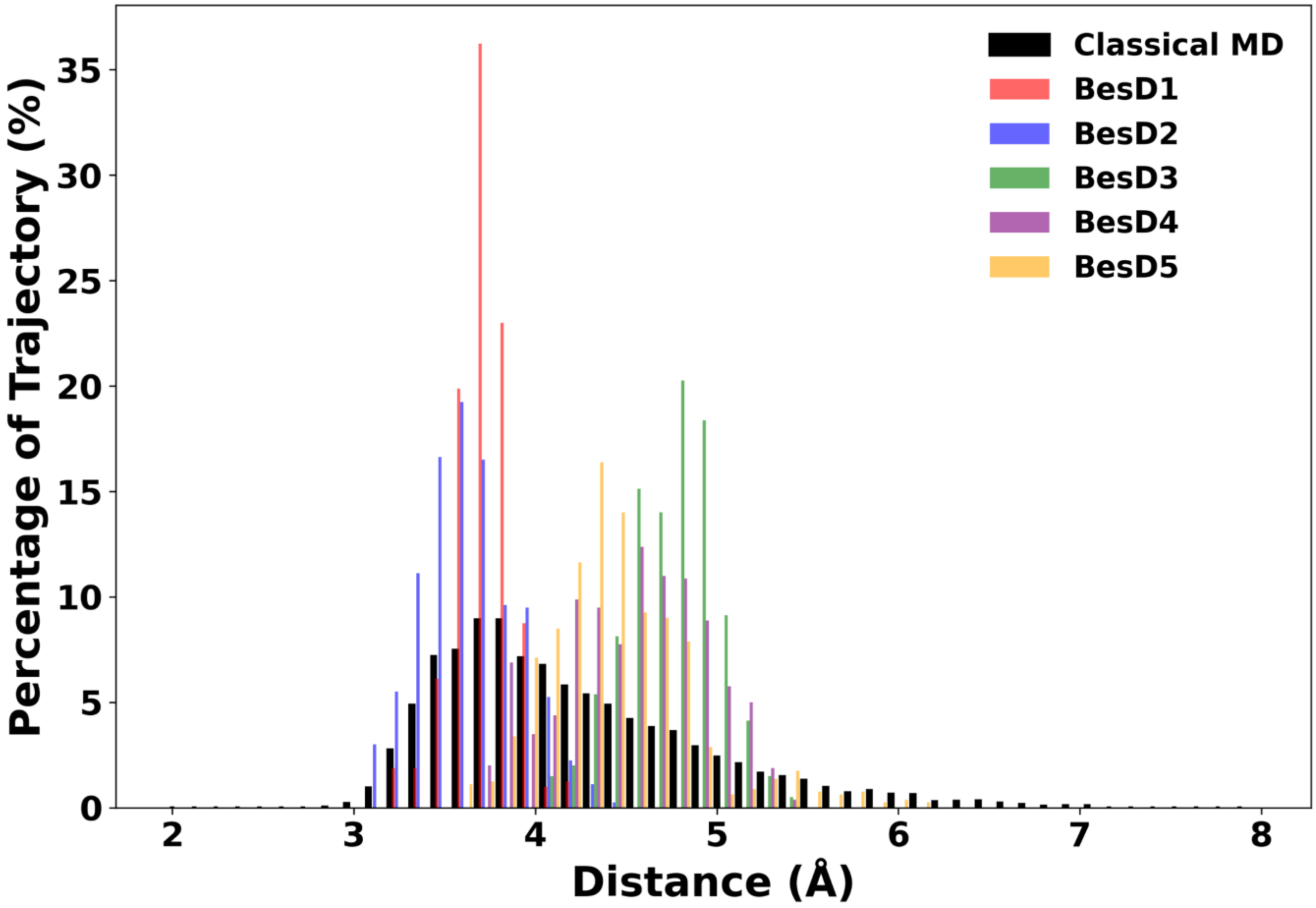

Figure S9. Histogram plot showing the clustered FeO–C distances derived from classical MD (1 μs) and QM/MM MD (4 ps) simulations. The QM/MM MD simulations were performed using five representative snapshots selected for the QM/MM calculations.

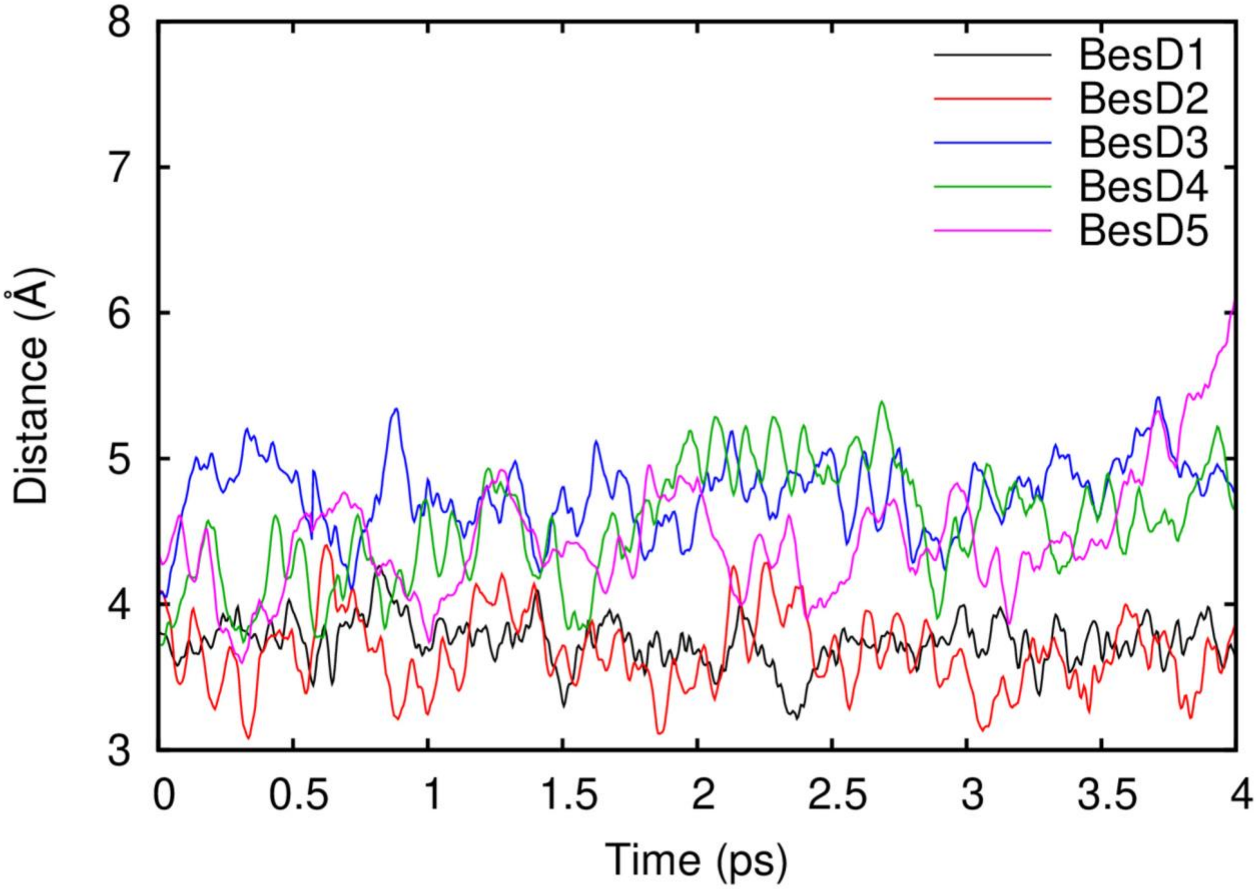

Figure S10. Time-dependent fluctuations of the FeO–C distance observed during the 4 ps QM/MM MD simulation.

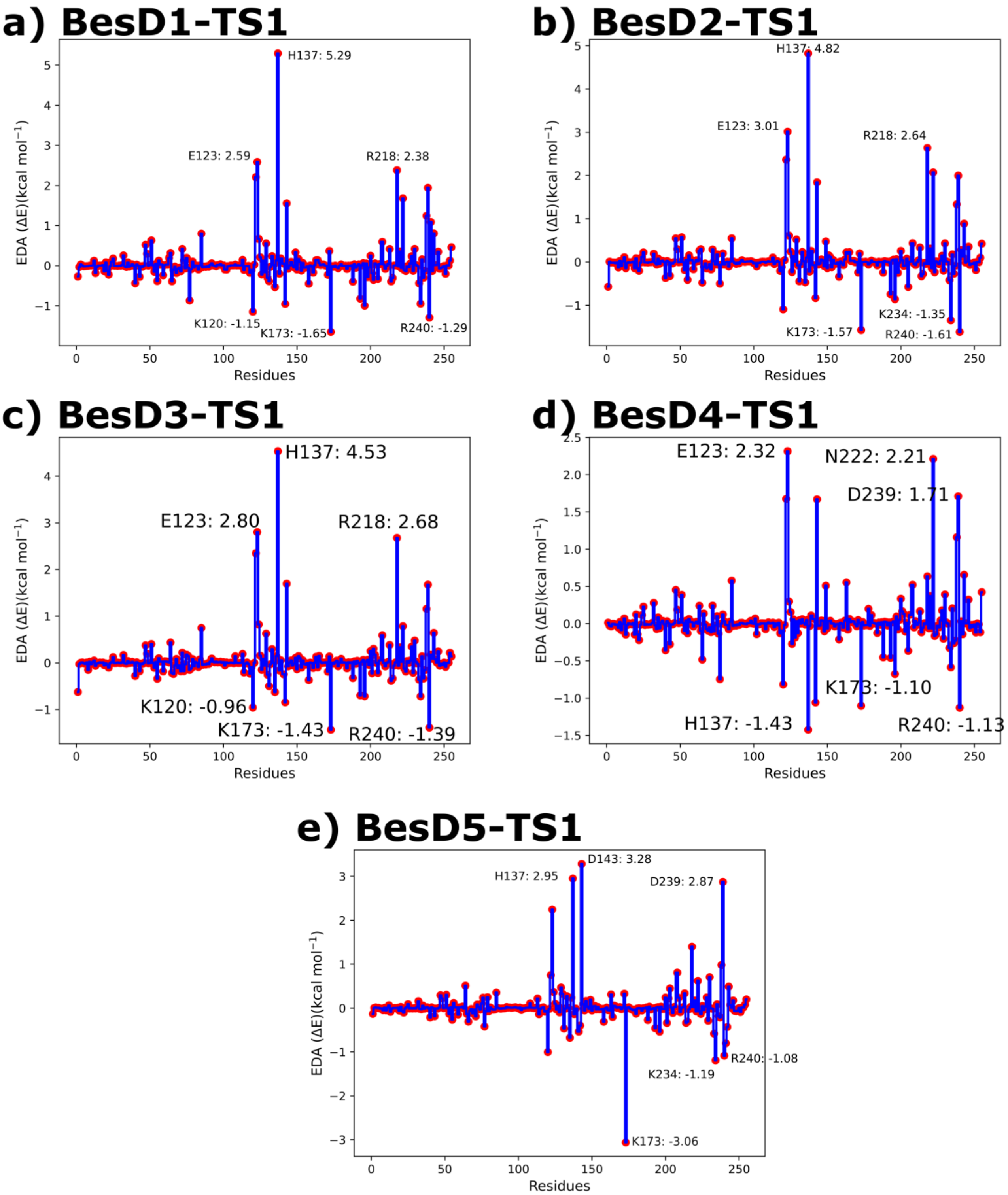

Figure S11. EDA analysis of HAT across the five snapshots used for the QM/MM calculations in BesD.

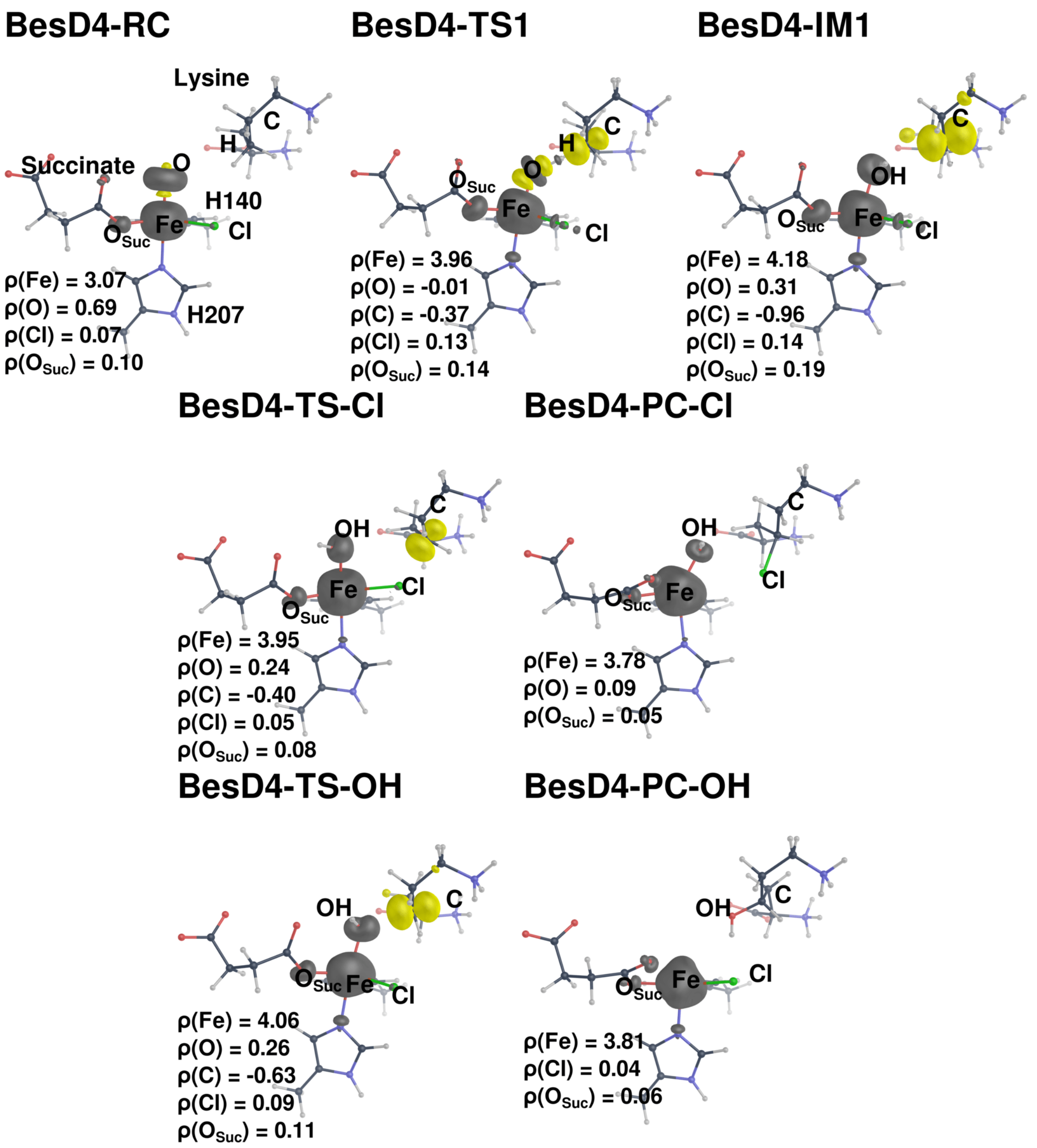

Figure S12. Spin density plots for the stationary points obtained during QM/MM calculations on the BesD4-RC snapshot before isomerization.

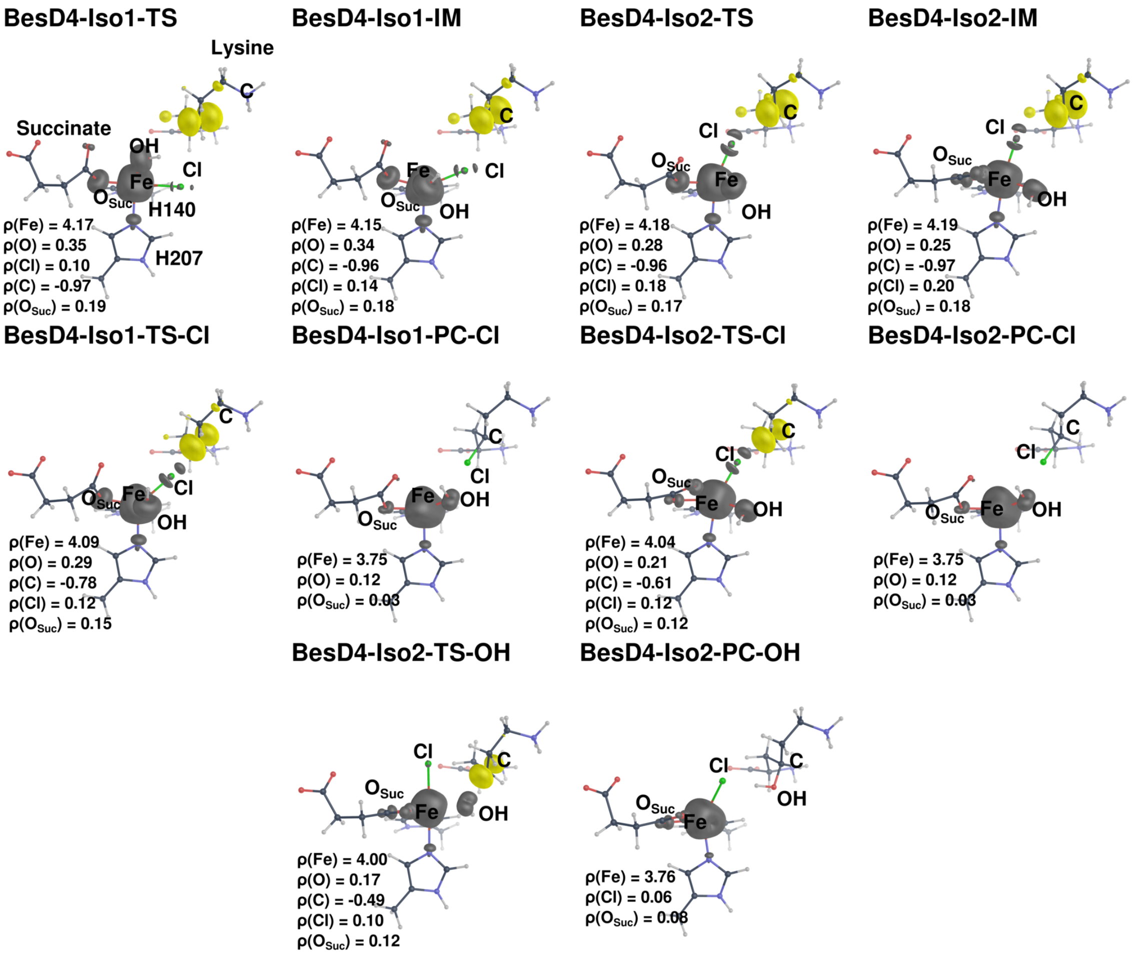

Figure S13. Spin density plots for the stationary points obtained during QM/MM calculations on the BesD4-RC snapshot during and after isomerization.

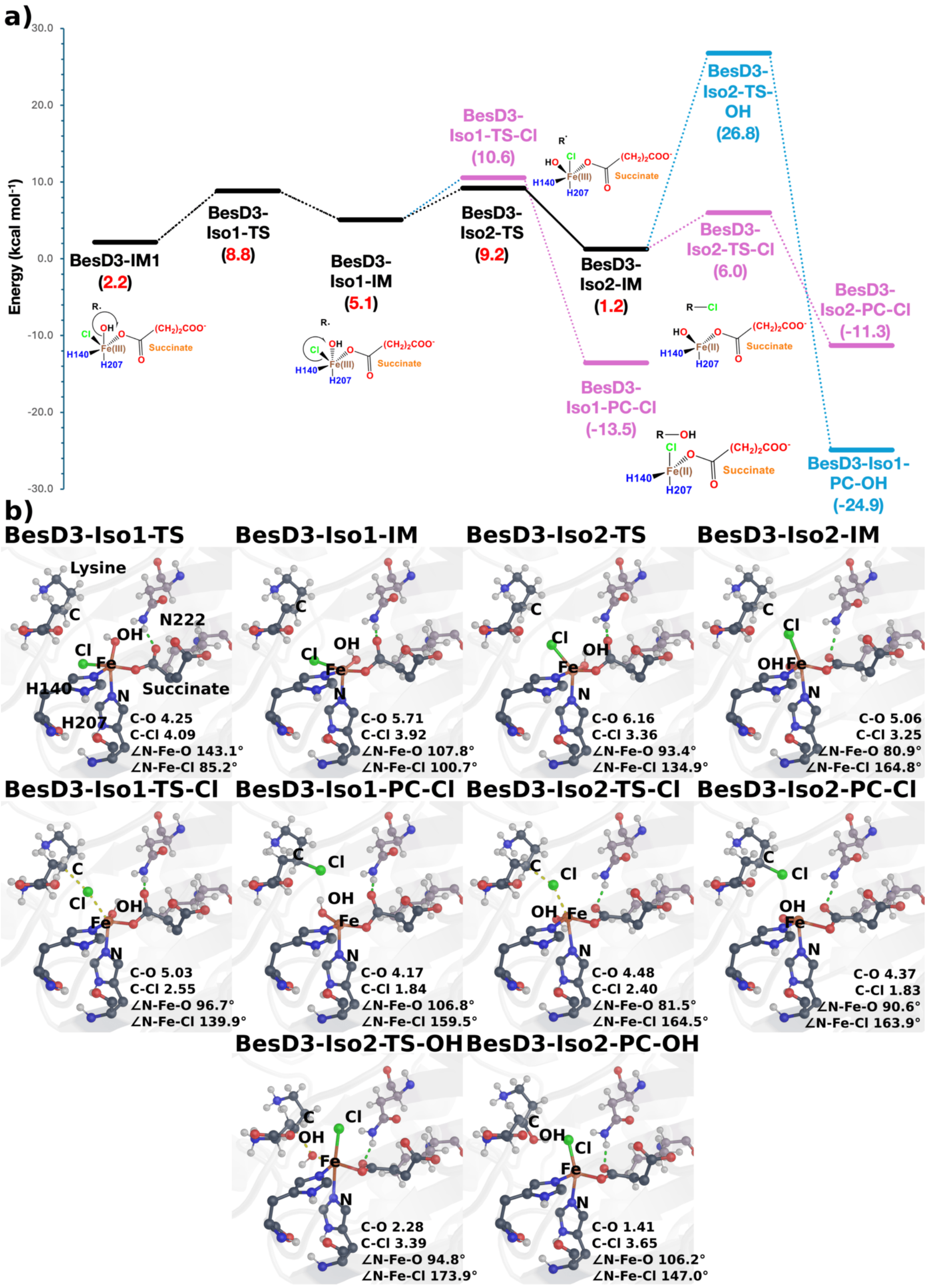

Figure S14. a) Reaction Profile of isomerization, hydroxylation, and chlorination reactions obtained from BesD3-RC, b) QM/MM optimized structures of stationary points obtained from BesD3-RC. Distances are mentioned in Å, and energies are mentioned in kcal/mol at the QM(B3)/MM level.

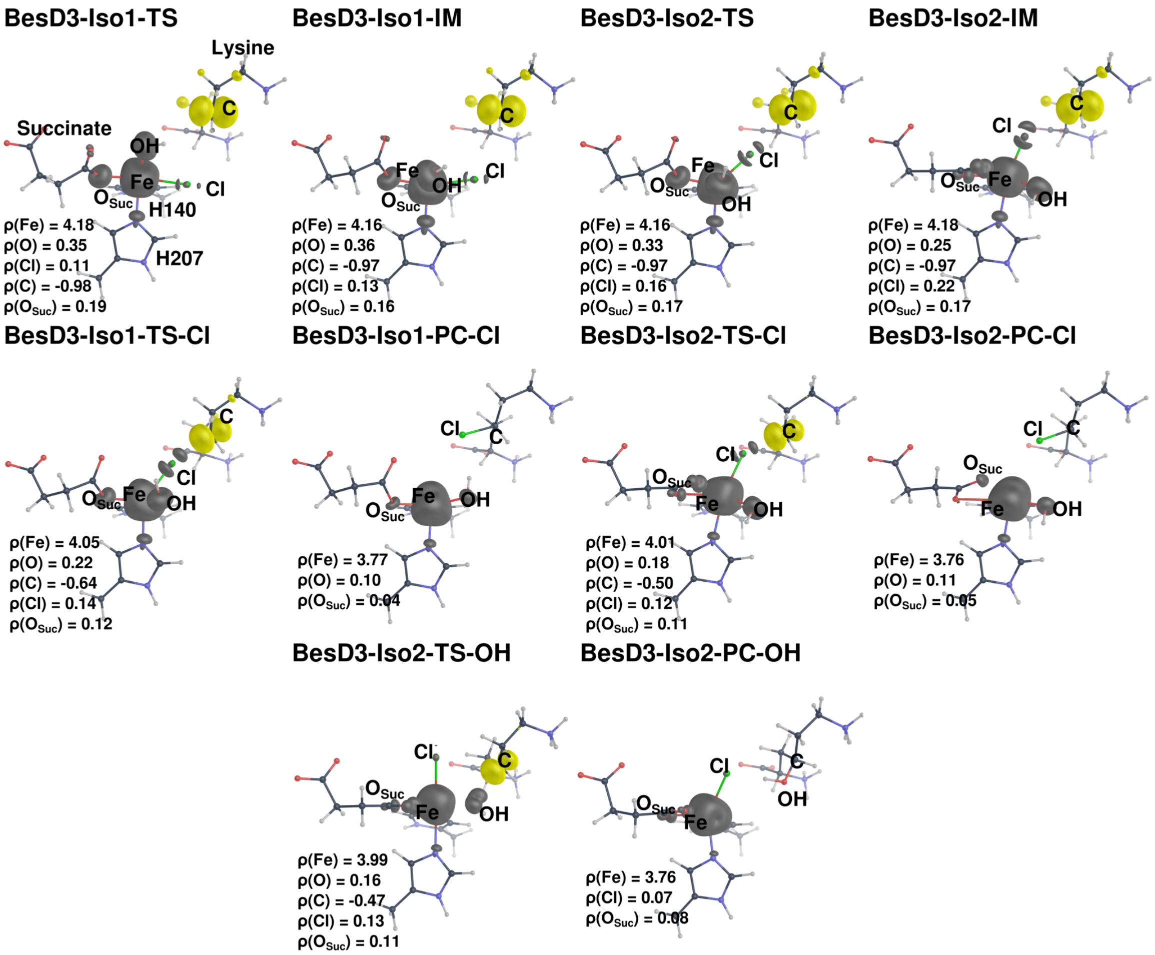

Figure S15. Spin density plots for the stationary points obtained during QM/MM calculations on the BesD3-RC snapshot during and after isomerization.

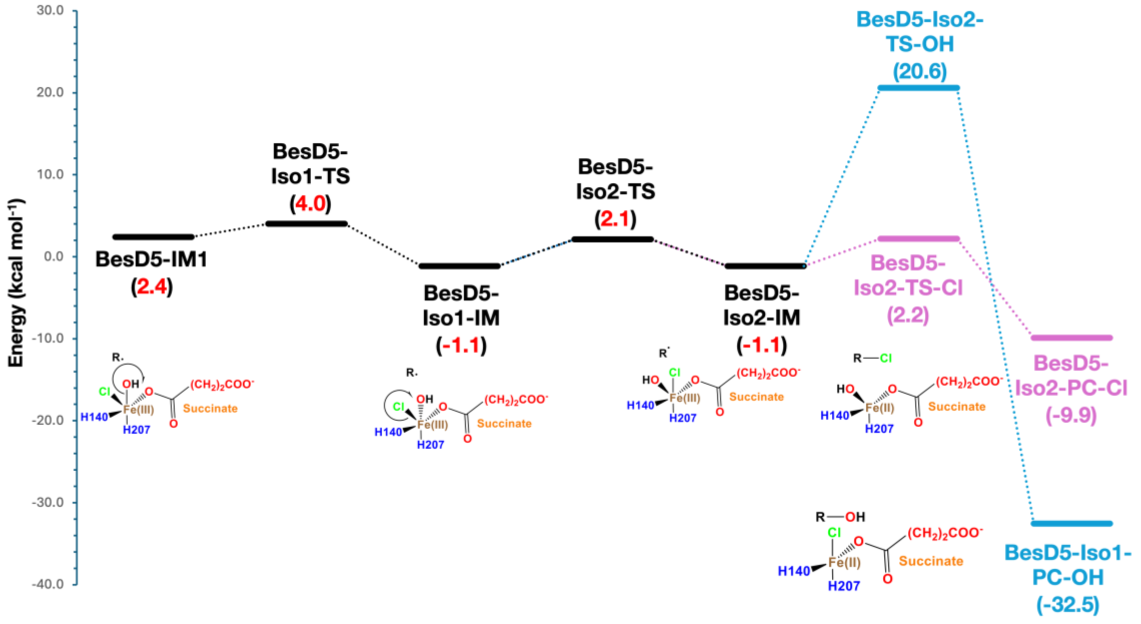

Figure S16. Reaction Profile of isomerization, hydroxylation, and chlorination reactions obtained from BesD5-RC.

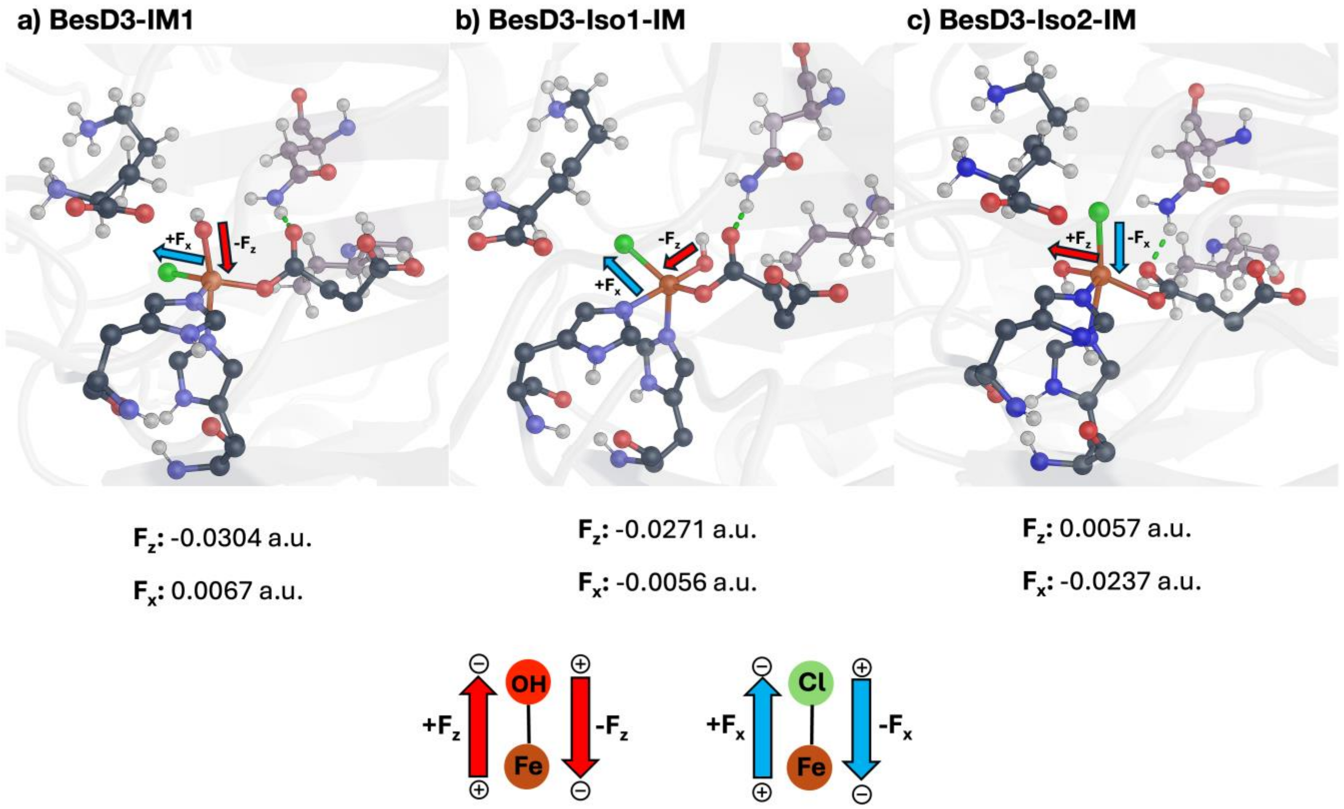

Figure S17. IEF variation along the Fe–Cl and Fe–O bonds in Cl–Fe(III)–OH isomers of BesD3-RC. The IEF vectors are defined from positive to negative according to the TITAN convention. The red arrows indicate the field components, with the z-axis aligned along the Fe–O bond and the x-axis along the Fe–Cl bond (shown in blue).

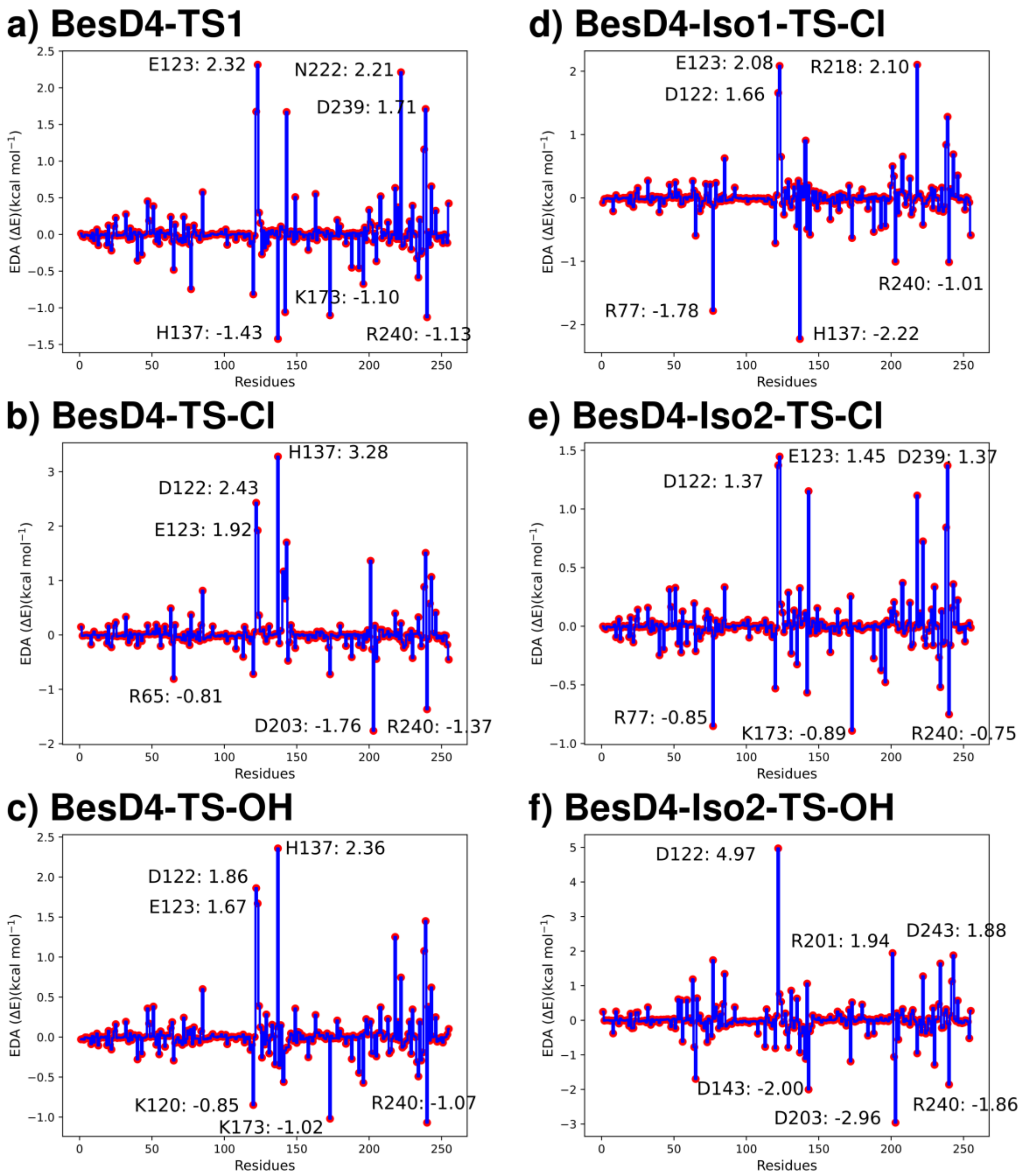

Figure S18. EDA plots of TS states obtained during HAT, hydroxylation, and chlorination reactions from BesD4-RC snapshot.

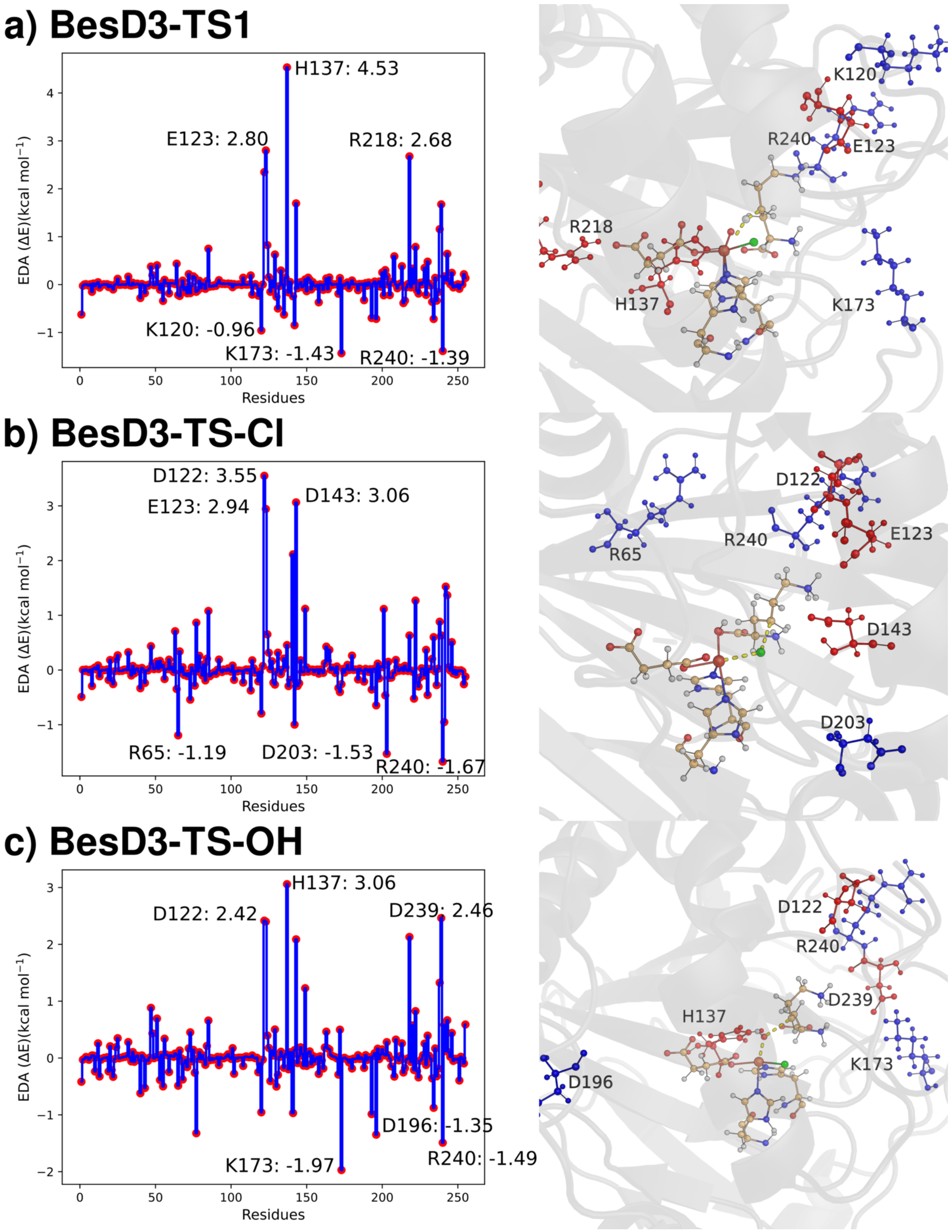

Figure S19. EDA analysis of HAT, hydroxylation, chlorination reactions of BesD3-RC snapshot.

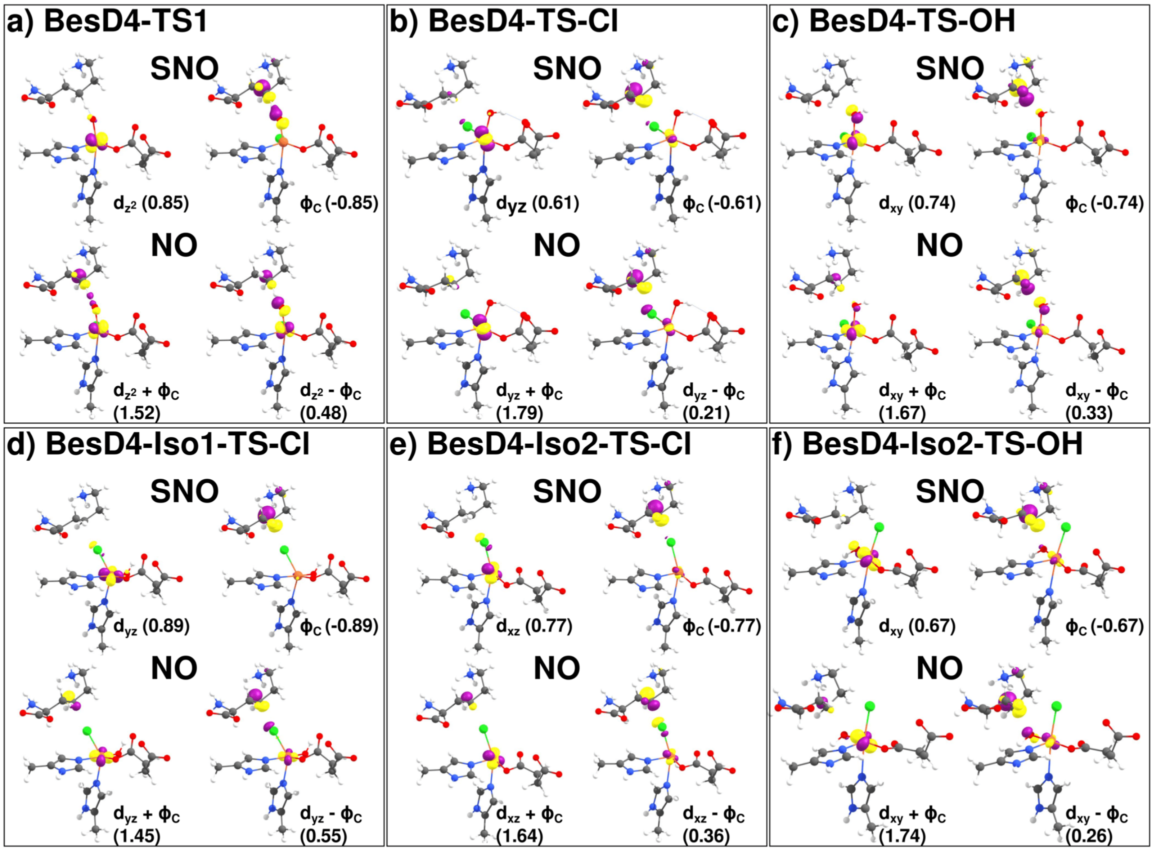

Figure S20. Spin Natural Orbital (SNO) and Natural Orbital (NO) analysis of the TS states of HAT, chlorination, and hydroxylation originating from different isomers of Cl-Fe(III)-OH intermediate obtained from BesD4-RC, a) BesD4-TS1, b) BesD4-TS-Cl, c) BesD4-TS-OH, d) BesD4-Iso1-TS-Cl, e) BesD4-Iso2-TS-Cl and f) BesD4-Iso2-TS-OH.

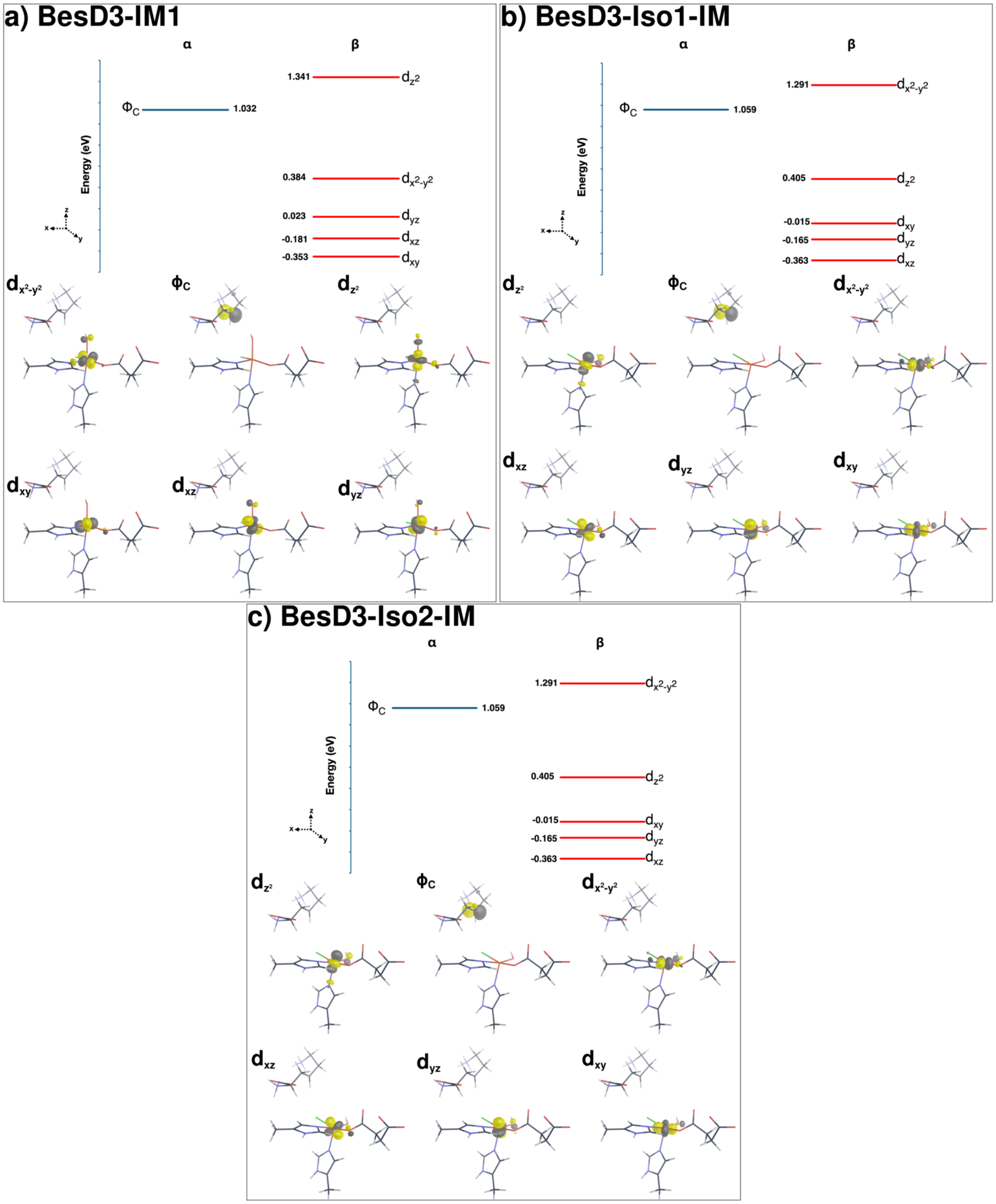

Figure S21. FMO analysis of the isomers of Cl-Fe(III)-OH intermediates obtained from BesD3-RC, a) BesD3-IM1, b) BesD3-Iso1-IM, and c) BesD3-Iso2-IM.

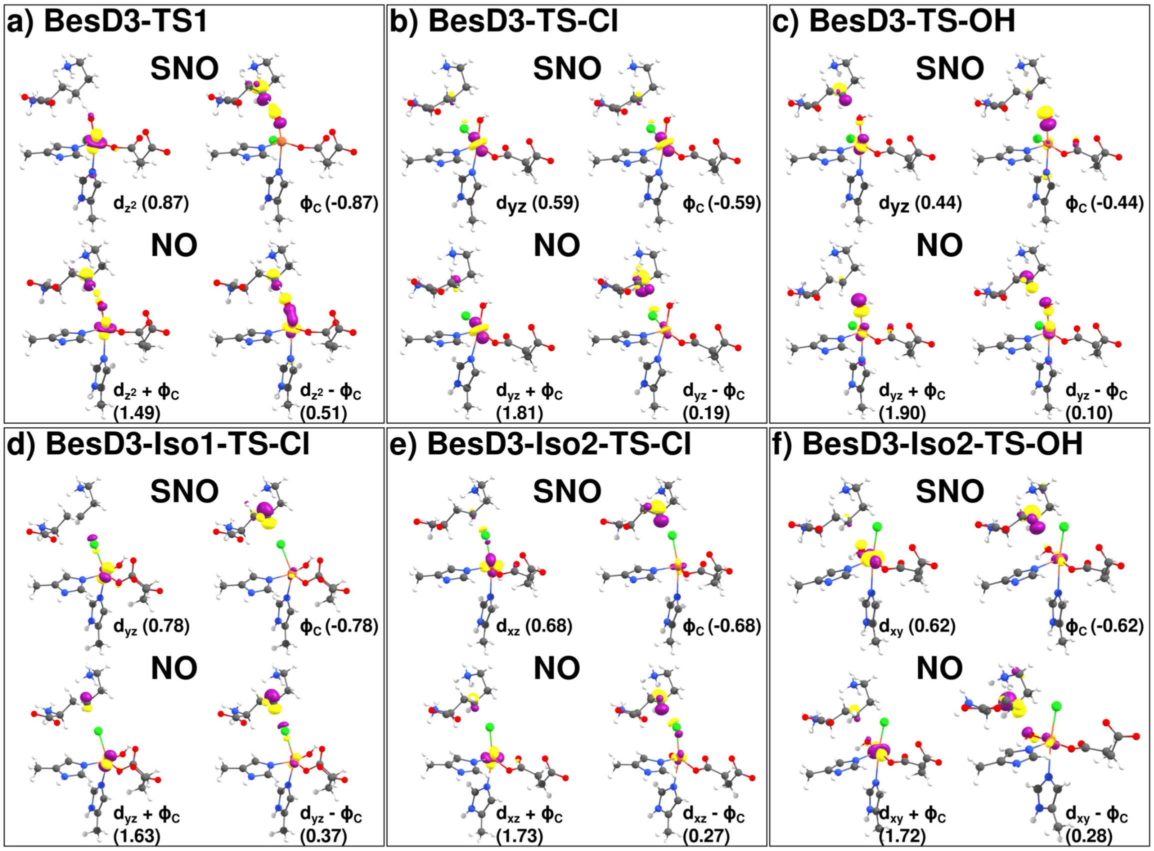

Figure S22. SNO and NO analysis of the TS states of HAT, chlorination, and hydroxylation originating from different isomers of Cl-Fe(III)-OH intermediate obtained from BesD3-RC, a) BesD3-TS1, b) BesD3-TS-Cl, c) BesD3-TS-OH, d) BesD3-Iso1-TS-Cl, e) BesD3-Iso2-TS-Cl, and f) BesD3-Iso2-TS-OH.

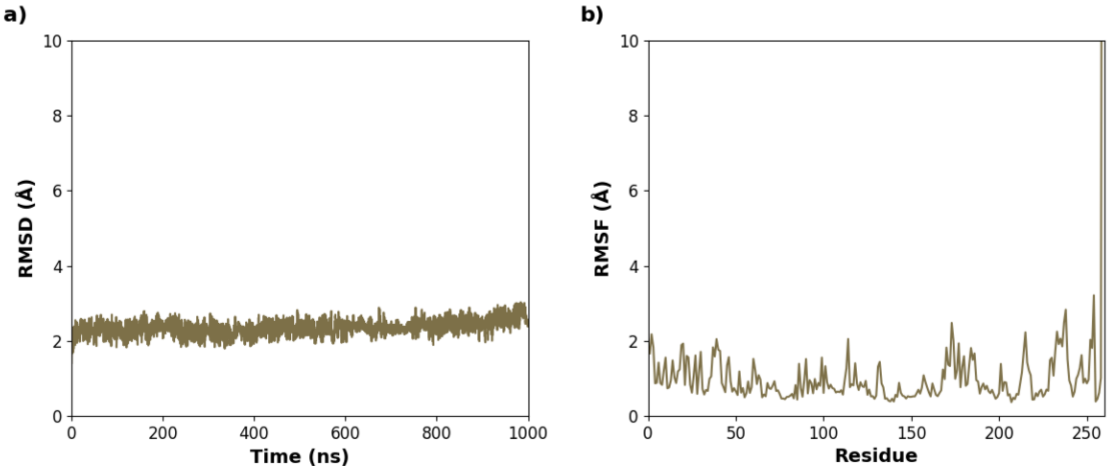

Figure S23. Conformational Dynamics of Hydrox-Fe(IV)=O•L-Lys system. a) RMSD of the dynamics suggests that the system is equilibrated, b) RMSF of the system identifies flexible regions.

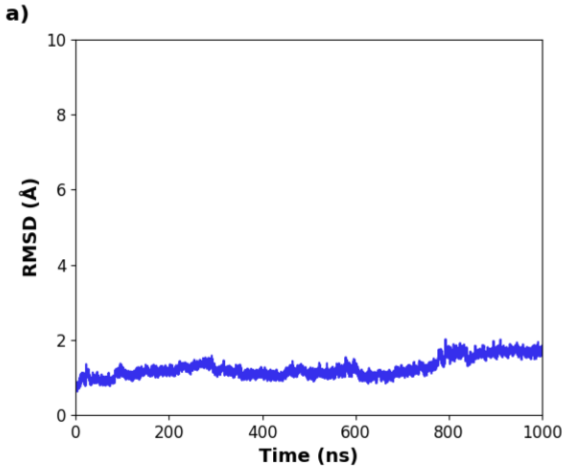

Figure S24. Conformational Dynamics of Hydrox-3R-Fe(IV)=O•L-Lys system. a) RMSD of the dynamics suggests that the system is equilibrated, b) RMSF of the system identifies flexible regions.

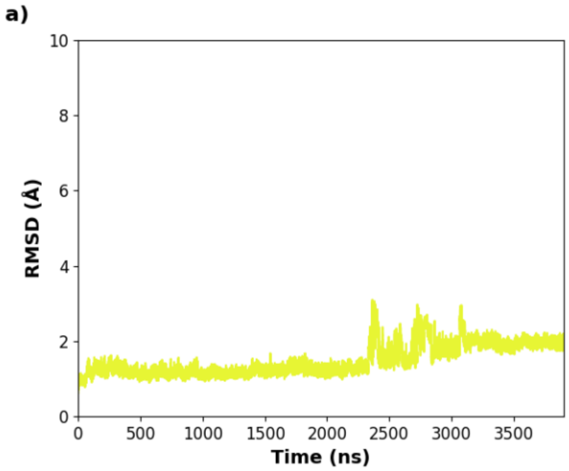

Figure S25. Conformational Dynamics of Chimera14-Fe(IV)=O•L-Lys system. a) RMSD of the dynamics suggests that the system is equilibrated, b) RMSF of the system identifies flexible regions.

2. MD simulation of the Fe(IV)=O Intermediate in Hydrox, Hydrox-3R and Chimera14

We first analyzed the interactions surrounding the L-Lys substrate. As in the native halogenase BesD, the substrate's amino group forms salt bridge interactions with D239, D143, and E123, while its carboxylate group engages in hydrogen-bonding interactions with R77, H137, and W241 in all three systems. Despite these conserved SCS interactions, the L-Lys substrate adopts distinct conformations in the three enzymes due to differences in the stability of these interactions. For example, in Hydrox, the C4 carbon maintains an average distance of 3.61 Å from the Fe(IV)=O, and the *pro-R* hydrogen is positioned more favorably for HAT with an average distance of 3.15 Å compared to 3.61 Å for the *pro-S* hydrogen (**Figure S26**). Comparatively, in the halogenase variants Hydrox-3R and Chimera14, the C4 carbon of the L-Lys substrate is positioned slightly further from the ferryl oxygen, with average distances of 4.23 Å and 4.53 Å, respectively (**Figure S27** and **Figure S28**). These values are consistent with the 4.45 Å distance observed in the native halogenase BesD. The preference for abstraction of the *pro-R* hydrogen was maintained in both engineered halogenase variants (*pro-R* vs *pro-S*, 3.85 Å vs 4.18 Å (Hydrox-3R), 3.94 Å vs 4.90 Å (Chimera14)), mirroring the preference observed in Hydrox and in native BesD (**Figure S27** and **Figure S28**). The closer approach of the substrate in Hydrox aligns with experimental observation of its higher turnover of hydroxylated substrate.[1] Atomistic analysis of the Hydrox–Fe(IV)=O•L-Lys trajectories showed that the non-Fe-coordinated carboxylate of succinate is stabilized through hydrogen-bonding interactions with H137, T128, and R219. In contrast, in Hydrox-3R–Fe(IV)=O•L-Lys and Chimera14–Fe(IV)=O•L-Lys, this succinate carboxylate retains hydrogen bonds with H137 and R219 (R218 in Chimera-14), but the interaction with T128 is lost, an outcome consistent with MD simulations of the native halogenase BesD, where the T128 interaction is also absent. In the halogenase variants, the Fe-coordinated succinate carboxylate forms an additional hydrogen bond with N223 (N222 in Chimera14) through its non-coordinating oxygen. This interaction is not observed in Hydrox, where a hydrophobic valine (V223) occupies the equivalent position. Notably, this succinate-asparagine hydrogen bond occurs more frequently in Chimera14 (50%) than in Hydrox-3R (15%).

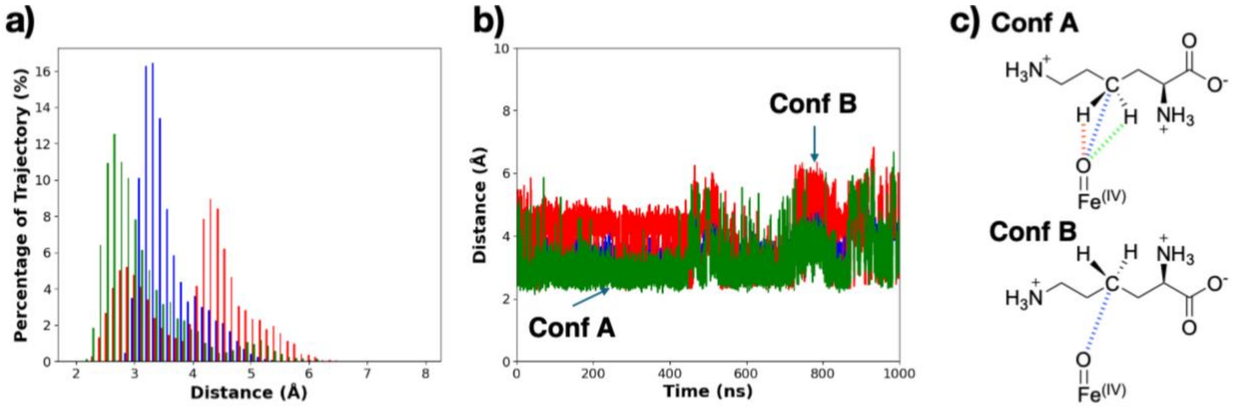

Figure S26. Substrate flexibility in Hydrox-Fe(IV)=O•L-Lys. a) Histogram plot of distance between Ferryl oxygen and C4-carbon of the substrate and hydrogens attached to it, b) Evolution of distances over time, c) ChemDraw image showing the color-coded distances depicted in (a) and (b).

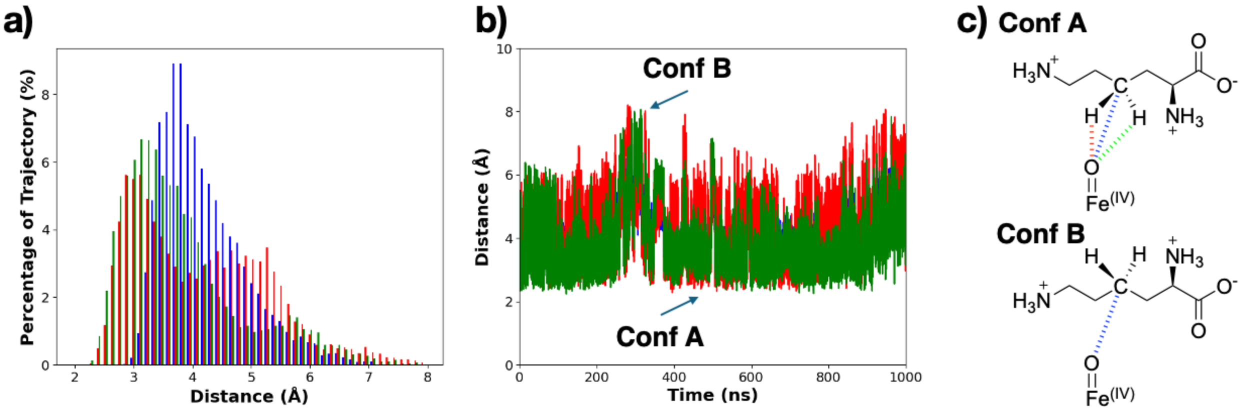

Figure S27. Substrate flexibility in Hydrox-3R-Fe(IV)=O•L-Lys. a) Histogram plot of distance between Ferryl oxygen and C4-carbon of the substrate and hydrogens attached to it, b) Evolution of distances over time, c) ChemDraw image showing the color-coded distances depicted in (a) and (b).

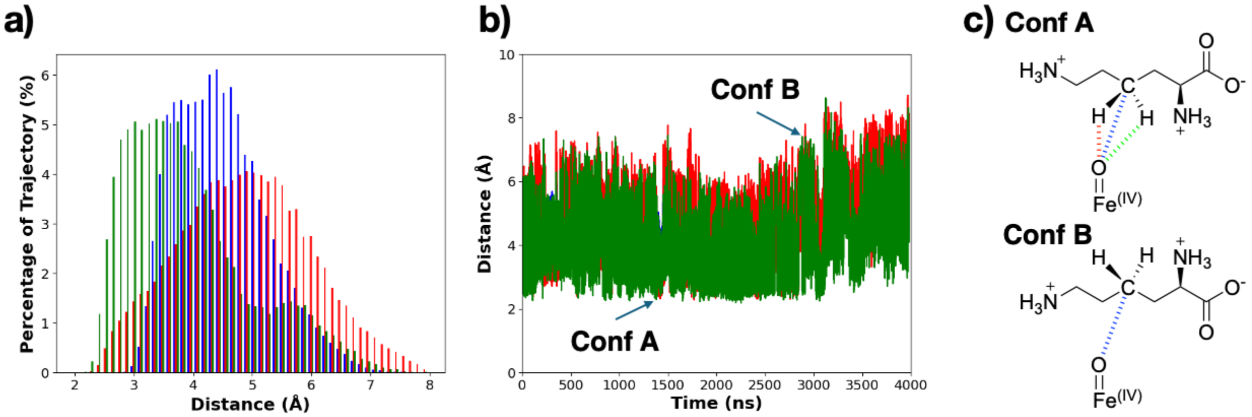

Figure S28. Substrate flexibility in Chimera14-Fe(IV)=O•L-Lys. a) Histogram plot of distance between Ferryl oxygen and C4-carbon of the substrate and hydrogens attached to it, b) Evolution of distances over time, c) ChemDraw image showing the color-coded distances depicted in (a) and (b).

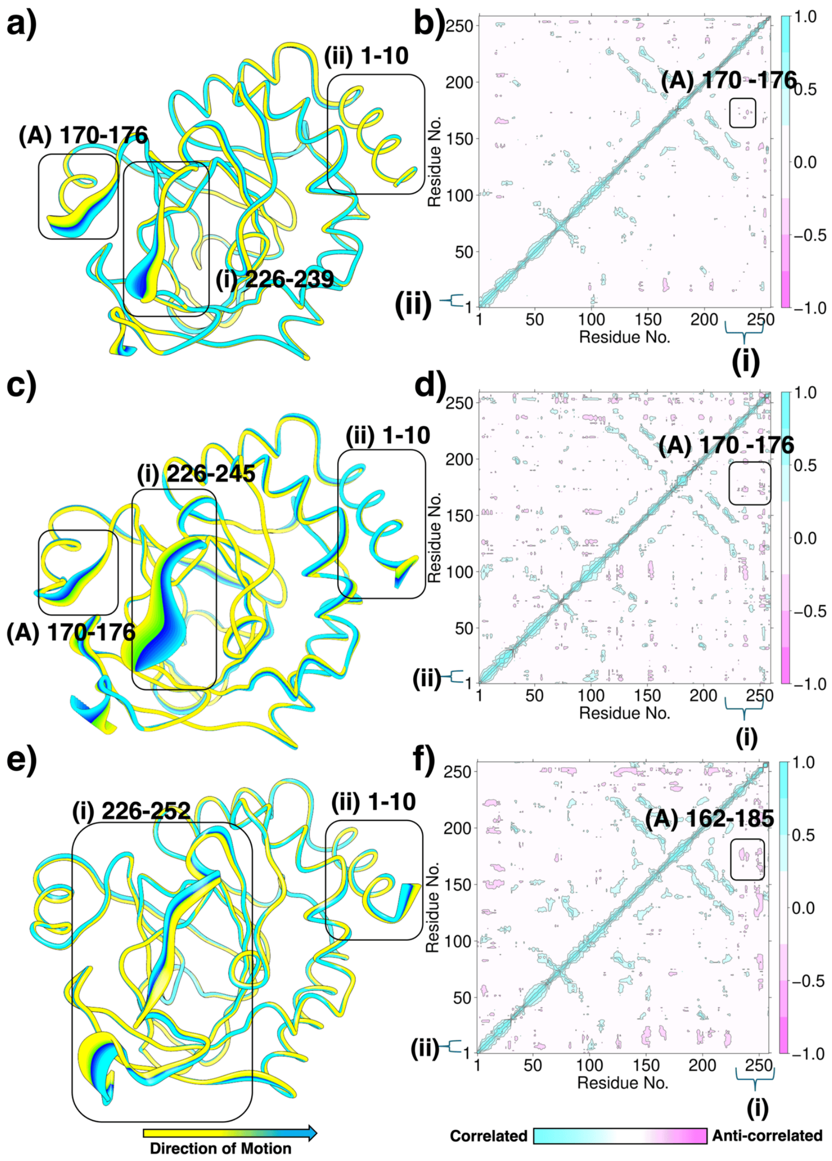

Figure S29. Conformational behavior of Fe(IV)=O species of Hydrox, Hydrox-3R, and Chimera14 variants. a), c), and e) PCA showing the dominant motions of the enzyme. Boxed regions showed dominant motions. b), d), and f) DCCA plot showing the correlated/anticorrelated motions involved in the system. Boxed regions show correlated and anticorrelated motions of the flexible regions in (a), (c), and (e).

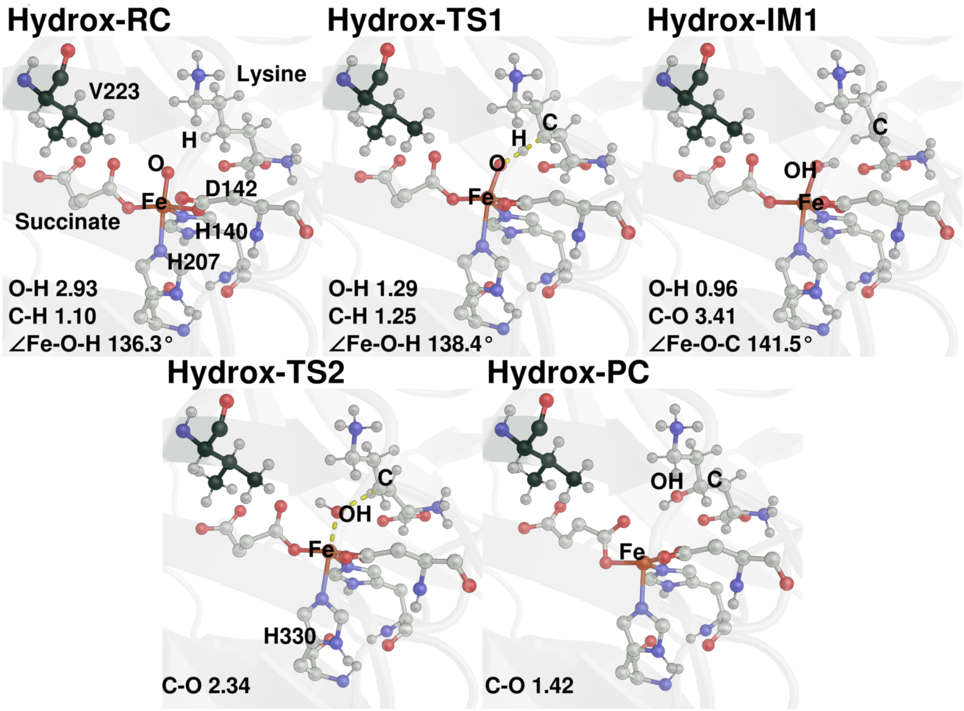

Figure S30. QM/MM optimized structures of stationary points obtained from Hydrox-RC. Distances are mentioned in Å.

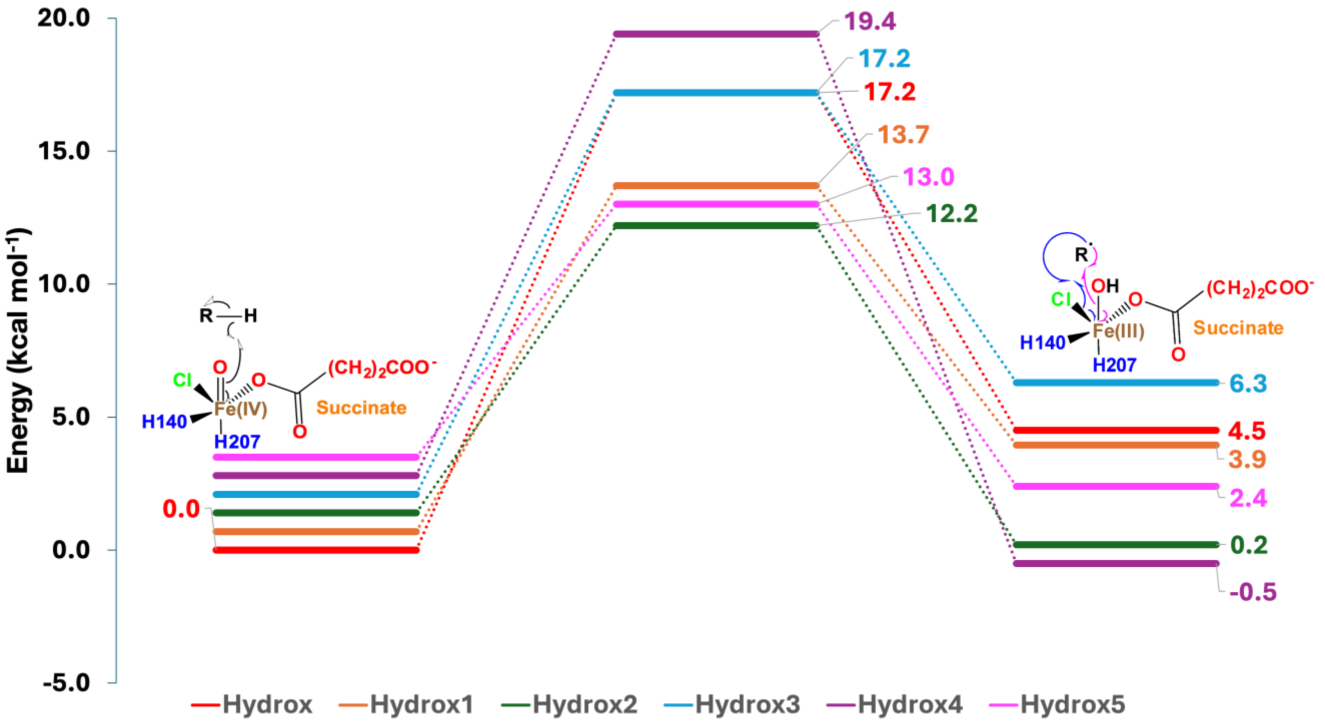

Figure S31. QM/MM reaction profiles of HAT reactions in Hydrox. The relative energies are given in kcal/mol at the QM(B3)/MM level.

3. IEF, Spin Density, and EDA analysis of HAT and Rebound Hydroxylation in Hydrox

The IEF value along the Fe-O bond was calculated as -0.0292 a.u. Spin density analyses support a σ-pathway for HAT, with spin densities of 4.26 on Fe and -0.97 on the substrate C4 carbon (**Figure S32**). We further performed EDA to probe the contributions of SCS residues during the hydroxylation mechanism. EDA predicted that residues R78, E123, and K173 stabilize the HAT TS, whereas residues H137, D122, and D240 contribute to its destabilization (**Figure S33**). For the rebound hydroxylation step, residues L126, K173, and R241 were identified as stabilizing TS (**Figure S33**). Notably, L126 forms hydrophobic interactions with the Lys substrate, which could help orient the substrate for efficient rebound hydroxylation.

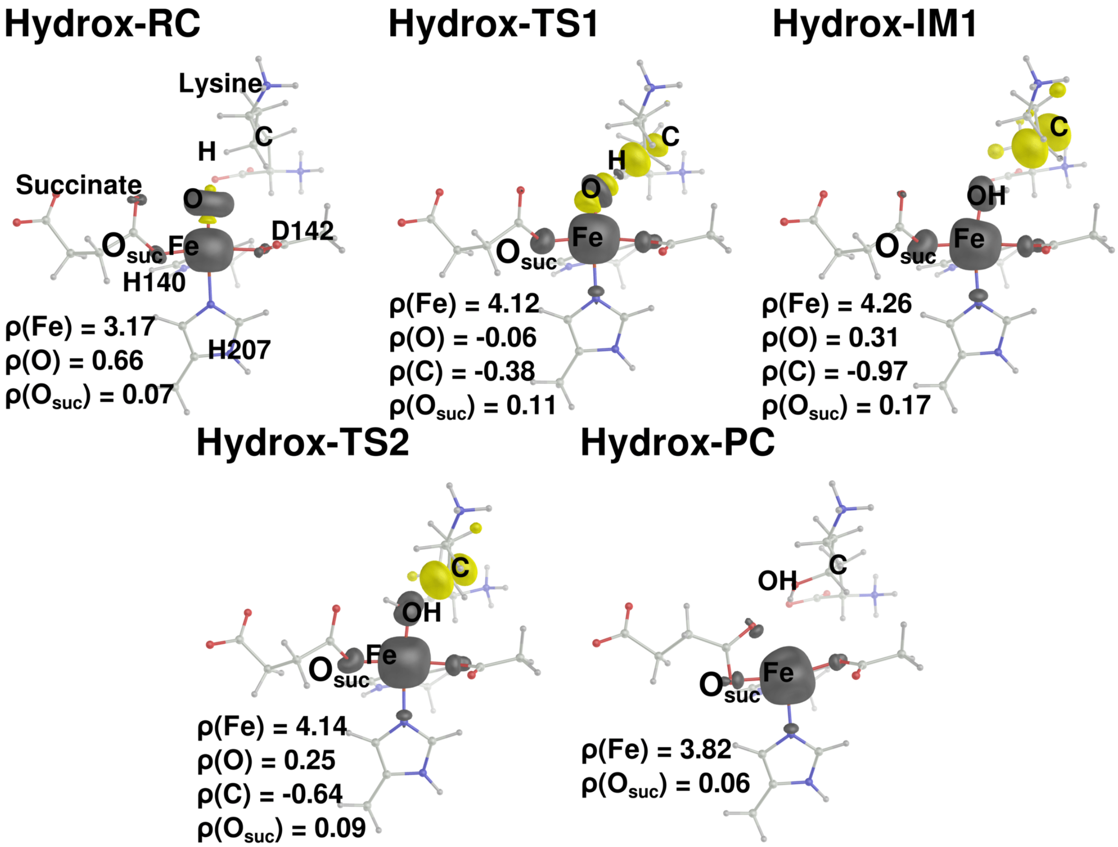

Figure S32. Spin density plots for the stationary points obtained during QM/MM calculations on the Hydrox-RC.

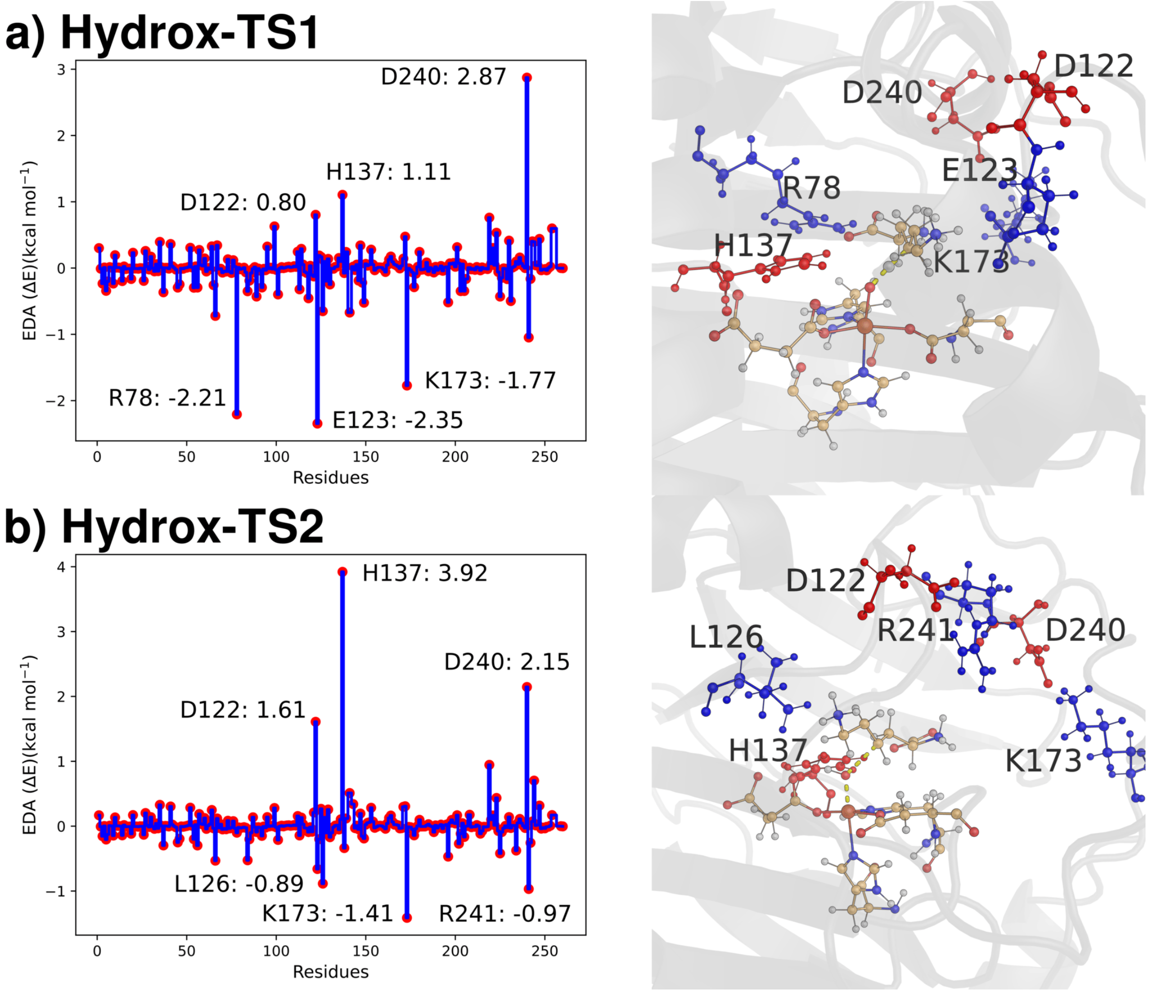

Figure S33. EDA analysis of chlorination and hydroxylation post-isomerization steps in Hydrox-RC snapshot.

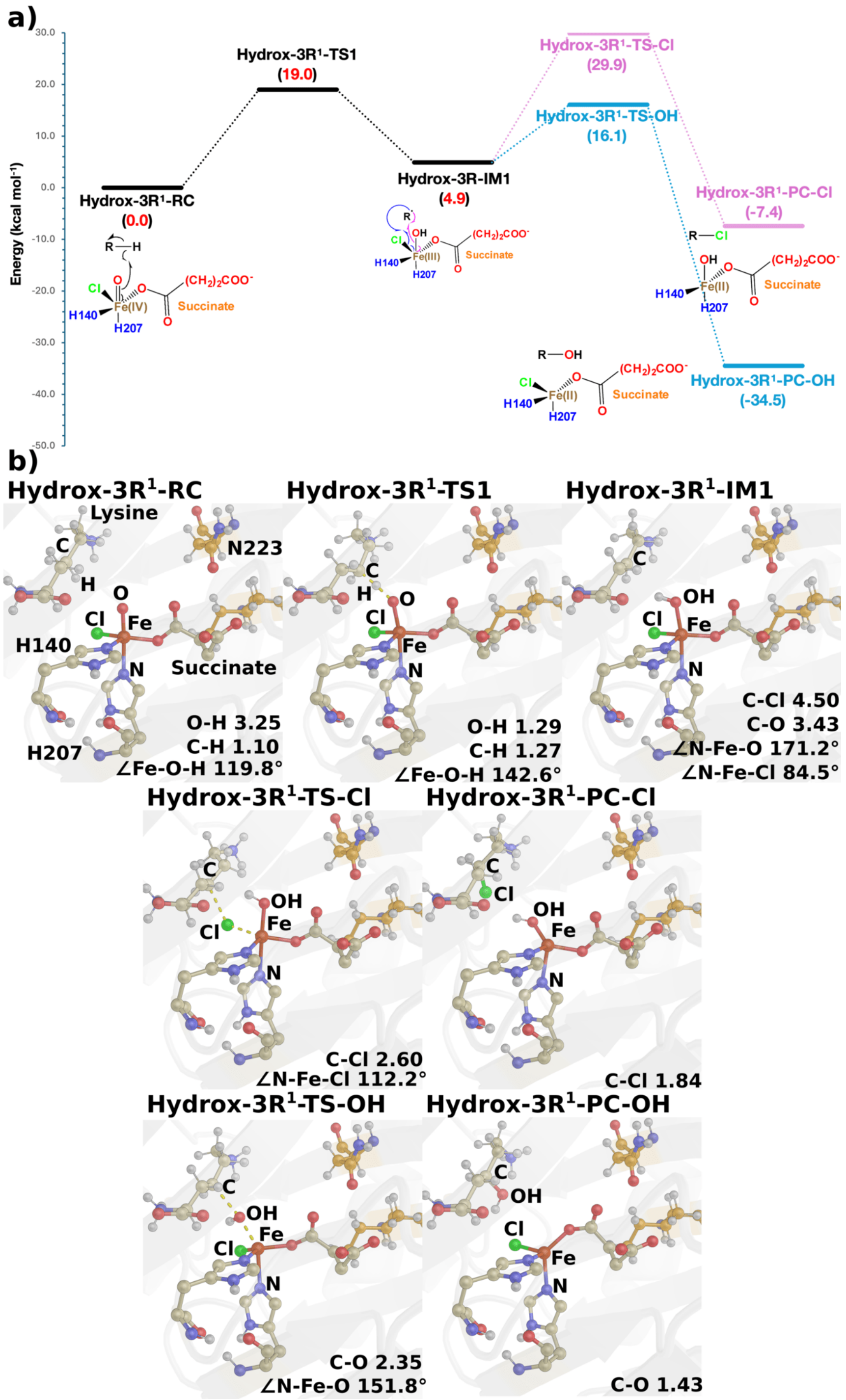

Figure S34. a) Reaction Profile of hydroxylation and chlorination reactions obtained from Hydrox-3R[1]-RC, b) QM/MM optimized structures of stationary points obtained from Hydrox-3R[1]-RC. Distances are mentioned in Å, and energies are mentioned in kcal/mol at the QM(B3)/MM level.

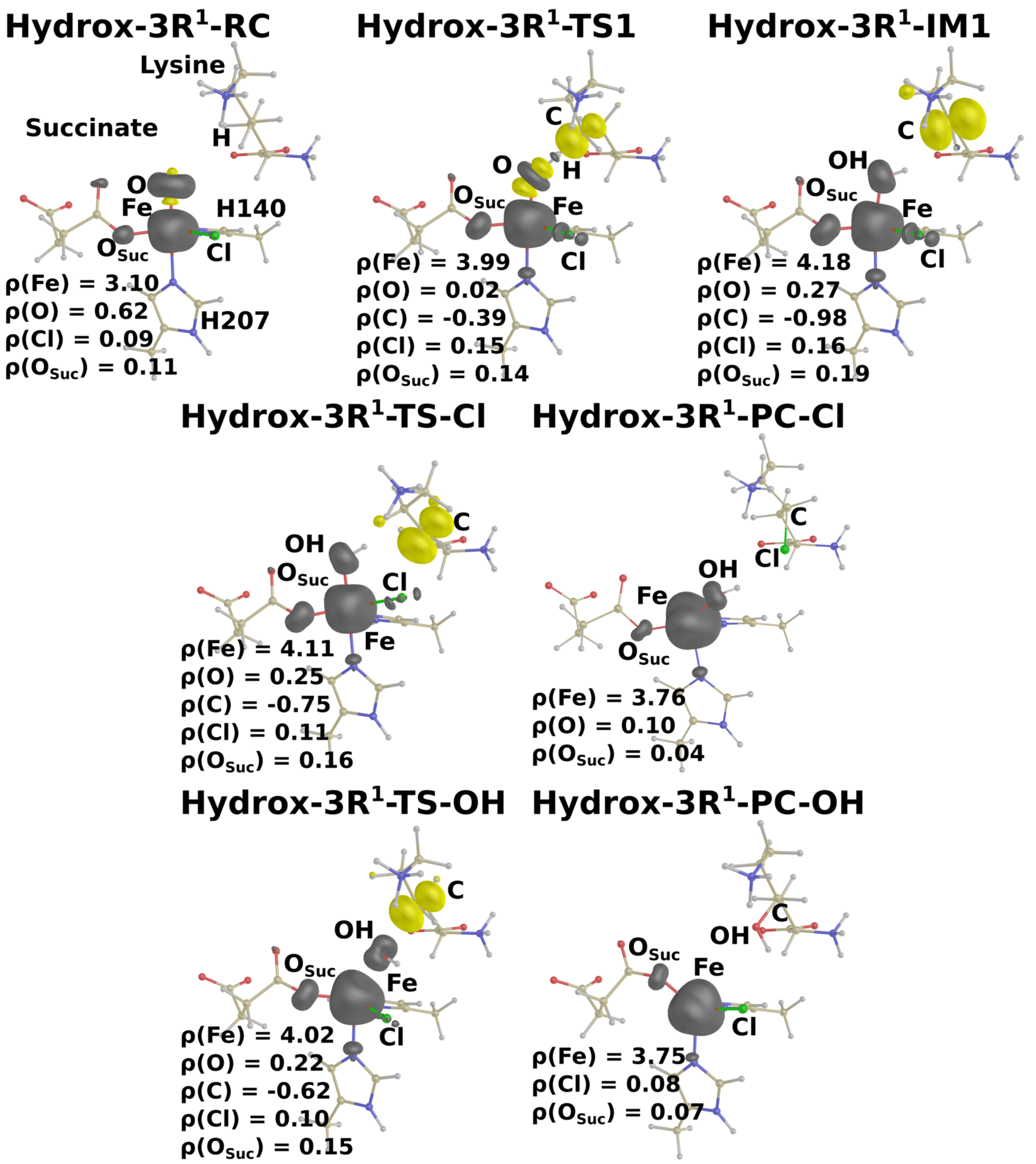

Figure S35. Spin density plots for the stationary points obtained for hydroxylation and chlorination reactions during QM/MM calculations on the Hydrox-3R[1]-RC.

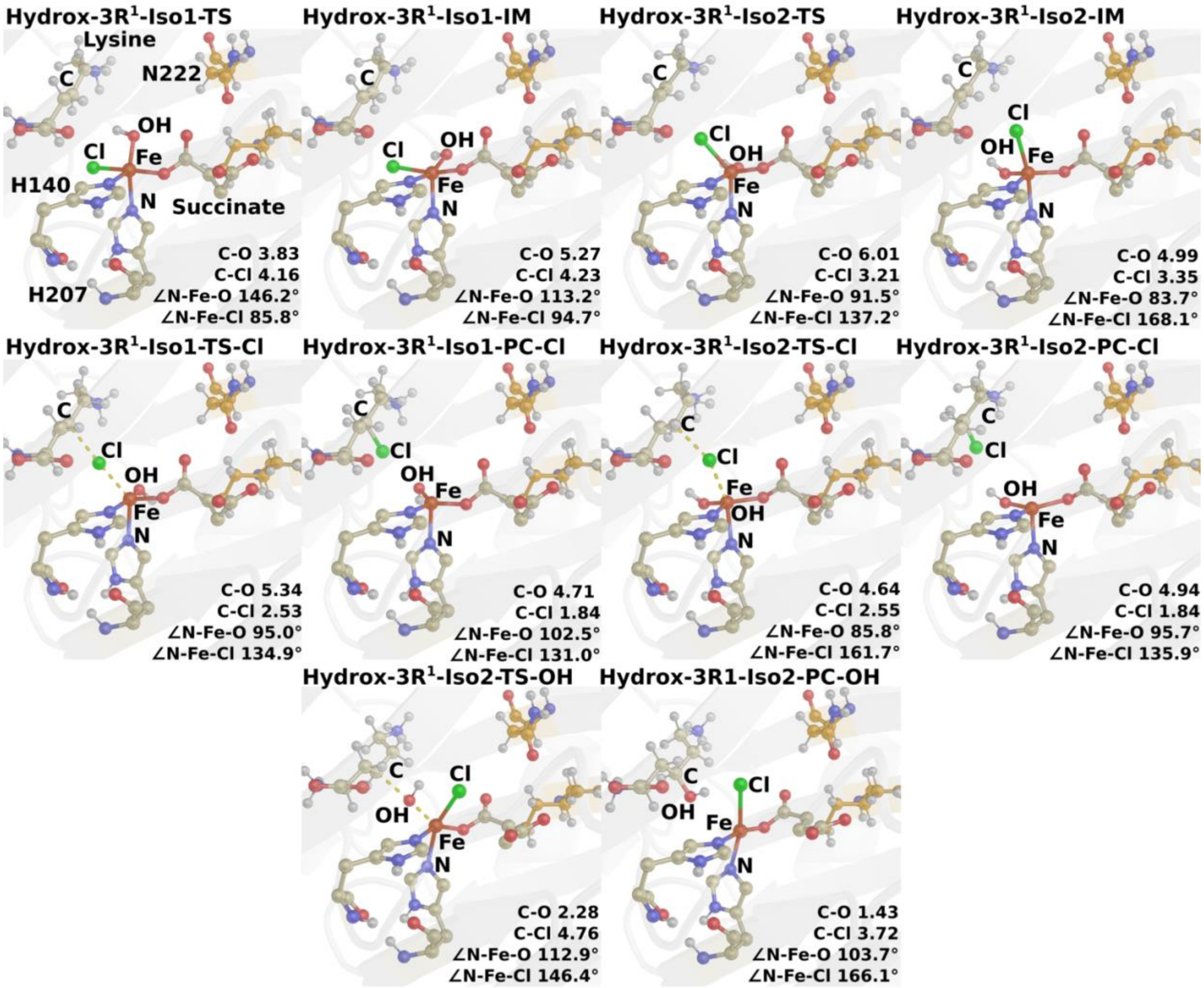

Figure S36. QM/MM optimized structures of stationary points obtained from Hydrox-3R^1-IM. Distances are mentioned in Å.

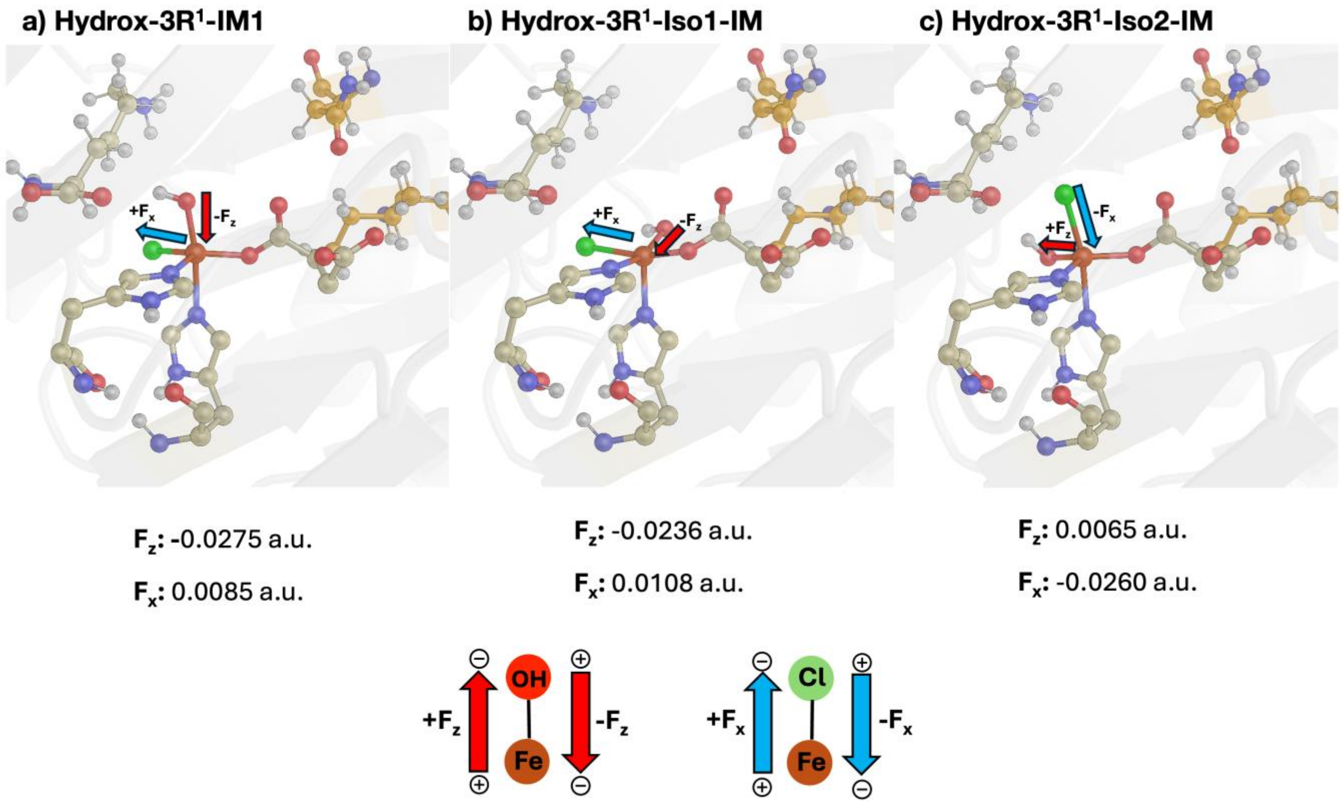

Figure S37. IEF variation along the Fe–Cl and Fe–O bonds in Cl–Fe(III)–OH isomers of Hydrox-3R[1]-RC. The IEF vectors are defined from positive to negative according to the TITAN convention. The red arrows indicate the field components, with the z-axis aligned along the Fe–O bond and the x-axis along the Fe–Cl bond (shown in blue).

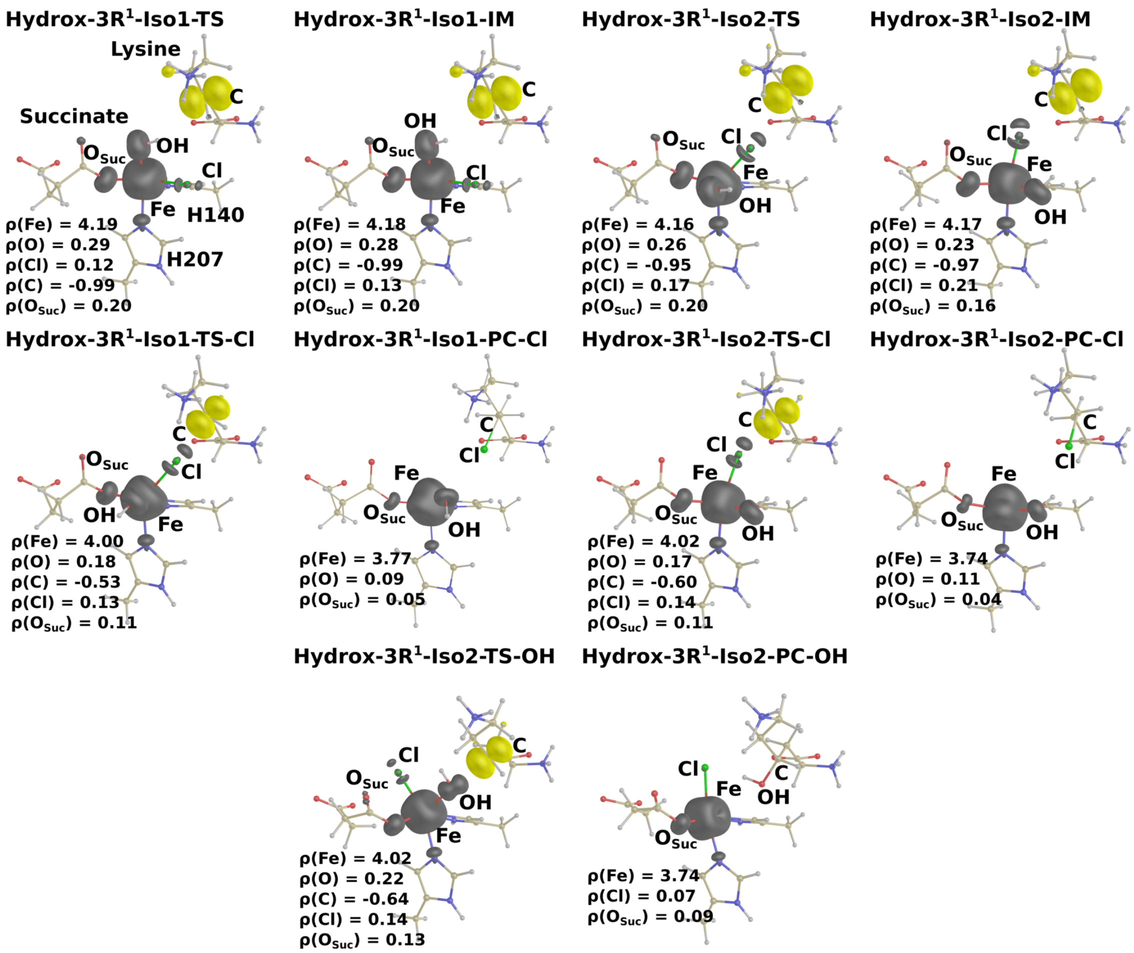

Figure S38. Spin density plots for the stationary points obtained for isomerization, hydroxylation, and chlorination reactions during QM/MM calculations on the Hydrox-3R^1-RC.

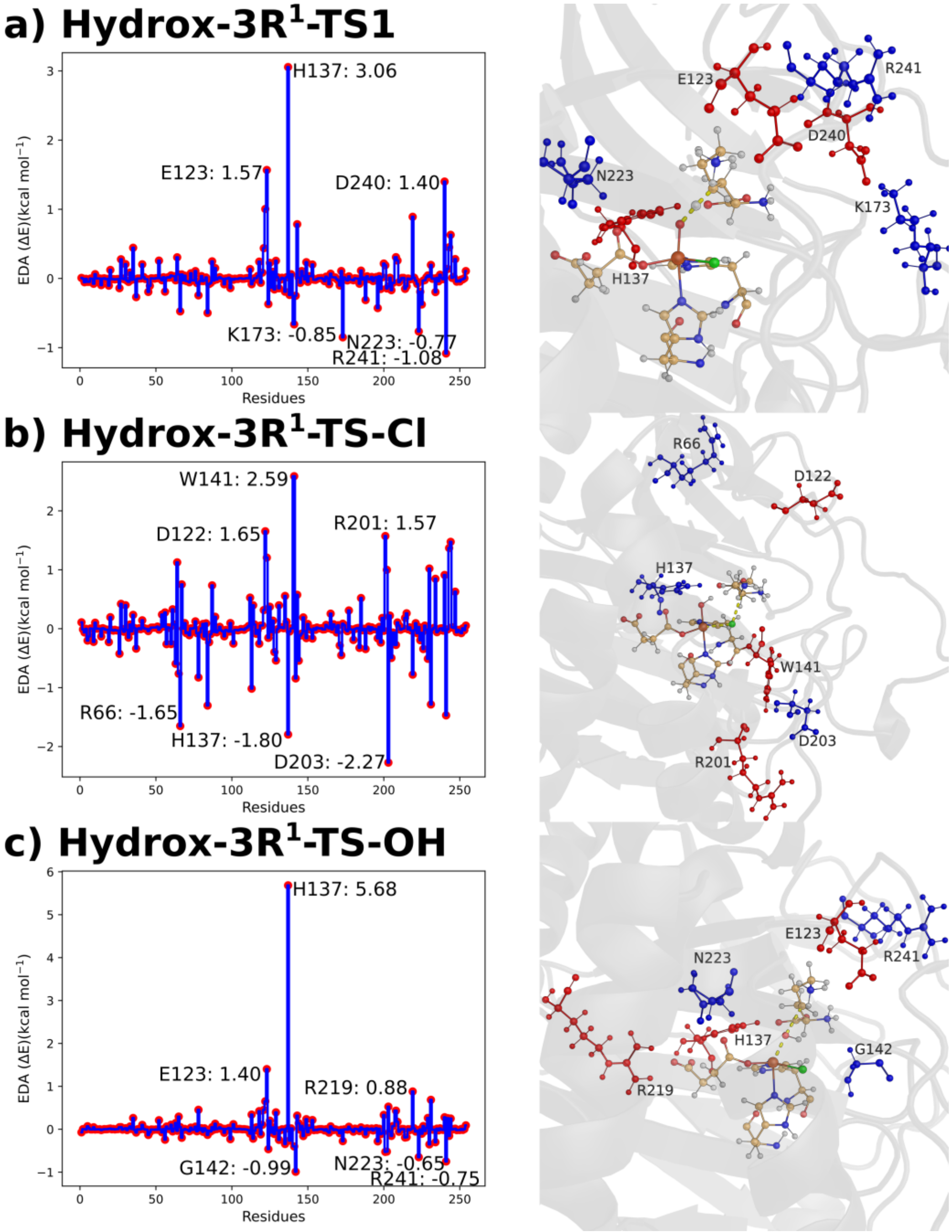

Figure S39. EDA analysis of HAT, hydroxylation, and chlorination reactions of Hydrox-3R^1-RC snapshot.

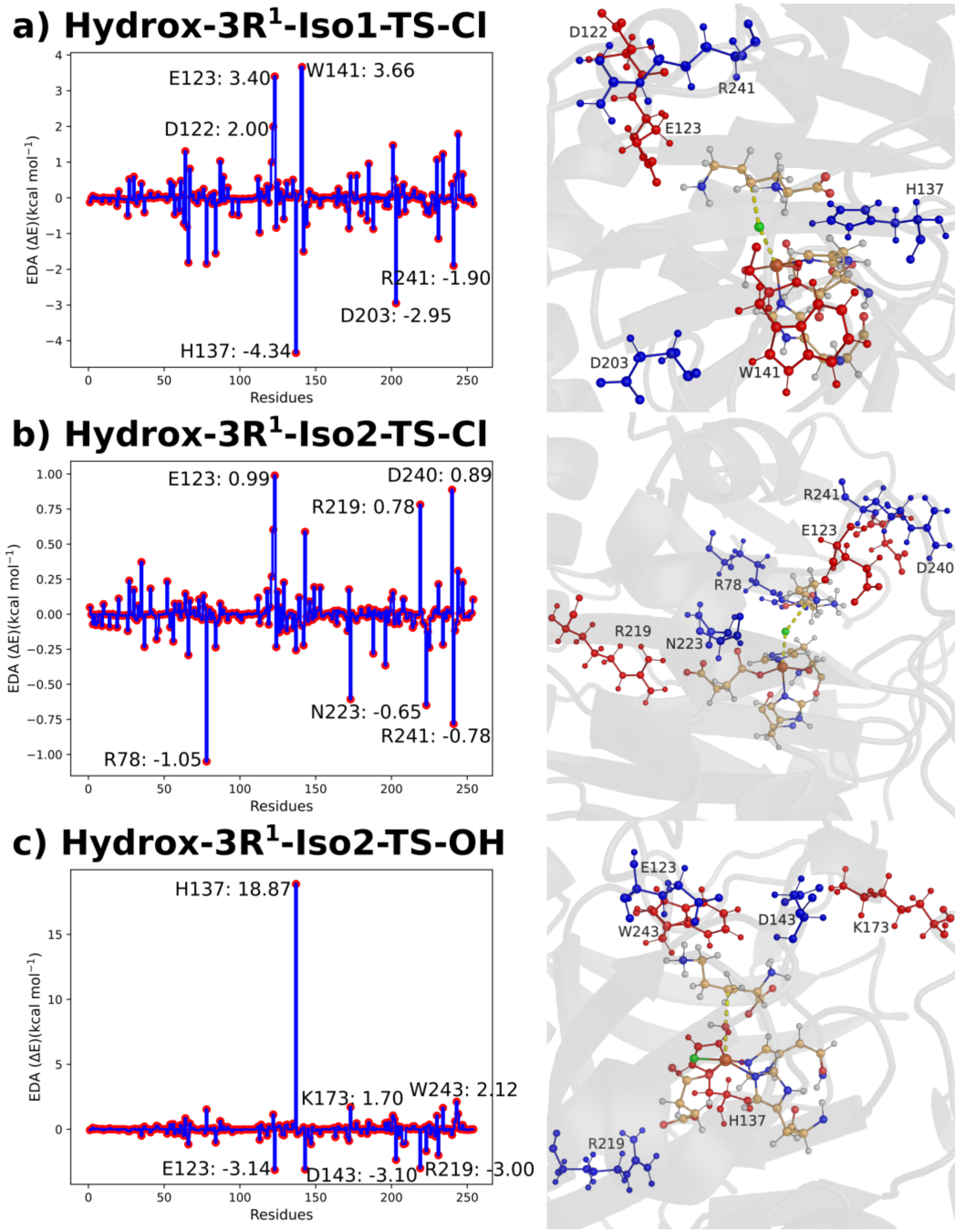

Figure S40. EDA analysis of chlorination and hydroxylation post-isomerization steps in Hydrox-3R^1-RC snapshot.

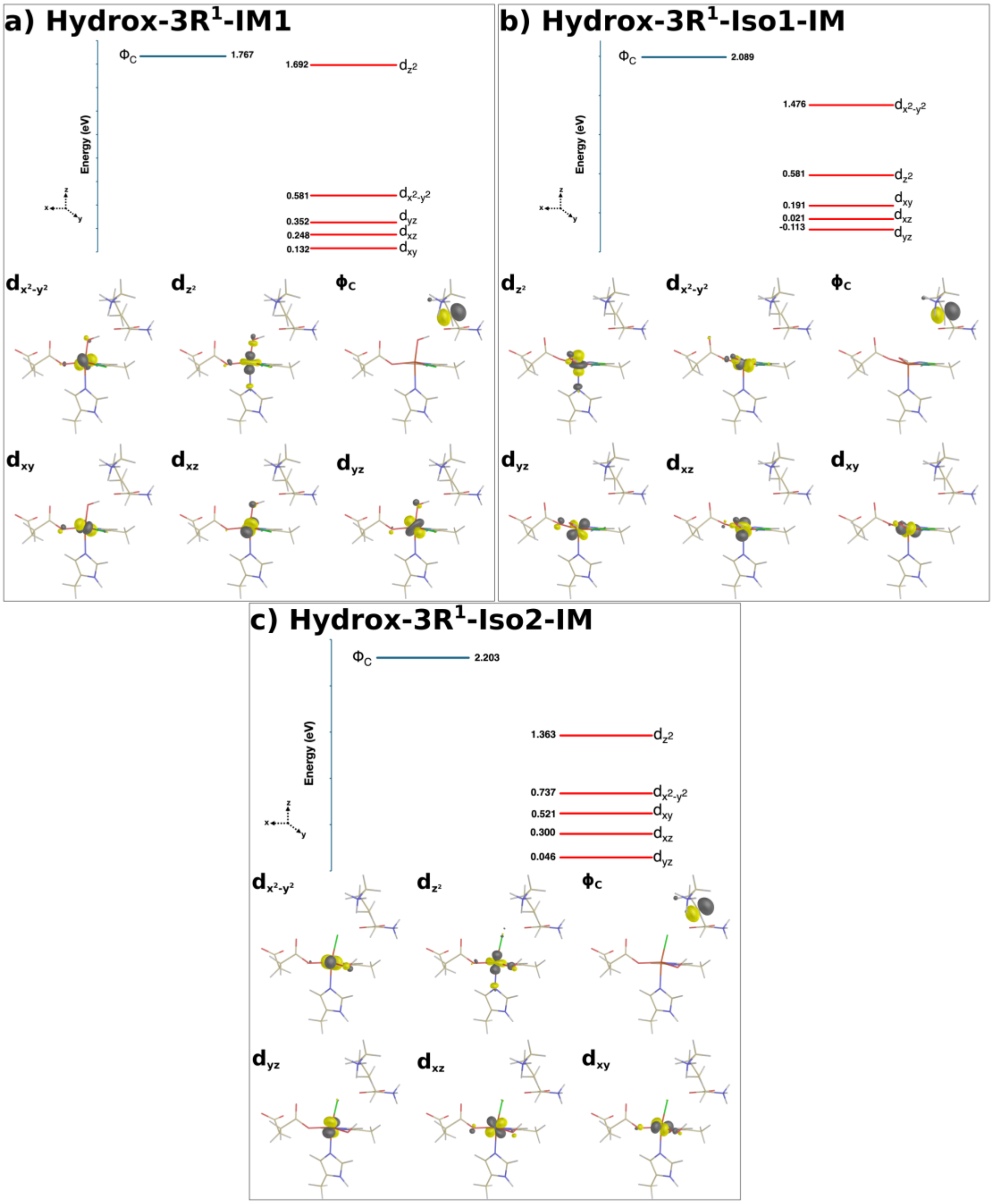

Figure S41. FMO analysis of the isomers of Cl-Fe(III)-OH intermediates obtained from Hydrox-3R^1-RC, a) Hydrox-3R^1-IM1, b) Hydrox-3R^1-Iso1-IM, and c) Hydrox-3R^1-Iso2-IM.

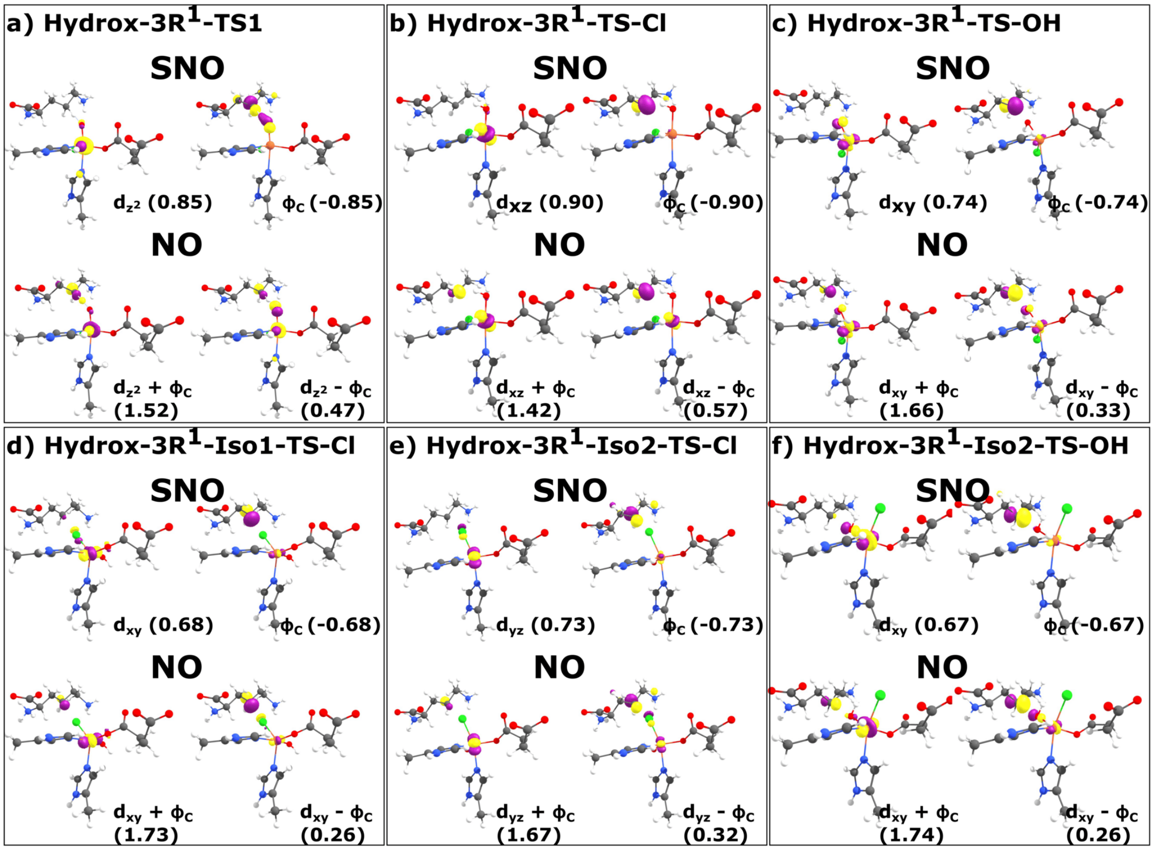

Figure S42. SNO and NO analysis of the TS states of HAT, chlorination, and hydroxylation originating from different isomers of Cl-Fe(III)-OH intermediate obtained from Hydrox-3R^1-RC, a) Hydrox-3R^1-TS1, b) Hydrox-3R^1-TS-Cl, c) Hydrox-3R^1-TS-OH, d) Hydrox-3R^1-Iso1-TS-Cl, e) Hydrox-3R^1-Iso2-TS-Cl, and f) Hydrox-3R^1-Iso2-TS-OH.

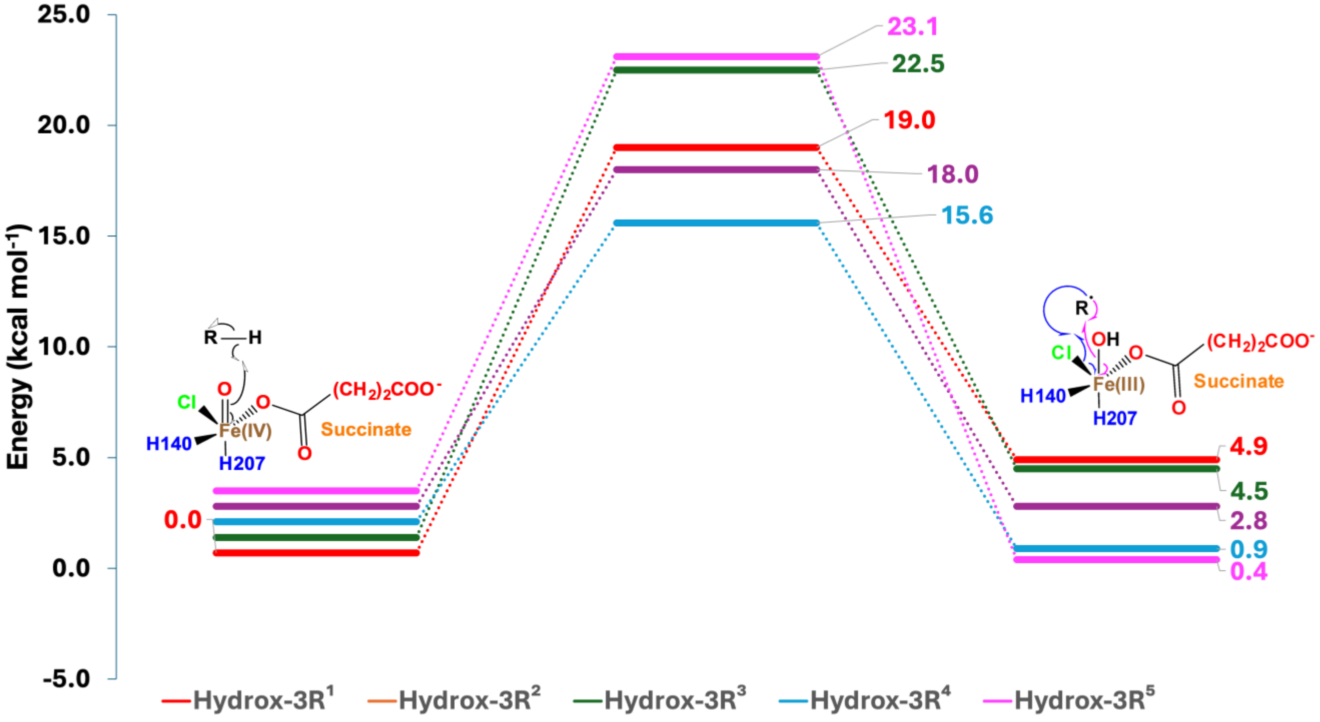

Figure S43. QM/MM reaction profiles of HAT reactions in Hydrox-3R. The relative energies are given in kcal/mol at the QM(B3)/MM level.

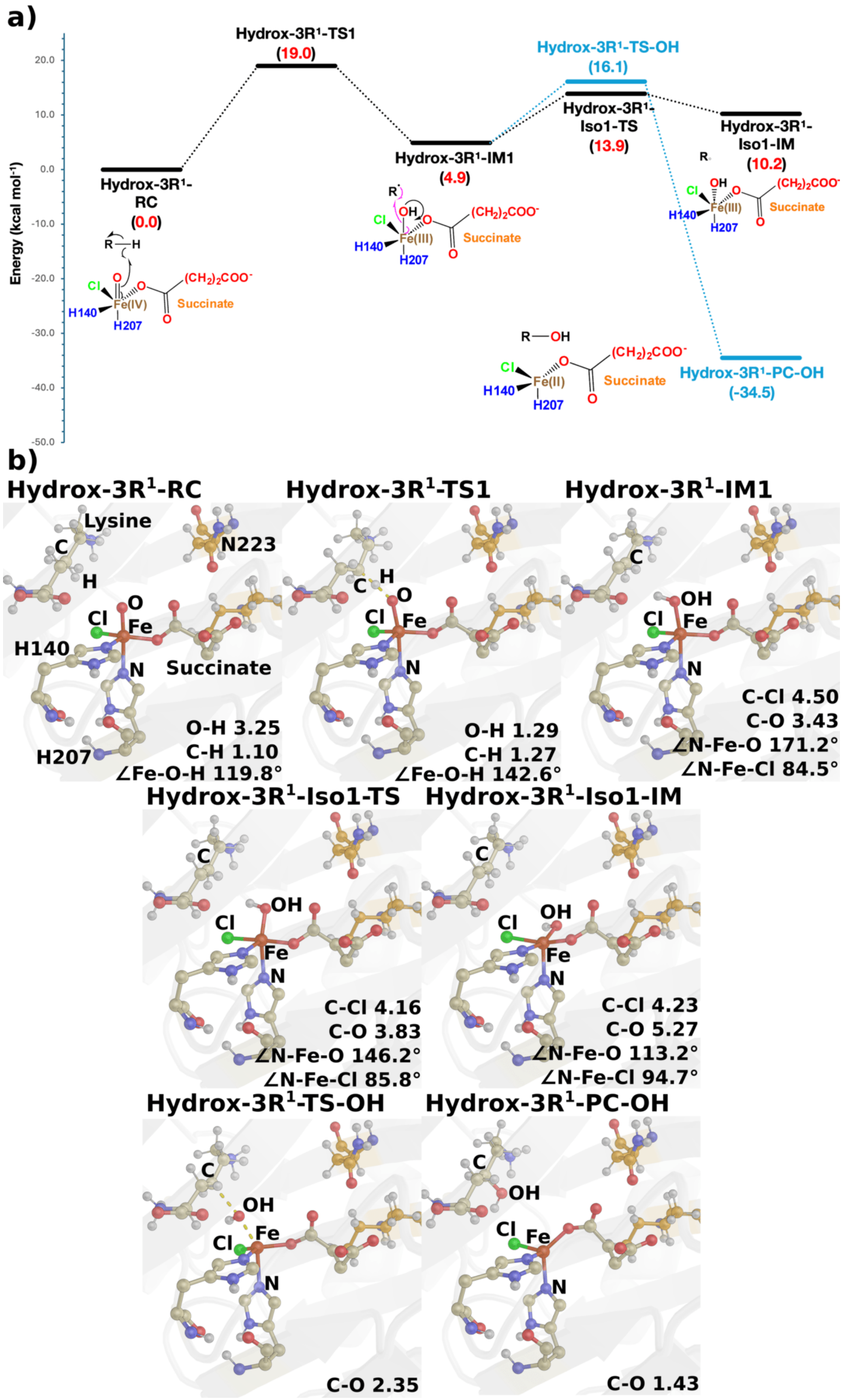

Figure S44. QM/MM reaction profile for HAT and the subsequent isomerization and rebound hydroxylation steps, starting from snapshot Hydrox-3R^1.

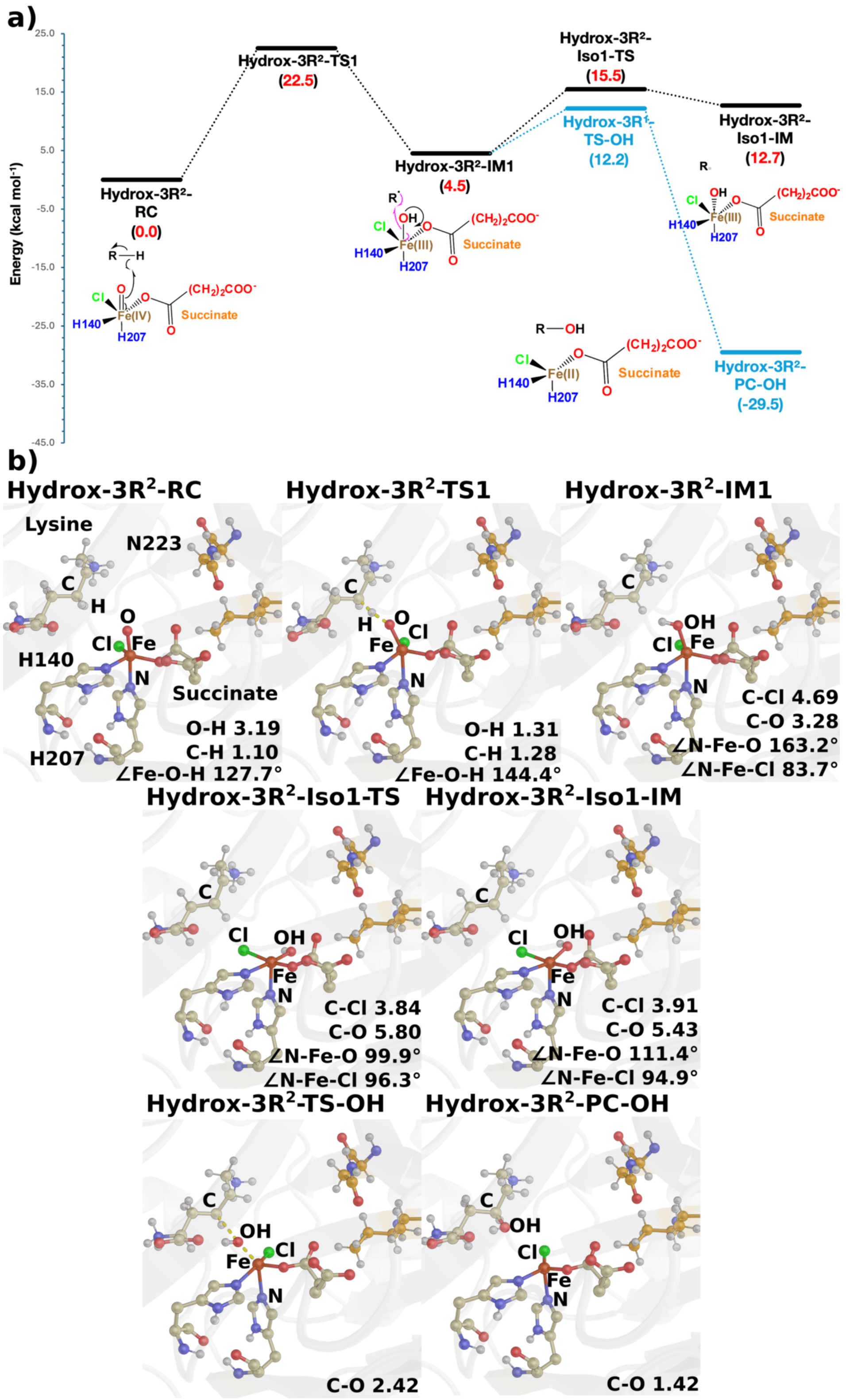

Figure S45. QM/MM reaction profile for HAT and the subsequent isomerization and rebound hydroxylation steps, starting from snapshot Hydrox-3R^2.

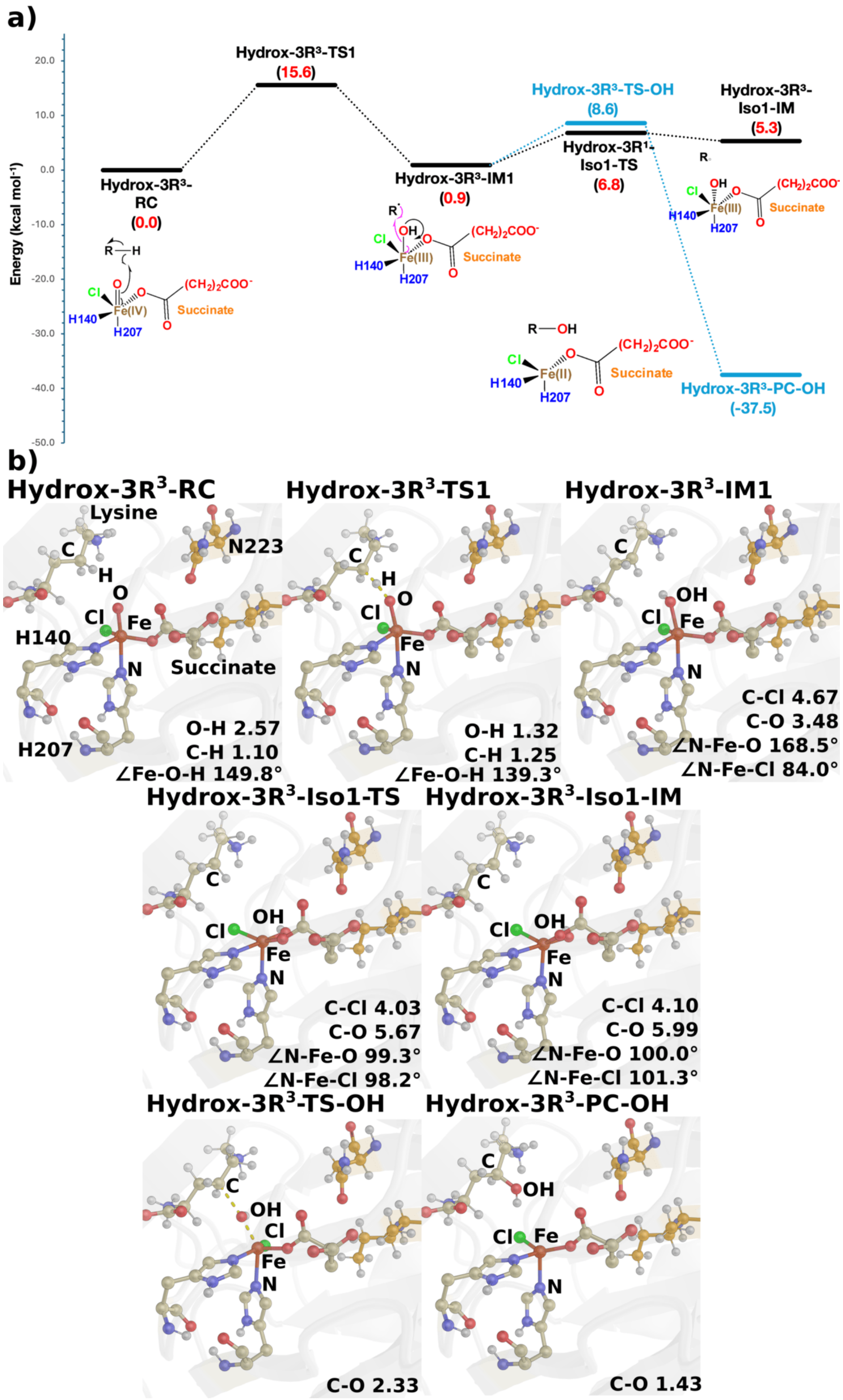

Figure S46. QM/MM reaction profile for HAT and the subsequent isomerization and rebound hydroxylation steps, starting from snapshot Hydrox-3R^3.

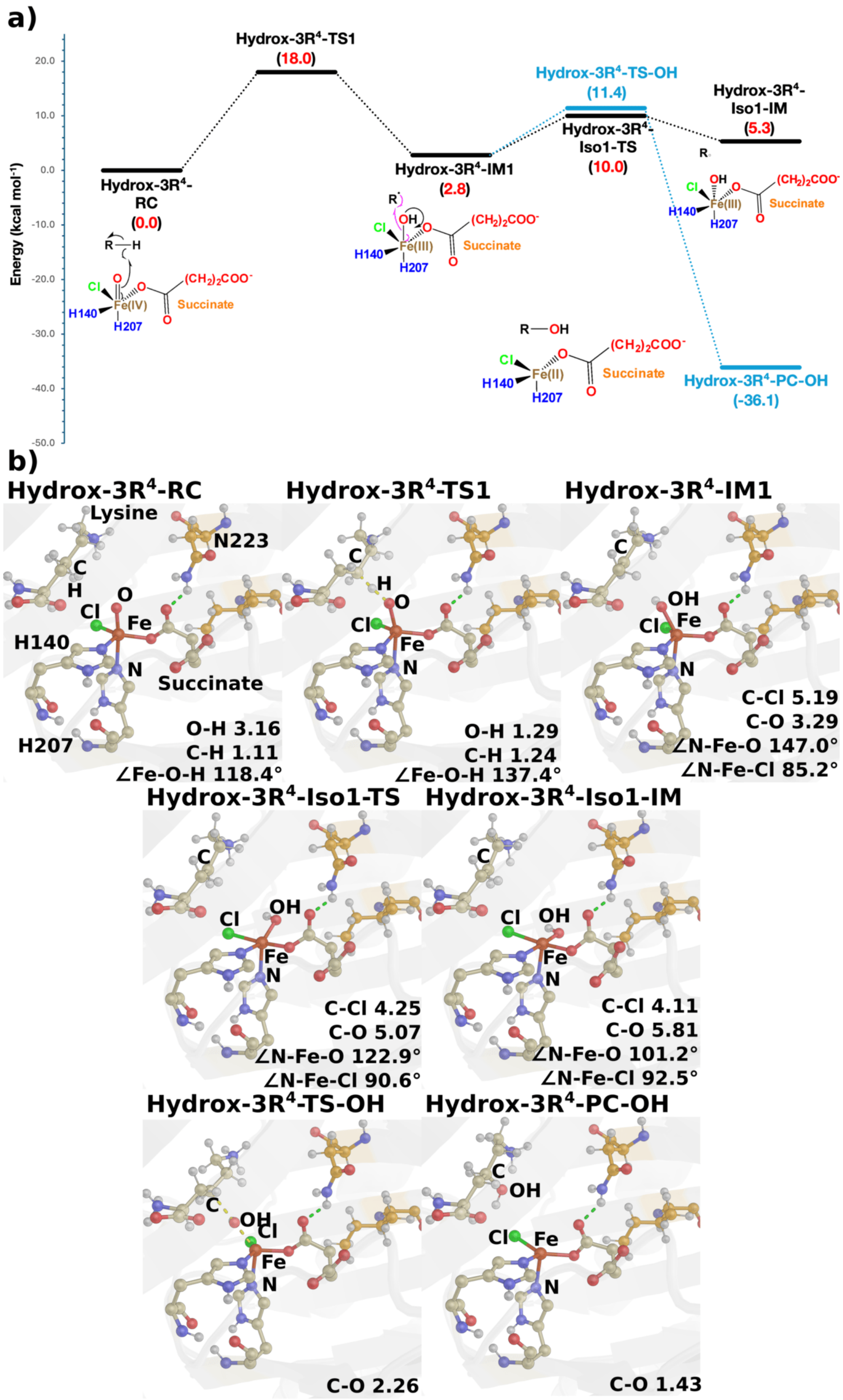

Figure S47. QM/MM reaction profile for HAT and the subsequent isomerization and rebound hydroxylation steps, starting from snapshot Hydrox-3R^4.

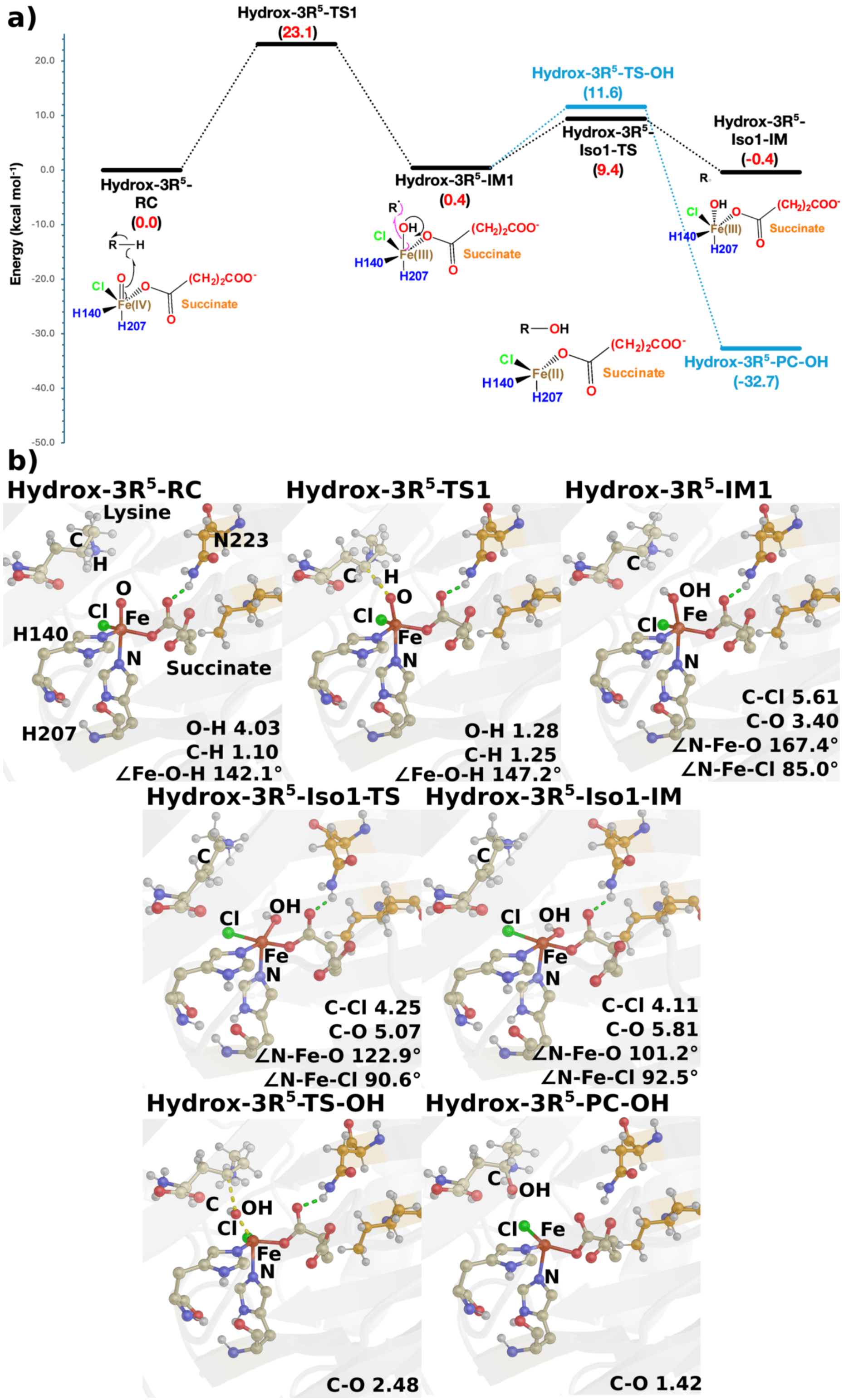

Figure S48. QM/MM reaction profile for HAT and the subsequent isomerization and rebound hydroxylation steps, starting from snapshot Hydrox-3R^5.

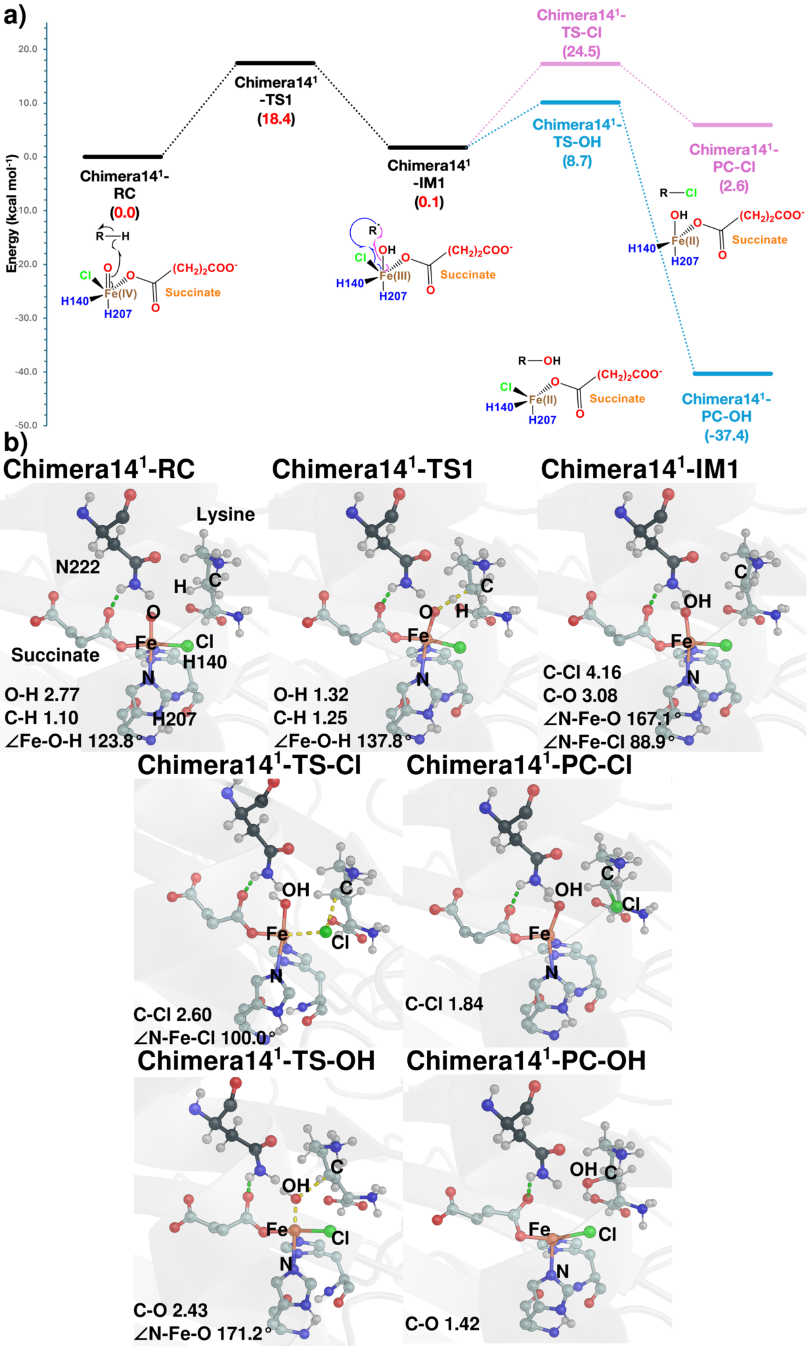

Figure S49. a) Reaction Profile of hydroxylation and chlorination reactions obtained from Chimera14¹-RC, b) QM/MM optimized structures of stationary points obtained from Chimera14¹-RC. Distances are mentioned in Å, and energies are mentioned in kcal/mol at the QM(B3)/MM level.

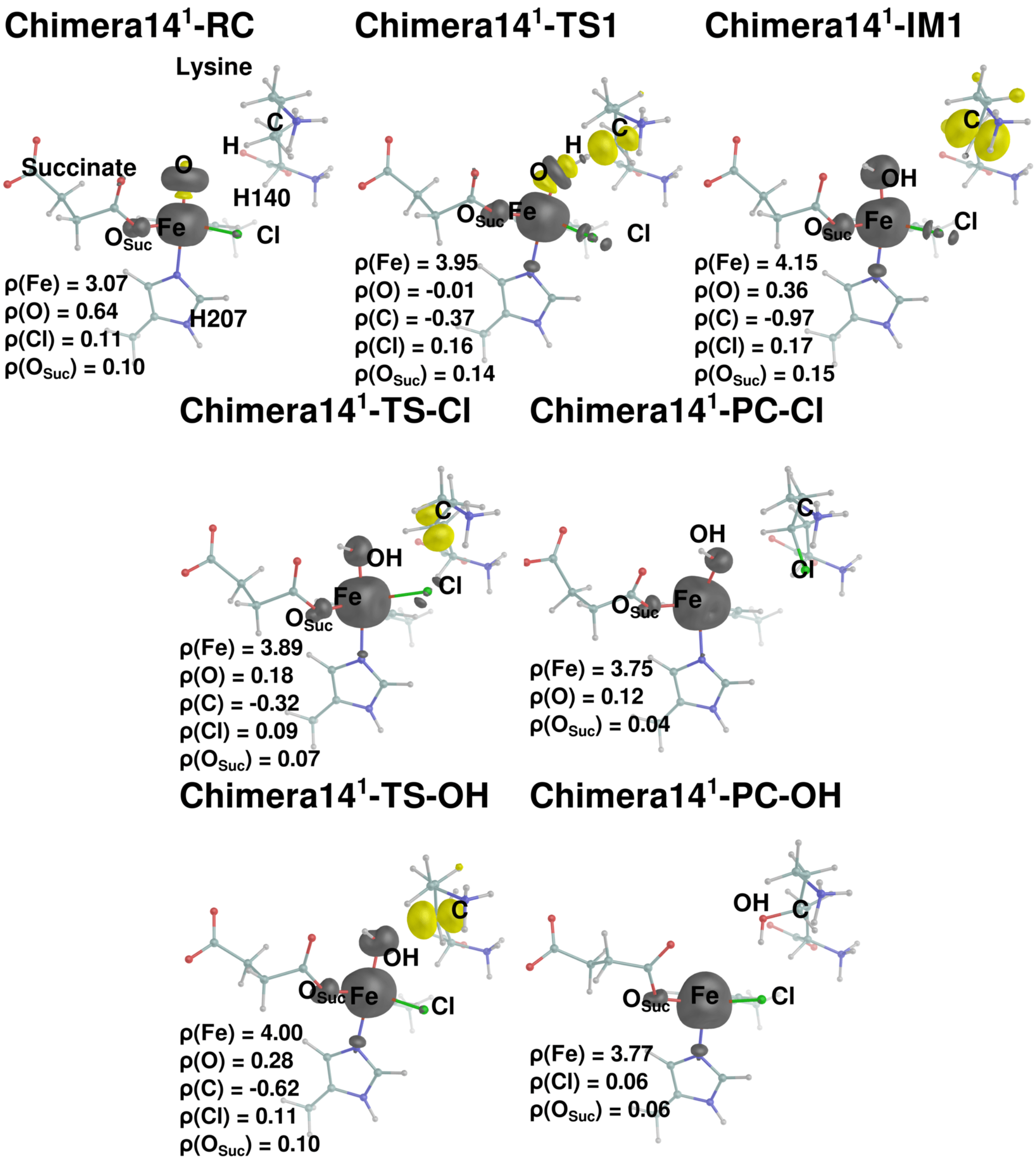

Figure S50. Spin density plots for the stationary points obtained for hydroxylation and chlorination reactions during QM/MM calculations on the Chimera14^1-RC.

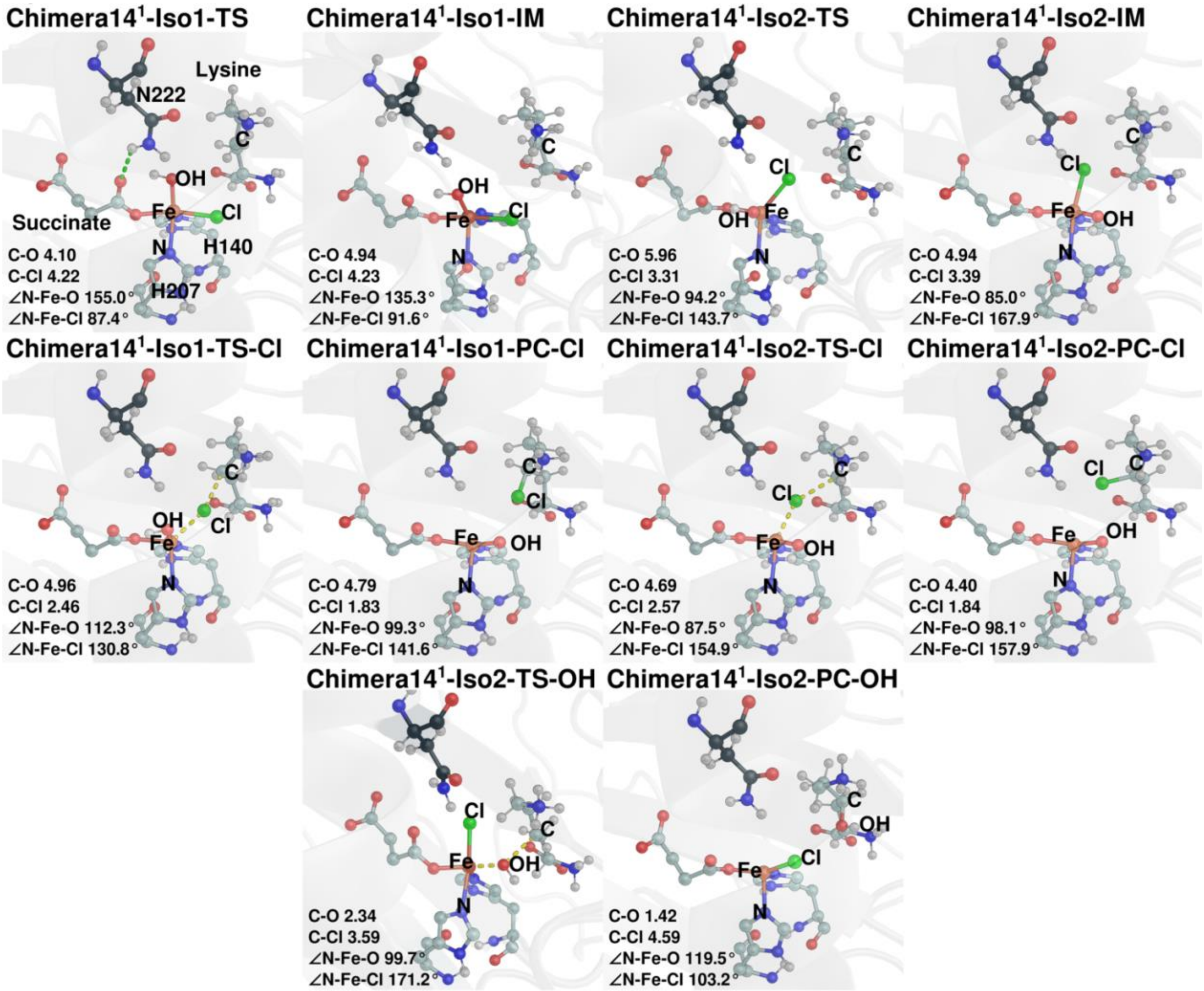

Figure S51. QM/MM optimized structures of stationary points obtained from Chimera14[1]-IM1. Distances are mentioned in Å.

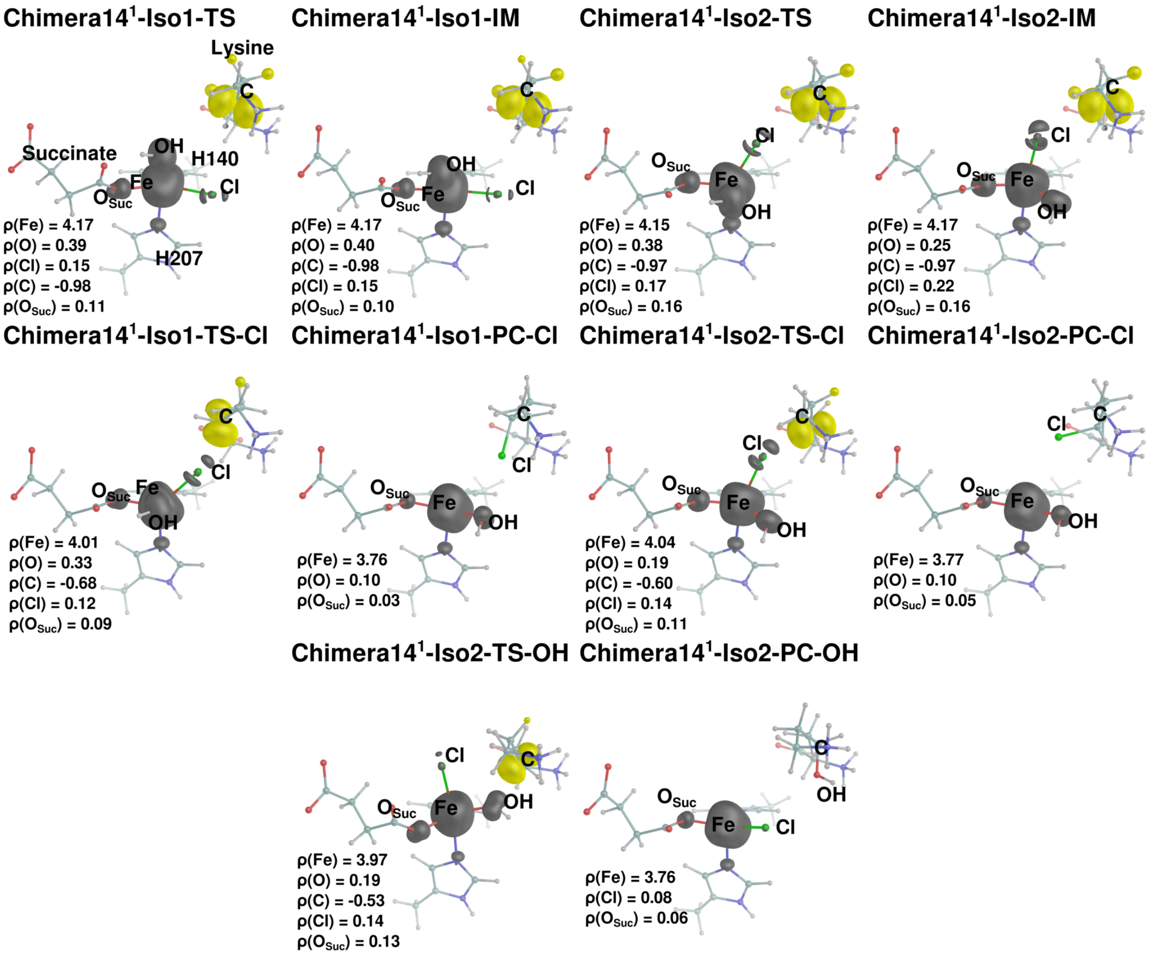

Figure S52. Spin density plots for the stationary points obtained for isomerization, hydroxylation, and chlorination reactions during QM/MM calculations on the Chimera14^{1}-RC.

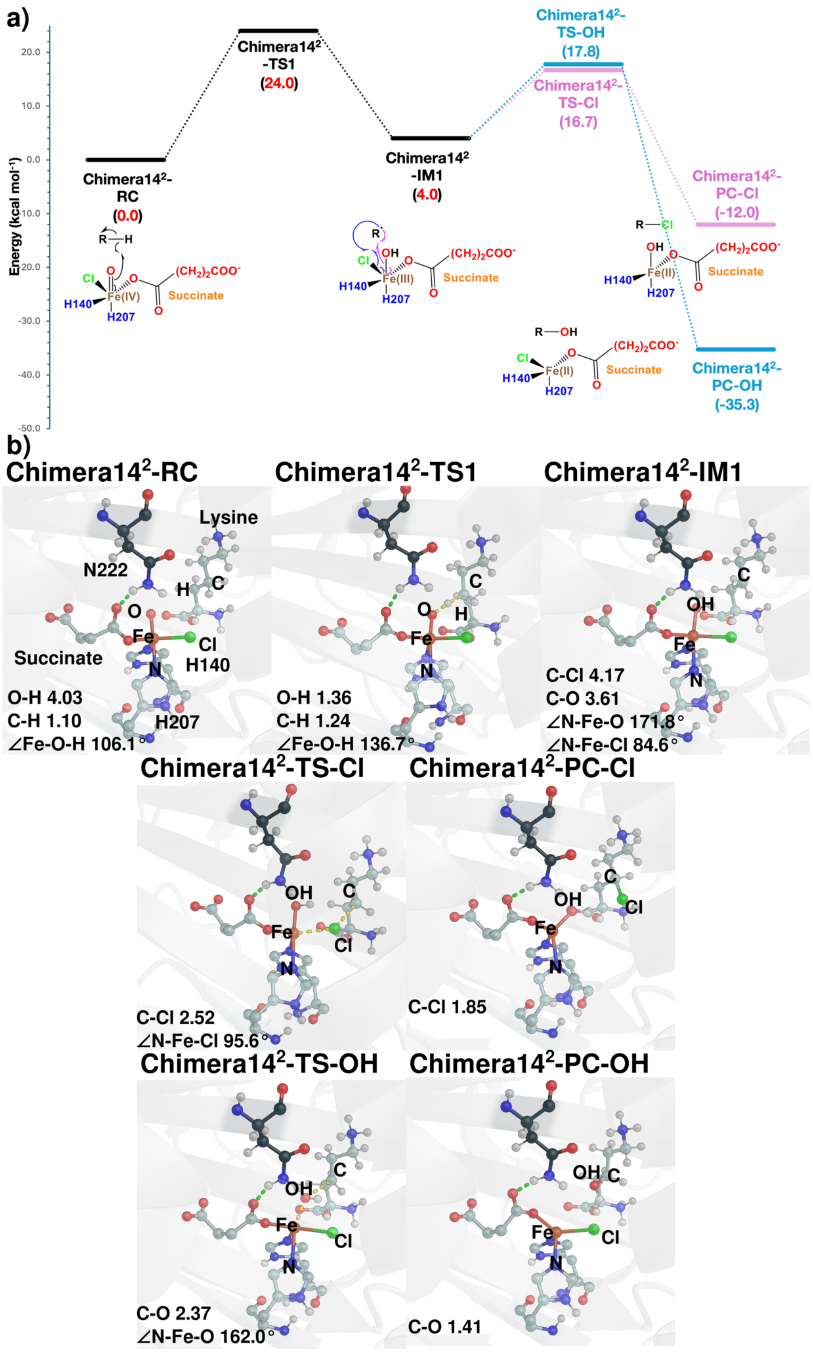

Figure S53. a) Reaction Profile of hydroxylation and chlorination reactions obtained from Chimera14^2-RC, b) QM/MM optimized structures of stationary points obtained from Chimera14^2-RC. Distances are mentioned in Å, and energies are mentioned in kcal/mol at the QM(B3)/MM level.

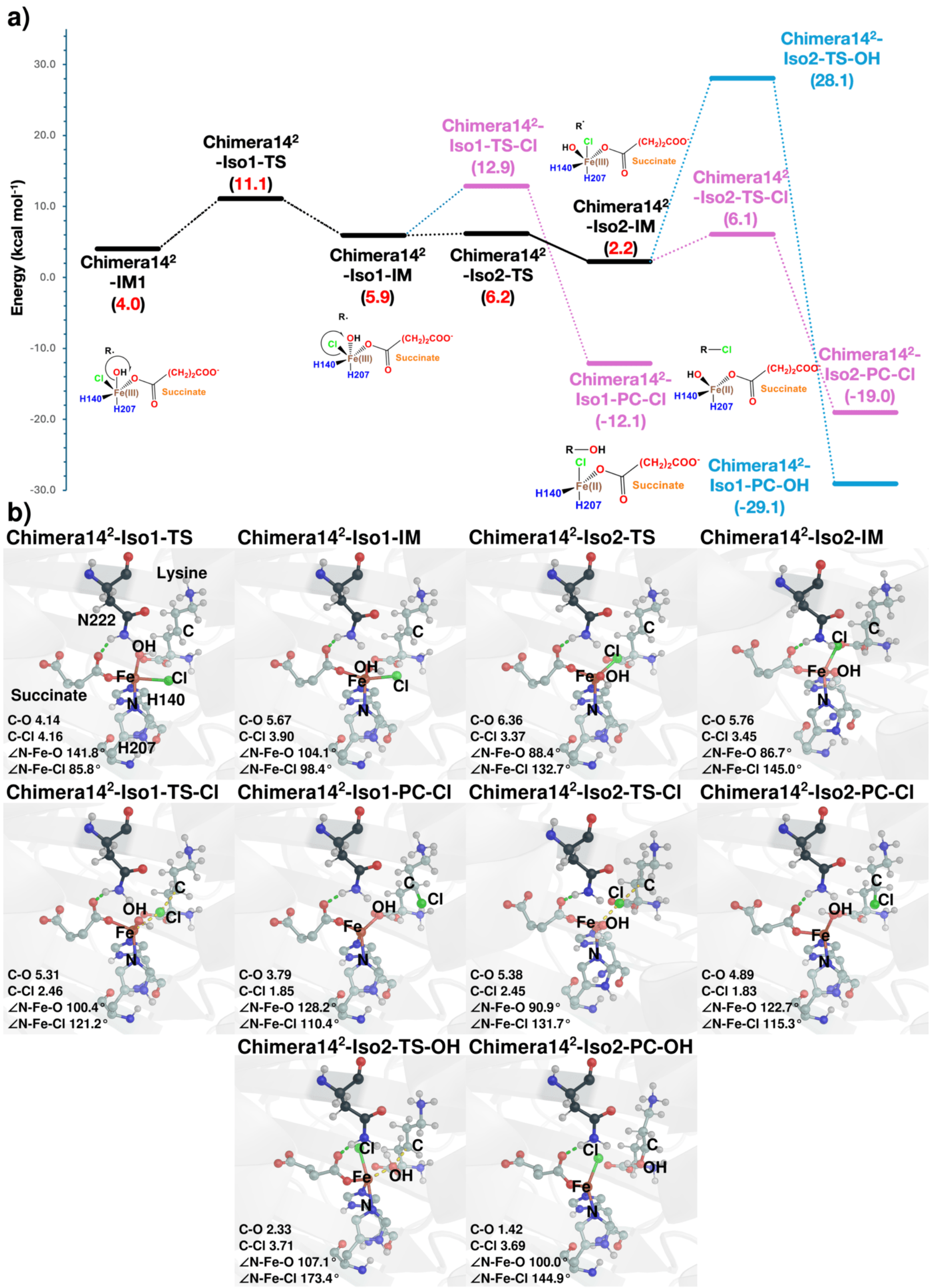

Figure S54. a) Reaction Profile of isomerization, hydroxylation, and chlorination reactions obtained from Chimera14^2-RC, b) QM/MM optimized structures of stationary points obtained from Chimera14^2-RC. Distances are mentioned in Å, and energies are mentioned in kcal/mol at the QM(B3)/MM level.

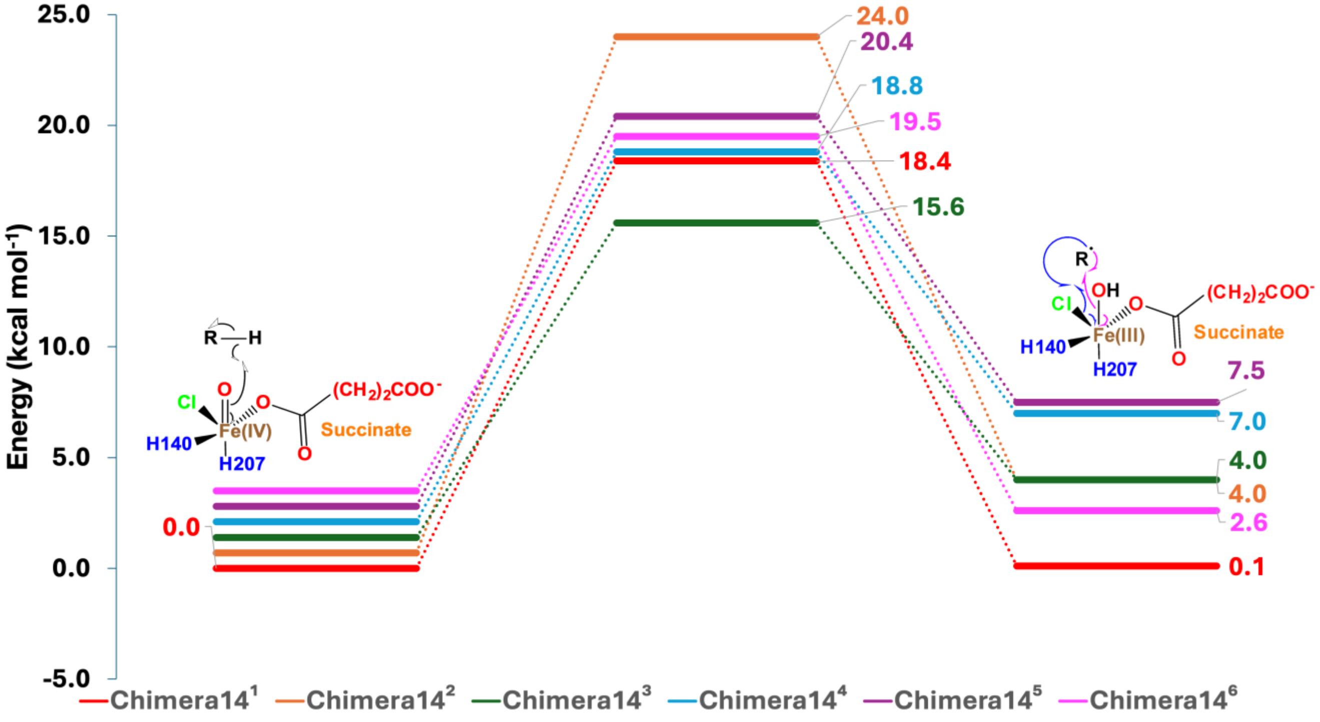

Figure S55. QM/MM reaction profiles of HAT reactions in Chimera14. The relative energies are given in kcal/mol at the QM(B3)/MM level.

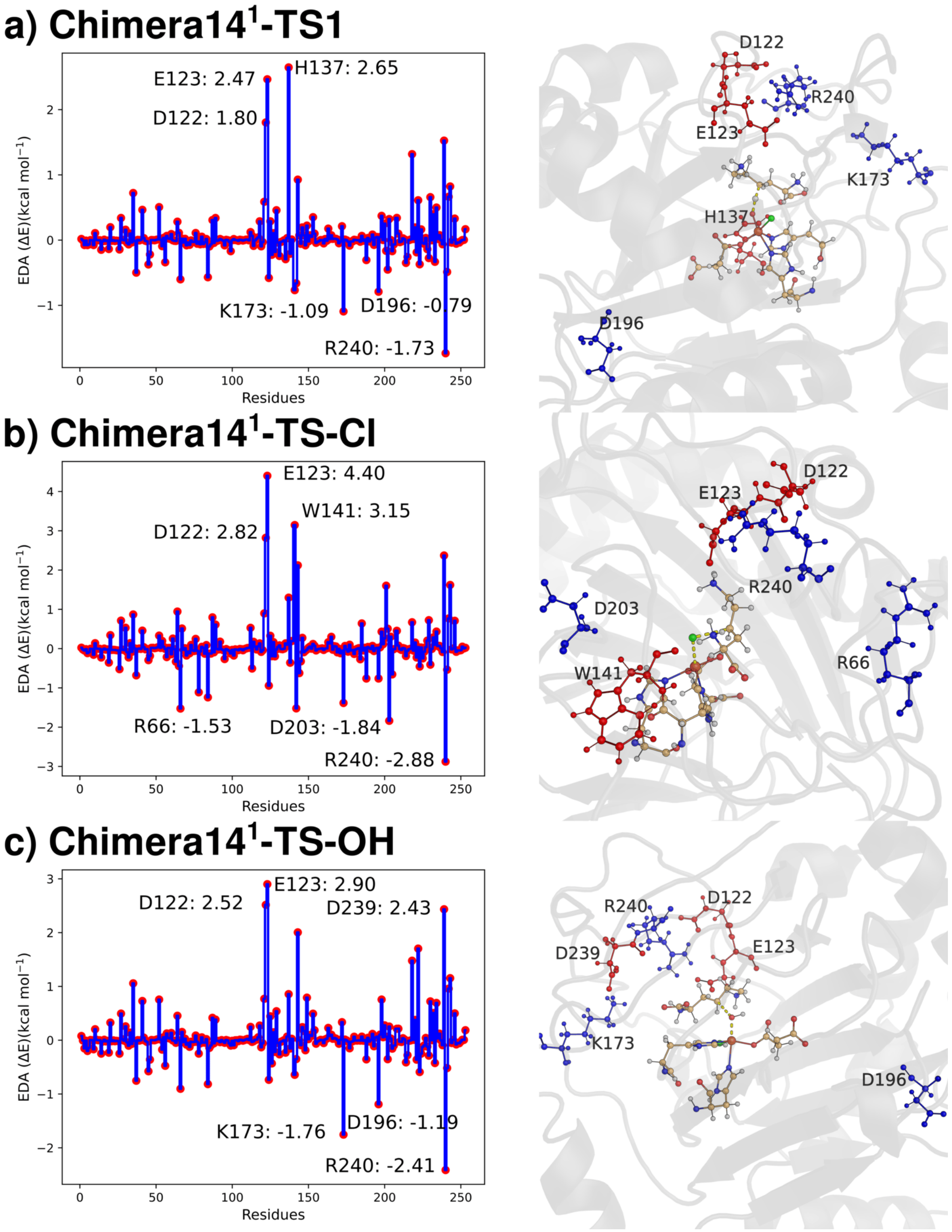

Figure S56. EDA analysis of HAT, chlorination and hydroxylation steps in Chimera14[1]-RC snapshot.

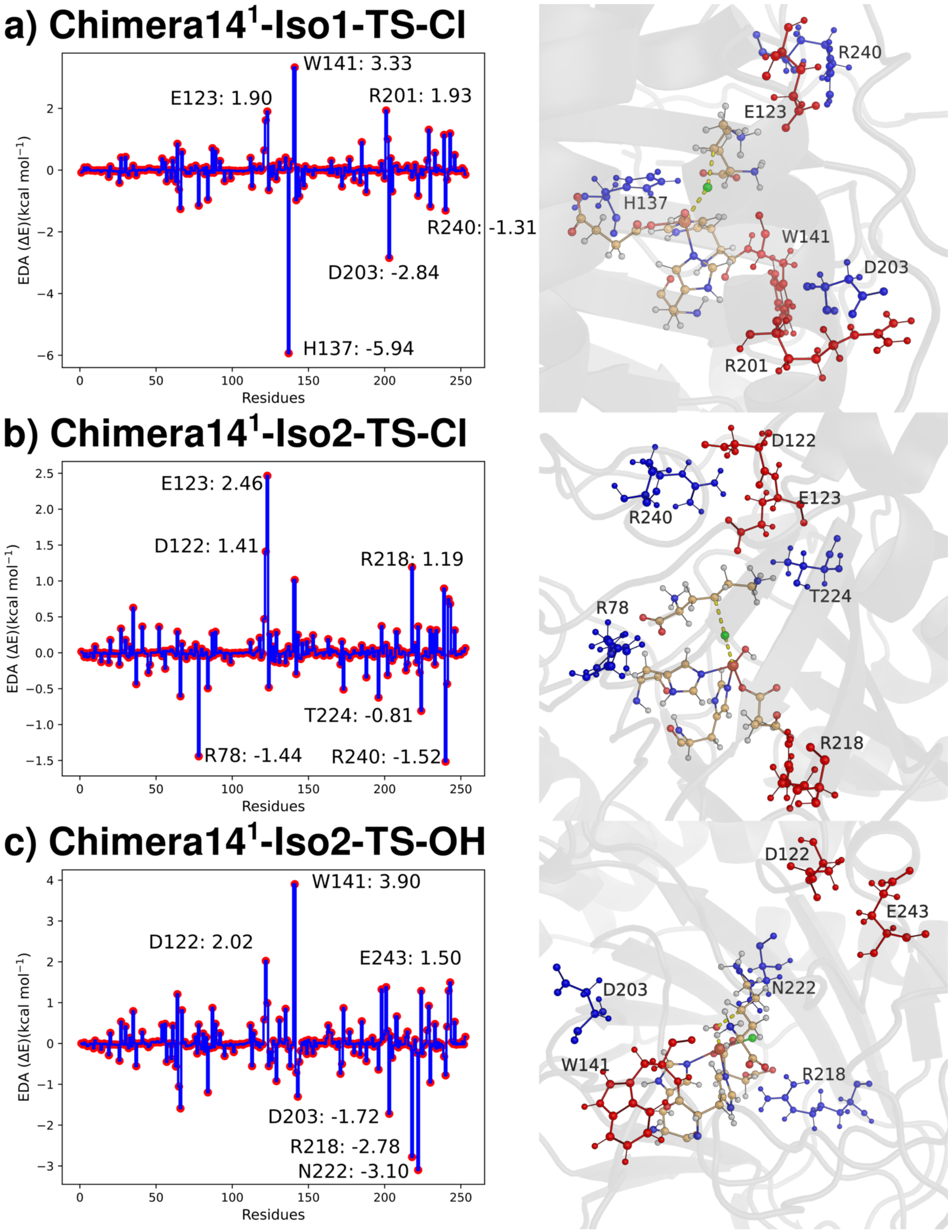

Figure S57. EDA analysis of chlorination and hydroxylation post-isomerization steps in Chimera14[1]-RC snapshot.

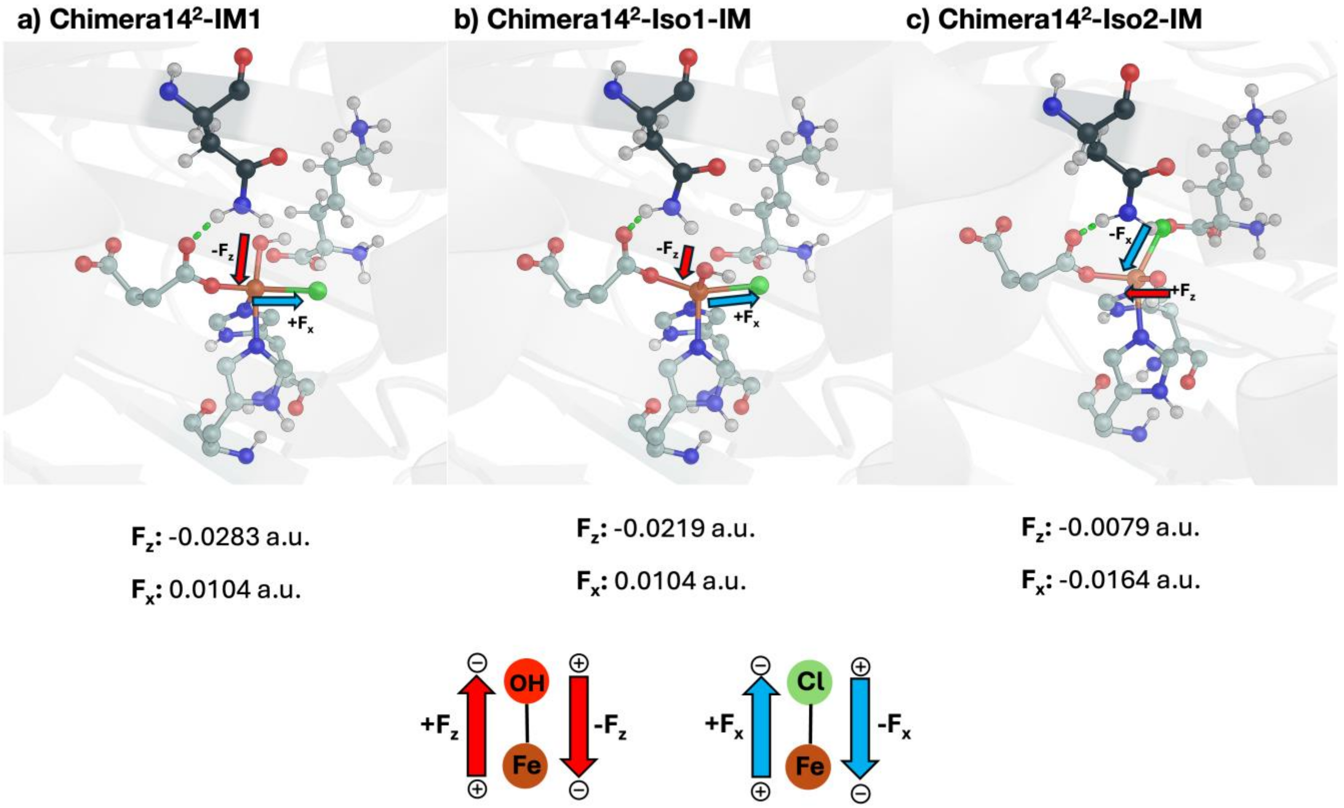

Figure S58. IEF variation along the Fe–Cl and Fe–O bonds in Cl–Fe(III)–OH isomers of Chimera14^2-RC. The IEF vectors are defined from positive to negative according to the TITAN convention. The red arrows indicate the field components, with the z-axis aligned along the Fe–O bond and the x-axis along the Fe–Cl bond (shown in blue).

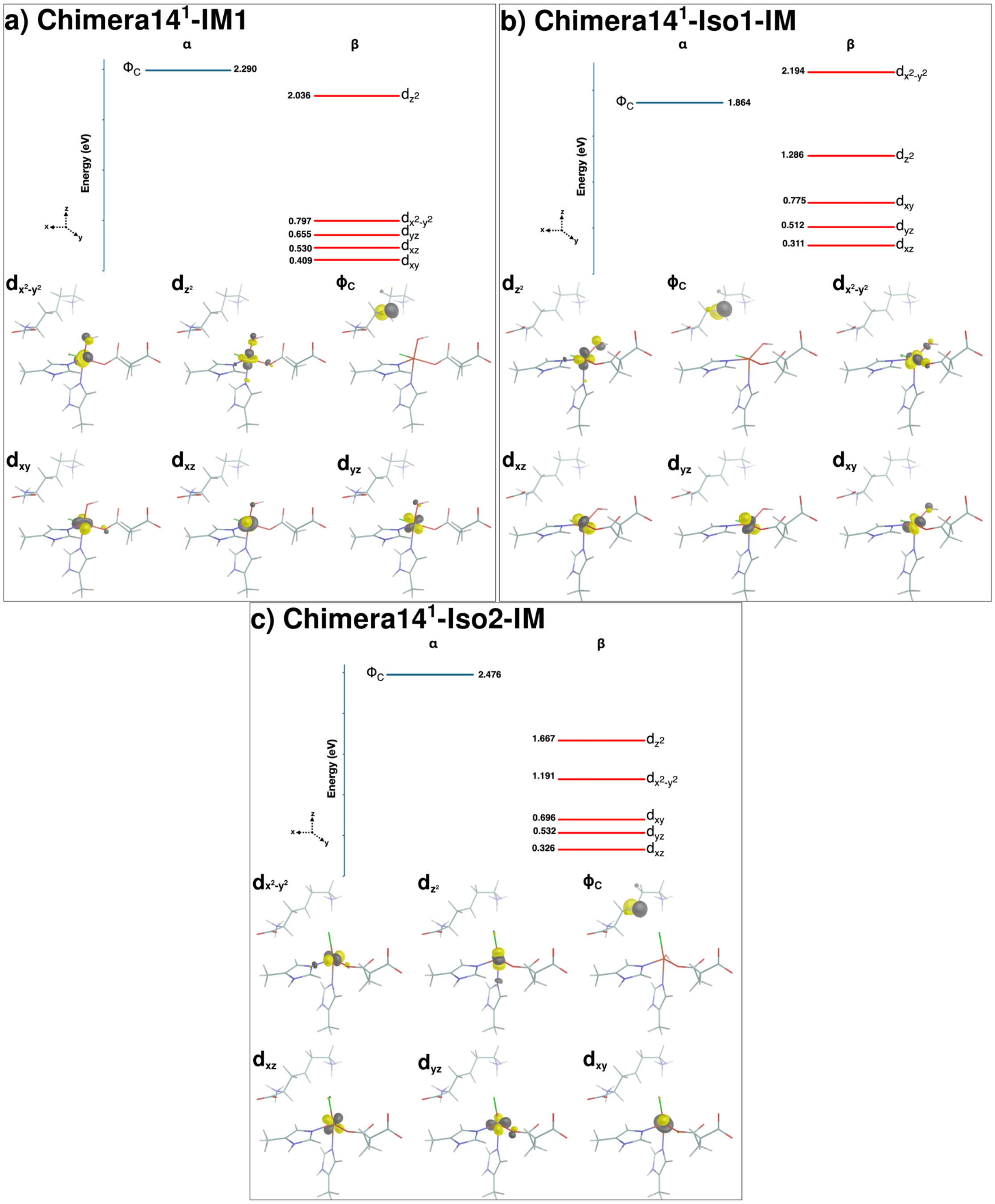

Figure S59. FMO analysis of the isomers of Cl-Fe(III)-OH intermediates obtained from Chimera14[1]-RC, a) Chimera14[1]-IM1, b) Chimera14[1]-Iso1-IM, and c) Chimera14[1]-Iso2-IM.

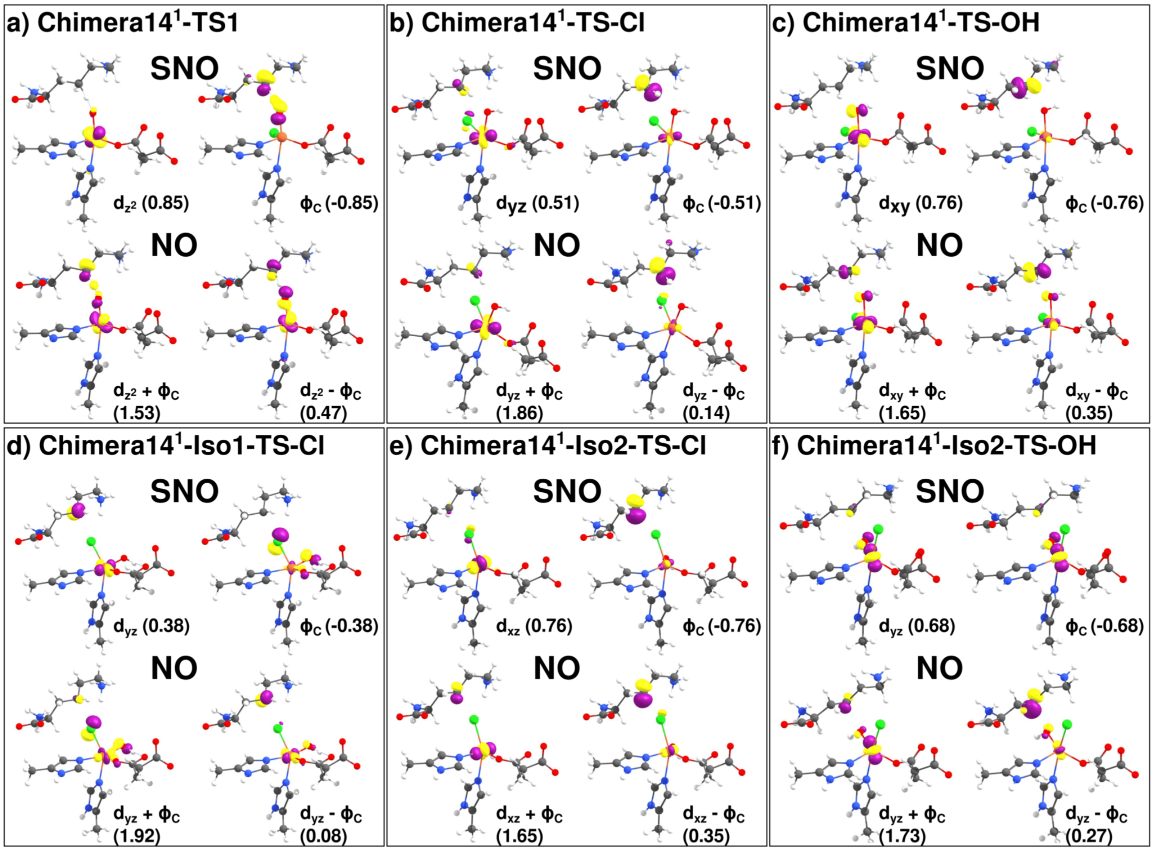

Figure S60. SNO and NO analysis of the TS states of HAT, chlorination, and hydroxylation originating from different isomers of Cl-Fe(III)-OH intermediate obtained from Chimera14^1-RC, a) Chimera14^1-TS1, b) Chimera14^1-TS-Cl, c) Chimera14^1-TS-OH, d) Chimera14^1-Iso1-TS-Cl, e) Chimera14^1-Iso2-TS-Cl, and f) Chimera14^1-Iso2-TS-OH.

Table S1. Tabulated B1, B2, and B3 energy values for the HAT reaction computed with an expanded QM region and MM environment, D3 dispersion corrections, and the def2-TZVP basis set, for the lowest-energy snapshot of BesD (BesD4).

	Reaction states	B1 (kcal/mol)	B2 (kcal/mol)	B3 (kcal/mol)
**Standard QM and MM regions**	**RC1**	0	0	0
	**TS1**	23.0	22.2	17.5
	**IM1**	2.9	2.0	1.7
**Extended QM region involving residues R77, R218 and N222**	**RC1**	0.0	0.0	0.0
	**TS1**	25.5	25.0	22.0
	**IM1**	3.7	3.6	5.5
**MM region with 12**	**RC1**	0.0	0.0	0.0
	**TS1**	21.7	21.0	16.2
	**IM1**	2.5	1.7	1.7
**Dispersion (D3) correction**	**RC1**	0.0	0.0	0.0
	**TS1**	23.0	20.6	15.9
	**IM1**	2.9	1.8	1.5
**def2-TZVP basis set**	**RC1**	-	0.0	-
	**TS1**	-	22.2	-
	**IM1**	-	1.5	-
**Extended QM region involving residue N222**	**RC1**	0.0	0.0	0.0
	**TS1**	25.1	24.9	21.7
	**IM1**	3.2	3.1	3.3
**Extended QM region involving residues R77 and R218**	**RC1**	0.0	0.0	0.0
	**TS1**	23.2	22.5	18.5
	**IM1**	3.2	2.5	3.5

4. Coordinates

BesD4-RC

QM(B1)/MM Energy = -3362.338854996667 a.u.

QM(B2)/MM Energy = -3364.468322000000 a.u.

QM(B3)/MM Energy = -3363.943585271025 a.u.

1 N	36.1706740	47.4159599	36.1364021
2 C	34.9093377	47.9218394	36.3720470
3 C	34.4129562	49.2196140	35.8171219
4 N	35.1864609	45.9755536	37.4543842
5 C	34.3063024	46.9997667	37.1976890
6 C	36.3080501	46.2560374	36.8094036
7 H	33.3130185	49.2217176	35.8540205
8 H	34.7134667	49.3378607	34.7653301
9 H	33.3140983	47.0159528	37.6380604
10 H	37.2116617	45.6504088	36.8043360
11 H	36.8851413	47.8589456	35.5494711
12 N	36.2600040	47.7045726	41.2949896
13 C	37.4726257	47.2313225	40.8241467
14 C	38.7940363	47.8941401	41.0382386
15 N	35.7982560	45.8790036	40.1721970
16 C	37.1596132	46.0837409	40.1376688
17 C	35.2835723	46.8752534	40.8761887
18 H	38.8878782	48.2799610	42.0654289
19 H	39.5972117	47.1536776	40.9177252
20 H	37.8212034	45.3838145	39.6373464
21 H	34.2250320	47.0115929	41.0837772
22 H	36.1095767	48.5114903	41.9098108
23 Cl	32.8732336	44.9588705	39.9789843
24 Fe	34.8581238	44.4748566	38.8951694
25 O	34.1385386	43.4579057	37.8720694
26 O	36.0685983	41.3722178	38.5855294
27 C	36.8077365	42.2568690	38.9778596
28 O	36.4521481	43.5080495	39.1781996
29 C	38.2736577	42.0161574	39.2859776
30 C	39.1537455	42.2370069	38.0419884
31 C	39.0396780	41.0647655	37.0434673
32 O	39.7129093	40.0373744	37.3300943
33 O	38.2915738	41.1940408	36.0509263
34 H	38.5857839	42.6837195	40.1021978
35 H	38.3915054	40.9780404	39.6219081
36 H	40.1994883	42.3016511	38.3803454
37 H	38.8848915	43.1864218	37.5471501
38 N	29.4399762	46.8338726	35.5848076
39 C	30.7747874	46.1432137	35.6255095
40 C	31.6264895	46.6061625	34.4054870
41 O	31.1649498	47.5435420	33.7040852
42 C	30.6189220	44.6233779	35.7635495
43 C	30.3510216	44.1556397	37.2052710
44 C	29.8748871	42.7003342	37.3560752
45 C	28.4261598	42.3931888	36.9639772
46 N	27.4560062	43.2557667	37.7175353
47 O	32.7140431	46.0190214	34.2222604
48 H	31.2853718	46.5384662	36.5194186
49 H	29.8367453	44.2738970	35.0685215
50 H	31.5607851	44.1788390	35.4109498
51 H	29.6304181	44.8303657	37.7007508
52 H	31.2825673	44.2673477	37.7819489
53 H	30.5210920	42.0311378	36.7631112
54 H	30.0252698	42.3933672	38.4056345
55 H	28.2293784	42.5439916	35.8928252
56 H	28.1797502	41.3471612	37.1985974
57 H	27.7282929	43.3434883	38.7138086
58 H	27.4176136	44.2347197	37.3626974

59 H 26.4710971 42.8984242 37.7304102
60 H 28.7447711 46.4894473 36.2835668
61 H 29.5661231 47.8524239 35.7228676
62 H 28.9715809 46.7662165 34.6557501
63 H 34.7882750 50.1075313 36.3258516
64 H 38.9861833 48.7457434 40.3856143

BesD4-TS1
QM(B1)/MM Energy = -3362.302230795213 a.u.
QM(B2)/MM Energy = -3364.433013000000 a.u.
QM(B3)/MM Energy = -3363.915776396148 a.u.

1 N 36.0547579 47.5429130 36.0777695
2 C 34.8020731 48.0851659 36.2710091
3 C 34.3540065 49.4062765 35.7454211
4 N 34.9676833 46.1160345 37.3211294
5 C 34.1357109 47.1711303 37.0495964
6 C 36.1253978 46.3676225 36.7316792
7 H 33.2551713 49.4366489 35.7896956
8 H 34.6582228 49.5458793 34.6977503
9 H 33.1284221 47.2199187 37.4489302
10 H 37.0044834 45.7283217 36.7518785
11 H 36.8038484 47.9632377 35.5184307
12 N 36.1271045 47.6092323 41.2449868
13 C 37.3090272 47.1301397 40.7067656
14 C 38.6507620 47.7501978 40.9498475
15 N 35.5684230 45.8554548 40.0403266
16 C 36.9334497 46.0275143 39.9701587
17 C 35.1140332 46.8238614 40.8182625
18 H 38.7434787 48.0901783 41.9940769
19 H 39.4400969 46.9974825 40.8073686
20 H 37.5604507 45.3398933 39.4082858
21 H 34.0693933 46.9719044 41.0829541
22 H 36.0261892 48.3924514 41.9000079
23 Cl 32.6609270 44.8138006 40.1649584
24 Fe 34.3791431 44.5615852 38.5885652
25 O 33.2170233 43.9670722 37.4083441
26 O 35.5507900 41.3894728 37.8056304
27 C 36.2527801 42.1935962 38.3983658
28 O 35.9220056 43.4540081 38.5762169
29 C 37.6026611 41.8295327 38.9955433
30 C 38.7835785 42.1020765 38.0368157
31 C 38.9301741 40.9815988 36.9881569
32 O 39.7454279 40.0624183 37.2644984
33 O 38.2140572 41.0343929 35.9631486
34 H 37.7384137 42.4060796 39.9228484
35 H 37.5904403 40.7590959 39.2473322
36 H 39.7086123 42.1491708 38.6307305
37 H 38.6441808 43.0750920 37.5336769
38 N 29.5193025 46.8267944 35.5911248
39 C 30.8314185 46.0980379 35.5670044
40 C 31.6700651 46.5942359 34.3531244
41 O 31.2167350 47.5664621 33.6964922
42 C 30.6359432 44.5744489 35.6383988
43 C 30.6968113 43.9922750 37.0476453
44 C 30.1695404 42.5755566 37.2541731
45 C 28.6775606 42.3093654 36.9713408
46 N 27.7454914 43.1729046 37.7698869
47 O 32.7403547 45.9897398 34.1352888
48 H 31.3779444 46.4321143 36.4614863
49 H 29.6796354 44.3129627 35.1520434
50 H 31.4196281 44.1063774 35.0250808
51 H 30.3450737 44.6910451 37.8255325
52 H 31.9303283 43.9485548 37.3213969
53 H 30.7221626 41.8807002 36.5985341
54 H 30.4057172 42.2632103 38.2856107

55 H 28.4188614 42.4589575 35.9130401
56 H 28.4364658 41.2652047 37.2198931
57 H 28.0208746 43.2328142 38.7671552
58 H 27.7157553 44.1631839 37.4442128
59 H 26.7513653 42.8357000 37.7718766
60 H 28.8494836 46.4867109 36.3160629
61 H 29.6777670 47.8400729 35.7392742
62 H 29.0122487 46.7806421 34.6808645
63 H 34.7591635 50.2627915 36.2842133
64 H 38.8806878 48.6264904 40.3437843

BesD4-IM1

QM(B1)/MM Energy = -3362.334212720499 a.u.

QM(B2)/MM Energy = -3364.465168000000 a.u.

QM(B3)/MM Energy = -3363.940838932991 a.u.

1 N 36.1365928 47.5257737 36.0740449
2 C 34.8738268 48.0422048 36.2788760
3 C 34.4026706 49.3554282 35.7489946
4 N 35.0903469 46.0711735 37.3204894
5 C 34.2321588 47.1104329 37.0581189
6 C 36.2378064 46.3507845 36.7214433
7 H 33.3032430 49.3688025 35.7887129
8 H 34.7071726 49.4937585 34.7010914
9 H 33.2218316 47.1266118 37.4535713
10 H 37.1317376 45.7318091 36.7333003
11 H 36.8706907 47.9636648 35.5084231
12 N 36.1741643 47.6553514 41.2616750
13 C 37.3690803 47.1799090 40.7487794
14 C 38.7030593 47.8151516 40.9840208
15 N 35.6557010 45.8738771 40.0850048
16 C 37.0189065 46.0600124 40.0293643
17 C 35.1763351 46.8510919 40.8361748
18 H 38.7940915 48.1708867 42.0227553
19 H 39.4969255 47.0660189 40.8505554
20 H 37.6598449 45.3674407 39.4901616
21 H 34.1256031 46.9930692 41.0807001
22 H 36.0534887 48.4472353 41.9025848
23 Cl 32.7368236 44.7518728 40.0956539
24 Fe 34.5843880 44.5324599 38.6430146
25 O 33.4419335 43.7477484 37.4015432
26 O 35.6832181 41.4011062 37.9523204
27 C 36.4278095 42.1715889 38.5384532
28 O 36.1347313 43.4324957 38.7771875
29 C 37.7954219 41.7719395 39.0596593
30 C 38.9179065 42.0768063 38.0438729
31 C 39.0072438 40.9686808 36.9785189
32 O 39.7707321 40.0046172 37.2540756
33 O 38.3013992 41.0653751 35.9503582
34 H 37.9824692 42.3136821 39.9986804
35 H 37.7899977 40.6933924 39.2708352
36 H 39.8751268 42.1177697 38.5848837
37 H 38.7427250 43.0573151 37.5681600
38 N 29.4622122 46.7465781 35.5270865
39 C 30.8182556 46.1030625 35.5788803
40 C 31.6784836 46.5991356 34.3806592
41 O 31.2122415 47.5449664 33.6920194
42 C 30.7561420 44.5786670 35.7014107
43 C 30.1913081 44.0780089 36.9925203
44 C 29.7847942 42.6460869 37.2032390
45 C 28.2959313 42.3173481 36.9867479
46 N 27.4180834 43.2489572 37.7584290
47 O 32.7712787 46.0230536 34.1976549
48 H 31.2918317 46.5013335 36.4905318
49 H 30.2513946 44.1278886 34.8270058
50 H 31.8154479 44.2574041 35.6454893

51 H 30.3755165 44.6874434 37.8847315

52 H 32.7312476 43.2421081 37.8216240

53 H 30.3448078 41.9794894 36.5222595

54 H 30.0579870 42.3405064 38.2287493

55 H 28.0090232 42.3957280 35.9299486

56 H 28.0731828 41.2930530 37.3200408

57 H 27.7297845 43.3380833 38.7429075

58 H 27.4434344 44.2220889 37.3835741

59 H 26.4148114 42.9506905 37.8041655

60 H 28.7834848 46.3832993 36.2339733

61 H 29.5597850 47.7681481 35.6683972

62 H 28.9915743 46.6715098 34.6006629

63 H 34.7920142 50.2232025 36.2813914

64 H 38.9208607 48.6831369 40.3617418

BesD4-TS-Cl

QM(B1)/MM Energy = -3362.306814272310 a.u.

QM(B2)/MM Energy = -3364.435738000000 a.u.

QM(B3)/MM Energy = -3363.916020081878 a.u.

1 N 36.1153958 47.4604349 36.0531232

2 C 34.8662834 48.0053499 36.2668382

3 C 34.4098290 49.3296942 35.7500441

4 N 35.0001014 45.9700941 37.2069330

5 C 34.1900962 47.0584920 36.9965617

6 C 36.1651720 46.2484035 36.6409691

7 H 33.3099573 49.3464744 35.7805129

8 H 34.7194607 49.4736952 34.7039508

9 H 33.1864200 47.1020921 37.4040513

10 H 37.0403391 45.6014937 36.6423360

11 H 36.8733457 47.9048779 35.5257586

12 N 35.9122685 47.6609528 41.1308940

13 C 37.1142979 47.1216994 40.7021762

14 C 38.4710461 47.7046469 40.9669117

15 N 35.3916838 45.8024386 40.0558424

16 C 36.7633150 45.9618115 40.0503767

17 C 34.9127716 46.8480418 40.7121863

18 H 38.5554302 48.0435901 42.0130518

19 H 39.2285834 46.9160642 40.8455313

20 H 37.3998536 45.2173382 39.5823641

21 H 33.8595248 47.0593594 40.8870763

22 H 35.7979174 48.4791013 41.7392976

23 Cl 32.1325295 44.8926070 39.0195257

24 Fe 34.5394366 44.3485352 38.4793783

25 O 33.7357487 42.9053910 37.6408888

26 O 35.9528924 41.3100580 38.2517805

27 C 36.6767038 42.2227920 38.6749265

28 O 36.3286719 43.4637552 38.7363663

29 C 38.0935559 41.9360078 39.1404580

30 C 39.1064972 42.1766396 38.0058484

31 C 39.0540109 41.0386007 36.9692490

32 O 39.7334105 40.0125963 37.2518879

33 O 38.3348386 41.1783944 35.9561440

34 H 38.3256160 42.5834083 39.9999785

35 H 38.1642052 40.8891828 39.4642821

36 H 40.1171446 42.2059166 38.4421194

37 H 38.9006775 43.1470299 37.5231287

38 N 29.4666211 46.8356118 35.6557213

39 C 30.8641794 46.2822727 35.7822077

40 C 31.6532911 46.6288265 34.4783785

41 O 31.1772159 47.5506828 33.7652474

42 C 30.8974525 44.8023233 36.1637262

43 C 30.3899036 44.4483811 37.5343839

44 C 30.0759291 43.0008930 37.8172057

45 C 28.7093794 42.5154823 37.2878632

46 N 27.5777327 43.2943048 37.8906385

47 O	32.6962799	45.9848189	34.2472097
48 H	31.3214787	46.8660542	36.5964588
49 H	30.3464956	44.2063301	35.4061999
50 H	31.9434075	44.4583380	36.0811302
51 H	29.7438396	45.1786935	38.0301175
52 H	34.3399499	42.1383639	37.7302508
53 H	30.8709738	42.3674189	37.3911256
54 H	30.1073079	42.8214502	38.9025058
55 H	28.6136907	42.5958065	36.1946649
56 H	28.5556266	41.4599173	37.5568277
57 H	27.7148684	43.4143263	38.9107851
58 H	27.5011867	44.2640205	37.5151988
59 H	26.6285288	42.8574502	37.7981526
60 H	28.7826430	46.5052610	36.3719368
61 H	29.5128378	47.8685203	35.7167084
62 H	29.0203692	46.6732826	34.7280033
63 H	34.7933080	50.1991102	36.2840167
64 H	38.7650782	48.5667422	40.3682432

BesD4-PC-Cl

QM(B1)/MM Energy = -3362.335499376142 a.u.

QM(B2)/MM Energy = -3364.466944000000 a.u.

QM(B3)/MM Energy = -3363.934162372134 a.u.

1 N	36.2442768	47.6429303	35.9228361
2 C	34.9743426	48.1073654	36.1883748
3 C	34.4471422	49.4204515	35.7170913
4 N	35.2804465	46.0928747	37.1290517
5 C	34.3909967	47.1196456	36.9468582
6 C	36.3957501	46.4391637	36.5118502
7 H	33.3499276	49.4046810	35.7989482
8 H	34.7091826	49.5967444	34.6627997
9 H	33.3932934	47.0951811	37.3742175
10 H	37.3060918	45.8431693	36.4755769
11 H	36.9378538	48.1180302	35.3408215
12 N	36.0578187	47.5523547	41.1052610
13 C	37.2910762	47.0985239	40.6717232
14 C	38.6070045	47.7531332	40.9609453
15 N	35.6563308	45.7018601	39.9585272
16 C	37.0153832	45.9452453	39.9737137
17 C	35.1119696	46.6927583	40.6477810
18 H	38.6545823	48.1030682	42.0051607
19 H	39.4083995	47.0068639	40.8570913
20 H	37.7061060	45.2797700	39.4623499
21 H	34.0472105	46.8410433	40.8244194
22 H	35.8931452	48.3550414	41.7226972
23 Cl	31.3776284	44.6204781	38.8950738
24 Fe	35.0152293	44.3058668	38.2406613
25 O	33.5478667	43.5556862	37.3047983
26 O	35.6694325	42.1247270	39.0258852
27 C	36.8238344	42.5338797	38.7281232
28 O	36.9809511	43.6363623	38.1043174
29 C	38.0511319	41.7950101	39.2045422
30 C	39.2507969	41.9029842	38.2615948
31 C	39.1254013	40.8719157	37.1288302
32 O	39.8373917	39.8351210	37.2505897
33 O	38.3051152	41.0807902	36.2075755
34 H	38.2979790	42.2349051	40.1884776
35 H	37.7841977	40.7454443	39.3944745
36 H	40.1799381	41.7071750	38.8158781
37 H	39.2813780	42.9249893	37.8559191
38 N	29.4610553	46.7670202	35.7094337
39 C	30.8362506	46.1480866	35.8417706
40 C	31.6510923	46.5282854	34.5545627
41 O	31.1856829	47.4809206	33.8702959
42 C	30.8064352	44.6513966	36.1628433

43 C 30.2044083 44.2552548 37.5197079
44 C 29.7783753 42.7810130 37.6223026
45 C 28.3802531 42.4761082 37.0748581
46 N 27.3355282 43.3015466 37.7692653
47 O 32.6858529 45.8834929 34.3070905
48 H 31.3102990 46.6842984 36.6801459
49 H 30.2583887 44.1085884 35.3706010
50 H 31.8515863 44.2857884 36.1574014
51 H 29.3638272 44.8982224 37.7971589
52 H 33.2739568 42.7375997 37.7433447
53 H 30.5083861 42.1449808 37.0971684
54 H 29.8099135 42.4747605 38.6792851
55 H 28.2851006 42.6616739 35.9957531
56 H 28.1343670 41.4186323 37.2495989
57 H 27.5462207 43.3971990 38.7800150
58 H 27.2729988 44.2793607 37.4137323
59 H 26.3651186 42.9062245 37.7371009
60 H 28.7409848 46.4557305 36.3964990
61 H 29.5501071 47.7958049 35.7901709
62 H 29.0301243 46.6431903 34.7688145
63 H 34.8386902 50.2865365 36.2506212
64 H 38.8607552 48.6241061 40.3567235

BesD4-TS-OH

QM(B1)/MM Energy = -3362.319237094735 a.u.

QM(B2)/MM Energy = -3364.447246000000 a.u.

QM(B3)/MM Energy = -3363.927452413514 a.u.

1 N 35.9498053 47.5487234 36.0459851
2 C 34.7224858 48.1454936 36.2408736
3 C 34.3224740 49.4857273 35.7313394
4 N 34.7774107 46.1446631 37.2436882
5 C 34.0078330 47.2503346 36.9962403
6 C 35.9531093 46.3552529 36.6705839
7 H 33.2249565 49.5443022 35.7743573
8 H 34.6343174 49.6328616 34.6869967
9 H 33.0042346 47.3473567 37.3932363
10 H 36.8004249 45.6742182 36.6963968
11 H 36.7306538 47.9523544 35.5173421
12 N 36.0696808 47.6119770 41.2169067
13 C 37.2502347 47.1293908 40.6781565
14 C 38.5974884 47.7321083 40.9351252
15 N 35.5051572 45.8703920 39.9926195
16 C 36.8700878 46.0360818 39.9293424
17 C 35.0541227 46.8339015 40.7775451
18 H 38.6876940 48.0620906 41.9829572
19 H 39.3773723 46.9697115 40.7902847
20 H 37.4917845 45.3460429 39.3642635
21 H 34.0094109 46.9847321 41.0419829
22 H 35.9735817 48.3903123 41.8783287
23 Cl 32.5231820 44.7841990 40.1046060
24 Fe 34.2511182 44.5920908 38.5333710
25 O 33.0628727 43.6884199 37.2790512
26 O 35.1307049 41.5396911 37.8139350
27 C 36.0153649 42.2364360 38.3229927
28 O 35.9043385 43.5187397 38.4815745
29 C 37.3020288 41.6461466 38.8791952
30 C 38.5757551 41.9728159 38.0672612
31 C 38.8244777 40.9232612 36.9717897
32 O 39.7425764 40.0889494 37.1879904
33 O 38.0743743 40.9352764 35.9670953
34 H 37.4210299 42.0709732 39.8883219
35 H 37.1804109 40.5566530 38.9728162
36 H 39.4412535 41.9851016 38.7452653
37 H 38.4818431 42.9759263 37.6166656
38 N 29.5217964 46.7429270 35.5713521

39 C 30.8769959 46.0960146 35.5718816

40 C 31.6883054 46.6114562 34.3450492

41 O 31.2135980 47.5821458 33.7038859

42 C 30.8380499 44.5660022 35.6880031

43 C 30.7818287 44.0114870 37.0788137

44 C 30.2584962 42.6293545 37.3528464

45 C 28.7735868 42.3334778 37.0332148

46 N 27.8030508 43.1681609 37.8131365

47 O 32.7625710 46.0215541 34.1034780

48 H 31.3889929 46.4988375 36.4574252

49 H 30.0389330 44.1470768 35.0486821

50 H 31.7843986 44.2161381 35.2420656

51 H 30.8771672 44.6925585 37.9284093

52 H 33.0977417 42.7437018 37.5205607

53 H 30.8198952 41.8982376 36.7408171

54 H 30.4658366 42.3715583 38.4044163

55 H 28.5393817 42.4818798 35.9688486

56 H 28.5538529 41.2825811 37.2721136

57 H 28.0483511 43.2230608 38.8184666

58 H 27.7638354 44.1608858 37.4959129

59 H 26.8149243 42.8137881 37.7826683

60 H 28.8667323 46.3909123 36.3043309

61 H 29.6277712 47.7656191 35.7075690

62 H 29.0179277 46.6687789 34.6616591

63 H 34.7457580 50.3223524 36.2871779

64 H 38.8437327 48.6098142 40.3375775

BesD4-PC-OH

QM(B1)/MM Energy = -3362.420373909443 a.u.

QM(B2)/MM Energy = -3364.544301000000 a.u.

QM(B3)/MM Energy = -3364.007842303593 a.u.

1 N 36.0578771 47.4354906 36.1404898

2 C 34.7924689 47.9426938 36.3653093

3 C 34.3250878 49.2595169 35.8308592

4 N 35.0418484 45.9614226 37.4036650

5 C 34.1711461 47.0001570 37.1549931

6 C 36.1774643 46.2606981 36.7846615

7 H 33.2259799 49.2887515 35.8748746

8 H 34.6187078 49.3786031 34.7771123

9 H 33.1708847 47.0117298 37.5788278

10 H 37.0726027 45.6431005 36.7947073

11 H 36.7828899 47.8798242 35.5681351

12 N 36.3166967 47.6631511 41.3541505

13 C 37.5416555 47.2286830 40.8779279

14 C 38.8429149 47.9347764 41.0631966

15 N 35.9178533 45.7935639 40.2747261

16 C 37.2684209 46.0576947 40.2147681

17 C 35.3715208 46.7835792 40.9639976

18 H 38.9398911 48.3411006 42.0818844

19 H 39.6611642 47.2112370 40.9456542

20 H 37.9482991 45.3970418 39.6833395

21 H 34.3085924 46.8934368 41.1709322

22 H 36.1410824 48.4836225 41.9439654

23 Cl 32.7223142 44.6566856 39.7776201

24 Fe 34.9511818 44.4911105 38.9207775

25 O 31.9484722 43.9152811 36.8920079

26 O 35.4716166 42.4478816 39.0116999

27 C 36.7054994 42.7148118 38.7496351

28 O 36.9961981 43.8290878 38.2536044

29 C 37.7878110 41.7732490 39.1993800

30 C 39.0625333 41.8501949 38.3607222

31 C 38.9513950 40.9134043 37.1490970

32 O 39.7344339 39.9231053 37.1450344

33 O 38.0693373 41.1575454 36.2936938

34 H 38.0035346 42.0754579 40.2413321

35 H 37.3885584 40.7489164 39.2508208
36 H 39.9348898 41.5654484 38.9636358
37 H 39.1975946 42.8905945 38.0266423
38 N 29.4228396 46.9917266 35.5804468
39 C 30.6573423 46.1498763 35.4637290
40 C 31.5411742 46.6908428 34.3021572
41 O 31.2723268 47.8359865 33.8672624
42 C 30.2731990 44.6605839 35.4134292
43 C 30.5429715 43.9590622 36.7500561
44 C 29.9685164 42.5323065 36.8837085
45 C 28.4630652 42.3018773 36.7246266
46 N 27.6171049 43.1666355 37.6142899
47 O 32.4662248 45.9419957 33.9059425
48 H 31.2481656 46.3338803 36.3743318
49 H 29.2118314 44.5687411 35.1411168
50 H 30.8679892 44.1563789 34.6374345
51 H 30.1091744 44.5819844 37.5613611
52 H 32.1697034 44.0094827 37.8416234
53 H 30.4670905 41.8933481 36.1357596
54 H 30.2973596 42.1532310 37.8671257
55 H 28.1254459 42.4762662 35.6947302
56 H 28.2195772 41.2580155 36.9725080
57 H 27.9687252 43.1968114 38.5890447
58 H 27.5894382 44.1654493 37.3153090
59 H 26.6189652 42.8511693 37.6822919
60 H 28.7609928 46.6541161 36.3125580
61 H 29.6640281 47.9822059 35.7624445
62 H 28.8737746 46.9989989 34.6945176
63 H 34.7312547 50.1352560 36.3369982
64 H 39.0057831 48.7775871 40.3914739

BesD4-Iso1-TS

QM(B1)/MM Energy = -3362.321831910203 a.u.

QM(B2)/MM Energy = -3364.453740000000 a.u.

QM(B3)/MM Energy = -3363.930130531084 a.u.

1 N 36.0860592 47.3467884 36.1790541
2 C 34.8395342 47.8944186 36.4150140
3 C 34.3799386 49.2031041 35.8548135
4 N 35.0348548 45.9158135 37.4626727
5 C 34.1975738 46.9844258 37.2215749
6 C 36.1748042 46.1720933 36.8274270
7 H 33.2801457 49.2288700 35.8855836
8 H 34.6885450 49.3068407 34.8037804
9 H 33.2110882 47.0373439 37.6720627
10 H 37.0485301 45.5240900 36.8230691
11 H 36.8216870 47.7732367 35.6049153
12 N 36.3126313 47.5792131 41.3215431
13 C 37.5256336 47.1145021 40.8412744
14 C 38.8292926 47.8198553 41.0252995
15 N 35.8679167 45.6904281 40.2998493
16 C 37.2253160 45.9280419 40.2139223
17 C 35.3472977 46.7119914 40.9686473
18 H 38.9266432 48.2089880 42.0511443
19 H 39.6510768 47.1022225 40.8954125
20 H 37.8856055 45.2323184 39.7033379
21 H 34.2863829 46.8507065 41.1640563
22 H 36.1591633 48.4119486 41.9013910
23 Cl 32.7023402 45.3042273 39.7543690
24 Fe 34.8561105 44.3978409 38.9364597
25 O 33.9749240 42.8061774 39.0358109
26 O 36.1041111 41.4078183 37.9115748
27 C 36.7903104 42.2622584 38.4441837
28 O 36.5172147 43.5605150 38.4065443
29 C 38.0884043 41.9283774 39.1515691
30 C 39.2787618 42.0987498 38.1883909

31 C 39.2361824 41.0223423 37.0863715
32 O 39.8935581 39.9707176 37.3131447
33 O 38.5309364 41.2329949 36.0747305
34 H 38.2145827 42.5753179 40.0334577
35 H 38.0475688 40.8821478 39.4828832
36 H 40.2152801 41.9955110 38.7570656
37 H 39.2450012 43.1059914 37.7404289
38 N 29.4603215 46.7313146 35.5819253
39 C 30.8014496 46.0573166 35.6269446
40 C 31.6757893 46.5483035 34.4347292
41 O 31.2235353 47.4987246 33.7456077
42 C 30.6915448 44.5306049 35.7220451
43 C 30.1873882 44.0177490 37.0345690
44 C 29.7243506 42.6012904 37.2293592
45 C 28.2282818 42.3298081 36.9877898
46 N 27.3693387 43.2740434 37.7666245
47 O 32.7653983 45.9609698 34.2584724
48 H 31.2858532 46.4292572 36.5444327
49 H 30.1148438 44.1234275 34.8714118
50 H 31.7259878 44.1668580 35.5545072
51 H 30.4548822 44.5964507 37.9261147
52 H 33.0756075 42.8628629 39.3868430
53 H 30.2691871 41.9165447 36.5528256
54 H 29.9754123 42.2783875 38.2543587
55 H 27.9575227 42.4379858 35.9291987
56 H 27.9684072 41.3066736 37.2969600
57 H 27.6818257 43.3541626 38.7513795
58 H 27.4045335 44.2493983 37.3990060
59 H 26.3624586 42.9890929 37.8151352
60 H 28.7735466 46.3825469 36.2888238
61 H 29.5804451 47.7505955 35.7261669
62 H 28.9871112 46.6685384 34.6553898
63 H 34.7691740 50.0887066 36.3570795
64 H 38.9930401 48.6778321 40.3732797

BesD4-Iso1-IM

QM(B1)/MM Energy = -3362.330447694167 a.u.

QM(B2)/MM Energy = -3364.463447000000 a.u.

QM(B3)/MM Energy = -3363.939988066623 a.u.

1 N 36.0721441 47.3565934 36.2092701
2 C 34.8281668 47.9071648 36.4455872
3 C 34.3646277 49.2101056 35.8738637
4 N 35.0301516 45.9432738 37.5296273
5 C 34.1953840 47.0057890 37.2714854
6 C 36.1599888 46.1889359 36.8800501
7 H 33.2645973 49.2352099 35.9047160
8 H 34.6704384 49.3095583 34.8216106
9 H 33.1984447 47.0476978 37.6975856
10 H 37.0300449 45.5364857 36.8707885
11 H 36.8030581 47.7736315 35.6234950
12 N 36.3071091 47.7389375 41.3409832
13 C 37.5115657 47.2512341 40.8663301
14 C 38.8333815 47.9195416 41.0520628
15 N 35.8282516 45.8890461 40.2793691
16 C 37.1880274 46.0866430 40.2113994
17 C 35.3224217 46.9032872 40.9608297
18 H 38.9429254 48.3109956 42.0752262
19 H 39.6359294 47.1805171 40.9222452
20 H 37.8386074 45.3835066 39.6972988
21 H 34.2677638 47.0690198 41.1704090
22 H 36.1633517 48.5648659 41.9318375
23 Cl 32.5138252 44.7288155 39.0814223
24 Fe 34.8604663 44.4205717 39.1556176
25 O 34.7026277 43.1162923 40.4340997
26 O 35.5756965 41.4877208 37.9081241

27 C 36.4332971 42.2436207 38.3460501
28 O 36.3273691 43.5538880 38.2371680
29 C 37.6822907 41.7657500 39.0518975
30 C 38.9583118 41.9935385 38.2227611
31 C 39.0398053 40.9624220 37.0850703
32 O 39.8470278 40.0088819 37.2473068
33 O 38.2677911 41.1017637 36.1079336
34 H 37.7478616 42.3122353 40.0061329
35 H 37.5672857 40.6949793 39.2717470
36 H 39.8389588 41.8863342 38.8724798
37 H 38.9493786 43.0162645 37.8098473
38 N 29.4266671 46.7348944 35.5194378
39 C 30.7803265 46.0859327 35.5725080
40 C 31.6618116 46.6126755 34.4055969
41 O 31.2032615 47.5619776 33.7182293
42 C 30.6988581 44.5569487 35.6272691
43 C 29.9618412 44.0283608 36.8125951
44 C 29.6280254 42.5805626 37.0120049
45 C 28.1261273 42.2418282 36.9547790
46 N 27.3138950 43.2292945 37.7296628
47 O 32.7615461 46.0442197 34.2339875
48 H 31.2387759 46.4376913 36.5110016
49 H 30.2961129 44.1454732 34.6821260
50 H 31.7542831 44.2204130 35.6531285
51 H 29.8872742 44.6819174 37.6875336
52 H 33.8868302 42.6041484 40.5248048
53 H 30.1238473 41.9494270 36.2558187
54 H 30.0269031 42.2605428 37.9931560
55 H 27.7524935 42.2553035 35.9233382
56 H 27.9287170 41.2431012 37.3709907
57 H 27.6647547 43.3421392 38.6983963
58 H 27.3490419 44.1895556 37.3220677
59 H 26.3064021 42.9611915 37.8252357
60 H 28.7371472 46.3619374 36.2113079
61 H 29.5269431 47.7531476 35.6824822
62 H 28.9658621 46.6783091 34.5866122
63 H 34.7527217 50.0981905 36.3726175
64 H 39.0079678 48.7683123 40.3908707

BesD4-Iso1-TS-Cl

QM(B1)/MM Energy = -3362.320267909412 a.u.

QM(B2)/MM Energy = -3364.451998000000 a.u.

QM(B3)/MM Energy = -3363.929330336129 a.u.

1 N 36.1026088 47.4321188 36.1723941
2 C 34.8468599 47.9706320 36.3668563
3 C 34.3878788 49.2754959 35.8012212
4 N 35.0339512 46.0161693 37.4616924
5 C 34.1968171 47.0656351 37.1723178
6 C 36.1823650 46.2688191 36.8500830
7 H 33.2877191 49.2944570 35.8213907
8 H 34.7061943 49.3912767 34.7544403
9 H 33.1826191 47.0938881 37.5541733
10 H 37.0576371 45.6234828 36.8685156
11 H 36.8422358 47.8481884 35.5977570
12 N 36.2269499 47.7578641 41.3509799
13 C 37.4043135 47.2477739 40.8348658
14 C 38.7508752 47.8684006 41.0254360
15 N 35.6584993 45.9633067 40.2322616
16 C 37.0250685 46.1180737 40.1485804
17 C 35.2067994 46.9680624 40.9626567
18 H 38.8754169 48.2347414 42.0566137
19 H 39.5316913 47.1088400 40.8780305
20 H 37.6389474 45.4068236 39.6016898
21 H 34.1677103 47.1650359 41.2181614
22 H 36.1203059 48.5696596 41.9679774

23 Cl 32.4205549 44.4098616 38.2474413
24 Fe 34.7237862 44.4973888 39.0104055
25 O 34.2736636 43.3764324 40.3940432
26 O 35.4857633 41.5382558 38.0739762
27 C 36.3886177 42.2962521 38.4178341
28 O 36.2937830 43.5953287 38.2751748
29 C 37.6698566 41.8137278 39.0653387
30 C 38.9192484 42.0361996 38.1960085
31 C 38.9831041 40.9809192 37.0785910
32 O 39.8072310 40.0416960 37.2372317
33 O 38.1819077 41.0900885 36.1211122
34 H 37.7743908 42.3605977 40.0171189
35 H 37.5623917 40.7439196 39.2932445
36 H 39.8184623 41.9508967 38.8232363
37 H 38.8882284 43.0497989 37.7622926
38 N 29.4338123 46.8298694 35.5321447
39 C 30.7810181 46.1666718 35.5774488
40 C 31.6389820 46.6524429 34.3761966
41 O 31.1768407 47.5945236 33.6803314
42 C 30.6719274 44.6456690 35.6909003
43 C 30.2133653 44.1633916 37.0364402
44 C 29.8472992 42.7252480 37.2751961
45 C 28.3828761 42.3393554 36.9821049
46 N 27.4309566 43.2394284 37.7082415
47 O 32.7298118 46.0715376 34.1926637
48 H 31.2704455 46.5448101 36.4887239
49 H 30.0439617 44.2437891 34.8701647
50 H 31.6850053 44.2573795 35.4920891
51 H 29.7971664 44.8985180 37.7305970
52 H 33.8477666 42.5425892 40.1479583
53 H 30.4825180 42.0612384 36.6649765
54 H 30.0704672 42.4729909 38.3262370
55 H 28.1408746 42.4022472 35.9120221
56 H 28.1807273 41.3095660 37.3136926
57 H 27.7092521 43.3526294 38.7002416
58 H 27.4159423 44.2089932 37.3240601
59 H 26.4411041 42.8981248 37.7360938
60 H 28.7459273 46.4676652 36.2293171
61 H 29.5425321 47.8488024 35.6813191
62 H 28.9671986 46.7623886 34.6027398
63 H 34.7690856 50.1541264 36.3215643
64 H 38.9540398 48.7267174 40.3850584

BesD4-Iso1-PC-Cl

QM(B1)/MM Energy = -3362.362716986123 a.u.

QM(B2)/MM Energy = -3364.497969000000 a.u.

QM(B3)/MM Energy = -3363.962499647668 a.u.

1 N 36.1167232 47.4361296 36.2797282
2 C 34.8408899 47.9411807 36.4198306
3 C 34.3835373 49.2391184 35.8330989
4 N 35.0398220 46.0011447 37.5492767
5 C 34.1841786 47.0230498 37.2100800
6 C 36.2026755 46.2839951 36.9770840
7 H 33.2831214 49.2596856 35.8465508
8 H 34.7076812 49.3403599 34.7861102
9 H 33.1562675 47.0418486 37.5580880
10 H 37.0979921 45.6699714 37.0481318
11 H 36.8662597 47.8632184 35.7264224
12 N 36.3469353 47.8594775 41.4397230
13 C 37.5330710 47.3490716 40.9493676
14 C 38.8699845 47.9963778 41.0859929
15 N 35.8177120 45.9968847 40.4070992
16 C 37.1789294 46.1796433 40.3198001
17 C 35.3450598 47.0261771 41.0843132
18 H 39.0213617 48.3976802 42.1000611

19 H	39.6537916	47.2405302	40.9400658
20 H	37.8067054	45.4554138	39.8064184
21 H	34.3013142	47.2148878	41.3285502
22 H	36.2108869	48.6951313	42.0146196
23 Cl	31.7143396	44.0797858	37.9152708
24 Fe	34.9165721	44.5494072	39.1350543
25 O	33.4416700	43.8070001	40.0462548
26 O	35.4775659	41.9162056	38.6518382
27 C	36.5886687	42.4710402	38.7749931
28 O	36.7257227	43.7398181	38.6199243
29 C	37.8192835	41.7129623	39.2232945
30 C	39.0467195	41.9153417	38.3227387
31 C	38.9960478	40.9207790	37.1549047
32 O	39.7788271	39.9345665	37.2274568
33 O	38.1475321	41.1079851	36.2524214
34 H	38.0481928	42.0868836	40.2366111
35 H	37.5718831	40.6455149	39.3091536
36 H	39.9659045	41.7434338	38.9003569
37 H	39.0543548	42.9536920	37.9550331
38 N	29.3889180	46.7938005	35.4830683
39 C	30.6972370	46.0588691	35.4909492
40 C	31.5911420	46.5816497	34.3275185
41 O	31.1526032	47.5474330	33.6527893
42 C	30.4980409	44.5366697	35.4970711
43 C	30.2164162	43.9492590	36.8831378
44 C	29.7519863	42.4836973	36.9236589
45 C	28.2686630	42.2339172	36.6413371
46 N	27.3905259	43.0850513	37.5090307
47 O	32.6807290	45.9955491	34.1597253
48 H	31.2097132	46.3512983	36.4205858
49 H	29.6885292	44.2600198	34.8028751
50 H	31.4185719	44.0845221	35.1021275
51 H	29.4980039	44.5767602	37.4246167
52 H	33.5458284	42.8538805	39.8915346
53 H	30.3435133	41.8801740	36.2158097
54 H	29.9846440	42.0927870	37.9265557
55 H	27.9948284	42.4280499	35.5973445
56 H	28.0250407	41.1835041	36.8568585
57 H	27.7426248	43.1240036	38.4845439
58 H	27.3466102	44.0792925	37.1985049
59 H	26.3979362	42.7586580	37.5865031
60 H	28.6801469	46.4379359	36.1617660
61 H	29.5474071	47.8007744	35.6721458
62 H	28.9239379	46.7830837	34.5496369
63 H	34.7612572	50.1266357	36.3407481
64 H	39.0371379	48.8349002	40.4099699

BesD4-Iso2-TS

QM(B1)/MM Energy = -3362.327332920506 a.u.

QM(B2)/MM Energy = -3364.460303000000 a.u.

QM(B3)/MM Energy = -3363.935076875811 a.u.

1 N	36.1178585	47.5125755	36.2061132
2 C	34.8441848	48.0279008	36.3309642
3 C	34.3995659	49.3343431	35.7611906
4 N	35.0211326	46.1065531	37.4764895
5 C	34.1719431	47.1228133	37.1178414
6 C	36.1916900	46.3678804	36.9128877
7 H	33.2996424	49.3564233	35.7692093
8 H	34.7323710	49.4578238	34.7198171
9 H	33.1347377	47.1390338	37.4330623
10 H	37.0804701	45.7467862	36.9901345
11 H	36.8755958	47.9330194	35.6584275
12 N	36.2672182	47.6830003	41.3654563
13 C	37.4526930	47.2248706	40.8223020
14 C	38.7845805	47.8727219	41.0219836

15 N	35.7431195	45.9038645	40.1906774
16 C	37.1002035	46.1075170	40.1013998
17 C	35.2694739	46.8695426	40.9591171
18 H	38.8952682	48.2426027	42.0532742
19 H	39.5808714	47.1281569	40.8804477
20 H	37.7337689	45.4364039	39.5283070
21 H	34.2274866	47.0231739	41.2321573
22 H	36.1428754	48.4827309	41.9950979
23 Cl	32.9145164	43.8079359	37.4874248
24 Fe	34.6543301	44.5114487	38.8720081
25 O	33.8178384	43.7480000	40.3447407
26 O	35.6356545	41.6602955	39.0227237
27 C	36.5665692	42.4442703	38.8574868
28 O	36.3597532	43.6700620	38.4484240
29 C	38.0014895	42.0886891	39.1885740
30 C	38.9980030	42.2539646	38.0296363
31 C	38.9302854	41.0608690	37.0518758
32 O	39.6975349	40.0919953	37.2996344
33 O	38.1047831	41.1135579	36.1134147
34 H	38.2939260	42.7430335	40.0289368
35 H	38.0250098	41.0552501	39.5589961
36 H	40.0131623	42.2995742	38.4511780
37 H	38.7918668	43.1957095	37.4951710
38 N	29.3980861	46.7395555	35.4154660
39 C	30.7279930	46.0471886	35.3828827
40 C	31.6301436	46.6716959	34.2878782
41 O	31.1823103	47.6606623	33.6516201
42 C	30.5846086	44.5297968	35.2508495
43 C	29.8808151	43.9057802	36.4094731
44 C	29.5831664	42.4438241	36.5378069
45 C	28.0953749	42.1009105	36.7557593
46 N	27.4090117	43.1117514	37.6183653
47 O	32.7393616	46.1233203	34.1103351
48 H	31.2057255	46.2558352	36.3530093
49 H	30.1252686	44.2559838	34.2829407
50 H	31.6221812	44.1533801	35.1991106
51 H	29.8479556	44.4900123	37.3358507
52 H	33.9214550	42.7880576	40.2038553
53 H	29.9148220	41.8907772	35.6442776
54 H	30.1752199	42.0479372	37.3854822
55 H	27.5531031	42.0755113	35.8041400
56 H	27.9755638	41.1180981	37.2341413
57 H	27.8635672	43.2069461	38.5450257
58 H	27.4295967	44.0725296	37.2095995
59 H	26.4060638	42.8778287	37.8036957
60 H	28.7200322	46.3322071	36.0993395
61 H	29.5244120	47.7433696	35.6381348
62 H	28.9146079	46.7408848	34.4924990
63 H	34.7789895	50.2033486	36.2987167
64 H	38.9749835	48.7333867	40.3808362

BesD-Iso2-IM

QM(B1)/MM Energy = -3362.334715696351 a.u.

QM(B2)/MM Energy = -3364.464461000000 a.u.

QM(B3)/MM Energy = -3363.937964925303 a.u.

1 N	35.9625855	47.4000960	36.2110264
2 C	34.7040150	47.9352732	36.4031418
3 C	34.2712121	49.2577898	35.8572849
4 N	34.8814762	45.9568158	37.4560743
5 C	34.0427790	47.0112347	37.1804993
6 C	36.0378098	46.2238726	36.8628064
7 H	33.1726674	49.3103174	35.8940850
8 H	34.5774403	49.3707275	34.8066272
9 H	33.0253778	47.0408745	37.5562529
10 H	36.9125289	45.5804554	36.8868768

11 H 36.7167902 47.8304119 35.6655284
12 N 36.2885424 47.5467971 41.2647639
13 C 37.5081493 47.1075351 40.7846621
14 C 38.8038835 47.8176559 41.0069097
15 N 35.8700658 45.6910537 40.1541065
16 C 37.2214528 45.9452608 40.1018691
17 C 35.3373355 46.6801930 40.8572757
18 H 38.8755666 48.2019911 42.0366615
19 H 39.6347908 47.1083500 40.8884388
20 H 37.9043749 45.2874833 39.5703611
21 H 34.2744993 46.8096498 41.0495971
22 H 36.1264952 48.3587490 41.8710526
23 Cl 32.9397343 43.3238877 37.4920182
24 Fe 34.5514597 44.3173852 38.8063691
25 O 33.3013184 44.6729059 40.1849621
26 O 35.4135062 42.5097122 39.4821766
27 C 36.5024900 42.7130374 38.8464250
28 O 36.5531828 43.7112239 38.0771270
29 C 37.7050898 41.8540797 39.1358503
30 C 38.8948740 42.0037174 38.1791308
31 C 38.8984013 40.9271989 37.0759434
32 O 39.7356143 39.9934919 37.2190767
33 O 38.0672215 41.0242617 36.1473005
34 H 38.0036989 42.1416575 40.1606563
35 H 37.3731639 40.8066095 39.2196735
36 H 39.8298271 41.9131219 38.7497905
37 H 38.8584244 43.0036823 37.7193701
38 N 29.3922074 46.7177040 35.4285561
39 C 30.7215608 46.0228615 35.4129119
40 C 31.6241369 46.6236670 34.3018600
41 O 31.1901307 47.6235135 33.6741834
42 C 30.5796723 44.5019490 35.3210660
43 C 29.8703543 43.8986432 36.4881947
44 C 29.5520233 42.4404231 36.6267083
45 C 28.0538371 42.1037300 36.7712697
46 N 27.3329508 43.1195482 37.5980295
47 O 32.7176798 46.0498595 34.1064686
48 H 31.1979984 46.2631576 36.3765349
49 H 30.1253910 44.2046098 34.3568315
50 H 31.6180224 44.1229734 35.2841913
51 H 29.8398709 44.4929303 37.4086946
52 H 33.8927137 44.7108550 40.9465450
53 H 29.9277637 41.8693608 35.7622275
54 H 30.0944051 42.0560563 37.5110883
55 H 27.5568360 42.0725539 35.7951576
56 H 27.9106447 41.1230237 37.2476700
57 H 27.7573969 43.2292142 38.5374846
58 H 27.3631775 44.0774375 37.1835951
59 H 26.3269577 42.8818832 37.7607643
60 H 28.7082492 46.3210165 36.1130073
61 H 29.5192801 47.7238944 35.6405280
62 H 28.9131079 46.7088604 34.5032294
63 H 34.6872777 50.1226493 36.3739808
64 H 38.9722113 48.6796206 40.3613481

BesD4-Iso2-TS-Cl

QM(B1)/MM Energy = -3362.323810928314 a.u.

QM(B2)/MM Energy = -3364.452097000000 a.u.

QM(B3)/MM Energy = -3363.930776137630 a.u.

1 N 35.9741921 47.4607419 36.2081958
2 C 34.7111427 47.9910198 36.3789874
3 C 34.2783393 49.3134467 35.8333643
4 N 34.8810026 46.0190136 37.4478430
5 C 34.0430863 47.0679810 37.1510924
6 C 36.0434862 46.2888306 36.8699355

7 H 33.1797191 49.3649719 35.8712722
8 H 34.5838103 49.4325798 34.7828824
9 H 33.0218712 47.0977077 37.5151014
10 H 36.9203881 45.6497206 36.9066425
11 H 36.7316324 47.8865261 35.6645454
12 N 36.2812969 47.5575205 41.3000890
13 C 37.4950507 47.1318736 40.7910517
14 C 38.7961256 47.8348138 41.0055433
15 N 35.8536699 45.7157615 40.1718943
16 C 37.2011632 45.9790587 40.0969385
17 C 35.3279327 46.6866321 40.9015945
18 H 38.8765698 48.2186321 42.0349038
19 H 39.6210379 47.1190212 40.8824514
20 H 37.8755260 45.3288496 39.5453589
21 H 34.2688378 46.7872709 41.1294642
22 H 36.1234484 48.3660330 41.9112607
23 Cl 32.5664559 43.9285566 37.4213562
24 Fe 34.5149749 44.3776403 38.8083626
25 O 33.3208163 44.5533513 40.2767932
26 O 35.2108437 42.3838747 39.1017765
27 C 36.3897848 42.6735261 38.6944436
28 O 36.5537595 43.7604776 38.0832750
29 C 37.5582319 41.8135513 39.1000533
30 C 38.8153615 41.9495816 38.2341199
31 C 38.8345823 40.9070363 37.1035753
32 O 39.7233128 40.0161169 37.1794005
33 O 37.9505214 40.9855247 36.2190141
34 H 37.7846899 42.1384117 40.1329410
35 H 37.2261733 40.7668408 39.1832708
36 H 39.7130158 41.8182783 38.8539663
37 H 38.8366014 42.9633454 37.8029214
38 N 29.4157496 46.7941539 35.4815334
39 C 30.7162742 46.0533087 35.4168310
40 C 31.5921803 46.6297467 34.2712255
41 O 31.1694023 47.6499993 33.6712421
42 C 30.4991862 44.5364953 35.3543360
43 C 30.2685407 43.9090021 36.7017689
44 C 29.8598381 42.4747477 36.8786579
45 C 28.3570395 42.1753332 36.7161987
46 N 27.5093815 43.0768915 37.5595201
47 O 32.6608744 46.0257302 34.0370866
48 H 31.2550021 46.2806671 36.3483350
49 H 29.6823874 44.2998234 34.6478869
50 H 31.4034612 44.1017430 34.9048398
51 H 29.9615845 44.5834640 37.5071021
52 H 33.9616840 44.4270137 40.9868341
53 H 30.3948204 41.8267856 36.1651700
54 H 30.1770010 42.1517613 37.8847760
55 H 28.0210381 42.2877999 35.6774312
56 H 28.1430006 41.1409091 37.0228089
57 H 27.8626053 43.1402983 38.5326689
58 H 27.4975195 44.0650454 37.2251904
59 H 26.5089813 42.7759805 37.6393076
60 H 28.7446330 46.4226551 36.1896483
61 H 29.5819234 47.7970906 35.6820143
62 H 28.9056453 46.7890369 34.5724127
63 H 34.6930760 50.1759006 36.3551249
64 H 38.9676131 48.6954270 40.3590110

BesD4-Iso2-PC-Cl

QM(B1)/MM Energy = -3362.362747986677 a.u.

QM(B2)/MM Energy = -3364.497864000000 a.u.

QM(B3)/MM Energy = -3363.962557331370 a.u.

1 N 36.1070914 47.4376194 36.2772657
2 C 34.8309240 47.9420077 36.4163035

3 C	34.3753983	49.2425047	35.8337875
4 N	35.0284601	45.9992083	37.5416017
5 C	34.1729811	47.0215713	37.2029471
6 C	36.1921991	46.2838212	36.9719535
7 H	33.2750524	49.2650401	35.8476325
8 H	34.6991428	49.3467903	34.7869317
9 H	33.1442184	47.0400839	37.5485974
10 H	37.0877168	45.6701109	37.0429463
11 H	36.8573788	47.8660461	35.7260214
12 N	36.3386046	47.8537066	41.4360358
13 C	37.5247371	47.3441135	40.9448073
14 C	38.8619594	47.9906553	41.0835540
15 N	35.8092267	45.9929061	40.3998368
16 C	37.1705126	46.1758840	40.3130190
17 C	35.3366846	47.0211504	41.0788720
18 H	39.0123978	48.3905894	42.0983430
19 H	39.6455935	47.2346301	40.9372897
20 H	37.7983418	45.4528172	39.7980876
21 H	34.2929445	47.2093189	41.3236069
22 H	36.2031285	48.6883813	42.0125575
23 Cl	31.7003337	44.0403031	37.8840185
24 Fe	34.9075965	44.5450259	39.1272611
25 O	33.4257584	43.8035874	40.0278890
26 O	35.4840500	41.9078785	38.6741608
27 C	36.5918156	42.4724786	38.7782662
28 O	36.7174915	43.7405158	38.6065386
29 C	37.8329681	41.7286525	39.2222006
30 C	39.0506493	41.9335889	38.3091507
31 C	38.9961639	40.9309258	37.1481507
32 O	39.7780835	39.9444554	37.2257582
33 O	38.1458987	41.1124071	36.2462508
34 H	38.0668148	42.1120160	40.2309207
35 H	37.5958723	40.6598644	39.3191695
36 H	39.9761129	41.7709652	38.8795747
37 H	39.0476298	42.9695485	37.9347700
38 N	29.3719396	46.7567875	35.4782595
39 C	30.6824216	46.0272530	35.4829502
40 C	31.5834791	46.5735326	34.3355925
41 O	31.1525148	47.5576020	33.6835171
42 C	30.4891879	44.5040281	35.4670050
43 C	30.2040997	43.9018479	36.8469782
44 C	29.7476280	42.4329652	36.8841701
45 C	28.2607334	42.1725178	36.6281115
46 N	27.3907036	43.0305656	37.4958068
47 O	32.6706186	45.9855768	34.1563009
48 H	31.1873118	46.3057366	36.4207982
49 H	29.6883808	44.2314311	34.7597703
50 H	31.4156351	44.0623660	35.0752051
51 H	29.4813966	44.5226367	37.3895316
52 H	33.5301495	42.8511318	39.8689795
53 H	30.3306182	41.8361150	36.1638365
54 H	29.9992094	42.0384830	37.8809269
55 H	27.9667812	42.3482116	35.5870432
56 H	28.0251567	41.1237535	36.8606513
57 H	27.7452467	43.0731475	38.4699207
58 H	27.3460988	44.0238779	37.1826047
59 H	26.3970478	42.7086593	37.5753362
60 H	28.6637592	46.3952636	36.1547046
61 H	29.5257904	47.7634224	35.6728589
62 H	28.9071574	46.7454553	34.5448204
63 H	34.7552608	50.1275965	36.3440659
64 H	39.0307054	48.8299764	40.4089192

BesD4-Iso2-TS-OH

QM(B1)/MM Energy = -3362.293853370662 a.u.

QM(B2)/MM Energy = -3364.418698000000 a.u.

QM(B3)/MM Energy = -3363.899471162626 a.u.

1 N	35.9357446	47.3589220	36.0126463
2 C	34.7098615	47.9549306	36.2242539
3 C	34.3102951	49.3095478	35.7416272
4 N	34.7270711	45.8795894	37.0958997
5 C	33.9821039	47.0143027	36.9092747
6 C	35.9143794	46.1239147	36.5575281
7 H	33.2111242	49.3612080	35.7537998
8 H	34.6395810	49.4748045	34.7049662
9 H	32.9712096	47.1002976	37.2803290
10 H	36.7471925	45.4254146	36.5641627
11 H	36.7290566	47.7898850	35.5260903
12 N	35.8671277	47.5432257	41.1417266
13 C	37.0460954	47.0468620	40.6154139
14 C	38.4095279	47.6039133	40.9052425
15 N	35.2915774	45.7945216	39.9093890
16 C	36.6605173	45.9599154	39.8602573
17 C	34.8501427	46.7706543	40.6849000
18 H	38.4883189	47.9090117	41.9620131
19 H	39.1614582	46.8131770	40.7623410
20 H	37.2878648	45.2937883	39.2751124
21 H	33.8106396	46.9624548	40.9400306
22 H	35.7796367	48.3242889	41.8021162
23 Cl	33.2401398	42.4432804	37.1328317
24 Fe	34.2353878	44.2130907	38.3970352
25 O	32.3241509	44.7867611	38.8130054
26 O	35.2517352	42.6881143	39.4510786
27 C	36.3212812	42.7941298	38.7588933
28 O	36.3780489	43.6782214	37.8646030
29 C	37.5077932	41.9151532	39.0948093
30 C	38.7436356	42.0440957	38.1890277
31 C	38.8268635	40.9691869	37.0836389
32 O	39.7029918	40.0726439	37.2432366
33 O	38.0290328	41.0399148	36.1239306
34 H	37.7740186	42.1826322	40.1328389
35 H	37.1530068	40.8721159	39.1531785
36 H	39.6496450	41.9630669	38.8067192
37 H	38.7319842	43.0390747	37.7162424
38 N	29.4389126	46.8089685	35.5684673
39 C	30.8355444	46.2621981	35.7001030
40 C	31.6253803	46.5841244	34.3894690
41 O	31.1723688	47.5267761	33.6879904
42 C	30.8877328	44.7965306	36.1241069
43 C	30.4984050	44.4709896	37.5363130
44 C	30.0750496	43.0577543	37.8429160
45 C	28.6891167	42.6125890	37.3278418
46 N	27.5615208	43.3683702	37.9604959
47 O	32.6428552	45.9059796	34.1441903
48 H	31.2852575	46.8779002	36.4920287
49 H	30.2818542	44.1812582	35.4269028
50 H	31.9170574	44.4334585	35.9555505
51 H	30.0360638	45.2663833	38.1287706
52 H	32.2691884	45.7538041	38.8433759
53 H	30.8132673	42.3739641	37.3980230
54 H	30.1167089	42.8729401	38.9241950
55 H	28.5796416	42.7176977	36.2381587
56 H	28.5394451	41.5499345	37.5698138
57 H	27.6973997	43.4450127	38.9843230
58 H	27.4982801	44.3484909	37.6147135
59 H	26.6119782	42.9368650	37.8469943
60 H	28.7792751	46.5220112	36.3250305
61 H	29.4853774	47.8432867	35.5697883
62 H	28.9665439	46.5938266	34.6651963
63 H	34.7108022	50.1499989	36.3084633
64 H	38.7147013	48.4820685	40.3362260

BesD4-Iso2-TS-OH

QM(B1)/MM Energy = -3362.393538026630 a.u.

QM(B2)/MM Energy = -3364.517819000000 a.u.

QM(B3)/MM Energy = -3363.980854059354 a.u.

1 N	36.1352984	47.4378602	36.1239213
2 C	34.8652501	47.9199597	36.3657158
3 C	34.3635473	49.2278484	35.8402555
4 N	35.1466612	45.9191700	37.3596282
5 C	34.2643210	46.9515297	37.1403402
6 C	36.2723731	46.2480721	36.7416400
7 H	33.2645327	49.2357414	35.8998462
8 H	34.6429653	49.3561922	34.7834718
9 H	33.2567456	46.9386310	37.5468829
10 H	37.1768910	45.6423839	36.7289465
11 H	36.8476098	47.9012641	35.5518678
12 N	36.2332839	47.5917921	41.2366054
13 C	37.4696980	47.1517153	40.8025914
14 C	38.7692557	47.8527212	41.0322144
15 N	35.8596585	45.7052954	40.1587721
16 C	37.2134527	45.9740744	40.1420926
17 C	35.2997765	46.7060530	40.8212452
18 H	38.8361776	48.2461106	42.0588115
19 H	39.5901088	47.1290847	40.9304131
20 H	37.9129117	45.3142494	39.6356566
21 H	34.2350889	46.8411851	41.0020338
22 H	36.0513604	48.4084555	41.8311014
23 Cl	33.3424097	42.9394806	38.0127435
24 Fe	35.1898817	44.2343024	38.6895160
25 O	30.9917850	44.6774448	38.4269692
26 O	35.9989846	42.3482719	39.5956174
27 C	37.0748030	42.6263990	39.0066392
28 O	37.1182745	43.6403140	38.2209024
29 C	38.3408144	41.8702495	39.3088648
30 C	39.4093411	41.9602108	38.2195220
31 C	39.1539519	40.9129694	37.1206542
32 O	39.7704106	39.8182231	37.2677312
33 O	38.3438903	41.1707413	36.2049499
34 H	38.7124074	42.2998425	40.2570793
35 H	38.0859905	40.8254873	39.5389939
36 H	40.3961532	41.7604291	38.6629583
37 H	39.3959837	42.9767611	37.8017571
38 N	29.3438262	46.8641591	35.6312531
39 C	30.6829653	46.1852751	35.7624919
40 C	31.5595376	46.5628507	34.5227305
41 O	31.1324259	47.5032007	33.7992177
42 C	30.5381765	44.6844860	36.0200295
43 C	30.0464363	44.3217089	37.4395377
44 C	29.6254768	42.8398568	37.5651035
45 C	28.2273799	42.4923429	37.0533721
46 N	27.1870256	43.3561619	37.7081347
47 O	32.6133724	45.9201401	34.3407848
48 H	31.1611247	46.6436579	36.6438648
49 H	29.8838106	44.2291076	35.2566397
50 H	31.5367314	44.2426766	35.8828429
51 H	29.1740722	44.9425854	37.6873718
52 H	31.8060930	44.1448455	38.2699604
53 H	30.3553930	42.1902475	37.0556894
54 H	29.6863111	42.5763579	38.6334431
55 H	28.1182151	42.6138713	35.9661956
56 H	27.9825229	41.4471132	37.2909354
57 H	27.4047588	43.4974029	38.7120215
58 H	27.1282647	44.3159784	37.3063433
59 H	26.2181537	42.9585695	37.7031594
60 H	28.6098515	46.5492732	36.3024484

61 H 29.4644870 47.8864849 35.7379265

62 H 28.9193728 46.7628946 34.6841748

63 H 34.7561879 50.1116798 36.3429906

64 H 38.9609602 48.7040375 40.3790856

BesD3-RC

QM(B1)/MM Energy = -3361.983932020749 a.u.

QM(B2)/MM Energy = -3364.114264000000 a.u.

QM(B3)/MM Energy = -3363.591425157907 a.u.

1 N 35.6402732 45.5603777 47.5895156

2 C 34.3019036 45.5308956 47.9159456

3 C 33.6576039 46.4063757 48.9427477

4 N 34.7577246 43.9620395 46.3818853

5 C 33.7660582 44.5259447 47.1444877

6 C 35.8806776 44.5987066 46.6724670

7 H 32.5938475 46.5009820 48.6786088

8 H 34.0822743 47.4211493 48.9342643

9 H 32.7451798 44.1550282 47.1068372

10 H 36.8570356 44.3962410 46.2380428

11 H 36.3266134 46.2019937 47.9995178

12 N 35.0109688 40.4265473 49.0540260

13 C 36.3228739 40.7130380 48.7163919

14 C 37.5055747 40.5776341 49.6157703

15 N 34.9584342 41.1955209 46.9949383

16 C 36.2650253 41.1758259 47.4262932

17 C 34.2247162 40.7371232 48.0001256

18 H 37.4509065 39.6650784 50.2303428

19 H 38.4152059 40.4762320 49.0068807

20 H 37.0758941 41.4879161 46.7768216

21 H 33.1423339 40.6361330 47.9883799

22 H 34.6913201 39.9965687 49.9304305

23 Cl 32.2558819 41.5906744 45.5996220

24 Fe 34.4466343 42.2340755 45.1985498

25 O 34.0430880 43.0455613 43.8654326

26 O 36.2094308 41.4236396 42.4390487

27 C 36.7578850 41.4279589 43.5203777

28 O 36.1794381 41.7160640 44.6745159

29 C 38.2207733 41.0462261 43.7049422

30 C 39.1705027 42.2566070 43.7856164

31 C 39.4256509 42.8674571 42.3947134

32 O 40.3861645 42.3868895 41.7360369

33 O 38.6470256 43.7685920 42.0036923

34 H 38.3031484 40.4404742 44.6192046

35 H 38.5130816 40.4168890 42.8559177

36 H 40.1326160 41.9180591 44.1998767

37 H 38.7496442 43.0226962 44.4595306

38 N 29.5373038 45.9783678 45.9270523

39 C 30.8099887 45.9596574 45.1206245

40 C 31.5905105 47.2717486 45.4310110

41 O 30.9687510 48.1523807 46.0707845

42 C 30.5313167 45.7486766 43.6251352

43 C 30.2540982 44.2778441 43.2712759

44 C 29.8165000 44.0113082 41.8209599

45 C 28.4262366 44.5025868 41.4081284

46 N 27.3613008 43.9755777 42.3220561

47 O 32.7684602 47.3417435 45.0163533

48 H 31.4155333 45.1175823 45.4848321

49 H 29.7057113 46.4130624 43.3169143

50 H 31.4246097 46.0933204 43.0806909

51 H 29.5056766 43.8547475 43.9653903

52 H 31.1715213 43.6968264 43.4632732

53 H 30.5376721 44.4687416 41.1222091

54 H 29.8746052 42.9246139 41.6351250

55 H 28.3376705 45.5986082 41.4125345

56 H 28.1893495 44.1546153 40.3923459

57 H 27.3486828 44.4578429 43.2449822

58 H 26.3879120 44.0327831 41.9399295

59 H 27.5070827 42.9762818 42.5529412

60 H 29.2926444 46.9815585 46.1082683

61 H 28.7107973 45.5496604 45.4530839

62 H 29.6631562 45.4910712 46.8299222

63 H 33.7560767 46.0409590 49.9649223

64 H 37.6326125 41.4017577 50.3177309

BesD3-TS1

QM(B1)/MM Energy = -3361.941878440799 a.u.

QM(B2)/MM Energy = -3364.070458000000 a.u.

QM(B3)/MM Energy = -3363.554941467896 a.u.

1 N 35.4786516 45.6589194 47.6813071

2 C 34.1519824 45.6540993 48.0522451

3 C 33.5484973 46.4837047 49.1366876

4 N 34.5025076 44.1611441 46.4227128

5 C 33.5612432 44.7073395 47.2523965

6 C 35.6567328 44.7431842 46.7067541

7 H 32.4736791 46.5591311 48.9201393

8 H 33.9514597 47.5067866 49.1483869

9 H 32.5325755 44.3648386 47.2408759

10 H 36.6058824 44.5328159 46.2201622

11 H 36.1996093 46.2646405 48.0864909

12 N 34.9319363 40.4620647 48.8815106

13 C 36.2190693 40.8475699 48.5492020

14 C 37.4148069 40.6613973 49.4275183

15 N 34.7954446 41.3771613 46.8781066

16 C 36.1061253 41.4064366 47.2962770

17 C 34.1132989 40.8013006 47.8569891

18 H 37.3502725 39.7195329 49.9957283

19 H 38.3216785 40.5779782 48.8102067

20 H 36.8895490 41.8265787 46.6724102

21 H 33.0400378 40.6264574 47.8450896

22 H 34.6514019 39.9784764 49.7430902

23 Cl 32.0778752 41.3831176 45.4578942

24 Fe 34.0394384 42.6231158 45.0707329

25 O 33.1335346 43.6814445 43.9933757

26 O 35.8314029 41.1628768 42.5906317

27 C 36.4096724 41.6576707 43.5368397

28 O 35.8336403 42.4794435 44.4005367

29 C 37.8576820 41.2893312 43.8545291

30 C 38.8835625 42.4363904 43.8580767

31 C 39.2329145 42.9283951 42.4376188

32 O 40.2838813 42.4868983 41.9070253

33 O 38.4219981 43.7271256 41.9026825

34 H 37.8520599 40.8069706 44.8468073

35 H 38.1610801 40.5201112 43.1313642

36 H 39.8053462 42.0791077 44.3420716

37 H 38.4858201 43.2844208 44.4426040

38 N 29.5958906 46.0006729 45.9912565

39 C 30.8958972 46.0210886 45.2324685

40 C 31.6088334 47.3681027 45.5305578

41 O 30.9613126 48.2166711 46.1837848

42 C 30.7093225 45.7462220 43.7352447

43 C 30.6880634 44.2585325 43.4017368

44 C 30.2668420 43.8686528 41.9864422

45 C 28.8768855 44.3401449 41.5010056

46 N 27.7406352 43.8857409 42.3690235

47 O 32.7745451 47.4861819 45.0921522

48 H 31.5339395 45.2272291 45.6383085

49 H 29.7944040 46.2583173 43.3815803

50 H 31.5448270 46.2287691 43.2046728

51 H 30.1647691 43.6578954 44.1660248

52 H 31.8861199 43.8654407 43.6112129

53 H 30.9842780 44.2840728 41.2575435
54 H 30.3357633 42.7727590 41.8825236
55 H 28.8001162 45.4354823 41.4335963
56 H 28.6919359 43.9365471 40.4943529
57 H 27.6734582 44.4023924 43.2722682
58 H 26.7941732 43.9747711 41.9233974
59 H 27.8283275 42.8910393 42.6466715
60 H 29.3210582 46.9971824 46.1735394
61 H 28.7939733 45.5598889 45.4872767
62 H 29.6953480 45.5072100 46.8943781
63 H 33.6953643 46.0726355 50.1354465
64 H 37.5642040 41.4409603 50.1745319

BesD3-IM1

QM(B1)/MM Energy = -3361.979005939164 a.u.

QM(B2)/MM Energy = -3364.109970000000 a.u.

QM(B3)/MM Energy = -3363.587976334019 a.u.

1 N 35.5925176 45.6377447 47.6619455
2 C 34.2541383 45.6158450 47.9921423
3 C 33.6186244 46.4613697 49.0467934
4 N 34.6870368 44.0891235 46.4146160
5 C 33.7041342 44.6408408 47.1957208
6 C 35.8204283 44.7020260 46.7194837
7 H 32.5509882 46.5426509 48.7975304
8 H 34.0291597 47.4816672 49.0567845
9 H 32.6788857 44.2847430 47.1517153
10 H 36.7890983 44.4952271 46.2704740
11 H 36.2865743 46.2671826 48.0771928
12 N 35.0008474 40.4259465 48.9600790
13 C 36.3060475 40.7557059 48.6366690
14 C 37.4839750 40.6072181 49.5422054
15 N 34.9356364 41.2395872 46.9130750
16 C 36.2391306 41.2485577 47.3556339
17 C 34.2133953 40.7368338 47.9043558
18 H 37.4221008 39.6838766 50.1401455
19 H 38.3988113 40.5132683 48.9393937
20 H 37.0439371 41.6118291 46.7238160
21 H 33.1340827 40.6040487 47.8826809
22 H 34.6898933 39.9790355 49.8312727
23 Cl 32.1959638 41.4973400 45.4662300
24 Fe 34.3597854 42.4813541 45.1189162
25 O 33.5839945 43.4498982 43.7496666
26 O 36.0589173 41.1611193 42.6130418
27 C 36.6967562 41.5341641 43.5790661
28 O 36.1879744 42.2579221 44.5599691
29 C 38.1480318 41.1287805 43.7911702
30 C 39.1567366 42.2890898 43.8196346
31 C 39.4194622 42.8473218 42.4087837
32 O 40.4113751 42.3891143 41.7843773
33 O 38.6060738 43.6945298 41.9646278
34 H 38.1908191 40.5795302 44.7466874
35 H 38.4161941 40.4198935 42.9980604
36 H 40.1078956 41.9236245 44.2355627
37 H 38.7725584 43.0931365 44.4695385
38 N 29.5600981 45.9760726 45.8334422
39 C 30.8581278 45.9627111 45.0726755
40 C 31.6330314 47.2707885 45.4134677
41 O 31.0000718 48.1435644 46.0521920
42 C 30.6635409 45.7678274 43.5634379
43 C 30.1538256 44.4128610 43.1779410
44 C 29.7341021 44.0747699 41.7757077
45 C 28.3040212 44.4772606 41.3714154
46 N 27.2984799 43.9373406 42.3356818
47 O 32.8155192 47.3464495 45.0138124
48 H 31.4487804 45.1116804 45.4398898

49 H 30.0375553 46.5782853 43.1437140
50 H 31.6712298 45.9208801 43.1259696
51 H 30.2938661 43.5892524 43.8879615
52 H 32.7367433 43.0996447 43.4379467
53 H 30.4019922 44.5687977 41.0445147
54 H 29.8503931 42.9897313 41.6121801
55 H 28.1730605 45.5677399 41.3417888
56 H 28.0658584 44.0787302 40.3745770
57 H 27.3299545 44.4267678 43.2553510
58 H 26.3083708 43.9777248 41.9969130
59 H 27.4747446 42.9431295 42.5684528
60 H 29.2764188 46.9721842 45.9943505
61 H 28.7574816 45.5099101 45.3521455
62 H 29.6716733 45.5162771 46.7519338
63 H 33.7371019 46.0687906 50.0567003
64 H 37.6122220 41.4155184 50.2621155

BesD3-TS-Cl

QM(B1)/MM Energy = -3361.959250592991 a.u.

QM(B2)/MM Energy = -3364.083992000000 a.u.

QM(B3)/MM Energy = -3363.568550949495 a.u.

1 N 35.4238597 45.8293807 47.5957621
2 C 34.1310636 45.6824477 48.0478142
3 C 33.5089625 46.4686945 49.1543863
4 N 34.5163654 44.2737945 46.3461422
5 C 33.5909651 44.6948753 47.2605962
6 C 35.6215828 44.9612884 46.5790192
7 H 32.4298705 46.5185173 48.9491555
8 H 33.8818030 47.5027457 49.1797009
9 H 32.6065164 44.2419455 47.3163651
10 H 36.5562243 44.8333239 46.0366820
11 H 36.1143121 46.4735481 47.9927474
12 N 34.7485131 40.5701492 48.8480290
13 C 36.0505964 40.8963025 48.5106246
14 C 37.2545593 40.6678206 49.3715483
15 N 34.6536578 41.4527229 46.8178555
16 C 35.9628625 41.4340949 47.2469793
17 C 33.9485892 40.9269277 47.8072930
18 H 37.1697156 39.7229332 49.9330577
19 H 38.1444559 40.5595627 48.7330424
20 H 36.7643600 41.8269708 46.6274103
21 H 32.8672059 40.8031164 47.8171155
22 H 34.4533891 40.1067567 49.7147819
23 Cl 31.6867116 42.1896971 44.9421117
24 Fe 34.2332962 42.7617518 44.8690529
25 O 33.6328069 43.7940692 43.3843483
26 O 35.2269469 41.2547902 43.6848172
27 C 36.3560066 41.8098511 43.8583579
28 O 36.4314417 42.8500401 44.5755562
29 C 37.5913353 41.1492279 43.2858769
30 C 38.9073119 41.9260725 43.4053368
31 C 39.2843494 42.7077230 42.1391462
32 O 40.4038666 42.4535144 41.6234878
33 O 38.4481351 43.5398740 41.6983533
34 H 37.6751759 40.1910753 43.8279453
35 H 37.3628581 40.8742653 42.2434104
36 H 39.7329911 41.2423579 43.6446415
37 H 38.8125356 42.6454251 44.2356553
38 N 29.5706175 46.0497666 45.9766243
39 C 30.9376030 45.8322665 45.3680650
40 C 31.7017779 47.1972889 45.3939252
41 O 31.0771031 48.1542955 45.9205667
42 C 30.9276653 45.1603888 43.9993310
43 C 30.1968677 43.8681185 43.8981574
44 C 29.9254902 43.3073349 42.5294988

45 C 28.7206433 43.9368752 41.7887057

46 N 27.4419164 43.7314571 42.5357542

47 O 32.8577753 47.2198190 44.9294653

48 H 31.4646418 45.1617234 46.0539767

49 H 30.5399478 45.8535691 43.2224601

50 H 31.9924162 44.9626430 43.7189566

51 H 29.4835622 43.6014513 44.6798454

52 H 33.2356794 43.1974680 42.7339423

53 H 30.8062359 43.4587337 41.8854479

54 H 29.7702655 42.2204361 42.5926246

55 H 28.8344906 45.0194277 41.6295031

56 H 28.6096734 43.4606241 40.8034869

57 H 27.3836879 44.3186989 43.3943653

58 H 26.5605825 43.8981260 41.9922562

59 H 27.3650390 42.7548603 42.8755546

60 H 29.4312692 47.0850408 46.0783359

61 H 28.7530294 45.6731513 45.4469361

62 H 29.5262782 45.6168742 46.9112430

63 H 33.6735074 46.0547323 50.1491865

64 H 37.4588868 41.4297933 50.1236886

BesD3-PC-Cl

QM(B1)/MM Energy = -3361.995199264264 a.u.

QM(B2)/MM Energy = -3364.125377000000 a.u.

QM(B3)/MM Energy = -3363.593109682247 a.u.

1 N 35.6921548 45.9252204 47.8289541

2 C 34.3515469 45.7914884 48.1157943

3 C 33.6201756 46.5592716 49.1661594

4 N 34.9661359 44.2831630 46.5762021

5 C 33.9200323 44.7623354 47.3148071

6 C 36.0251513 44.9968643 46.9046924

7 H 32.5576712 46.5737816 48.8837046

8 H 33.9578707 47.6043926 49.2256206

9 H 32.9214067 44.3386740 47.2397512

10 H 37.0220279 44.8739280 46.4854249

11 H 36.3085282 46.6323394 48.2342140

12 N 34.9022781 40.5248454 48.9615745

13 C 36.2141383 40.8232878 48.6411386

14 C 37.3978228 40.6295377 49.5326412

15 N 34.8573096 41.3784545 46.9183150

16 C 36.1599190 41.3384544 47.3683193

17 C 34.1259967 40.8795431 47.9028966

18 H 37.3195003 39.6974288 50.1153552

19 H 38.3030437 40.5237102 48.9167945

20 H 36.9790310 41.7041350 46.7532049

21 H 33.0430156 40.7638055 47.9037630

22 H 34.5826896 40.0748240 49.8267871

23 Cl 30.8140676 42.5809287 43.9389735

24 Fe 34.7341982 42.7664936 45.1045683

25 O 33.5548169 43.7800331 44.0297494

26 O 35.5101869 41.2532006 43.6543614

27 C 36.6670902 41.6982993 43.8838858

28 O 36.8307049 42.6857722 44.6739264

29 C 37.8698280 40.9845025 43.3116532

30 C 39.1907149 41.7517146 43.3859125

31 C 39.4532705 42.5966734 42.1354057

32 O 40.5208990 42.3663039 41.5102611

33 O 38.5802556 43.4420397 41.8017760

34 H 37.9474061 40.0447994 43.8864694

35 H 37.6191479 40.6854274 42.2825005

36 H 40.0347586 41.0618789 43.5209873

37 H 39.1510485 42.4181953 44.2627137

38 N 29.6201941 45.8993558 45.8379030

39 C 30.9169514 45.7523431 45.0724524

40 C 31.6988121 47.1026810 45.2715828

41 O 31.0617661 48.0040095 45.8839741

42 C 30.7393845 45.3721961 43.5984212

43 C 29.9632114 44.0795811 43.3171873

44 C 29.5944021 43.8655817 41.8369936

45 C 28.2709076 44.5088262 41.4105209

46 N 27.1518864 44.0621653 42.3064675

47 O 32.8576883 47.1812915 44.8316468

48 H 31.5040959 44.9511299 45.5413213

49 H 30.2267022 46.1875172 43.0547135

50 H 31.7628269 45.2826769 43.2023682

51 H 29.0565132 44.0443038 43.9191490

52 H 33.0510821 43.2151251 43.4305075

53 H 30.3941682 44.2485546 41.1829840

54 H 29.5310891 42.7836727 41.6448185

55 H 28.2895097 45.6076237 41.4342317

56 H 28.0238852 44.2001259 40.3849095

57 H 27.1227222 44.5865060 43.2063917

58 H 26.1924407 44.1151943 41.8902444

59 H 27.2634442 43.0707126 42.5897412

60 H 29.5537109 46.9291378 46.0502991

61 H 28.7391850 45.6141630 45.3563593

62 H 29.6743739 45.3806007 46.7303388

63 H 33.7437110 46.1367974 50.1633161

64 H 37.5614615 41.4190709 50.2660762

BesD3-TS-OH

QM(B1)/MM Energy = -3361.960911923859 a.u.

QM(B2)/MM Energy = -3364.086258000000 a.u.

QM(B3)/MM Energy = -3363.566124216978 a.u.

1 N 35.3528890 45.7359520 47.6652906

2 C 34.0415291 45.6782754 48.0805636

3 C 33.4579277 46.4787348 49.1972253

4 N 34.3846064 44.2224929 46.4075939

5 C 33.4590467 44.7192030 47.2856292

6 C 35.5258317 44.8408578 46.6683464

7 H 32.3753727 46.5417869 49.0177905

8 H 33.8443723 47.5081484 49.2165984

9 H 32.4481060 44.3292542 47.3107294

10 H 36.4623389 44.6574341 46.1486903

11 H 36.0732924 46.3426035 48.0686316

12 N 34.9430967 40.4856473 48.8193745

13 C 36.2385043 40.8580811 48.5065726

14 C 37.4157837 40.6624432 49.4085142

15 N 34.8453521 41.4025829 46.8119435

16 C 36.1517854 41.4185264 47.2517526

17 C 34.1428335 40.8342078 47.7827251

18 H 37.3317287 39.7215580 49.9764039

19 H 38.3337700 40.5711073 48.8090105

20 H 36.9448235 41.8483341 46.6468503

21 H 33.0663031 40.6832096 47.7582990

22 H 34.6459147 40.0092174 49.6785520

23 Cl 31.9507240 41.5554164 45.5702672

24 Fe 34.0515264 42.5935216 45.0839597

25 O 33.0915466 43.6140973 43.7369225

26 O 35.0420947 41.5029659 43.5245821

27 C 36.1825369 41.9811317 43.8218663

28 O 36.2570892 42.9395671 44.6369330

29 C 37.4316515 41.2875273 43.3172677

30 C 38.7587840 42.0379827 43.4876408

31 C 39.1774234 42.8559172 42.2576143

32 O 40.2813329 42.5675069 41.7282639

33 O 38.3782072 43.7431978 41.8552236

34 H 37.4812063 40.3651915 43.9208628

35 H 37.2569460 40.9535418 42.2828560

36 H 39.5649976 41.3286539 43.7180406

37 H 38.6544086 42.7239284 44.3446675

38 N 29.6107479 45.9508378 45.9767501

39 C 30.9251307 45.9937435 45.2413767

40 C 31.5956108 47.3658969 45.5381762

41 O 30.9443145 48.1763893 46.2320434

42 C 30.8098129 45.7117964 43.7416274

43 C 30.6866595 44.2662060 43.3835712

44 C 30.2398731 43.8431747 42.0131087

45 C 28.8521012 44.3344817 41.5225382

46 N 27.7142144 43.8835608 42.3836080

47 O 32.7343179 47.5409858 45.0493340

48 H 31.5717593 45.2197385 45.6703057

49 H 29.9971070 46.3179374 43.2941983

50 H 31.7376926 46.1077467 43.2916193

51 H 30.6867272 43.5171199 44.1799392

52 H 33.1750683 43.0853862 42.9232972

53 H 30.9458124 44.2368825 41.2565086

54 H 30.2868787 42.7448552 41.9334808

55 H 28.7916355 45.4310831 41.4595275

56 H 28.6736029 43.9360773 40.5127574

57 H 27.6455669 44.4018005 43.2859854

58 H 26.7693479 43.9728054 41.9345246

59 H 27.7987828 42.8894872 42.6665817

60 H 29.3388608 46.9426717 46.1860323

61 H 28.8102539 45.5254387 45.4576910

62 H 29.6989984 45.4338855 46.8692616

63 H 33.6405384 46.0570303 50.1856006

64 H 37.5585248 41.4402202 50.1586843

BesD3-PC-OH

QM(B1)/MM Energy = -3362.071069978969 a.u.

QM(B2)/MM Energy = -3364.195174000000 a.u.

QM(B3)/MM Energy = -3363.659617616420 a.u.

1 N 35.5175236 45.5757355 47.6186126

2 C 34.1772408 45.5691310 47.9461030

3 C 33.5607042 46.4242473 49.0051312

4 N 34.5951682 44.0359461 46.3600520

5 C 33.6188405 44.6011365 47.1449000

6 C 35.7347683 44.6382776 46.6752135

7 H 32.4885696 46.5086356 48.7750824

8 H 33.9733657 47.4439930 48.9980259

9 H 32.5899460 44.2559663 47.0940711

10 H 36.6989848 44.4070745 46.2290000

11 H 36.2189737 46.1980912 48.0321463

12 N 35.0398305 40.3626995 49.0888621

13 C 36.3506289 40.6564064 48.7533405

14 C 37.5280204 40.5555697 49.6621340

15 N 34.9932095 41.0786899 47.0110485

16 C 36.2972653 41.0884971 47.4516122

17 C 34.2580174 40.6378117 48.0217448

18 H 37.4873239 39.6494785 50.2872604

19 H 38.4406897 40.4626470 49.0571100

20 H 37.1056298 41.4269259 46.8082830

21 H 33.1746068 40.5347684 48.0155413

22 H 34.7180229 39.9546163 49.9747197

23 Cl 32.0887467 41.7364616 45.3179025

24 Fe 34.4325810 42.2097335 45.3150437

25 O 31.7747901 44.0360306 43.2203917

26 O 35.1975541 41.5953826 43.5106739

27 C 36.4189780 41.8417213 43.8124318

28 O 36.6823912 42.5528070 44.8131739

29 C 37.5108212 41.1290915 43.0540870

30 C 38.9165442 41.6966321 43.2598188

31 C 39.3410268 42.6695546 42.1558542

32 O 40.4350640 42.4344967 41.5809146

33 O	38.5604373	43.6247125	41.8979638
34 H	37.4723047	40.0952413	43.4393856
35 H	37.2218352	41.0590119	41.9935389
36 H	39.6551248	40.8896088	43.3342368
37 H	38.9297362	42.2383046	44.2198283
38 N	29.5703409	46.0128418	45.9630718
39 C	30.7984762	46.0676737	45.0933319
40 C	31.5804550	47.3619830	45.4483416
41 O	30.9711341	48.2086706	46.1433182
42 C	30.4351999	45.9495280	43.6048278
43 C	30.4336244	44.4816083	43.1475755
44 C	29.9132042	44.2453116	41.7159365
45 C	28.4766928	44.6416985	41.3743704
46 N	27.4673139	44.0409039	42.3079774
47 O	32.7467206	47.4565892	45.0044497
48 H	31.4407488	45.2143228	45.3479174
49 H	29.4752632	46.4529577	43.4096448
50 H	31.2073427	46.4731891	43.0208424
51 H	29.8112465	43.8925332	43.8514876
52 H	31.8123209	43.1816083	43.6926451
53 H	30.5737423	44.7879260	41.0194584
54 H	30.0622715	43.1738990	41.4968213
55 H	28.3209614	45.7290985	41.3940792
56 H	28.2295415	44.2910797	40.3621016
57 H	27.4437649	44.5080227	43.2391118
58 H	26.4838892	44.0593187	41.9464015
59 H	27.6675937	43.0479903	42.5258004
60 H	29.3062772	47.0024893	46.1836113
61 H	28.7395321	45.5758488	45.5055364
62 H	29.7463827	45.4995187	46.8436751
63 H	33.6953100	46.0462514	50.0185747
64 H	37.6375783	41.3903262	50.3544098

BesD3-Iso1-TS

QM(B1)/MM Energy = -3361.968646563775 a.u.

QM(B2)/MM Energy = -3364.099643000000 a.u.

QM(B3)/MM Energy = -3363.577337524452 a.u.

1 N	35.6456042	45.5226717	47.6126545
2 C	34.3036491	45.5321055	47.9337345
3 C	33.6743763	46.4170924	48.9609467
4 N	34.7179551	43.9672013	46.3847408
5 C	33.7395660	44.5537457	47.1532071
6 C	35.8624490	44.5686437	46.6880646
7 H	32.6125830	46.5246172	48.6950810
8 H	34.1121469	47.4261957	48.9542173
9 H	32.7111167	44.2046679	47.1123242
10 H	36.8275921	44.3363566	46.2442017
11 H	36.3485017	46.1419017	48.0287194
12 N	35.0190201	40.3941279	48.9194864
13 C	36.3318676	40.6873285	48.5878593
14 C	37.5043296	40.5755176	49.5061191
15 N	34.9793812	41.0808408	46.8261770
16 C	36.2820646	41.0986503	47.2785114
17 C	34.2420800	40.6523773	47.8444611
18 H	37.4541454	39.6589301	50.1159678
19 H	38.4231295	40.4852432	48.9089746
20 H	37.0927698	41.4247478	46.6353848
21 H	33.1581584	40.5682370	47.8355195
22 H	34.6960563	39.9927814	49.8083807
23 Cl	32.0949517	41.8985393	45.7543695
24 Fe	34.4444011	42.3350640	45.0867895
25 O	33.7981354	42.2675400	43.3902571
26 O	36.3869029	41.3115160	42.5433353
27 C	36.9046101	41.6033974	43.6013026
28 O	36.3161211	42.2949860	44.5753816

29 C 38.3270171 41.1739283 43.9297464
30 C 39.3361447 42.3313998 43.8838751
31 C 39.5238067 42.8521332 42.4466478
32 O 40.4646125 42.3562112 41.7740648
33 O 38.7039283 43.7083044 42.0340547
34 H 38.3339796 40.7112549 44.9296724
35 H 38.6171992 40.4039538 43.2040675
36 H 40.3053323 41.9773255 44.2674446
37 H 38.9792319 43.1516397 44.5296167
38 N 29.5076598 45.9699992 45.8682885
39 C 30.7958934 45.9513691 45.0921672
40 C 31.5971254 47.2431210 45.4317148
41 O 30.9855942 48.1291151 46.0710117
42 C 30.5689686 45.7799535 43.5833658
43 C 30.1232822 44.4068509 43.1833709
44 C 29.7053680 44.0688384 41.7810161
45 C 28.2753726 44.4680860 41.3760554
46 N 27.2733469 43.9321052 42.3453758
47 O 32.7802260 47.2947683 45.0245832
48 H 31.3706132 45.0848189 45.4493149
49 H 29.8844944 46.5641191 43.2081621
50 H 31.5458883 46.0105339 43.1092789
51 H 30.3348537 43.5830074 43.8745880
52 H 32.8608527 42.0400977 43.3029164
53 H 30.3735373 44.5630097 41.0490572
54 H 29.8251602 42.9843709 41.6180382
55 H 28.1424704 45.5580864 41.3400765
56 H 28.0357809 44.0643608 40.3817880
57 H 27.3097500 44.4205464 43.2653923
58 H 26.2818521 43.9733091 42.0114222
59 H 27.4456308 42.9373471 42.5772897
60 H 29.2193765 46.9646943 46.0251412
61 H 28.7043470 45.4988296 45.3929811
62 H 29.6292354 45.5102283 46.7864789
63 H 33.7667230 46.0458672 49.9815989
64 H 37.6223295 41.3921578 50.2183283

BesD3-Iso1-IM

QM(B1)/MM Energy = -3361.972801999819 a.u.

QM(B2)/MM Energy = -3364.106103000000 a.u.

QM(B3)/MM Energy = -3363.583329651918 a.u.

1 N 35.5908557 45.4408792 47.5156884
2 C 34.2552029 45.4595032 47.8618972
3 C 33.6487411 46.3657951 48.8858236
4 N 34.6279462 43.8693001 46.3150788
5 C 33.6738530 44.4743461 47.0998645
6 C 35.7790396 44.4720502 46.5944126
7 H 32.5867423 46.4925109 48.6266329
8 H 34.1047913 47.3669031 48.8631279
9 H 32.6401262 44.1424263 47.0764212
10 H 36.7347031 44.2285841 46.1362192
11 H 36.3056182 46.0581501 47.9152535
12 N 35.0632170 40.3676766 49.1696359
13 C 36.3737052 40.6247201 48.8031468
14 C 37.5613378 40.5437473 49.6996443
15 N 34.9959213 40.9974602 47.0754317
16 C 36.3090685 41.0049963 47.4865025
17 C 34.2651378 40.6122227 48.1115541
18 H 37.5365303 39.6436216 50.3340410
19 H 38.4680700 40.4534107 49.0860160
20 H 37.1084135 41.2955663 46.8101332
21 H 33.1801382 40.5343786 48.1289047
22 H 34.7462981 39.9860786 50.0690102
23 Cl 32.0841336 42.0688146 45.5436658
24 Fe 34.4728097 41.9140690 45.2870251

25 O	34.2337827	40.6084239	44.0389609
26 O	36.0561375	42.4583250	42.5107191
27 C	36.6709018	42.0243456	43.4805320
28 O	36.3039320	42.2916176	44.7111238
29 C	37.8846127	41.1279319	43.3537467
30 C	39.2121730	41.8214334	43.7094884
31 C	39.6208141	42.7846954	42.5844856
32 O	40.4861803	42.3656744	41.7667919
33 O	39.0355486	43.8884850	42.5387801
34 H	37.7279571	40.2606632	44.0147705
35 H	37.9301240	40.7699728	42.3164700
36 H	39.9934293	41.0582351	43.8312316
37 H	39.1084054	42.3714055	44.6596235
38 N	29.5028879	45.9755334	45.8509484
39 C	30.7855319	45.9731788	45.0655263
40 C	31.5824746	47.2627115	45.4196628
41 O	30.9708260	48.1363692	46.0755812
42 C	30.5458953	45.8263716	43.5558106
43 C	30.1088048	44.4561012	43.1372312
44 C	29.6841062	44.1296121	41.7343250
45 C	28.2393629	44.4957741	41.3517944
46 N	27.2635503	43.9369971	42.3348024
47 O	32.7622494	47.3258655	45.0052703
48 H	31.3681712	45.1044920	45.4039920
49 H	29.8515914	46.6107975	43.2006561
50 H	31.5162706	46.0756706	43.0775525
51 H	30.3387005	43.6237686	43.8112919
52 H	33.3619675	40.4675864	43.6444607
53 H	30.3289901	44.6518702	41.0010124
54 H	29.8298292	43.0507162	41.5557118
55 H	28.0831200	45.5823253	41.3205600
56 H	27.9938603	44.0891447	40.3603734
57 H	27.3044271	44.4242043	43.2554259
58 H	26.2665263	43.9626725	42.0161500
59 H	27.4573416	42.9453399	42.5624252
60 H	29.2125062	46.9675674	46.0209571
61 H	28.6986346	45.5064045	45.3756199
62 H	29.6311638	45.5057113	46.7628145
63 H	33.7371074	46.0119542	49.9129824
64 H	37.6659235	41.3881456	50.3809082

BesD3-Iso1-TS-Cl

QM(B1)/MM Energy = -3361.959473547638 a.u.

QM(B2)/MM Energy = -3364.093322000000 a.u.

QM(B3)/MM Energy = -3363.574584428219 a.u.

1 N	35.5891417	45.4794978	47.6241660
2 C	34.2491945	45.5390855	47.9425120
3 C	33.6470692	46.4317690	48.9797700
4 N	34.6009597	43.9639208	46.3787199
5 C	33.6530200	44.5891446	47.1475815
6 C	35.7637595	44.5169915	46.6915334
7 H	32.5819035	46.5475086	48.7306500
8 H	34.0916030	47.4381400	48.9648598
9 H	32.6033383	44.3213858	47.0823210
10 H	36.7217200	44.2555523	46.2472022
11 H	36.3127535	46.0790893	48.0333111
12 N	34.8792449	40.4222344	49.1641585
13 C	36.1601257	40.7347460	48.7452905
14 C	37.4001765	40.5953095	49.5673032
15 N	34.6723052	41.2044387	47.1192975
16 C	36.0073105	41.2047548	47.4641729
17 C	34.0201861	40.7233678	48.1649398
18 H	37.3911441	39.6660991	50.1595475
19 H	38.2752833	40.5189603	48.9056754
20 H	36.7664523	41.5358308	46.7612654

21 H	32.9448830	40.5839275	48.2453383
22 H	34.6185743	39.9776242	50.0521230
23 Cl	32.4589924	43.5102799	44.1056832
24 Fe	34.2392383	42.1476385	45.2052680
25 O	33.3439424	40.7043637	44.4446966
26 O	35.8166738	42.3441410	42.4504698
27 C	36.4812892	41.9551520	43.4120634
28 O	36.1144509	42.1901660	44.6387456
29 C	37.7420582	41.1275583	43.2565223
30 C	39.0390723	41.8252928	43.7025680
31 C	39.5026861	42.8200004	42.6270148
32 O	40.4205831	42.4310509	41.8517907
33 O	38.9040745	43.9168885	42.5740103
34 H	37.5996825	40.2131402	43.8554148
35 H	37.8263954	40.8395829	42.1998575
36 H	39.8193641	41.0665161	43.8514164
37 H	38.8752031	42.3488179	44.6588893
38 N	29.4686904	46.1625324	46.0073278
39 C	30.7598784	46.1555452	45.2387477
40 C	31.5389018	47.4566886	45.5479363
41 O	30.9247551	48.3402996	46.1880725
42 C	30.5330718	45.9410654	43.7409246
43 C	30.2282006	44.5183486	43.3901771
44 C	29.9682205	44.0982497	41.9741339
45 C	28.5569264	44.4459855	41.4466818
46 N	27.4857451	43.9264824	42.3532193
47 O	32.7147894	47.5122483	45.1225243
48 H	31.3613719	45.3140846	45.6017950
49 H	29.7339825	46.6224586	43.3837730
50 H	31.4480898	46.2657099	43.2194578
51 H	29.7699431	43.8962147	44.1648977
52 H	33.3405254	40.8116010	43.4804135
53 H	30.6865354	44.5798733	41.2890455
54 H	30.1297960	43.0102532	41.8853969
55 H	28.4045796	45.5313817	41.3594380
56 H	28.3998504	44.0004746	40.4529605
57 H	27.4522620	44.4327748	43.2638247
58 H	26.5169368	43.9686275	41.9560788
59 H	27.6407877	42.9322775	42.6043653
60 H	29.1690545	47.1563810	46.1520585
61 H	28.6763846	45.6814339	45.5248007
62 H	29.5862167	45.7096380	46.9268308
63 H	33.7469479	46.0630235	50.0006119
64 H	37.5642867	41.3982069	50.2859761

BesD3-Iso1-PC-Cl

QM(B1)/MM Energy = -3362.006634058120 a.u.

QM(B2)/MM Energy = -3364.146666000000 a.u.

QM(B3)/MM Energy = -3363.613012685877 a.u.

1 N	35.5919974	45.4717169	47.5901232
2 C	34.2496572	45.5093805	47.8994218
3 C	33.6398459	46.4022807	48.9328221
4 N	34.6402433	43.9238150	46.3501929
5 C	33.6753535	44.5396193	47.1078253
6 C	35.7884218	44.5017708	46.6658142
7 H	32.5750633	46.5174603	48.6805316
8 H	34.0841491	47.4089271	48.9160722
9 H	32.6379085	44.2200282	47.0531547
10 H	36.7589619	44.2457106	46.2455585
11 H	36.3041418	46.0788213	48.0077359
12 N	34.9824694	40.3908054	49.2831031
13 C	36.2733451	40.6321425	48.8517713
14 C	37.5012409	40.5545565	49.6953791
15 N	34.8148287	40.9894273	47.1752417
16 C	36.1447528	40.9885435	47.5315354

17 C 34.1405851 40.6251456 48.2510724
18 H 37.5120170 39.6466463 50.3201363
19 H 38.3838226 40.4785834 49.0445962
20 H 36.9145030 41.2418455 46.8076518
21 H 33.0593324 40.5402806 48.3316523
22 H 34.6987122 40.0100637 50.1913736
23 Cl 32.0725639 44.0218526 42.7683836
24 Fe 34.2807023 42.0222453 45.3879132
25 O 32.4226679 41.7688974 45.1430586
26 O 35.6531638 42.4223674 42.6616208
27 C 36.4148410 41.8655439 43.4696032
28 O 36.1543275 41.7986643 44.7269974
29 C 37.6954684 41.1719165 43.0407529
30 C 38.9935039 41.8658850 43.5118492
31 C 39.5046658 42.8397537 42.4397223
32 O 40.4364595 42.4119958 41.6990640
33 O 38.9436265 43.9514443 42.3476971
34 H 37.6613591 40.1647097 43.4838798
35 H 37.6969305 41.0698722 41.9456522
36 H 39.7648830 41.1048032 43.6919133
37 H 38.8110360 42.3970932 44.4603054
38 N 29.5520111 45.9559839 45.9072856
39 C 30.7477888 46.0249566 44.9977274
40 C 31.5537983 47.3055782 45.3842981
41 O 30.9549456 48.1332654 46.1083705
42 C 30.3277202 45.9981300 43.5120363
43 C 30.3274001 44.5939353 42.8976933
44 C 29.7324347 44.4321914 41.4915932
45 C 28.2542216 44.7655736 41.2958777
46 N 27.3504619 44.0408087 42.2410358
47 O 32.7119858 47.3993803 44.9286624
48 H 31.3876076 45.1568605 45.2076772
49 H 29.3227736 46.4412858 43.4153706
50 H 31.0109031 46.6365516 42.9326540
51 H 29.8640959 43.8735871 43.5891113
52 H 32.1422266 42.3779020 44.4436161
53 H 30.2953095 45.0681074 40.7901328
54 H 29.9157295 43.3916639 41.1769836
55 H 28.0528924 45.8370754 41.4093075
56 H 27.9624856 44.4802865 40.2758571
57 H 27.3770132 44.4269961 43.2083402
58 H 26.3422847 44.0546964 41.9536559
59 H 27.5876224 43.0371745 42.3416107
60 H 29.2982568 46.9457373 46.1450395
61 H 28.7055086 45.5237343 45.4757582
62 H 29.7634818 45.4398849 46.7799877
63 H 33.7397138 46.0432606 49.9571258
64 H 37.6304588 41.3876366 50.3862825

BesD3-Iso2-TS
QM(B1)/MM Energy = -3361.965482109318 a.u.
QM(B2)/MM Energy = -3364.099969000000 a.u.
QM(B3)/MM Energy = -3363.576732874189 a.u.

1 N 35.6178827 45.4504938 47.6489928
2 C 34.2705959 45.4889842 47.9402805
3 C 33.6410702 46.3955181 48.9494145
4 N 34.6925190 43.8615389 46.4496146
5 C 33.7122339 44.4941601 47.1718506
6 C 35.8346015 44.4581454 46.7592769
7 H 32.5783351 46.5005627 48.6839828
8 H 34.0793239 47.4043708 48.9278273
9 H 32.6704304 44.1997829 47.0865579
10 H 36.8052967 44.2073722 46.3377034
11 H 36.3198623 46.0826347 48.0467168
12 N 35.1040050 40.3183034 49.1874687

13 C	36.3945616	40.6466909	48.8161135
14 C	37.5930133	40.5608525	49.6968110
15 N	34.9726256	41.0682646	47.1340775
16 C	36.2908346	41.1001944	47.5245895
17 C	34.2800480	40.5894459	48.1558523
18 H	37.5821038	39.6491355	50.3142074
19 H	38.4955150	40.4947778	49.0739742
20 H	37.0701807	41.4483490	46.8525192
21 H	33.2025952	40.4429689	48.1975893
22 H	34.8104482	39.8955181	50.0757142
23 Cl	32.4688013	42.8896828	44.4250691
24 Fe	34.4391439	41.9754297	45.3079641
25 O	34.1266123	40.3687296	44.4734769
26 O	35.9524292	42.2971573	42.4864022
27 C	36.6077806	41.9622903	43.4701588
28 O	36.2546671	42.3149364	44.6811332
29 C	37.8394980	41.0868560	43.3733853
30 C	39.1583109	41.7845173	43.7439400
31 C	39.5763299	42.7566045	42.6290319
32 O	40.4748319	42.3617798	41.8360284
33 O	38.9657844	43.8459897	42.5683945
34 H	37.6723425	40.2226388	44.0372229
35 H	37.9050804	40.7214504	42.3403339
36 H	39.9411929	41.0242015	43.8695047
37 H	39.0434528	42.3287965	44.6956541
38 N	29.4575558	46.0947347	45.8114931
39 C	30.7643131	46.0731792	45.0683849
40 C	31.5729427	47.3379834	45.4605425
41 O	30.9540430	48.2252315	46.0927324
42 C	30.5736174	45.9522380	43.5516643
43 C	29.9257878	44.6739710	43.1264669
44 C	29.6217013	44.3280307	41.7002667
45 C	28.1578870	44.5415115	41.2701000
46 N	27.2129889	43.9831313	42.2831520
47 O	32.7688233	47.3775903	45.0936185
48 H	31.3158600	45.1829381	45.4024615
49 H	30.0328223	46.8347786	43.1576935
50 H	31.5918129	46.0288267	43.1213201
51 H	29.7912500	43.8870355	43.8742695
52 H	33.7103346	40.4002778	43.5995520
53 H	30.2410240	44.9231483	41.0074167
54 H	29.8873500	43.2694059	41.5265521
55 H	27.9234050	45.6079247	41.1648904
56 H	27.9566486	44.0489987	40.3081389
57 H	27.2559185	44.4982766	43.1892158
58 H	26.2115532	43.9714844	41.9796770
59 H	27.4394035	43.0045941	42.5365391
60 H	29.1501013	47.0860705	45.9520506
61 H	28.6701650	45.6043654	45.3284686
62 H	29.5641403	45.6588704	46.7399953
63 H	33.7337733	46.0396576	49.9754928
64 H	37.6897308	41.3940178	50.3929061

BesD3-Iso2-IM

QM(B1)/MM Energy = -3361.982267158164 a.u.

QM(B2)/MM Energy = -3364.112375000000 a.u.

QM(B3)/MM Energy = -3363.589436390741 a.u.

1 N	35.4925826	45.4945448	47.6341627
2 C	34.1521953	45.5281429	47.9550697
3 C	33.5541337	46.4057969	49.0063524
4 N	34.5357444	43.9527340	46.4030180
5 C	33.5726051	44.5613249	47.1666394
6 C	35.6880086	44.5337058	46.7074618
7 H	32.4819435	46.5008414	48.7812407
8 H	33.9812423	47.4195486	48.9875545

9 H 32.5300130 44.2684312 47.1021075
10 H 36.6447902 44.2858413 46.2581137
11 H 36.2098487 46.1007191 48.0443792
12 N 35.0870856 40.4040657 48.9663124
13 C 36.3986521 40.7112417 48.6542561
14 C 37.5547749 40.5867133 49.5876272
15 N 35.0671886 41.1526706 46.8922353
16 C 36.3603295 41.1660083 47.3562189
17 C 34.3223956 40.6909170 47.8877321
18 H 37.4903403 39.6710149 50.1966517
19 H 38.4820116 40.4963223 49.0048837
20 H 37.1821781 41.5178413 46.7390674
21 H 33.2395257 40.5862912 47.8662202
22 H 34.7582033 39.9834359 49.8437342
23 Cl 32.9866456 43.6148164 43.6618625
24 Fe 34.2193828 42.2860122 45.0620977
25 O 32.7466251 41.1382780 45.4120027
26 O 35.2815578 41.1757736 43.6495396
27 C 36.4074599 41.7296546 43.8897694
28 O 36.4366633 42.6939561 44.6967715
29 C 37.6601826 41.1254420 43.3035597
30 C 38.9615300 41.9212672 43.4752929
31 C 39.3472296 42.7514714 42.2411793
32 O 40.4157806 42.4369437 41.6560061
33 O 38.5665502 43.6741365 41.8949822
34 H 37.7607110 40.1557945 43.8211996
35 H 37.4532291 40.8709100 42.2518625
36 H 39.7885368 41.2338383 43.6972216
37 H 38.8456148 42.6036801 44.3326685
38 N 29.4921411 46.1233593 45.8085336
39 C 30.7884225 46.1172761 45.0448574
40 C 31.6013967 47.3787591 45.4386537
41 O 30.9845246 48.2622700 46.0803406
42 C 30.5661203 46.0086936 43.5330553
43 C 29.9708690 44.7020044 43.1159464
44 C 29.6537792 44.3509849 41.6938984
45 C 28.1878139 44.5680279 41.2761834
46 N 27.2503230 44.0029921 42.2926822
47 O 32.7954581 47.4203454 45.0667985
48 H 31.3535248 45.2281607 45.3561997
49 H 29.9748452 46.8690535 43.1661311
50 H 31.5690094 46.1271223 43.0796781
51 H 29.9294989 43.8943302 43.8530982
52 H 33.2014102 40.3191723 45.6487959
53 H 30.2693715 44.9441661 40.9955201
54 H 29.9190138 43.2931585 41.5204347
55 H 27.9522693 45.6351234 41.1796885
56 H 27.9780553 44.0813540 40.3129802
57 H 27.3000409 44.5129080 43.2013094
58 H 26.2477771 43.9909098 41.9938907
59 H 27.4818557 43.0232121 42.5383603
60 H 29.1748569 47.1107746 45.9509593
61 H 28.7056277 45.6240619 45.3325459
62 H 29.6088803 45.6898350 46.7365228
63 H 33.6877433 46.0312537 50.0212086
64 H 37.6541632 41.4082130 50.2970829

BesD3-Iso2-TS-Cl

QM(B1)/MM Energy = -3361.972240081562 a.u.

QM(B2)/MM Energy = -3364.101190000000 a.u.

QM(B3)/MM Energy = -3363.581852721458 a.u.

1 N 35.4100082 45.5397643 47.6288980
2 C 34.0791824 45.5756328 47.9856809
3 C 33.5070274 46.4308711 49.0686331
4 N 34.4025303 44.0596298 46.3579646

5 C	33.4712089	44.6437964	47.1776097
6 C	35.5696032	44.6147825	46.6592926
7 H	32.4294578	46.5239124	48.8707945
8 H	33.9277127	47.4473842	49.0572692
9 H	32.4288816	44.3485532	47.1435262
10 H	36.5121137	44.3661789	46.1811756
11 H	36.1461799	46.1214088	48.0417034
12 N	35.0285316	40.4285931	48.9614227
13 C	36.3339232	40.7478354	48.6346035
14 C	37.5037587	40.6092293	49.5510418
15 N	34.9737731	41.2074438	46.8953577
16 C	36.2721332	41.2211899	47.3428894
17 C	34.2481892	40.7278105	47.8943731
18 H	37.4423736	39.6877883	50.1521063
19 H	38.4242771	40.5201782	48.9566045
20 H	37.0821216	41.5857444	46.7162263
21 H	33.1654518	40.6156617	47.8854880
22 H	34.7139290	39.9980337	49.8388220
23 Cl	32.6273984	43.7777651	43.6118148
24 Fe	34.0250449	42.3889038	45.0412149
25 O	32.5620272	41.2209412	45.4060088
26 O	35.1050443	41.4586328	43.5355988
27 C	36.2695736	41.9054457	43.8266325
28 O	36.3834254	42.8132146	44.6804139
29 C	37.4738354	41.2166807	43.2211386
30 C	38.8290326	41.9072503	43.4167731
31 C	39.2524740	42.7807997	42.2270231
32 O	40.3459735	42.4989379	41.6713414
33 O	38.4719000	43.7074426	41.8850335
34 H	37.4985228	40.2267492	43.7103418
35 H	37.2571645	41.0120594	42.1602979
36 H	39.6122694	41.1583474	43.5939836
37 H	38.7679622	42.5482779	44.3120439
38 N	29.5097835	46.1096433	46.0072268
39 C	30.7786637	46.1258918	45.1999382
40 C	31.5542944	47.4327212	45.5165734
41 O	30.9407137	48.2984619	46.1815537
42 C	30.5040119	45.9402856	43.7037387
43 C	30.3565157	44.5024501	43.2983251
44 C	30.0259739	44.1406139	41.8765847
45 C	28.6118935	44.5412731	41.3977624
46 N	27.5369572	44.0256726	42.3020542
47 O	32.7209498	47.5092362	45.0716448
48 H	31.4020836	45.2874817	45.5305708
49 H	29.6047138	46.5233885	43.4206168
50 H	31.3386246	46.3945638	43.1462927
51 H	29.9825230	43.8112960	44.0612909
52 H	33.0487065	40.4286658	45.6680687
53 H	30.7332181	44.6330450	41.1875726
54 H	30.1625082	43.0551943	41.7404886
55 H	28.4850135	45.6317670	41.3372427
56 H	28.4301323	44.1254022	40.3955799
57 H	27.4968404	44.5253592	43.2163176
58 H	26.5693040	44.0675226	41.9012438
59 H	27.6862970	43.0325973	42.5606094
60 H	29.2205929	47.1030264	46.1771528
61 H	28.7042628	45.6446625	45.5310958
62 H	29.6452928	45.6366907	46.9154843
63 H	33.6621209	46.0420053	50.0750110
64 H	37.6185300	41.4210691	50.2692415

BesD3-Iso2-PC-Cl

QM(B1)/MM Energy = -3362.009636350698 a.u.

QM(B2)/MM Energy = -3364.143070000000 a.u.

QM(B3)/MM Energy = -3363.609444158201 a.u.

1 N	35.4258415	45.5931036	47.6320978
2 C	34.0921444	45.6056595	47.9758591
3 C	33.5034353	46.4406017	49.0659144
4 N	34.4560928	44.1069268	46.3352471
5 C	33.5060653	44.6734079	47.1504593
6 C	35.6104862	44.6758956	46.6580880
7 H	32.4252287	46.5211015	48.8654374
8 H	33.9107752	47.4626652	49.0667295
9 H	32.4709335	44.3491676	47.1121906
10 H	36.5611468	44.4265769	46.1932169
11 H	36.1481619	46.1864405	48.0513240
12 N	34.9869143	40.4229263	49.0773253
13 C	36.2834900	40.7288537	48.7083365
14 C	37.4827725	40.5947785	49.5861889
15 N	34.8709723	41.2130640	47.0227114
16 C	36.1872614	41.2082059	47.4224425
17 C	34.1725851	40.7384817	48.0431895
18 H	37.4465655	39.6735259	50.1895300
19 H	38.3845823	40.5119497	48.9629171
20 H	36.9743705	41.5759967	46.7679337
21 H	33.0885716	40.6457750	48.0837118
22 H	34.6922127	39.9880546	49.9590559
23 Cl	32.0879951	43.8212560	43.0047956
24 Fe	34.1014894	42.2824402	45.2641410
25 O	32.3738141	41.4762846	45.5047177
26 O	35.2618524	42.0053792	43.6331380
27 C	36.5134816	42.1232192	43.9409639
28 O	36.9112025	42.8238503	44.8846317
29 C	37.4967121	41.2517617	43.1814232
30 C	38.9506910	41.7115447	43.2688637
31 C	39.3600405	42.6576710	42.1380149
32 O	40.4379856	42.4013953	41.5409740
33 O	38.5902072	43.6226845	41.8817926
34 H	37.4069412	40.2580262	43.6561997
35 H	37.1518065	41.1267391	42.1431360
36 H	39.6346565	40.8545231	43.2862068
37 H	39.0765043	42.2477621	44.2244621
38 N	29.5814826	45.9945224	45.9345757
39 C	30.7940215	46.0307322	45.0427211
40 C	31.5908468	47.3302630	45.3772843
41 O	30.9921016	48.1778313	46.0793471
42 C	30.4123965	45.9197045	43.5543278
43 C	30.3716327	44.4774471	43.0355831
44 C	29.8149515	44.2668334	41.6188714
45 C	28.3722258	44.6914999	41.3346197
46 N	27.3814991	44.0757885	42.2707762
47 O	32.7458028	47.4149805	44.9104620
48 H	31.4373604	45.1819285	45.3070275
49 H	29.4336618	46.4033853	43.3942690
50 H	31.1459585	46.4851946	42.9606665
51 H	29.8523465	43.8219903	43.7511102
52 H	32.6527133	40.6842321	45.9795823
53 H	30.4508945	44.8143087	40.9050760
54 H	29.9341201	43.1983269	41.3752598
55 H	28.2347910	45.7797952	41.3831703
56 H	28.1077162	44.3697926	40.3172162
57 H	27.3784622	44.5206521	43.2128665
58 H	26.3909315	44.1010480	41.9288009
59 H	27.5765109	43.0749306	42.4589965
60 H	29.3440146	46.9909362	46.1608364
61 H	28.7317787	45.5753052	45.4953853
62 H	29.7655809	45.4814582	46.8145925
63 H	33.6629660	46.0479523	50.0701273
64 H	37.6114832	41.4093440	50.2989188

BesD3-Iso2-TS-OH

QM(B1)/MM Energy = -3361.940622580306 a.u.

QM(B2)/MM Energy = -3364.065723000000 a.u.

QM(B3)/MM Energy = -3363.548718208297 a.u.

1 N	35.3601183	45.7792534	47.4481776
2 C	34.0851754	45.6661536	47.9574410
3 C	33.5181353	46.4522864	49.0941797
4 N	34.3233366	44.3206383	46.1701273
5 C	33.4696287	44.7404839	47.1535811
6 C	35.4695106	44.9516039	46.3832450
7 H	32.4372685	46.5368570	48.9109308
8 H	33.9203977	47.4753436	49.1198306
9 H	32.4706209	44.3383715	47.2571572
10 H	36.3702888	44.7986905	45.7936890
11 H	36.0989800	46.3659535	47.8503680
12 N	34.6457891	40.5583377	48.8653492
13 C	35.9116430	40.9491478	48.4684861
14 C	37.1659457	40.7101862	49.2548340
15 N	34.3979692	41.5043901	46.8728045
16 C	35.7285042	41.5275767	47.2307917
17 C	33.7766598	40.9163999	47.8830532
18 H	37.1209519	39.7419669	49.7805626
19 H	38.0216771	40.6387798	48.5659165
20 H	36.4794914	41.9692539	46.5814397
21 H	32.7070260	40.7309221	47.9592157
22 H	34.4126525	40.0608069	49.7327941
23 Cl	33.4417931	43.7082511	42.6118283
24 Fe	33.8848283	42.7544513	44.7315616
25 O	31.8718785	42.6281904	44.9403169
26 O	35.0372329	41.3049638	43.8074683
27 C	36.1700530	41.9061256	43.8773632
28 O	36.2541628	42.9896732	44.4991909
29 C	37.3744546	41.2323079	43.2517486
30 C	38.7240953	41.9500732	43.4008969
31 C	39.1871947	42.7474132	42.1691661
32 O	40.3348647	42.4869310	41.7187827
33 O	38.3972964	43.6087790	41.7042763
34 H	37.4338404	40.2411993	43.7320631
35 H	37.1243973	41.0306272	42.1962679
36 H	39.5088171	41.2230244	43.6501664
37 H	38.6508476	42.6616750	44.2407218
38 N	29.5260186	46.1777680	45.9817382
39 C	30.8816143	45.9707152	45.3537326
40 C	31.6686872	47.3180663	45.3853394
41 O	31.0710088	48.2787873	45.9312695
42 C	30.8291653	45.3328154	43.9760390
43 C	30.3088846	43.9360449	43.9037880
44 C	30.0459493	43.3663349	42.5370358
45 C	28.8168662	43.9556872	41.8022320
46 N	27.5363871	43.7213739	42.5409377
47 O	32.8189757	47.3175346	44.9015037
48 H	31.4217451	45.2842767	46.0104941
49 H	30.2460387	45.9737877	43.2802116
50 H	31.8571099	45.3326536	43.5707617
51 H	29.6617673	43.5857427	44.7120673
52 H	31.5814168	42.7815680	45.8521868
53 H	30.9251879	43.5513860	41.9018489
54 H	29.9281733	42.2758905	42.5867808
55 H	28.8984784	45.0395618	41.6336545
56 H	28.7204717	43.4718647	40.8194925
57 H	27.4699031	44.2844175	43.4144444
58 H	26.6559258	43.9013751	42.0000226
59 H	27.4588756	42.7337967	42.8467409
60 H	29.3201787	47.2047195	46.0299841
61 H	28.7232853	45.7258382	45.4873951
62 H	29.5065088	45.8065261	46.9410288

63 H 33.6790440 46.0310379 50.0865129

64 H 37.4034657 41.4392922 50.0294667

BesD3-Iso2-PC-OH

QM(B1)/MM Energy = -3362.042329576419 a.u.

QM(B2)/MM Energy = -3364.166121000000 a.u.

QM(B3)/MM Energy = -3363.631091054520 a.u.

1 N 35.6038561 45.7148097 47.6300749

2 C 34.2677871 45.6297995 47.9561639

3 C 33.5981048 46.4478974 49.0117775

4 N 34.7684740 44.1402887 46.3522202

5 C 33.7664525 44.6409371 47.1425661

6 C 35.8695826 44.8023525 46.6717426

7 H 32.5296726 46.5024883 48.7563248

8 H 33.9809792 47.4790892 49.0296620

9 H 32.7480252 44.2642048 47.0921777

10 H 36.8480933 44.6352541 46.2250635

11 H 36.2683398 46.3696483 48.0524476

12 N 34.9690312 40.4523648 48.9564139

13 C 36.2820755 40.7577292 48.6516581

14 C 37.4542230 40.6014855 49.5635025

15 N 34.9483377 41.2519519 46.8962397

16 C 36.2449748 41.2417455 47.3671960

17 C 34.2044516 40.7697060 47.8821535

18 H 37.3795257 39.6843059 50.1690278

19 H 38.3685098 40.4919837 48.9626625

20 H 37.0711933 41.6038889 46.7605135

21 H 33.1228657 40.6455669 47.8730848

22 H 34.6398559 40.0161247 49.8265668

23 Cl 33.4547158 43.2028705 43.2858478

24 Fe 34.8050137 42.4603608 45.0429936

25 O 30.6733431 42.9241642 44.2478596

26 O 35.7544593 40.9639926 43.7356817

27 C 36.8468452 41.5527437 43.9472557

28 O 36.8679665 42.6046902 44.6779734

29 C 38.1350820 40.9524232 43.4486286

30 C 39.3507456 41.8806334 43.4638580

31 C 39.4925933 42.6580359 42.1491797

32 O 40.4961825 42.3806195 41.4422781

33 O 38.5905118 43.4820365 41.8460329

34 H 38.3095226 40.0780968 44.1003231

35 H 37.9459928 40.5364163 42.4486021

36 H 40.2702520 41.2988816 43.6180692

37 H 39.2365641 42.5866472 44.3007900

38 N 29.4452803 45.9981818 45.9389578

39 C 30.7333305 45.7852056 45.1799546

40 C 31.5911969 47.0916746 45.3053770

41 O 31.0323470 48.0524266 45.8976455

42 C 30.4945833 45.3309330 43.7400766

43 C 29.8892979 43.9171864 43.6077423

44 C 29.5811312 43.5552372 42.1377570

45 C 28.3343983 44.2147294 41.5391570

46 N 27.1309169 43.9420844 42.3932908

47 O 32.7438218 47.0741774 44.8310316

48 H 31.2728701 44.9777836 45.6913554

49 H 29.8595640 46.0637247 43.2118282

50 H 31.4793118 45.3414067 43.2490132

51 H 28.9470745 43.8760963 44.1651182

52 H 31.5908718 42.9623976 43.8892080

53 H 30.4441722 43.7967423 41.4958215

54 H 29.4627136 42.4603985 42.0846700

55 H 28.4177545 45.3073485 41.4406426

56 H 28.1323992 43.8073097 40.5380762

57 H 27.0940828 44.5381699 43.2467684

58 H 26.2037032 44.0291644 41.9161580

59 H 27.1435559 42.9713995 42.7570487

60 H 29.3432683 47.0307351 46.0878443

61 H 28.5761913 45.6704612 45.4596798

62 H 29.4901650 45.5308197 46.8578398

63 H 33.7227031 46.0609453 50.0231184

64 H 37.5929335 41.4151580 50.2753758

Hydrox-RC

QM(B1)/MM Energy = -3127.287824488242 a.u.

QM(B2)/MM Energy = -3129.527547000000 a.u.

QM(B3)/MM Energy = -3128.961708046254 a.u.

1 C 36.8816101 38.5076226 46.4725099

2 H 37.4530064 39.3822401 46.1299491

3 H 37.5809060 37.8783749 47.0428177

4 C 36.3403030 37.7800317 45.2809416

5 N 35.9170062 36.4669763 45.3052523

6 H 35.9451754 35.8494803 46.1238760

7 C 35.4297161 36.1489233 44.0869563

8 H 35.0187309 35.1787419 43.8210291

9 N 35.5345148 37.1857159 43.2689362

10 C 36.0953361 38.2064330 43.9936039

11 H 36.2761619 39.1747692 43.5397370

12 C 34.6707979 41.5868965 40.1798611

13 H 33.8137600 42.1469925 39.7794744

14 H 35.4326335 41.6509282 39.3848563

15 C 34.2689715 40.1053679 40.2170059

16 O 34.5708765 39.3564528 41.2213213

17 O 33.6742884 39.7128771 39.2027155

18 C 30.5392826 36.1797916 45.0438950

19 H 29.5433309 36.6508059 45.1109432

20 H 30.3659641 35.1515764 44.6913309

21 C 31.4062171 36.9022217 44.0579038

22 N 31.5145046 38.2818194 43.9719093

23 H 31.0082499 38.9827866 44.5236664

24 C 32.3878775 38.5813031 42.9894024

25 H 32.6868981 39.5915430 42.7245878

26 N 32.8481696 37.4757118 42.4245307

27 C 32.2365511 36.4233703 43.0692347

28 H 32.4444762 35.3982679 42.7759114

29 Fe 34.6516192 37.3331021 41.2872066

30 O 36.0386854 37.3346234 40.4530830

31 O 35.7970609 34.6182996 39.7532586

32 C 34.7771214 34.5343592 40.4117293

33 O 34.3430679 35.4442655 41.2754865

34 C 33.8660072 33.3160289 40.3509157

35 C 34.3331258 32.1738436 41.2822130

36 C 35.3729897 31.2326573 40.6294331

37 O 36.5949196 31.5200637 40.7284464

38 O 34.8985117 30.2495630 40.0185101

39 H 32.8469930 33.6261182 40.6191556

40 H 33.8495377 32.9534749 39.3127742

41 H 34.7327634 32.5967518 42.2221500

42 H 33.4516457 31.5645986 41.5322883

43 N 39.7477775 41.3242645 43.6652674

44 C 39.3552130 39.9898631 43.0908736

45 H 38.2563868 39.9985710 43.0558201

46 C 39.9271516 39.8244286 41.6676278

47 H 40.1453784 40.8259705 41.2626661

48 H 40.8946923 39.2999967 41.7159208

49 C 39.0002924 39.1149853 40.6708057

50 H 38.0039120 39.5887191 40.6994701

51 H 38.8355375 38.0693748 40.9808192

52 C 39.5549932 39.1966826 39.2387622

53 H 39.8453982 40.2417254 39.0440174

54 H 40.4863599 38.6087223 39.1541762

55 C 38.5559501 38.7691045 38.1612076

56 H 38.4649038 37.6772427 38.0678381

57 H 37.5442380 39.1487151 38.3612433

58 N 38.9426052 39.3180425 36.8263003

59 H 39.0032443 40.3618066 36.8650579

60 H 38.2544236 39.0933764 36.0800416

61 H 39.8646344 39.0144955 36.4533239

62 C 39.8319169 38.9101424 44.0868256

63 O 39.4409518 37.7363696 43.9234238

64 O 40.6127847 39.3198213 44.9826074

65 H 39.3240774 41.4427291 44.6020053

66 H 39.4328965 42.1076860 43.0561870

67 H 40.7736707 41.4126814 43.8140066

68 H 36.1181675 38.8312120 47.1799789

69 H 35.0368403 42.1157009 41.0598872

70 H 30.9024571 36.1446391 46.0709956

Hydrox-TS1

QM(B1)/MM Energy = -3127.254419573205 a.u.

QM(B2)/MM Energy = -3129.495206000000 a.u.

QM(B3)/MM Energy = -3128.934329684608 a.u.

1 C 36.9005948 38.5887120 46.6090340

2 H 37.4661021 39.4687292 46.2716583

3 H 37.5980620 37.9740401 47.1962920

4 C 36.3976637 37.8450444 45.4129328

5 N 36.0588008 36.5087309 45.4117252

6 H 36.1064187 35.8835178 46.2230512

7 C 35.6190518 36.1752338 44.1797163

8 H 35.2868650 35.1830893 43.8854818

9 N 35.6701789 37.2301377 43.3831516

10 C 36.1396298 38.2748986 44.1319221

11 H 36.2434612 39.2610330 43.6952685

12 C 34.6880640 41.6920945 40.2127257

13 H 33.8233369 42.2328109 39.8055513

14 H 35.4618297 41.7570943 39.4281571

15 C 34.3315626 40.2057640 40.3046522

16 O 34.9158725 39.4618036 41.1726072

17 O 33.5320587 39.7970689 39.4433752

18 C 30.6845918 36.2001346 44.9034437

19 H 29.6843982 36.6668508 44.9171733

20 H 30.5333838 35.1708152 44.5428208

21 C 31.5903506 36.9274740 43.9538982

22 N 31.6519769 38.3077097 43.8393754

23 H 31.0982728 38.9954436 44.3616891

24 C 32.5497877 38.6110345 42.8734606

25 H 32.8109768 39.6280775 42.5940984

26 N 33.0714826 37.5147490 42.3487851

27 C 32.4756230 36.4602636 43.0048115

28 H 32.7273137 35.4333070 42.7519200

29 Fe 35.1425450 37.3737817 41.3105254

30 O 36.8417782 37.5345663 40.8107599

31 O 36.0984535 34.6238897 39.5665401

32 C 35.1449533 34.5655772 40.3239708

33 O 34.8927818 35.4460995 41.2803041

34 C 34.1256825 33.4333025 40.2805159

35 C 34.4554915 32.2745406 41.2495463

36 C 35.4420400 31.2516605 40.6433952

37 O 36.6770190 31.4877998 40.7295553

38 O 34.9226329 30.2675706 40.0726510

39 H 33.1401757 33.8515863 40.5319324

40 H 34.0787422 33.0443735 39.2535271

41 H 34.8553280 32.6831474 42.1952943

42 H 33.5205346 31.7406184 41.4769292

43 N 39.5704137 41.1950165 43.4663526

44 C 39.1912396 39.7964194 43.0679753

45 H 38.0985939 39.7465540 43.1505534

46 C 39.6292602 39.4305499 41.6353172

47 H 40.4138935 40.1369725 41.3096631

48 H 40.1221046 38.4464226 41.6725176

49 C 38.5634914 39.3943571 40.5439419

50 H 37.8760673 40.2559482 40.5752559

51 H 37.7494554 38.4559712 40.7456983

52 C 39.1870685 39.2034073 39.1675112

53 H 39.7289603 40.1357914 38.9217508

54 H 39.9614280 38.4167135 39.2180725

55 C 38.2012604 38.8864270 38.0424103

56 H 37.9511048 37.8172763 38.0013244

57 H 37.2529509 39.4307245 38.1509992

58 N 38.7663617 39.3042846 36.7244743

59 H 38.9178411 40.3392009 36.7212868

60 H 38.1386598 39.0951333 35.9232337

61 H 39.6910726 38.9099368 36.4641148

62 C 39.7941460 38.8487766 44.1310471

63 O 39.4801946 37.6424041 44.0579540

64 O 40.5600348 39.3754719 44.9722613

65 H 39.2034202 41.3847535 44.4164467

66 H 39.1996644 41.9261308 42.8252569

67 H 40.5981258 41.3269586 43.5628474

68 H 36.1122035 38.9020033 47.2933986

69 H 35.0443498 42.2200244 41.0972694

70 H 30.9852841 36.1622901 45.9504485

Hydrox-IM1

QM(B1)/MM Energy = -3127.280348611462 a.u.

QM(B2)/MM Energy = -3129.521894000000 a.u.

QM(B3)/MM Energy = -3128.954462077702 a.u.

1 C 36.8880598 38.5578889 46.5328140

2 H 37.4473729 39.4362773 46.1803307

3 H 37.5968171 37.9461034 47.1096448

4 C 36.3593355 37.8055848 45.3521409

5 N 36.0013643 36.4741324 45.3839841

6 H 36.0702096 35.8607291 46.2029093

7 C 35.5223398 36.1245056 44.1725466

8 H 35.1681146 35.1318386 43.9069940

9 N 35.5669593 37.1637055 43.3536327

10 C 36.0780776 38.2159845 44.0685239

11 H 36.1948418 39.1904353 43.6070109

12 C 34.6646687 41.6376107 40.1866684

13 H 33.8141084 42.1922061 39.7676536

14 H 35.4409855 41.6879899 39.4032324

15 C 34.2718046 40.1554619 40.2741186

16 O 34.7492663 39.4078309 41.2016746

17 O 33.5402326 39.7519174 39.3512572

18 C 30.5837121 36.1606418 44.9888582

19 H 29.5847817 36.6278578 45.0350367

20 H 30.4200551 35.1281899 44.6436901

21 C 31.4609283 36.8783018 44.0080039

22 N 31.5445155 38.2578623 43.8966963

23 H 31.0129771 38.9563836 44.4278106

24 C 32.4246350 38.5524200 42.9154702

25 H 32.7049704 39.5642423 42.6351097

26 N 32.9132043 37.4471870 42.3765862

27 C 32.3145172 36.3977101 43.0388854

28 H 32.5447943 35.3682935 42.7756271

29 Fe 34.8792294 37.2597301 41.3276929

30 O 36.5808060 37.5233089 40.6102174

31 O 35.9465286 34.5257767 39.6060393

32 C 34.9797964 34.4561828 40.3442121

33 O 34.7218310 35.3282442 41.3103786

34 C 33.9752739 33.3185037 40.2774060

35 C 34.3375038 32.1609783 41.2340371
36 C 35.3864824 31.2005552 40.6308838
37 O 36.6071189 31.4839268 40.7582495
38 O 34.9240508 30.2133318 40.0173927
39 H 32.9802993 33.7119620 40.5309741
40 H 33.9446608 32.9409882 39.2459071
41 H 34.6959332 32.5737185 42.1944822
42 H 33.4248508 31.5783260 41.4305952
43 N 39.7849437 41.1224258 43.3633052
44 C 39.2974428 39.7347405 43.0616187
45 H 38.2007966 39.7903566 43.1201352
46 C 39.6959161 39.1762262 41.6930326
47 H 40.7988774 39.1280028 41.6124444
48 H 39.3669494 38.1186291 41.7367813
49 C 39.1044501 39.8184627 40.4838917
50 H 38.3135929 40.5698307 40.5866353
51 H 36.7670243 38.4680361 40.5013400
52 C 39.6135469 39.5033672 39.1127491
53 H 40.1388761 40.3939850 38.7197894
54 H 40.3749519 38.7052880 39.1612970
55 C 38.5115153 39.1180025 38.1115175
56 H 38.2216416 38.0609036 38.1915267
57 H 37.5973413 39.7099517 38.2582864
58 N 38.9447682 39.4071553 36.7131997
59 H 39.0924884 40.4375375 36.6267203
60 H 38.2408999 39.1464438 35.9940949
61 H 39.8425248 38.9872021 36.4010570
62 C 39.8057479 38.8132216 44.1915527
63 O 39.4250042 37.6225870 44.1670645
64 O 40.5813327 39.3368893 45.0242735
65 H 39.4269485 41.3828826 44.3017023
66 H 39.4529721 41.8431613 42.6895031
67 H 40.8149950 41.2042955 43.4630361
68 H 36.1136162 38.8735981 47.2318360
69 H 35.0211864 42.1716880 41.0674202
70 H 30.9280475 36.1360829 46.0227330

Hydrox-TS2

QM(B1)/MM Energy = -3127.270129790571 a.u.

QM(B2)/MM Energy = -3129.506399000000 a.u.

QM(B3)/MM Energy = -3128.945206154995 a.u.

1 C 36.8616414 38.7115952 46.6183519
2 H 37.3978256 39.6134654 46.2899740
3 H 37.5779126 38.1189639 47.2059132
4 C 36.3989912 37.9604301 45.4106131
5 N 36.1307839 36.6085935 45.3850068
6 H 36.1879986 35.9743616 46.1890174
7 C 35.7388767 36.2724727 44.1361987
8 H 35.4547469 35.2713799 43.8211724
9 N 35.7511035 37.3383959 43.3508890
10 C 36.1399039 38.3952521 44.1311301
11 H 36.1884005 39.3949078 43.7160324
12 C 34.7002663 41.7501348 40.2341360
13 H 33.8438747 42.2783281 39.7946598
14 H 35.4975790 41.8076869 39.4724856
15 C 34.3487603 40.2674025 40.3556855
16 O 34.9383045 39.5455414 41.2368881
17 O 33.5388104 39.8358405 39.5148331
18 C 30.6663181 36.1788302 44.8985802
19 H 29.6607390 36.6341734 44.9124345
20 H 30.5261860 35.1458106 44.5439557
21 C 31.5630662 36.9132463 43.9467325
22 N 31.6012851 38.2934600 43.8275747
23 H 31.0394555 38.9752779 44.3486705
24 C 32.4944647 38.6081000 42.8608433

25 H 32.7346343 39.6294063 42.5769193
26 N 33.0348803 37.5191069 42.3407927
27 C 32.4580204 36.4568856 43.0004572
28 H 32.7295134 35.4332656 42.7518512
29 Fe 35.0929968 37.4715015 41.3049917
30 O 36.7376065 37.6362086 40.3025334
31 O 36.2820416 34.9051347 39.5426022
32 C 35.3284558 34.6929730 40.3022401
33 O 35.0243376 35.4494390 41.3165943
34 C 34.3907776 33.5066610 40.1131571
35 C 34.5671043 32.3700494 41.1512094
36 C 35.5217055 31.2773068 40.6297517
37 O 36.7628307 31.4970788 40.6945389
38 O 34.9839629 30.2689084 40.1247027
39 H 33.3693486 33.9094874 40.1892430
40 H 34.5151551 33.0954854 39.1034874
41 H 34.9336476 32.7883203 42.1052044
42 H 33.5897220 31.9024352 41.3400230
43 N 39.6886582 40.9973207 43.1143965
44 C 39.1697595 39.5989817 42.9532878
45 H 38.0901137 39.6647627 43.1392686
46 C 39.4007577 38.9184937 41.5946273
47 H 40.4524373 39.0542198 41.2791319
48 H 39.3105443 37.8431618 41.8311967
49 C 38.4759024 39.2104848 40.4481421
50 H 37.5802649 39.8111554 40.6160277
51 H 36.9163989 36.7682169 39.8807291
52 C 39.0665052 39.1665070 39.0758496
53 H 39.5440259 40.1551210 38.9033053
54 H 39.9042427 38.4458600 39.0490864
55 C 38.0985878 38.8892619 37.9257430
56 H 37.8516871 37.8236094 37.8407057
57 H 37.1465696 39.4230801 38.0423818
58 N 38.6916209 39.3460351 36.6329094
59 H 38.8375762 40.3813200 36.6547903
60 H 38.0799682 39.1458073 35.8174757
61 H 39.6235296 38.9611491 36.3803279
62 C 39.7801492 38.7677437 44.1097635
63 O 39.4479142 37.5635037 44.1631282
64 O 40.5508696 39.3623477 44.8942134
65 H 39.4420431 41.3004823 44.0785148
66 H 39.2834937 41.7026482 42.4664966
67 H 40.7201750 41.0804129 43.1045498
68 H 36.0565802 38.9894156 47.2986161
69 H 35.0264213 42.2911776 41.1223717
70 H 30.9716482 36.1520154 45.9445829

Hydrox-PC

QM(B1)/MM Energy = -3127.371087022366 a.u.

QM(B2)/MM Energy = -3129.606237000000 a.u.

QM(B3)/MM Energy = -3129.027035276447 a.u.

1 C 36.8574703 38.5109070 46.4713106
2 H 37.4411288 39.3750183 46.1223422
3 H 37.5520717 37.8760129 47.0412422
4 C 36.3018800 37.7837924 45.2856715
5 N 35.9000631 36.4653792 45.3076785
6 H 35.9330289 35.8527340 46.1290094
7 C 35.3956532 36.1517869 44.0909254
8 H 34.9809482 35.1805548 43.8313324
9 N 35.4650711 37.1939620 43.2765660
10 C 36.0245561 38.2128219 44.0057324
11 H 36.1873436 39.1864454 43.5559263
12 C 34.7894296 41.6400484 40.1139473
13 H 33.9690359 42.2051345 39.6525613
14 H 35.6152432 41.6692430 39.3807949

15 C 34.3534106 40.1778920 40.2074431
16 O 34.9562563 39.3730335 41.0089157
17 O 33.4611160 39.8342961 39.4112330
18 C 30.4619898 36.2413527 45.1990557
19 H 29.4556613 36.6819696 45.3082524
20 H 30.3042297 35.2148232 44.8351637
21 C 31.2708240 37.0021439 44.1941712
22 N 31.3586920 38.3819712 44.1329570
23 H 30.9009391 39.0746942 44.7324715
24 C 32.1649569 38.7125222 43.1008412
25 H 32.4062173 39.7414125 42.8403744
26 N 32.6008441 37.6293107 42.4835242
27 C 32.0451752 36.5565130 43.1450797
28 H 32.2456627 35.5375614 42.8210497
29 Fe 34.4257401 37.4267836 41.3494948
30 O 38.3584698 37.7592217 40.9780834
31 O 35.9577812 36.0954974 40.2148045
32 C 35.2116020 35.1355202 40.5701846
33 O 34.2222123 35.3270698 41.3404511
34 C 35.4442170 33.7380755 40.0454867
35 C 35.0346274 32.5982672 40.9901439
36 C 35.7700238 31.3038149 40.5962599
37 O 37.0349858 31.3603484 40.6758922
38 O 35.0943769 30.3369643 40.1958487
39 H 34.8496518 33.6599149 39.1209076
40 H 36.4986527 33.6348667 39.7471449
41 H 35.3223000 32.8567052 42.0245627
42 H 33.9462677 32.4509244 40.9784564
43 N 39.6305796 41.2141180 43.5549238
44 C 39.2562362 39.8373039 43.0702074
45 H 38.1586414 39.8031832 43.0864510
46 C 39.7732132 39.5948590 41.6341962
47 H 40.1681454 40.5414533 41.2359191
48 H 40.6200359 38.8895598 41.6468762
49 C 38.7352480 39.0822931 40.6230090
50 H 37.8468650 39.7425718 40.6660372
51 H 37.4672173 37.5554178 40.6459811
52 C 39.3309558 39.1582196 39.2038076
53 H 39.6926096 40.1848434 39.0384845
54 H 40.2169320 38.5008680 39.1551130
55 C 38.3491429 38.8031675 38.0881270
56 H 38.1621993 37.7221812 38.0199428
57 H 37.3711446 39.2890996 38.2256876
58 N 38.8583444 39.2807829 36.7682494
59 H 38.9806353 40.3200290 36.7885182
60 H 38.2116457 39.0773942 35.9800406
61 H 39.7844010 38.9180605 36.4663081
62 C 39.7984600 38.8430930 44.1248833
63 O 39.4271551 37.6562658 44.0604785
64 O 40.5889798 39.3370304 44.9689172
65 H 39.2296111 41.3681152 44.4967346
66 H 39.2904975 41.9703481 42.9262208
67 H 40.6563088 41.3259566 43.6880834
68 H 36.1022572 38.8515058 47.1796275
69 H 35.1117823 42.1650196 41.0131476
70 H 30.8614401 36.2020434 46.2124468

Hydrox-3R[1]-RC
QM(B1)/MM Energy = -3358.773537913588 a.u.
QM(B2)/MM Energy = -3360.902457000000 a.u.
QM(B3)/MM Energy = -3360.378062273080 a.u.

1 C 39.4145213 44.0578717 38.1284141
2 H 40.3433803 43.4850290 37.9908720
3 H 39.5301742 44.6122814 39.0703028
4 C 38.2547413 43.1158246 38.1989701

5 N 37.1277874 43.3244370 38.9603113
6 H 36.9682401 44.1512214 39.5482817
7 C 36.2663135 42.3071234 38.7505114
8 H 35.2944968 42.2200718 39.2343220
9 N 36.7943310 41.4414852 37.8948621
10 C 38.0285377 41.9290340 37.5414713
11 H 38.6619393 41.4031953 36.8315781
12 C 33.4256762 44.2114716 34.8070353
13 H 33.1668094 44.4473723 33.7615776
14 H 32.4782840 43.9965889 35.3223743
15 C 34.3168701 43.0160488 34.8930625
16 N 35.3768931 42.7605777 34.0418644
17 H 35.6586883 43.3253173 33.2328790
18 C 35.9870430 41.6300188 34.4465835
19 H 36.8548124 41.1860353 33.9659618
20 N 35.3748180 41.1320424 35.5100988
21 C 34.3260501 41.9771187 35.7929252
22 H 33.6508007 41.7832221 36.6211720
23 Cl 37.5371914 38.9916977 35.2674007
24 Fe 36.0743007 39.7203488 36.9198419
25 O 36.6848465 38.7054215 38.0210752
26 O 34.0854855 38.1956106 39.1364203
27 C 33.5825402 38.7715987 38.1882881
28 O 34.2432472 39.4006359 37.2312699
29 C 32.0779171 38.9252547 38.0296298
30 C 31.5859225 40.1949907 38.7491242
31 C 31.6096309 40.0032705 40.2672719
32 O 32.3479473 40.7600845 40.9602786
33 O 30.8753676 39.0883693 40.7169840
34 H 31.8335790 38.9739043 36.9593626
35 H 31.5737812 38.0650965 38.4890392
36 H 32.1781732 41.0797829 38.4673450
37 H 30.5395691 40.3660358 38.4595455
38 N 42.1011486 40.0563924 38.0768131
39 C 40.7855343 39.9242536 38.7863093
40 H 40.0106467 40.1268834 38.0345164
41 C 40.5397772 38.5484105 39.4057575
42 H 41.3071762 38.3585392 40.1766356
43 H 39.5849962 38.6415849 39.9456299
44 C 40.4702761 37.3610920 38.4421621
45 H 41.4366539 37.2411539 37.9252760
46 H 39.7133709 37.5663791 37.6645067
47 C 40.1604370 36.0518805 39.1885957
48 H 40.6999175 36.0531900 40.1470089
49 H 39.0899255 35.9908889 39.4491809
50 C 40.6069318 34.7956377 38.4540150
51 H 41.5999671 34.9423864 38.0049498
52 H 40.6730148 33.9338391 39.1323026
53 N 39.6711133 34.3788046 37.3482380
54 H 38.9714200 33.6998496 37.7076578
55 H 40.1996480 33.8963418 36.5889623
56 H 39.1788896 35.1658680 36.8839588
57 C 40.7327521 41.0174945 39.8837619
58 O 41.8016824 41.6402712 40.0871182
59 O 39.6500914 41.1628994 40.4887547
60 H 42.9161417 40.0409337 38.7269967
61 H 42.1672003 41.0006018 37.6587779
62 H 42.2730289 39.3580816 37.3227209
63 H 39.3194106 44.8038524 37.3394059
64 H 33.8477512 45.1293399 35.2162266

Hydrox-3R^1-TS1

QM(B1)/MM Energy = -3358.735665490353 a.u.

QM(B2)/MM Energy = -3360.865391000000 a.u.

QM(B3)/MM Energy = -3360.347763423320 a.u.

1 C 39.4544185 44.1012055 38.1254292

2 H 40.3888412 43.5386519 37.9841758

3 H 39.5713454 44.6633890 39.0625444

4 C 38.3118742 43.1410716 38.2155181

5 N 37.1925440 43.3223620 38.9951146

6 H 37.0154450 44.1522191 39.5737605

7 C 36.3650199 42.2704556 38.8185796

8 H 35.4053431 42.1589071 39.3219363

9 N 36.9066344 41.4082827 37.9683650

10 C 38.1108665 41.9404526 37.5775969

11 H 38.7403837 41.4375532 36.8477022

12 C 33.4609979 44.1074531 34.8727272

13 H 33.1767400 44.3206254 33.8286441

14 H 32.5270813 43.8795403 35.4079611

15 C 34.3849563 42.9346032 34.9541136

16 N 35.4121961 42.6887935 34.0616171

17 H 35.6508111 43.2524826 33.2386076

18 C 36.0588250 41.5710345 34.4568193

19 H 36.9105274 41.1361733 33.9393384

20 N 35.5078722 41.0736020 35.5510743

21 C 34.4579760 41.9052403 35.8655680

22 H 33.8193790 41.7152723 36.7243850

23 Cl 37.6625863 38.9079541 35.1783389

24 Fe 36.3613135 39.5819029 37.0306416

25 O 37.2626028 38.5637458 38.1593738

26 O 34.2243730 38.1863265 39.3764667

27 C 33.8010094 38.6626818 38.3353291

28 O 34.5527594 39.1520974 37.3717599

29 C 32.3123988 38.8306654 38.0761545

30 C 31.7877979 40.1316225 38.7166850

31 C 31.7164035 40.0101818 40.2419559

32 O 32.4432277 40.7699914 40.9443917

33 O 30.9230900 39.1439588 40.6891180

34 H 32.1343243 38.8404256 36.9919006

35 H 31.7699598 37.9929843 38.5349517

36 H 32.4047890 40.9995391 38.4352124

37 H 30.7628028 40.2972375 38.3536573

38 N 42.0239573 40.0448969 38.0246002

39 C 40.7260677 39.9674055 38.7704390

40 H 39.9369198 40.2492923 38.0640157

41 C 40.4195480 38.5817108 39.3456251

42 H 41.3657329 38.0972544 39.6443057

43 H 39.8636045 38.7364946 40.2830988

44 C 39.6107099 37.6105737 38.4973325

45 H 39.8893911 37.6063340 37.4292282

46 H 38.4346030 38.0900786 38.3942704

47 C 39.5579729 36.2112150 39.1244026

48 H 39.8779495 36.2826914 40.1749542

49 H 38.5280200 35.8218482 39.1713200

50 C 40.4480578 35.1759026 38.4265482

51 H 41.3498277 35.6299779 37.9909799

52 H 40.7806665 34.3957820 39.1253949

53 N 39.7069377 34.4697980 37.3278078

54 H 39.1114477 33.7108253 37.7200778

55 H 40.3499141 34.0132629 36.6430202

56 H 39.1076657 35.1057380 36.7635417

57 C 40.7676856 41.0387867 39.8915257

58 O 41.8500599 41.6467148 40.0421086

59 O 39.7188699 41.1871913 40.5538227

60 H 42.8476733 40.0473955 38.6669776

61 H 42.0958512 40.9690215 37.5651150

62 H 42.1735581 39.3092387 37.3017258

63 H 39.3433617 44.8394416 37.3312372

64 H 33.8634426 45.0446567 35.2571163

Hydrox-3R^{1}-IM1

QM(B1)/MM Energy = -3358.763004307216 a.u.

QM(B2)/MM Energy = -3360.894607000000 a.u.

QM(B3)/MM Energy = -3360.370331800854 a.u.

1 C	39.4041563	44.0644249	38.1400746
2 H	40.3324195	43.4906716	38.0009686
3 H	39.5228706	44.6195053	39.0812246
4 C	38.2439535	43.1231251	38.2155937
5 N	37.1211148	43.3288223	38.9841607
6 H	36.9621202	44.1571436	39.5702317
7 C	36.2637199	42.3059095	38.7855903
8 H	35.2974541	42.2162316	39.2804814
9 N	36.7881668	41.4366324	37.9308149
10 C	38.0167932	41.9338119	37.5643031
11 H	38.6445757	41.4163527	36.8423894
12 C	33.4133003	44.1760469	34.8345090
13 H	33.1345891	44.4108512	33.7936890
14 H	32.4754378	43.9559319	35.3651576
15 C	34.3123228	42.9849830	34.9027936
16 N	35.3556552	42.7370313	34.0295376
17 H	35.6225369	43.3069117	33.2196826
18 C	35.9738309	41.6022167	34.4174812
19 H	36.8340416	41.1647176	33.9164665
20 N	35.3864842	41.0952600	35.4883762
21 C	34.3436983	41.9383719	35.7949375
22 H	33.6832242	41.7438725	36.6360920
23 Cl	37.6139803	38.9597477	35.2168452
24 Fe	36.1723138	39.6307105	36.9770092
25 O	37.0798402	38.5415222	38.1873970
26 O	34.0780348	38.1993212	39.2928024
27 C	33.6277843	38.6922589	38.2712790
28 O	34.3638725	39.1838771	37.2935698
29 C	32.1384069	38.8877097	38.0537915
30 C	31.6646473	40.1838195	38.7389805
31 C	31.6516452	40.0227556	40.2612202
32 O	32.3945455	40.7737033	40.9560316
33 O	30.8827817	39.1376396	40.7124530
34 H	31.9315950	38.9255014	36.9752998
35 H	31.5889650	38.0538219	38.5105314
36 H	32.2861485	41.0474948	38.4536760
37 H	30.6296734	40.3765228	38.4228073
38 N	42.1285774	40.0521872	38.0785056
39 C	40.8119443	39.9649908	38.7838519
40 H	40.0462668	40.1992540	38.0319071
41 C	40.5148982	38.5993207	39.4005507
42 H	41.2877176	38.3568943	40.1548610
43 H	39.5964996	38.7567093	40.0011512
44 C	40.3205218	37.4465022	38.4732658
45 H	40.1250426	37.6204057	37.4070658
46 H	38.0425576	38.5120037	38.0809819
47 C	40.1682475	36.0735307	39.0520300
48 H	40.6725066	36.0416516	40.0293037
49 H	39.1031597	35.8566444	39.2816384
50 C	40.7394447	34.9453560	38.1921285
51 H	41.6039056	35.2862405	37.6056634
52 H	41.0699146	34.1027710	38.8143184
53 N	39.7330240	34.3813731	37.2295531
54 H	39.1134717	33.6922374	37.7039350
55 H	40.2273026	33.8723573	36.4644571
56 H	39.1470195	35.1017123	36.7630507
57 C	40.7800510	41.0531615	39.8863730
58 O	41.8520682	41.6681759	40.0825873
59 O	39.7008340	41.1965215	40.4996472
60 H	42.9399118	40.0501229	38.7344342
61 H	42.2075275	40.9795287	37.6259431
62 H	42.2890122	39.3178098	37.3555001

63 H 39.3093211 44.8097428 37.3504072

64 H 33.8351294 45.0979218 35.2348515

Hydrox-3R[1]-TS-Cl

QM(B1)/MM Energy = -3358.724876315689 a.u.

QM(B2)/MM Energy = -3360.852558000000 a.u.

QM(B3)/MM Energy = -3360.330399648283 a.u.

1 C 39.4070114 44.1024148 38.1615936

2 H 40.3333014 43.5237459 38.0261407

3 H 39.5350525 44.6708694 39.0935759

4 C 38.2485225 43.1603717 38.2663487

5 N 37.1636928 43.3292612 39.0975480

6 H 37.0017116 44.1618619 39.6762910

7 C 36.3420557 42.2677077 38.9627970

8 H 35.4063514 42.1472381 39.5083359

9 N 36.8530892 41.4066369 38.0893950

10 C 38.0273327 41.9609735 37.6366739

11 H 38.6311059 41.4606951 36.8850692

12 C 33.5976599 43.9204879 34.8661385

13 H 33.3145467 44.1158891 33.8176821

14 H 32.6674297 43.6511302 35.3898545

15 C 34.5596202 42.7743835 34.9553351

16 N 35.6284402 42.5586765 34.1010366

17 H 35.8817280 43.1429298 33.2961230

18 C 36.2597084 41.4263201 34.4947498

19 H 37.1447546 41.0217611 34.0062830

20 N 35.6591316 40.8902577 35.5438161

21 C 34.5917286 41.7130931 35.8304050

22 H 33.9133675 41.4904590 36.6491355

23 Cl 38.3286235 38.7969186 36.1553446

24 Fe 36.3139449 39.5201473 37.2603671

25 O 36.8022652 38.3053331 38.5935206

26 O 33.9987890 38.2681071 39.3896092

27 C 33.6363463 38.7219671 38.3146580

28 O 34.4368011 39.1254777 37.3489889

29 C 32.1641548 38.9738144 38.0320471

30 C 31.7107027 40.2602801 38.7469673

31 C 31.6847646 40.0695683 40.2663075

32 O 32.4267345 40.8013085 40.9829777

33 O 30.8965634 39.1896619 40.6953112

34 H 31.9988022 39.0516875 36.9481978

35 H 31.5691402 38.1445885 38.4387741

36 H 32.3499909 41.1193282 38.4858414

37 H 30.6812368 40.4803131 38.4304351

38 N 42.1931177 40.2147759 38.0050601

39 C 40.8184594 40.1885923 38.5998358

40 H 40.1622666 40.6182016 37.8346965

41 C 40.3114960 38.7985990 38.9465659

42 H 40.9879991 38.3299493 39.6933811

43 H 39.3839386 38.9736213 39.5202792

44 C 40.0779069 37.8398920 37.8249410

45 H 40.6566053 37.9434508 36.9082666

46 H 37.7525420 38.2070356 38.7257690

47 C 39.6120603 36.4612702 38.2062446

48 H 39.6210361 36.3523485 39.3036866

49 H 38.5511947 36.3463327 37.9267150

50 C 40.4296974 35.3414519 37.5528339

51 H 40.9994633 35.7213968 36.6966157

52 H 41.1510566 34.8808898 38.2426896

53 N 39.5537702 34.2517361 37.0063444

54 H 39.1939355 33.5912546 37.7279899

55 H 40.1089980 33.6967263 36.3196715

56 H 38.7483370 34.6675850 36.4981722

57 C 40.7929747 41.1013977 39.8514983

58 O 41.8657717 41.6887498 40.1224320

59 O 39.7143105 41.1641799 40.4811424

60 H 42.9553945 40.1001066 38.7069442

61 H 42.3689494 41.1600039 37.6238024

62 H 42.3498227 39.5236882 37.2409148

63 H 39.3188332 44.8381824 37.3622578

64 H 33.9346080 44.8838549 35.2488357

Hydrox-3R[1]-PC-Cl

QM(B1)/MM Energy = -3358.787002429435 a.u.

QM(B2)/MM Energy = -3360.923636000000 a.u.

QM(B3)/MM Energy = -3360.389798847904 a.u.

1 C 39.2841508 43.9078939 38.1334652

2 H 40.2090484 43.3251165 38.0049425

3 H 39.3897615 44.4408723 39.0896554

4 C 38.1083022 42.9791297 38.1705438

5 N 36.9610456 43.2232185 38.8898537

6 H 36.7930173 44.0706454 39.4441286

7 C 36.1005902 42.1982303 38.6872589

8 H 35.1125260 42.1410429 39.1436296

9 N 36.6391997 41.2851273 37.8886635

10 C 37.8862629 41.7654311 37.5557135

11 H 38.5477713 41.2192535 36.8855436

12 C 33.4636952 44.2113958 34.7176636

13 H 33.1828798 44.4865678 33.6869100

14 H 32.5256774 43.9605827 35.2348718

15 C 34.3632882 43.0176417 34.7381148

16 N 35.4548765 42.8142942 33.9149124

17 H 35.7857827 43.4292271 33.1650128

18 C 36.0194634 41.6307938 34.2488668

19 H 36.9002274 41.2286976 33.7506731

20 N 35.3547579 41.0533964 35.2326600

21 C 34.3126142 41.9007655 35.5381901

22 H 33.5944115 41.6410584 36.3115135

23 Cl 40.0753052 37.6207972 36.8079189

24 Fe 35.7768119 39.5966893 36.7845992

25 O 36.9941772 38.1982876 36.4382755

26 O 33.9101926 38.3907430 39.1482859

27 C 33.3061556 38.8835438 38.1937792

28 O 33.8770643 39.4692610 37.1886723

29 C 31.7900593 38.9597035 38.1505840

30 C 31.3263914 40.2231009 38.8967586

31 C 31.4955710 40.0199963 40.3993083

32 O 32.2590345 40.7947757 41.0438360

33 O 30.8362746 39.0700774 40.8925534

34 H 31.4665765 38.9921087 37.1009186

35 H 31.3445912 38.0907336 38.6514607

36 H 31.8725159 41.1183971 38.5605159

37 H 30.2541988 40.3738469 38.7077061

38 N 42.2013848 40.0996717 38.0912581

39 C 40.8822393 39.9363884 38.7788983

40 H 40.1218349 40.1409143 38.0151965

41 C 40.6361750 38.5500925 39.3721004

42 H 41.2959832 38.3925887 40.2436719

43 H 39.6120931 38.5775921 39.7717327

44 C 40.8152297 37.3362164 38.4688856

45 H 41.8714401 37.1824825 38.2220226

46 H 37.9138365 38.4445787 36.6023340

47 C 40.2400687 36.0625610 39.0790425

48 H 40.6619905 35.9771486 40.0927009

49 H 39.1494109 36.1612468 39.2117707

50 C 40.6002634 34.7873164 38.3211356

51 H 41.5758793 34.8901646 37.8248153

52 H 40.6717488 33.9332457 39.0074657

53 N 39.6009722 34.3827093 37.2661217

54 H 38.9639312 33.6527720 37.6427991

55 H 40.1101649 33.9503342 36.4640773

56 H 39.0344118 35.1558900 36.8609021

57 C 40.7718016 41.0003228 39.9022115

58 O 41.8206260 41.6441991 40.1384844

59 O 39.6724427 41.0948972 40.4834989

60 H 43.0185135 40.0747987 38.7377117

61 H 42.2550716 41.0543983 37.6924548

62 H 42.3778531 39.4224673 37.3173780

63 H 39.2358852 44.6773488 37.3629610

64 H 33.8675808 45.1239144 35.1561307

Hydrox-3R[1]-TS-OH

QM(B1)/MM Energy = -3358.742919811499 a.u.

QM(B2)/MM Energy = -3360.872421000000 a.u.

QM(B3)/MM Energy = -3360.352370695470 a.u.

1 C 39.4356861 44.0992061 38.1449502

2 H 40.3768961 43.5462204 38.0087431

3 H 39.5468248 44.6696453 39.0778400

4 C 38.3093887 43.1209805 38.2452981

5 N 37.1824680 43.2821577 39.0190389

6 H 36.9787553 44.1184611 39.5796299

7 C 36.3961827 42.1933375 38.8713689

8 H 35.4383442 42.0641442 39.3752169

9 N 36.9707053 41.3234875 38.0480274

10 C 38.1510426 41.8970278 37.6431507

11 H 38.8051625 41.4042205 36.9296858

12 C 33.4750800 44.0749158 34.9107757

13 H 33.1789176 44.2710140 33.8668462

14 H 32.5497713 43.8368798 35.4567236

15 C 34.4238927 42.9216384 34.9933121

16 N 35.4122997 42.6661382 34.0609977

17 H 35.6133454 43.2191842 33.2209833

18 C 36.0889884 41.5648123 34.4494819

19 H 36.9055180 41.1132635 33.8913664

20 N 35.5969083 41.0885268 35.5811666

21 C 34.5553448 41.9183749 35.9272963

22 H 33.9587177 41.7449785 36.8193240

23 Cl 37.3093319 38.6782799 34.8292762

24 Fe 36.4640116 39.5519134 36.9601968

25 O 37.9997991 38.4338588 37.6321462

26 O 34.2642371 38.1649892 39.5560166

27 C 33.9273253 38.5920962 38.4574918

28 O 34.7669830 38.9505953 37.5228491

29 C 32.4636130 38.8145949 38.1110894

30 C 31.9368344 40.1306971 38.7187124

31 C 31.7889954 40.0310939 40.2409175

32 O 32.5020713 40.7819828 40.9666128

33 O 30.9506014 39.1939914 40.6622851

34 H 32.3487434 38.8253418 37.0178128

35 H 31.8681560 37.9951161 38.5367738

36 H 32.5859617 40.9830968 38.4616638

37 H 30.9348189 40.3172422 38.3030470

38 N 42.0788430 40.0704873 38.0920059

39 C 40.7623733 40.0202852 38.8056437

40 H 39.9928881 40.2768599 38.0704082

41 C 40.4074383 38.6671466 39.4050880

42 H 41.2325253 38.3241875 40.0611100

43 H 39.5702579 38.8532945 40.0972790

44 C 40.0163330 37.5731390 38.4723496

45 H 40.3438689 37.6310921 37.4297806

46 H 38.4502798 38.3229848 36.7807297

47 C 39.8501562 36.2017371 39.0720339

48 H 40.2996573 36.2083193 40.0788381

49 H 38.7855653 35.9675165 39.2538696

50 C 40.5120931 35.0689399 38.2825924

51 H 41.4201666 35.4140663 37.7678503

52 H 40.8088177 34.2487351 38.9501847

53 N 39.6090292 34.4590617 37.2511964

54 H 38.9766256 33.7557259 37.6847103

55 H 40.1738826 33.9508517 36.5346148

56 H 39.0428830 35.1490904 36.7169444

57 C 40.7839336 41.0991529 39.9135473

58 O 41.8643895 41.7085500 40.0735346

59 O 39.7288324 41.2466841 40.5668159

60 H 42.8941787 40.0581092 38.7436606

61 H 42.1733055 40.9945677 37.6347534

62 H 42.2274202 39.3350813 37.3685645

63 H 39.3223302 44.8297638 37.3440114

64 H 33.8655992 45.0228648 35.2808494

Hydrox-3R[1]-PC-OH

QM(B1)/MM Energy = -3358.843730164869 a.u.

QM(B2)/MM Energy = -3360.967947000000 a.u.

QM(B3)/MM Energy = -3360.433072802625 a.u.

1 C 39.3261463 43.9499801 38.1467787

2 H 40.2521471 43.3717088 38.0082260

3 H 39.4485614 44.5025035 39.0893372

4 C 38.1599928 43.0136175 38.2235361

5 N 37.0425948 43.2292443 38.9975664

6 H 36.8888742 44.0655672 39.5736041

7 C 36.1848083 42.2008114 38.8155830

8 H 35.2270297 42.1176512 39.3299222

9 N 36.6988569 41.3144884 37.9684931

10 C 37.9279011 41.8127677 37.5923470

11 H 38.5550678 41.2844874 36.8773962

12 C 33.3322260 44.2812457 34.7861030

13 H 33.0786658 44.5477211 33.7469729

14 H 32.3807482 44.0898332 35.3009748

15 C 34.1676529 43.0475103 34.8465779

16 N 35.2105879 42.7494990 33.9921653

17 H 35.5318823 43.3179161 33.2005035

18 C 35.7536163 41.5765194 34.3738756

19 H 36.6091077 41.1045663 33.8947676

20 N 35.1116001 41.0904919 35.4269946

21 C 34.1178505 41.9938090 35.7279311

22 H 33.4342571 41.8478942 36.5613611

23 Cl 37.6270352 38.8998027 35.3281484

24 Fe 35.9632627 39.7032221 36.7621550

25 O 39.2354635 37.5559306 37.6917233

26 O 34.1258746 38.0100624 39.8561242

27 C 34.0403923 38.3786448 38.6910461

28 O 35.0852820 38.4332329 37.8979607

29 C 32.6962715 38.8154808 38.1191809

30 C 32.1900551 40.1427883 38.7166324

31 C 31.8425290 40.0379232 40.2104671

32 O 32.4906775 40.7682942 41.0134066

33 O 30.9219319 39.2439351 40.5241373

34 H 32.7635518 38.8845079 37.0224272

35 H 31.9551676 38.0456780 38.3795651

36 H 32.9246001 40.9532242 38.5754867

37 H 31.2646899 40.4197068 38.1855979

38 N 42.1480842 40.0925752 38.0797451

39 C 40.8492521 39.9375627 38.8105256

40 H 40.0496673 40.1389674 38.0844069

41 C 40.6396457 38.5580877 39.4363611

42 H 41.4882390 38.3314944 40.1008667

43 H 39.7524267 38.6507829 40.0807574

44 C 40.4184045 37.3837813 38.4753274

45 H 41.2929103 37.2829566 37.8066046

46 H 39.3486406 38.1502912 36.9309675

47 C 40.2418143 36.0613269 39.2306368

48 H 40.8999288 36.0459581 40.1067353

49 H 39.2103301 36.0132222 39.6194735

50 C 40.5769247 34.8294191 38.4015468

51 H 41.5569773 34.9493446 37.9169011

52 H 40.6232689 33.9288960 39.0290353

53 N 39.5756492 34.5415992 37.3174209

54 H 38.8258399 33.9186479 37.6699461

55 H 40.0483906 34.0368619 36.5343738

56 H 39.1739783 35.4006363 36.8948443

57 C 40.8039208 41.0287961 39.9112136

58 O 41.8717579 41.6519098 40.1066563

59 O 39.7241610 41.1706162 40.5205748

60 H 42.9684454 40.1165281 38.7247040

61 H 42.1807768 41.0268481 37.6353766

62 H 42.3409162 39.3836943 37.3405745

63 H 39.2472567 44.6997463 37.3595722

64 H 33.7936278 45.1718180 35.2127838

Hydrox-3R[1]-Iso1-TS

QM(B1)/MM Energy = -3358.750173359607 a.u.

QM(B2)/MM Energy = -3360.880160000000 a.u.

QM(B3)/MM Energy = -3360.355897921549 a.u.

1 C 39.4998857 44.1655129 38.1197947

2 H 40.4362882 43.6075008 37.9787188

3 H 39.6122560 44.7309817 39.0551032

4 C 38.3681237 43.1949069 38.2071432

5 N 37.2363365 43.3664296 38.9743556

6 H 37.0457469 44.1925477 39.5543661

7 C 36.4190677 42.3145796 38.7862668

8 H 35.4509006 42.1866284 39.2666353

9 N 36.9782639 41.4599862 37.9323580

10 C 38.1884712 41.9976870 37.5618417

11 H 38.8282823 41.4947230 36.8417224

12 C 33.4380051 44.0987913 34.8582178

13 H 33.1696347 44.3223665 33.8120388

14 H 32.4931997 43.8912778 35.3820710

15 C 34.3220720 42.8961347 34.9440190

16 N 35.3721288 42.6287236 34.0822020

17 H 35.6521747 43.1989741 33.2758532

18 C 35.9640859 41.4822801 34.4739779

19 H 36.8418130 41.0448955 34.0037558

20 N 35.3508838 40.9811034 35.5384592

21 C 34.3194137 41.8482599 35.8345516

22 H 33.6613667 41.6676988 36.6795585

23 Cl 38.0200379 39.2440534 35.4939672

24 Fe 36.2194997 39.7085079 37.1148397

25 O 36.5725612 37.9793503 37.6756393

26 O 34.1110340 38.1335410 39.4317692

27 C 33.7377693 38.9086231 38.5683171

28 O 34.5221554 39.8044890 37.9928997

29 C 32.2896037 38.9981052 38.1239563

30 C 31.6069728 40.2150632 38.7764687

31 C 31.5647386 40.0112521 40.2921817

32 O 32.3241260 40.7251542 41.0100065

33 O 30.7984346 39.1088636 40.7103206

34 H 32.2541746 39.0802462 37.0256065

35 H 31.7615892 38.0910974 38.4436143

36 H 32.1409739 41.1468057 38.5355743

37 H 30.5763387 40.2771254 38.4023511

38 N 42.1065637 40.0276367 38.0878006

39 C 40.7848148 39.9349641 38.7853276

40 H 40.0245904 40.1550368 38.0244597

41 C 40.4844058 38.5682021 39.3983566

42 H 41.2529696 38.3196932 40.1548141

43 H 39.5629509 38.7293578 39.9921500

44 C 40.2836720 37.4220270 38.4659112

45 H 40.0560076 37.6141438 37.4098374

46 H 37.4909400 37.8089573 37.4121668

47 C 40.1264571 36.0457838 39.0352297

48 H 40.6251607 36.0048438 40.0154568

49 H 39.0591779 35.8217987 39.2547053

50 C 40.7046823 34.9248603 38.1708908

51 H 41.5800759 35.2681664 37.6024844

52 H 41.0170958 34.0702927 38.7860293

53 N 39.7068592 34.3836069 37.1850727

54 H 39.0769184 33.6939696 37.6453233

55 H 40.2091399 33.8779955 36.4229195

56 H 39.1361113 35.1175096 36.7201187

57 C 40.7410790 41.0250567 39.8860253

58 O 41.8076003 41.6497720 40.0851864

59 O 39.6602215 41.1631946 40.4994801

60 H 42.9165216 40.0139919 38.7442981

61 H 42.1930619 40.9612506 37.6495653

62 H 42.2677547 39.3031715 37.3549516

63 H 39.3760129 44.8985933 37.3227276

64 H 33.8545301 45.0265890 35.2503472

Hydrox-3R[1]-Iso1-IM

QM(B1)/MM Energy = -3358.754661577947 a.u.

QM(B2)/MM Energy = -3360.886415000000 a.u.

QM(B3)/MM Energy = -3360.361730257459 a.u.

1 C 39.4332394 44.0187877 38.1012687

2 H 40.3862919 43.4844008 37.9778341

3 H 39.5100401 44.5695851 39.0495332

4 C 38.3173483 43.0234725 38.1453297

5 N 37.1667017 43.1684885 38.8896631

6 H 36.9470515 43.9905719 39.4648830

7 C 36.3806558 42.0928727 38.6787291

8 H 35.4051934 41.9435738 39.1378665

9 N 36.9711733 41.2469846 37.8355578

10 C 38.1737600 41.8220659 37.4933895

11 H 38.8425642 41.3376210 36.7878049

12 C 33.3954719 44.1687381 34.8123118

13 H 33.1333963 44.4196329 33.7711662

14 H 32.4478039 43.9652964 35.3316148

15 C 34.2574881 42.9508176 34.8750696

16 N 35.3179462 42.6896517 34.0243318

17 H 35.6264514 43.2770886 33.2404853

18 C 35.8855674 41.5254788 34.3877752

19 H 36.7671167 41.0881678 33.9239595

20 N 35.2386938 41.0069237 35.4240951

21 C 34.2204985 41.8807533 35.7371596

22 H 33.5479791 41.6947625 36.5704894

23 Cl 38.0308479 39.1934618 35.5182691

24 Fe 36.0032786 39.5973901 36.7781869

25 O 35.5560531 37.8354428 36.3356330

26 O 34.1701250 37.9250921 39.4041998

27 C 33.8323976 38.7823281 38.6044892

28 O 34.6683707 39.6968375 38.1450144

29 C 32.4004852 38.9344779 38.1270108

30 C 31.7292739 40.1784053 38.7383390

31 C 31.6103782 40.0012838 40.2550502

32 O 32.3506895 40.7121531 40.9950611

33 O 30.8065027 39.1233360 40.6544885

34 H 32.4089650 38.9980107 37.0262938

35 H 31.8345907 38.0462051 38.4341093

36 H 32.3007008 41.0918696 38.5129272

37 H 30.7177823 40.2683510 38.3181735

38 N 42.1099497 40.0149344 38.1276870

39 C 40.8056605 39.9065541 38.8541497

40 H 40.0213107 40.0937850 38.1095475

41 C 40.5574378 38.5473142 39.5068125

42 H 41.3772966 38.3183648 40.2128026

43 H 39.6773482 38.7116918 40.1600259

44 C 40.2968168 37.3906375 38.6026023

45 H 39.9320012 37.5844207 37.5867947

46 H 36.0823328 37.6273188 35.5474714

47 C 40.1538425 36.0227544 39.1950265

48 H 40.6782382 35.9885329 40.1614467

49 H 39.0911556 35.8030354 39.4385726

50 C 40.7054342 34.8887557 38.3317152

51 H 41.6250949 35.1957813 37.8144496

52 H 40.9378385 34.0035076 38.9396388

53 N 39.7256875 34.4345508 37.2884920

54 H 39.0208305 33.7903102 37.6969060

55 H 40.2245686 33.9045265 36.5400165

56 H 39.2582290 35.2186409 36.7897755

57 C 40.7571572 41.0189479 39.9293556

58 O 41.8216761 41.6505361 40.1184341

59 O 39.6738240 41.1659565 40.5361160

60 H 42.9318680 40.0245802 38.7692722

61 H 42.1723240 40.9424162 37.6723888

62 H 42.2759275 39.2838945 37.4022278

63 H 39.3289861 44.7660891 37.3146691

64 H 33.8328986 45.0770864 35.2265924

Hydrox-3R[1]-Iso1-TS-Cl

QM(B1)/MM Energy = -3358.744861563385 a.u.

QM(B2)/MM Energy = -3360.865990000000 a.u.

QM(B3)/MM Energy = -3360.347896372931 a.u.

1 C 39.4606816 44.0771042 38.1135599

2 H 40.4092174 43.5368254 37.9805219

3 H 39.5526773 44.6348759 39.0563064

4 C 38.3406499 43.0880879 38.1770931

5 N 37.1812819 43.2596719 38.8997737

6 H 36.9627299 44.0922842 39.4595586

7 C 36.3895323 42.1826343 38.7015935

8 H 35.4077562 42.0502539 39.1531294

9 N 36.9878956 41.3119566 37.8973731

10 C 38.1962275 41.8677726 37.5596179

11 H 38.8893063 41.3538066 36.9002655

12 C 33.5073739 44.0402440 34.8549469

13 H 33.2075685 44.2569322 33.8159247

14 H 32.5825725 43.7918730 35.3973346

15 C 34.4433681 42.8735573 34.9093622

16 N 35.4599227 42.6371160 34.0017265

17 H 35.7003584 43.2220320 33.1935563

18 C 36.0908907 41.4960805 34.3573279

19 H 36.9334930 41.0805565 33.8064923

20 N 35.5394469 40.9767867 35.4415763

21 C 34.5076360 41.8193219 35.7920868

22 H 33.8896039 41.6155607 36.6631463

23 Cl 38.3265791 38.3932666 37.4577602

24 Fe 36.1966351 39.5035660 36.9374607

25 O 35.8381008 38.0066052 35.8589006

26 O 34.2533608 37.6618811 39.2089663

27 C 33.8716610 38.6278964 38.5604439

28 O 34.6648220 39.5840635 38.1457123

29 C 32.4060401 38.8196306 38.1889053

30 C 31.7963930 40.1066118 38.7676846

31 C 31.6436689 39.9733659 40.2844511

32 O 32.3527485 40.7148214 41.0258981

33 O 30.8369537 39.1001021 40.6911437

34 H 32.3527062 38.8432241 37.0859716

35 H 31.8318381 37.9586434 38.5526462

36 H 32.4153992 40.9833946 38.5274025

37 H 30.7930062 40.2426717 38.3380293

38 N 42.1944789 40.1765543 38.1422200

39 C 40.8915124 40.0129177 38.8587140

40 H 40.1002401 40.1994080 38.1252806

41 C 40.6725616 38.6256217 39.4507467

42 H 41.5166928 38.3753483 40.1247894

43 H 39.7972593 38.7098250 40.1151064

44 C 40.4508492 37.4984497 38.5008674

45 H 40.9363868 37.5249955 37.5214521

46 H 34.9656861 38.0841339 35.4488984

47 C 40.1348723 36.1557563 39.0883031

48 H 40.6575763 36.0782599 40.0579655

49 H 39.0636971 36.0938476 39.3505509

50 C 40.5525380 34.9569864 38.2435983

51 H 41.4652582 35.1734021 37.6705201

52 H 40.7635212 34.0856096 38.8775896

53 N 39.5000829 34.5176358 37.2644255

54 H 38.8676288 33.8122146 37.6905472

55 H 39.9758518 34.0571292 36.4563260

56 H 38.9417739 35.2990625 36.8724371

57 C 40.8114925 41.0788824 39.9722096

58 O 41.8602615 41.7333176 40.1782987

59 O 39.7262631 41.1730693 40.5832213

60 H 43.0178680 40.1704975 38.7818804

61 H 42.2369436 41.1195259 37.7173904

62 H 42.3776870 39.4802410 37.3887027

63 H 39.3493074 44.8189282 37.3227628

64 H 33.8903845 44.9845615 35.2417731

Hydrox-3R[1]-Iso1-PC-Cl

QM(B1)/MM Energy = -3358.785648680046 a.u.

QM(B2)/MM Energy = -3360.922016000000 a.u.

QM(B3)/MM Energy = -3360.388166294203 a.u.

1 C 39.4349422 44.0203387 38.0992806

2 H 40.3871045 43.4857946 37.9692049

3 H 39.5157386 44.5669194 39.0497997

4 C 38.3148056 43.0288695 38.1415124

5 N 37.1408145 43.2168034 38.8339132

6 H 36.9178969 44.0535119 39.3854502

7 C 36.3406720 42.1485089 38.6175516

8 H 35.3413315 42.0389279 39.0356205

9 N 36.9460526 41.2641448 37.8328295

10 C 38.1718187 41.8048420 37.5254422

11 H 38.8716590 41.2854918 36.8759900

12 C 33.4495740 44.1614747 34.7770381

13 H 33.1533060 44.4222225 33.7469221

14 H 32.5197904 43.9200353 35.3130659

15 C 34.3511556 42.9691398 34.7963728

16 N 35.4090047 42.7598982 33.9320174

17 H 35.7032442 43.3681204 33.1610044

18 C 36.0017485 41.5913203 34.2642933

19 H 36.8743782 41.1942377 33.7477823

20 N 35.3823170 41.0262432 35.2852121

21 C 34.3466254 41.8679627 35.6223978

22 H 33.6820797 41.6279371 36.4491428

23 Cl 39.3292885 37.9005959 37.2483601

24 Fe 36.0458966 39.6116915 36.7817750

25 O 36.8732725 38.2544734 35.7290160

26 O 34.2274668 37.7412271 38.9631215

27 C 33.7138047 38.6809602 38.3580912

28 O 34.3852432 39.6598507 37.8250647

29 C 32.2051331 38.7934742 38.1783839

30 C 31.6214300 40.0748661 38.7946276

31 C	31.5629828	39.9413187	40.3153232
32 O	32.2653672	40.7232685	41.0208213
33 O	30.8258896	39.0276060	40.7645856
34 H	32.0133231	38.7848291	37.0917779
35 H	31.7147860	37.9231556	38.6312862
36 H	32.2109601	40.9579075	38.5097184
37 H	30.5903141	40.1996892	38.4345751
38 N	42.2134731	40.1974703	38.1899662
39 C	40.9166406	39.9953454	38.9088196
40 H	40.1263178	40.1810254	38.1736765
41 C	40.7303284	38.5952538	39.4866792
42 H	41.5503660	38.3651389	40.1863361
43 H	39.8104876	38.6385803	40.0876684
44 C	40.6229171	37.4614091	38.4734638
45 H	41.5475885	37.3711859	37.8883298
46 H	36.6239546	38.3421953	34.7999339
47 C	40.3029184	36.1065773	39.1029152
48 H	40.9181427	36.0132088	40.0082967
49 H	39.2562688	36.0803815	39.4452136
50 C	40.6336384	34.9052228	38.2222549
51 H	41.5494083	35.0880129	37.6417651
52 H	40.8097301	34.0147079	38.8397881
53 N	39.5536936	34.5233473	37.2421975
54 H	38.9650289	33.7639174	37.6375320
55 H	40.0076484	34.1434252	36.3798949
56 H	38.9376899	35.3062276	36.9529205
57 C	40.7931167	41.0545564	40.0236046
58 O	41.8295106	41.7210839	40.2556346
59 O	39.6889295	41.1346948	40.5992317
60 H	43.0446224	40.1821819	38.8187956
61 H	42.2429113	41.1510341	37.7867995
62 H	42.3990356	39.5268107	37.4130081
63 H	39.3321689	44.7734609	37.3180561
64 H	33.8635994	45.0790929	35.1949220

Hydrox-3R^1-Iso2-TS

QM(B1)/MM Energy = -3358.743165812407 a.u.

QM(B2)/MM Energy = -3360.876767000000 a.u.

QM(B3)/MM Energy = -3360.352239775147 a.u.

1 C	39.4059139	44.0328443	38.1226752
2 H	40.3498525	43.4815828	38.0003385
3 H	39.4917990	44.5860463	39.0688251
4 C	38.2698453	43.0603105	38.1641120
5 N	37.0975946	43.2540355	38.8604399
6 H	36.8894051	44.0835517	39.4285982
7 C	36.2814788	42.2024015	38.6264973
8 H	35.2892914	42.0932394	39.0613544
9 N	36.8754546	41.3274819	37.8226590
10 C	38.1110221	41.8504552	37.5291982
11 H	38.8138080	41.3158646	36.8967875
12 C	33.3919106	44.2108027	34.8182876
13 H	33.1165105	44.4503839	33.7781897
14 H	32.4532825	43.9968280	35.3494748
15 C	34.2781382	43.0111747	34.8824571
16 N	35.2882880	42.7313444	33.9825369
17 H	35.5544103	43.2969367	33.1684998
18 C	35.8904785	41.5861883	34.3630832
19 H	36.7234259	41.1362597	33.8258942
20 N	35.3243168	41.1107256	35.4594285
21 C	34.3148799	41.9799877	35.7927293
22 H	33.6852066	41.8154042	36.6632886
23 Cl	38.1059214	38.5048761	37.1987597
24 Fe	36.0549020	39.5656896	36.7620525
25 O	35.7076380	38.2496229	35.4774224
26 O	34.0967080	37.8621197	39.4280805

27 C 33.7660828 38.6513123 38.5594780
28 O 34.6251310 39.4619483 37.9705812
29 C 32.3249888 38.8500616 38.1294042
30 C 31.7324116 40.1389027 38.7285702
31 C 31.6152260 39.9972419 40.2487363
32 O 32.3531746 40.7263766 40.9725681
33 O 30.8082335 39.1314340 40.6672982
34 H 32.2924792 38.8886119 37.0286923
35 H 31.7301456 37.9998091 38.4858871
36 H 32.3476758 41.0170261 38.4800130
37 H 30.7231932 40.2775086 38.3159595
38 N 42.1768844 40.0554267 38.1581304
39 C 40.8843221 39.8944556 38.8972976
40 H 40.0806077 40.0034177 38.1584220
41 C 40.7272550 38.5410015 39.5896859
42 H 41.5231916 38.4392148 40.3585851
43 H 39.7912975 38.6223609 40.1702387
44 C 40.6737098 37.3246673 38.7306031
45 H 40.9739900 37.3611453 37.6801421
46 H 36.4997419 37.9849306 34.9870868
47 C 40.2496537 36.0246135 39.3377910
48 H 40.6228660 35.9630028 40.3751486
49 H 39.1441047 35.9792462 39.4415480
50 C 40.7395518 34.7911086 38.5834891
51 H 41.7285747 34.9711714 38.1399459
52 H 40.8204850 33.9154480 39.2417199
53 N 39.8060904 34.3963716 37.4724203
54 H 39.0741234 33.7452886 37.8167796
55 H 40.3153930 33.8941131 36.7111864
56 H 39.3778216 35.2090139 36.9888495
57 C 40.7947837 41.0267074 39.9445695
58 O 41.8471516 41.6818196 40.1343983
59 O 39.7040853 41.1685555 40.5377208
60 H 43.0056683 40.0751091 38.7899366
61 H 42.2047237 40.9908832 37.7170093
62 H 42.3569316 39.3435746 37.4188350
63 H 39.3121551 44.7781006 37.3328212
64 H 33.8263236 45.1249243 35.2229285

Hydrox-3R[1]-Iso2-IM

QM(B1)/MM Energy = -3358.749747910079 a.u.

QM(B2)/MM Energy = -3360.881219000000 a.u.

QM(B3)/MM Energy = -3360.355054605910 a.u.

1 C 39.4041440 44.1254473 38.1516822
2 H 40.3194762 43.5310626 38.0128685
3 H 39.5413280 44.6938412 39.0820456
4 C 38.2273126 43.2079997 38.2459044
5 N 37.1042384 43.4376021 39.0089961
6 H 36.9626769 44.2662963 39.5979483
7 C 36.2211922 42.4389396 38.7979690
8 H 35.2525320 42.3648223 39.2910414
9 N 36.7304873 41.5642150 37.9403568
10 C 37.9762312 42.0276077 37.5923882
11 H 38.6079550 41.4884713 36.8927702
12 C 33.4027396 44.2999659 34.7757770
13 H 33.1566210 44.5462781 33.7297610
14 H 32.4485139 44.0967453 35.2818285
15 C 34.2682682 43.0880893 34.8671033
16 N 35.3338441 42.8084200 34.0306382
17 H 35.6378907 43.3665160 33.2254219
18 C 35.9183492 41.6686012 34.4549313
19 H 36.7903736 41.2000316 34.0051653
20 N 35.2831517 41.1925779 35.5116121
21 C 34.2479572 42.0540879 35.7738858
22 H 33.5600631 41.8894438 36.5999401

23 Cl 37.4864273 38.6083255 38.4388446

24 Fe 36.1758419 39.7859590 36.9376431

25 O 37.2765815 39.3193528 35.4655748

26 O 34.1756748 37.8397084 39.0382109

27 C 33.7021876 38.5258438 38.1444735

28 O 34.4183522 39.1129500 37.2078263

29 C 32.2126013 38.8091163 38.0257975

30 C 31.8107178 40.1300094 38.7094617

31 C 31.7359124 39.9884397 40.2334262

32 O 32.4491691 40.7507625 40.9449702

33 O 30.9454161 39.1137749 40.6711480

34 H 31.9588091 38.8527274 36.9566871

35 H 31.6470770 37.9995256 38.5053095

36 H 32.4901801 40.9537320 38.4406067

37 H 30.7986756 40.3909056 38.3654242

38 N 42.1515312 40.0793282 38.1315371

39 C 40.8577127 39.9220940 38.8673353

40 H 40.0517808 40.0514541 38.1323928

41 C 40.7013114 38.5586978 39.5382823

42 H 41.5183281 38.4205608 40.2748501

43 H 39.7823566 38.6506825 40.1467756

44 C 40.5868608 37.3696260 38.6448854

45 H 40.5464982 37.4968077 37.5576008

46 H 38.1759812 39.1074571 35.7622476

47 C 40.2571949 36.0394840 39.2449168

48 H 40.6725625 35.9831176 40.2639255

49 H 39.1586426 35.9347748 39.3874791

50 C 40.7836376 34.8415823 38.4551243

51 H 41.7389242 35.0807070 37.9675060

52 H 40.9449467 33.9712684 39.1055853

53 N 39.8275773 34.3903214 37.3878823

54 H 39.1065669 33.7524970 37.7805797

55 H 40.3265484 33.8514512 36.6451271

56 H 39.3810540 35.1674969 36.8657272

57 C 40.7720663 41.0462207 39.9270116

58 O 41.8273170 41.6927308 40.1241646

59 O 39.6797167 41.1880015 40.5177465

60 H 42.9748658 40.1059060 38.7713059

61 H 42.1808827 41.0082843 37.6773737

62 H 42.3337828 39.3534376 37.4057512

63 H 39.3116222 44.8579651 37.3498564

64 H 33.8391645 45.2065534 35.1949425

Hydrox-3R^1-Iso2-TS-Cl

QM(B1)/MM Energy = -3358.741038509481 a.u.

QM(B2)/MM Energy = -3360.871730000000 a.u.

QM(B3)/MM Energy = -3360.352993924920 a.u.

1 C 39.3876716 44.0938233 38.1542533

2 H 40.3094463 43.5085089 38.0182475

3 H 39.5152701 44.6588826 39.0883609

4 C 38.2193469 43.1636134 38.2392883

5 N 37.0834440 43.3859813 38.9840762

6 H 36.9191258 44.2216448 39.5567495

7 C 36.2245691 42.3618060 38.7787339

8 H 35.2503018 42.2827998 39.2605057

9 N 36.7601721 41.4767946 37.9493932

10 C 37.9972487 41.9661845 37.6050593

11 H 38.6443351 41.4330834 36.9144553

12 C 33.4040383 44.2739855 34.7950822

13 H 33.1520719 44.5172321 33.7496769

14 H 32.4525467 44.0696409 35.3061930

15 C 34.2747689 43.0651852 34.8834403

16 N 35.3201925 42.7772340 34.0253417

17 H 35.6150101 43.3336800 33.2159727

18 C 35.9105077 41.6375353 34.4449124

19 H	36.7701257	41.1596423	33.9807720
20 N	35.3003379	41.1703286	35.5199572
21 C	34.2752591	42.0380391	35.7990696
22 H	33.6061977	41.8844030	36.6427223
23 Cl	37.8422472	38.6293323	38.3497374
24 Fe	36.2538508	39.6976610	36.8434178
25 O	37.3487569	39.2733555	35.3498449
26 O	34.3204323	37.9353968	39.2032776
27 C	33.8442315	38.4658265	38.2037962
28 O	34.5587078	38.8711951	37.1844163
29 C	32.3520211	38.7308672	38.0624735
30 C	31.9282864	40.0692712	38.7015827
31 C	31.7969023	39.9729386	40.2255850
32 O	32.4958373	40.7464363	40.9410562
33 O	30.9776300	39.1245167	40.6604701
34 H	32.1037862	38.7354937	36.9918344
35 H	31.7902415	37.9323307	38.5659660
36 H	32.6174498	40.8862285	38.4353247
37 H	30.9293997	40.3243882	38.3151315
38 N	42.1563786	40.1149290	38.0956258
39 C	40.8705313	39.9863525	38.8491421
40 H	40.0549207	40.1725144	38.1404472
41 C	40.6665987	38.6125500	39.4895913
42 H	41.6125921	38.2885060	39.9633661
43 H	39.9691690	38.7662821	40.3288420
44 C	40.1147267	37.5185082	38.6335311
45 H	40.2762885	37.5580206	37.5510932
46 H	38.2646745	39.0894907	35.6056110
47 C	39.8870046	36.1702507	39.2619772
48 H	40.2421941	36.1902288	40.3050660
49 H	38.8048193	35.9503344	39.3318701
50 C	40.5978886	35.0170337	38.5417384
51 H	41.5575389	35.3391535	38.1133698
52 H	40.8047214	34.1881118	39.2325014
53 N	39.7680580	34.4432036	37.4317294
54 H	39.0724767	33.7624770	37.7999731
55 H	40.3510260	33.9151300	36.7432341
56 H	39.2991075	35.1590718	36.8433127
57 C	40.8421880	41.0898271	39.9337101
58 O	41.9022674	41.7337780	40.0949462
59 O	39.7742000	41.2181489	40.5702872
60 H	42.9862462	40.1377798	38.7288488
61 H	42.1892312	41.0406054	37.6336885
62 H	42.3213555	39.3813938	37.3735434
63 H	39.2988890	44.8291631	37.3545908
64 H	33.8380611	45.1840661	35.2091409

Hydrox-3R^1-Iso2-PC-Cl

QM(B1)/MM Energy = -3358.791705334643 a.u.

QM(B2)/MM Energy = -3360.925957000000 a.u.

QM(B3)/MM Energy = -3360.391042706502 a.u.

1 C	39.3060971	43.9843230	38.1420290
2 H	40.2217061	43.3891174	38.0044573
3 H	39.4389904	44.5366163	39.0836348
4 C	38.1216535	43.0706495	38.2187908
5 N	36.9947219	43.3242327	38.9667863
6 H	36.8529730	44.1667112	39.5355546
7 C	36.1124911	42.3167009	38.7722269
8 H	35.1381509	42.2668940	39.2582430
9 N	36.6198762	41.4070719	37.9487573
10 C	37.8697463	41.8693404	37.5963030
11 H	38.4897858	41.3226093	36.8901679
12 C	33.3464553	44.4465167	34.6805418
13 H	33.1158380	44.7414776	33.6438798
14 H	32.3836809	44.2526630	35.1716097

15 C 34.1639568 43.2029201 34.7390789
16 N 35.2501005 42.9118157 33.9372907
17 H 35.6209399 43.4845476 33.1725675
18 C 35.7711866 41.7333326 34.3435621
19 H 36.6518353 41.2528818 33.9215391
20 N 35.0741875 41.2478824 35.3549477
21 C 34.0669504 42.1411999 35.6073457
22 H 33.3405587 41.9834711 36.4022537
23 Cl 39.7140989 37.5518161 36.9430024
24 Fe 35.8978784 39.8344649 36.6731835
25 O 37.2833943 39.2777195 35.5096856
26 O 34.1081398 38.2611003 39.2650856
27 C 33.6010015 38.5911946 38.1910053
28 O 34.2831492 38.8083545 37.1041806
29 C 32.1052942 38.8317138 38.0720700
30 C 31.6959214 40.1432524 38.7658791
31 C 31.6799107 40.0014575 40.2893523
32 O 32.3759200 40.7996666 40.9806650
33 O 30.9398365 39.0966674 40.7517258
34 H 31.8339216 38.8590814 37.0082861
35 H 31.5585678 38.0185861 38.5702952
36 H 32.3431784 40.9852015 38.4708693
37 H 30.6654867 40.3802411 38.4610550
38 N 42.1675375 40.0880750 38.0416400
39 C 40.8637363 39.9274380 38.7601101
40 H 40.0804326 40.1256811 38.0169697
41 C 40.6406326 38.5440431 39.3721369
42 H 41.3671568 38.3814723 40.1883545
43 H 39.6507969 38.5856023 39.8486720
44 C 40.7332569 37.3294334 38.4557335
45 H 41.7525007 37.2205783 38.0652288
46 H 38.1939517 39.1679197 35.8099666
47 C 40.3228547 36.0275132 39.1415062
48 H 40.7934190 36.0154362 40.1353196
49 H 39.2359245 36.0209945 39.3251299
50 C 40.7750228 34.7691586 38.4025426
51 H 41.7206375 34.9508430 37.8715653
52 H 40.9552898 33.9486683 39.1098446
53 N 39.7890165 34.2452117 37.4002632
54 H 39.1832740 33.5135700 37.8222200
55 H 40.2881786 33.7924920 36.6029797
56 H 39.1728678 34.9508224 36.9437623
57 C 40.7944185 41.0006913 39.8785428
58 O 41.8537159 41.6355144 40.0867548
59 O 39.7120099 41.1118069 40.4890808
60 H 42.9920887 40.0766584 38.6805255
61 H 42.2085576 41.0386003 37.6322358
62 H 42.3330925 39.4030317 37.2729943
63 H 39.2414881 44.7355374 37.3549015
64 H 33.8127555 45.3180731 35.1399116

Hydrox-3R[1]-Iso2-TS-OH

QM(B1)/MM Energy = -3358.718649390370 a.u.

QM(B2)/MM Energy = -3360.844996000000 a.u.

QM(B3)/MM Energy = -3360.325637793396 a.u.

1 C 39.4099555 44.1309881 38.2192268
2 H 40.2973864 43.5005645 38.0538050
3 H 39.6220165 44.7276082 39.1180519
4 C 38.2116103 43.2589103 38.4307508
5 N 37.1748630 43.5358681 39.2987632
6 H 37.0886037 44.4022735 39.8400523
7 C 36.2802433 42.5231373 39.2504646
8 H 35.3604995 42.4725180 39.8352457
9 N 36.6965615 41.6004049 38.3966870
10 C 37.8877089 42.0453670 37.8782463

11 H	38.4330428	41.4659167	37.1384319
12 C	33.5594982	44.1301644	34.8557356
13 H	33.3336310	44.3153877	33.7929179
14 H	32.6008300	43.9003722	35.3448589
15 C	34.4924914	42.9734345	35.0257975
16 N	35.5330008	42.6476376	34.1759282
17 H	35.7845127	43.1531698	33.3190616
18 C	36.1720074	41.5682263	34.6917190
19 H	37.0204595	41.0836072	34.2120901
20 N	35.6045257	41.1806839	35.8205072
21 C	34.5494674	42.0374288	36.0298937
22 H	33.8843990	41.9374513	36.8840899
23 Cl	35.4653446	38.8127083	39.6894048
24 Fe	35.9334647	39.7958069	37.5874515
25 O	37.6281867	38.7897318	37.4692851
26 O	33.8092860	36.8021553	37.1863537
27 C	33.6271214	38.0044750	37.2936184
28 O	34.4560978	38.8856198	36.7620362
29 C	32.3661139	38.5506494	37.9759864
30 C	32.3889622	39.9764920	38.5594823
31 C	32.0147928	40.1027216	40.0525246
32 O	32.5613061	41.0291961	40.7078792
33 O	31.1564888	39.2959418	40.5109200
34 H	31.5708671	38.4653761	37.2152021
35 H	32.0852603	37.8271836	38.7528638
36 H	33.3723213	40.4476668	38.4372775
37 H	31.6692305	40.6203462	38.0223138
38 N	42.1897243	40.1393316	37.9288243
39 C	40.8453498	40.1586111	38.5897955
40 H	40.1672440	40.6417599	37.8749827
41 C	40.2788718	38.7804518	38.9157662
42 H	41.0224867	38.1767986	39.4749866
43 H	39.4644098	38.9640637	39.6387118
44 C	39.7413620	37.9960890	37.7654312
45 H	39.9897508	38.3055947	36.7463659
46 H	37.4387060	38.2053903	38.2231701
47 C	39.3046734	36.5784953	37.9688437
48 H	39.0948905	36.3771488	39.0316017
49 H	38.3502231	36.4598585	37.4341363
50 C	40.2769994	35.5343339	37.4040768
51 H	40.7910611	35.9206007	36.5153337
52 H	41.0457644	35.2217726	38.1254260
53 N	39.5384896	34.3119644	36.9478875
54 H	39.2004371	33.6919414	37.7156796
55 H	40.1649153	33.7423900	36.3380315
56 H	38.7248557	34.5888516	36.3602108
57 C	40.9338579	41.0714705	39.8372448
58 O	42.0231862	41.6498306	40.0320347
59 O	39.8993614	41.1600490	40.5336944
60 H	42.9766472	40.0735579	38.6124574
61 H	42.3456368	41.0614233	37.4875063
62 H	42.3117426	39.4035146	37.2009424
63 H	39.3137943	44.8466460	37.4027381
64 H	33.9271216	45.0783645	35.2479558

Hydrox-3R^1-Iso2-PC-OH

QM(B1)/MM Energy = -3358.797784714111 a.u.

QM(B2)/MM Energy = -3360.921868000000 a.u.

QM(B3)/MM Energy = -3360.387370131078 a.u.

1 C	39.3402772	44.0539618	38.2434472
2 H	40.2095645	43.3972400	38.0884305
3 H	39.5486564	44.6365977	39.1523980
4 C	38.1102426	43.2187135	38.4216713
5 N	37.0640912	43.5411850	39.2620732
6 H	37.0051210	44.4071949	39.8077821

7 C 36.1206453 42.5785495 39.1773361
8 H 35.1894352 42.5673809 39.7453367
9 N 36.5133993 41.6399023 38.3281209
10 C 37.7467001 42.0240740 37.8497431
11 H 38.2976635 41.4133496 37.1371894
12 C 33.4945616 44.2523437 34.7800350
13 H 33.2822849 44.4741942 33.7216559
14 H 32.5259651 44.0452575 35.2577534
15 C 34.3753295 43.0557509 34.9300172
16 N 35.4539216 42.7339530 34.1287536
17 H 35.7614540 43.2565294 33.3002853
18 C 36.0313645 41.6148508 34.6266429
19 H 36.8984559 41.1302132 34.1817684
20 N 35.3846909 41.1949580 35.7003297
21 C 34.3433531 42.0739896 35.8889579
22 H 33.6297363 41.9655105 36.7032891
23 Cl 36.4136175 38.4733221 39.2342348
24 Fe 35.6168271 39.9361825 37.4810672
25 O 38.6185383 38.7885821 37.2204629
26 O 33.8994019 36.5715206 37.1886431
27 C 33.7095064 37.7674568 37.3313110
28 O 34.4282994 38.6775141 36.7030830
29 C 32.5893237 38.3048508 38.2367202
30 C 32.8799322 39.6387799 38.9309858
31 C 32.0814462 39.9941176 40.2029314
32 O 32.5310292 40.9733536 40.8605264
33 O 31.0854805 39.2913617 40.5174706
34 H 31.6765778 38.3949155 37.6215717
35 H 32.3689590 37.5200152 38.9695986
36 H 33.9294518 39.6765546 39.2664968
37 H 32.7421342 40.4870211 38.2376353
38 N 42.1882657 40.2025614 37.7701161
39 C 40.8858913 40.1174832 38.5078553
40 H 40.1313406 40.5467371 37.8370282
41 C 40.4525695 38.6825521 38.8005329
42 H 41.2990779 38.0695791 39.1494083
43 H 39.7344214 38.7399047 39.6337815
44 C 39.7365890 38.0012171 37.6218157
45 H 40.3969250 37.9678807 36.7398814
46 H 37.9966380 38.7671833 37.9810154
47 C 39.2766035 36.5683007 37.9715277
48 H 39.0120943 36.4920466 39.0373787
49 H 38.3319254 36.4266974 37.4293721
50 C 40.2456866 35.4564446 37.5638084
51 H 40.9310206 35.7954368 36.7745365
52 H 40.8607008 35.0781623 38.3926112
53 N 39.5001206 34.2905186 36.9821928
54 H 39.0827261 33.6529023 37.6941921
55 H 40.1454211 33.7229360 36.3913572
56 H 38.7469563 34.6266980 36.3483918
57 C 40.9461807 41.0331670 39.7552959
58 O 42.0124457 41.6632331 39.9404631
59 O 39.9124037 41.0905402 40.4606241
60 H 42.9957983 40.3092114 38.4246583
61 H 42.1808650 41.0496375 37.1756356
62 H 42.3730131 39.3966686 37.1373152
63 H 39.2804371 44.7799750 37.4326507
64 H 33.8994859 45.1728613 35.2004523

Chimera141-RC

QM(B1)/MM Energy = -3361.052951240781 a.u.

QM(B2)/MM Energy = -3363.181324000000 a.u.

QM(B3)/MM Energy = -3362.657278504609 a.u.

1 C 36.5059939 28.3085831 28.7443115
2 H 35.8367417 27.5023517 29.0659032

3 H 37.5231332 27.8925230 28.7332217

4 C 36.4144172 29.4353623 29.7190279

5 N 37.4430120 30.3062098 30.0040097

6 H 38.3575758 30.3071454 29.5359334

7 C 37.0090323 31.2046944 30.9139864

8 H 37.6229182 32.0086453 31.3140076

9 N 35.7525570 30.9422511 31.2439916

10 C 35.3654065 29.8491163 30.5063546

11 H 34.3551712 29.4532028 30.5621177

12 C 35.6736233 34.9073008 27.7778820

13 H 35.0231075 35.5023609 27.1148854

14 H 36.2777994 35.6313275 28.3444956

15 C 34.8662840 34.0829781 28.7302662

16 N 33.7269538 33.3691323 28.3990106

17 H 33.2691057 33.3254874 27.4821082

18 C 33.2875298 32.7260328 29.5005611

19 H 32.4154284 32.0775057 29.5339536

20 N 34.0714217 32.9918531 30.5316643

21 C 35.0494322 33.8441421 30.0715513

22 H 35.8141804 34.2370007 30.7354267

23 Cl 32.2587965 30.9710111 32.0297878

24 Fe 34.3114543 31.9752390 32.3906455

25 O 34.6802559 31.1518925 33.7278391

26 O 34.7140988 33.8206653 35.2507486

27 C 35.0589222 34.2455323 34.1574417

28 O 34.7704616 33.7012740 33.0008785

29 C 35.9384205 35.4779069 33.9970808

30 C 37.4151302 35.1137769 34.2927276

31 C 37.9050137 35.6676081 35.6446681

32 O 38.4726683 34.8757690 36.4432816

33 O 37.7319242 36.8971355 35.8162030

34 H 35.8171815 35.8675232 32.9767476

35 H 35.6218265 36.2591527 34.7028135

36 H 37.5886635 34.0269885 34.2453629

37 H 38.0622797 35.5789369 33.5292811

38 N 32.8187861 26.2264756 31.6340574

39 C 33.9738998 26.9252160 32.2847592

40 H 33.8706330 27.9866108 32.0177270

41 C 33.8981429 26.7909379 33.8070566

42 H 33.5999995 25.7611435 34.0561848

43 H 34.9143719 26.9277098 34.1972604

44 C 32.9606675 27.7938393 34.4952593

45 H 32.0229035 27.8988246 33.9250077

46 H 33.4301073 28.7939482 34.4850600

47 C 32.6519064 27.3526104 35.9282617

48 H 32.1150559 26.3885640 35.9109410

49 H 33.5966236 27.1560976 36.4651048

50 C 31.8760669 28.3434859 36.7806859

51 H 31.7788443 27.9684922 37.8088109

52 H 32.3672811 29.3270951 36.8273811

53 N 30.4743827 28.5882069 36.3016379

54 H 29.9336384 29.0197976 37.0779839

55 H 29.9850573 27.7095174 36.0184635

56 H 30.4020783 29.2235542 35.4823037

57 C 35.3124800 26.4337455 31.6779938

58 O 36.3457602 26.9316556 32.1838836

59 O 35.2745658 25.5860651 30.7532571

60 H 31.9405189 26.3374888 32.1842994

61 H 32.9541545 25.1973950 31.5553623

62 H 32.6473697 26.6244112 30.6921690

63 H 36.2782241 28.5794134 27.7133706

64 H 36.3307177 34.3509428 27.1094812

Chimera141-TS1

QM(B1)/MM Energy = -3361.016942934823 a.u.

QM(B2)/MM Energy = -3363.145002000000 a.u.

QM(B3)/MM Energy = -3362.627953979400 a.u.

1 C	36.5187270	28.2496963	28.6452443
2 H	35.8593536	27.4287632	28.9476474
3 H	37.5404572	27.8460492	28.6261334
4 C	36.4076929	29.3459369	29.6505825
5 N	37.4207597	30.2140742	29.9927974
6 H	38.3386495	30.2540585	29.5325060
7 C	36.9638099	31.0537790	30.9480983
8 H	37.5626173	31.8408098	31.4008030
9 N	35.7099866	30.7533049	31.2545200
10 C	35.3467008	29.7061381	30.4445715
11 H	34.3385326	29.3040107	30.4483850
12 C	35.6632047	34.8491107	27.9198728
13 H	35.0093006	35.4758528	27.2898038
14 H	36.2765435	35.5468390	28.5100526
15 C	34.8502591	33.9913390	28.8402870
16 N	33.6760372	33.3528180	28.4808574
17 H	33.2167636	33.3717469	27.5639490
18 C	33.2144921	32.6745807	29.5551081
19 H	32.3069116	32.0754959	29.5568595
20 N	34.0144778	32.8380017	30.5937443
21 C	35.0295445	33.6632141	30.1663835
22 H	35.8257720	33.9749284	30.8378117
23 Cl	31.9584995	30.9851392	31.9912172
24 Fe	34.1765927	31.5462092	32.5023227
25 O	34.3551038	30.2624956	33.6889937
26 O	34.6681207	33.6047971	35.4825822
27 C	35.0410063	33.8740150	34.3467638
28 O	34.8427414	33.1251864	33.2958385
29 C	35.8529764	35.1235743	34.0293938
30 C	37.3615014	34.8733891	34.2850633
31 C	37.8704625	35.5251049	35.5873365
32 O	38.4498468	34.7898674	36.4329297
33 O	37.6986504	36.7630549	35.6752628
34 H	35.6701661	35.4001854	32.9814665
35 H	35.5214437	35.9566679	34.6658400
36 H	37.6006233	33.7982718	34.2823010
37 H	37.9449341	35.3399817	33.4732712
38 N	32.8482447	26.2514157	31.6335054
39 C	34.0200758	26.9777257	32.2171990
40 H	33.9450521	28.0089448	31.8516280
41 C	33.9698629	27.0211068	33.7440853
42 H	33.6442885	26.0410129	34.1355354
43 H	35.0013723	27.1643811	34.0907607
44 C	33.0973217	28.1195751	34.3362760
45 H	32.1473301	28.2668063	33.7952911
46 H	33.6807421	29.1999644	34.0921596
47 C	32.9185905	27.9525869	35.8365676
48 H	32.5421845	26.9286070	36.0326325
49 H	33.9074770	27.9910803	36.3257274
50 C	32.0230270	28.9472993	36.5609272
51 H	32.1213188	28.8139273	37.6463500
52 H	32.2902892	29.9871826	36.3231197
53 N	30.5566056	28.7898065	36.2645643
54 H	30.0258777	29.0662954	37.1152404
55 H	30.2501643	27.8202956	36.0209434
56 H	30.2267238	29.3894553	35.4822306
57 C	35.3445614	26.4102484	31.6487287
58 O	36.3886081	26.8682349	32.1695166
59 O	35.2853620	25.5591609	30.7305618
60 H	31.9881676	26.3750328	32.2085089
61 H	32.9810418	25.2200458	31.5739208
62 H	32.6408030	26.6203570	30.6866801
63 H	36.2842691	28.5433389	27.6220773
64 H	36.3114114	34.3255259	27.2171962

Chimera141-IM1

QM(B1)/MM Energy = -3361.052105116099 a.u.

QM(B2)/MM Energy = -3363.180599000000 a.u.

QM(B3)/MM Energy = -3362.657083352852 a.u.

1 C	36.5099451	28.3170411	28.7368452
2 H	35.8467200	27.5069446	29.0612886
3 H	37.5304499	27.9092514	28.7289272
4 C	36.4080739	29.4468328	29.7074017
5 N	37.4370667	30.3130758	30.0054632
6 H	38.3522712	30.3178479	29.5383785
7 C	37.0007565	31.2012301	30.9236964
8 H	37.6167828	31.9985307	31.3339137
9 N	35.7411450	30.9414580	31.2474916
10 C	35.3560824	29.8584899	30.4912903
11 H	34.3450990	29.4628640	30.5348986
12 C	35.6912698	34.9209804	27.7439532
13 H	35.0471773	35.5152282	27.0738475
14 H	36.3002729	35.6449403	28.3052510
15 C	34.8760091	34.1124432	28.7016946
16 N	33.7427111	33.3913756	28.3687594
17 H	33.2925998	33.3319265	27.4492924
18 C	33.2921762	32.7690574	29.4795165
19 H	32.4200230	32.1195187	29.5077291
20 N	34.0608775	33.0525232	30.5154848
21 C	35.0414176	33.8963129	30.0504972
22 H	35.8009942	34.3041731	30.7131139
23 Cl	32.2333007	30.9379475	32.0666621
24 Fe	34.2829082	31.9510853	32.4581194
25 O	34.8521081	31.1826875	34.0237605
26 O	34.7693226	33.6756571	35.3373283
27 C	34.9685676	34.2310548	34.2470084
28 O	34.5492530	33.7813273	33.1086486
29 C	35.8325728	35.4744573	34.1435816
30 C	37.3171559	35.0833435	34.3698750
31 C	37.8967086	35.6528787	35.6786023
32 O	38.5138945	34.8669102	36.4463707
33 O	37.7392709	36.8843340	35.8482071
34 H	35.6767618	35.9284554	33.1557167
35 H	35.5416119	36.2103094	34.9065187
36 H	37.4616406	33.9919601	34.3438743
37 H	37.9335465	35.5097419	33.5597931
38 N	32.8261815	26.2483146	31.7558392
39 C	34.0081923	26.9763873	32.3121551
40 H	33.8763777	28.0239324	32.0072477
41 C	34.0502464	26.9344907	33.8430135
42 H	34.1147166	25.8904697	34.1944227
43 H	35.0169151	27.4052123	34.1014497
44 C	32.9493048	27.6620146	34.5409911
45 H	32.6530447	28.6438389	34.1541484
46 H	34.7580777	31.8076394	34.7728445
47 C	32.4920098	27.1855843	35.8800488
48 H	31.8506675	26.2873093	35.7723030
49 H	33.3615214	26.8229980	36.4621893
50 C	31.7827263	28.2012924	36.7620730
51 H	31.6422555	27.7936877	37.7725340
52 H	32.3510047	29.1387320	36.8524366
53 N	30.4140590	28.5675284	36.2755760
54 H	29.9028684	29.0499780	37.0420880
55 H	29.8634046	27.7249364	35.9978438
56 H	30.3959758	29.1941240	35.4464882
57 C	35.3220756	26.4628621	31.6727581
58 O	36.3709511	26.9905489	32.1109975
59 O	35.2552931	25.5644935	30.7999558
60 H	31.9770316	26.3713488	32.3509952

61 H 32.9590123 25.2184346 31.6770219

62 H 32.5989287 26.6264361 30.8175895

63 H 36.2789352 28.5803734 27.7046835

64 H 36.3437699 34.3515362 27.0821042

Chimera141-TS-Cl

QM(B1)/MM Energy = -3361.008842566401 a.u.

QM(B2)/MM Energy = -3363.135521000000 a.u.

QM(B3)/MM Energy = -3362.618208022402 a.u.

1 C 36.5096722 28.2672545 28.6843099

2 H 35.8541997 27.4443789 28.9912523

3 H 37.5331452 27.8669912 28.6723489

4 C 36.3984692 29.3699109 29.6851072

5 N 37.4304059 30.2084628 30.0451988

6 H 38.3459230 30.2442707 29.5811417

7 C 36.9893270 31.0329737 31.0226966

8 H 37.6089812 31.7951383 31.4912272

9 N 35.7289853 30.7588915 31.3267594

10 C 35.3457812 29.7402379 30.4883088

11 H 34.3297534 29.3565783 30.4875335

12 C 35.4929377 34.7140014 28.0342752

13 H 34.8324939 35.3469793 27.4154448

14 H 36.0650303 35.4077046 28.6693466

15 C 34.6778928 33.8104168 28.9140480

16 N 33.5724262 33.0810133 28.5076614

17 H 33.1565459 33.0681983 27.5696054

18 C 33.0806694 32.4252332 29.5872502

19 H 32.2138009 31.7688071 29.5401693

20 N 33.7881111 32.6903756 30.6708058

21 C 34.7771545 33.5602460 30.2650921

22 H 35.4894534 33.9649218 30.9787521

23 Cl 32.4602442 29.8209248 32.7359167

24 Fe 34.2936546 31.6467505 32.6986570

25 O 34.8270561 30.9206763 34.3593817

26 O 34.7981334 33.6330109 35.4908662

27 C 34.9318798 34.0894620 34.3414951

28 O 34.4368634 33.5616995 33.2785382

29 C 35.8064520 35.3148289 34.1037832

30 C 37.2927943 34.9532735 34.3528745

31 C 37.8673743 35.5658402 35.6446315

32 O 38.4951799 34.8069074 36.4330833

33 O 37.7066517 36.8013441 35.7814564

34 H 35.6404875 35.6710732 33.0773216

35 H 35.5288756 36.1274562 34.7916807

36 H 37.4502879 33.8633707 34.3620406

37 H 37.9089759 35.3624856 33.5330549

38 N 32.8874367 26.2833371 31.7196286

39 C 34.1018018 26.9961849 32.2169996

40 H 34.0216344 28.0178877 31.8359984

41 C 34.1779259 27.0766719 33.7443236

42 H 33.9236081 26.0940178 34.1707573

43 H 35.2464766 27.2559652 33.9273453

44 C 33.4573943 28.1640387 34.4870035

45 H 33.9817163 29.1259424 34.6194426

46 H 34.8627180 31.6385919 35.0172998

47 C 32.5323100 27.6994801 35.5525597

48 H 31.8120935 26.9500532 35.1797121

49 H 33.1971822 27.0827637 36.1986039

50 C 31.8463988 28.7275992 36.4500344

51 H 31.9376804 28.4359820 37.5043543

52 H 32.2784212 29.7320358 36.3383600

53 N 30.3810876 28.8264881 36.1897497

54 H 29.9120733 29.2212497 37.0297081

55 H 29.9389505 27.9023692 35.9846532

56 H 30.1456439 29.4332691 35.3798393

57 C	35.3958048	26.3916899	31.6199107
58 O	36.4579546	26.8592881	32.0966633
59 O	35.2998312	25.5136119	30.7344747
60 H	32.0403400	26.4683102	32.2983929
61 H	32.9757521	25.2452343	31.6846218
62 H	32.6597413	26.6251759	30.7661678
63 H	36.2769736	28.5416582	27.6554168
64 H	36.1800101	34.2600918	27.3201570

Chimera141-PC-Cl

QM(B1)/MM Energy = -3361.054587096595 a.u.

QM(B2)/MM Energy = -3363.184245000000 a.u.

QM(B3)/MM Energy = -3362.653075637759 a.u.

1 C	36.5299681	28.2648362	28.7298937
2 H	35.8338921	27.4807687	29.0511789
3 H	37.5287094	27.8068414	28.6977584
4 C	36.5068009	29.3817673	29.7213582
5 N	37.5638283	30.2361028	29.9447946
6 H	38.4528149	30.2217771	29.4332761
7 C	37.2109453	31.1199817	30.9016355
8 H	37.8654895	31.9073164	31.2706112
9 N	35.9823507	30.8714590	31.3267490
10 C	35.5284808	29.7993020	30.5956020
11 H	34.5214213	29.4083917	30.7163858
12 C	35.6418611	34.8504473	27.8888367
13 H	34.9666566	35.4419378	27.2467171
14 H	36.2394376	35.5799138	28.4560331
15 C	34.8650100	34.0046586	28.8506247
16 N	33.7367610	33.2656851	28.5393730
17 H	33.2714180	33.2159814	27.6260282
18 C	33.3138848	32.6470891	29.6678879
19 H	32.4451762	31.9911885	29.6936268
20 N	34.0929898	32.9461535	30.6947642
21 C	35.0565841	33.8005579	30.1975865
22 H	35.8180851	34.2146371	30.8538968
23 Cl	31.9466880	28.7061158	33.2634232
24 Fe	34.7355700	32.0089832	32.6276699
25 O	34.3330263	30.9171459	34.0982089
26 O	34.8119434	33.9486238	35.2081360
27 C	35.3963487	34.3872373	34.2054356
28 O	35.6125482	33.7186213	33.1255576
29 C	35.9874012	35.7908273	34.1583786
30 C	37.5237891	35.7405397	34.3219688
31 C	37.9470341	35.8449866	35.7984114
32 O	38.4594214	34.8484146	36.3707474
33 O	37.7646623	36.9706679	36.3268274
34 H	35.7260074	36.2214717	33.1782994
35 H	35.5528211	36.4212502	34.9451518
36 H	37.9268946	34.8286844	33.8566056
37 H	37.9665965	36.6123283	33.8117022
38 N	32.8456230	26.1072171	31.4689840
39 C	34.0003237	26.7807699	32.1462901
40 H	33.9406489	27.8281017	31.8261592
41 C	33.8357868	26.7101140	33.6683250
42 H	33.2061347	25.8435876	33.9156352
43 H	34.8197492	26.5367210	34.1259546
44 C	33.2441009	27.9587297	34.3351052
45 H	33.9610947	28.7946062	34.3942909
46 H	34.4072146	31.4911887	34.8782945
47 C	32.6730091	27.6120413	35.7062643
48 H	32.0191743	26.7253410	35.6329918
49 H	33.5285364	27.2932441	36.3276389
50 C	31.9500371	28.7266723	36.4583015
51 H	31.9949319	28.5410690	37.5390501
52 H	32.3965370	29.7113392	36.2566599

53 N 30.4842141 28.8206502 36.1346938

54 H 29.9828234 29.1774878 36.9724068

55 H 30.0608318 27.8988072 35.8836514

56 H 30.2620616 29.4552312 35.3439777

57 C 35.3534038 26.2692827 31.6088996

58 O 36.3604918 26.7114765 32.2121929

59 O 35.3516998 25.4929892 30.6246130

60 H 31.9523324 26.3497315 31.9511481

61 H 32.8951220 25.0671693 31.4952984

62 H 32.7787678 26.4284735 30.4872760

63 H 36.3076400 28.5516575 27.7020947

64 H 36.3025534 34.3295082 27.1958899

Chimera14^{1}-TS-OH

QM(B1)/MM Energy = -3361.033658808626 a.u.

QM(B2)/MM Energy = -3363.160176000000 a.u.

QM(B3)/MM Energy = -3362.643367707554 a.u.

1 C 36.5221519 28.2451990 28.6127506

2 H 35.8715537 27.4203037 28.9216065

3 H 37.5486761 27.8540515 28.6031915

4 C 36.3928492 29.3488843 29.6071900

5 N 37.4103900 30.1896687 29.9961146

6 H 38.3387115 30.2248775 29.5570920

7 C 36.9375477 31.0196075 30.9537267

8 H 37.5386841 31.7871794 31.4367390

9 N 35.6680356 30.7418343 31.2155679

10 C 35.3120833 29.7221774 30.3673814

11 H 34.2951139 29.3436413 30.3249489

12 C 35.6481811 34.8408346 27.9007185

13 H 35.0041463 35.4715976 27.2641544

14 H 36.2597856 35.5350768 28.4968043

15 C 34.8231443 33.9876862 28.8141448

16 N 33.6740483 33.3176126 28.4318891

17 H 33.2351669 33.3196072 27.5053878

18 C 33.1957022 32.6475385 29.5041234

19 H 32.3066027 32.0206376 29.4866397

20 N 33.9599081 32.8475658 30.5624228

21 C 34.9671166 33.6893014 30.1518560

22 H 35.7335173 34.0348936 30.8420840

23 Cl 31.9838263 30.6685227 31.8671756

24 Fe 34.0872162 31.5423577 32.4400637

25 O 34.4430826 30.3182766 33.9172359

26 O 34.7376545 32.9704786 35.5202191

27 C 34.8657742 33.6242680 34.4729360

28 O 34.4380251 33.2460640 33.3186224

29 C 35.6294390 34.9409550 34.4533329

30 C 37.1614418 34.6800970 34.4904258

31 C 37.8482213 35.4325631 35.6462063

32 O 38.4739963 34.7433142 36.4984919

33 O 37.7268908 36.6791921 35.6404669

34 H 35.3411351 35.5065209 33.5574089

35 H 35.3554850 35.5427965 35.3284393

36 H 37.3832806 33.6055802 34.5773445

37 H 37.6191071 35.0371310 33.5527736

38 N 32.8642521 26.3586142 31.7768116

39 C 34.0569823 27.1360177 32.2400169

40 H 33.9519429 28.1262325 31.7841659

41 C 34.1252335 27.3404759 33.7521497

42 H 34.0873367 26.3693379 34.2828390

43 H 35.1349681 27.7443302 33.9335350

44 C 33.1540964 28.2971576 34.3598723

45 H 32.4133315 28.8041072 33.7363379

46 H 34.3977288 30.8450439 34.7397775

47 C 32.9732323 28.1850329 35.8370247

48 H 32.6949881 27.1352778 36.0754426

49 H	33.9558422	28.3141940	36.3313434
50 C	31.9963204	29.1273047	36.5231384
51 H	32.0835056	29.0256303	37.6127063
52 H	32.2030350	30.1753304	36.2621928
53 N	30.5513612	28.8736336	36.2054816
54 H	29.9876230	29.1711503	37.0268291
55 H	30.2862605	27.8794783	36.0198526
56 H	30.2093951	29.4128820	35.3855332
57 C	35.3565348	26.5081016	31.6708863
58 O	36.4233256	27.0083468	32.0941436
59 O	35.2578283	25.5650816	30.8513414
60 H	32.0378855	26.4592146	32.4038604
61 H	33.0262261	25.3318631	31.7024036
62 H	32.5743772	26.7031674	30.8415784
63 H	36.2838919	28.5272928	27.5872154
64 H	36.2993650	34.3139724	27.2032639

Chimera14[1]-PC-OH

QM(B1)/MM Energy = -3361.127427214739 a.u.

QM(B2)/MM Energy = -3363.253631000000 a.u.

QM(B3)/MM Energy = -3362.716904481792 a.u.

1 C	36.4619144	28.4023767	28.9012097
2 H	35.7828436	27.6020974	29.2188972
3 H	37.4754853	27.9763787	28.9128133
4 C	36.3647341	29.5396584	29.8662118
5 N	37.3871918	30.4259553	30.1292118
6 H	38.2950115	30.4301765	29.6490182
7 C	36.9546960	31.3194183	31.0478778
8 H	37.5637968	32.1387241	31.4249209
9 N	35.7076795	31.0470882	31.4110771
10 C	35.3284272	29.9427812	30.6777816
11 H	34.3256551	29.5298177	30.7534073
12 C	35.7279981	34.9908218	27.6468105
13 H	35.1127233	35.5788212	26.9452501
14 H	36.3366985	35.7171730	28.2043703
15 C	34.8758634	34.2264995	28.6064118
16 N	33.7477937	33.5004005	28.2707365
17 H	33.3282710	33.3880106	27.3425656
18 C	33.2585578	32.9267221	29.3901596
19 H	32.3832928	32.2802287	29.4098572
20 N	34.0013046	33.2481032	30.4338984
21 C	35.0052812	34.0635398	29.9657312
22 H	35.7468369	34.4902733	30.6377901
23 Cl	32.3135084	30.8932190	32.3589339
24 Fe	34.1873932	32.2815691	32.3027698
25 O	33.7813717	28.9556406	34.4774156
26 O	34.7011813	32.7160356	35.0859669
27 C	35.0982179	33.7149761	34.4635184
28 O	34.9159611	33.8604939	33.1941535
29 C	35.8980943	34.8056547	35.1362591
30 C	37.3073159	34.9140026	34.5309024
31 C	38.1828437	35.7698501	35.4536010
32 O	38.9269002	35.1447007	36.2535613
33 O	38.0483117	37.0144616	35.3678238
34 H	35.3917852	35.7751429	35.0059903
35 H	35.9610561	34.5838141	36.2128565
36 H	37.7544043	33.9114455	34.4363184
37 H	37.2404464	35.3703176	33.5316516
38 N	32.8272837	26.1935780	31.5610736
39 C	33.9814206	26.8400836	32.2641923
40 H	33.9186165	27.9153039	32.0456556
41 C	33.8545552	26.6500526	33.7757276
42 H	33.4224170	25.6623905	33.9841310
43 H	34.8598912	26.6782824	34.2149572
44 C	33.0322479	27.7475047	34.4670434

45 H 32.0919429 27.9092904 33.9077629

46 H 33.3298913 29.6116455 33.9168581

47 C 32.6913439 27.3194359 35.8985222

48 H 32.1209169 26.3761947 35.8738657

49 H 33.6338885 27.0988696 36.4283829

50 C 31.9559546 28.3535359 36.7337117

51 H 31.9103359 28.0330638 37.7829282

52 H 32.4634964 29.3273437 36.6903639

53 N 30.5273230 28.5762445 36.3163432

54 H 30.0110427 28.9779483 37.1251982

55 H 30.0427399 27.6933620 36.0378502

56 H 30.4003143 29.2237420 35.5137559

57 C 35.3242801 26.3577554 31.6668772

58 O 36.3485703 26.8326679 32.2103802

59 O 35.2983640 25.5493019 30.7076326

60 H 31.9404555 26.3221687 32.0923591

61 H 32.9347621 25.1619321 31.4753429

62 H 32.6941375 26.6136731 30.6227080

63 H 36.2552390 28.6496524 27.8599570

64 H 36.3834017 34.3863665 27.0197961

Chimera14[1]-Iso1-TS

QM(B1)/MM Energy = -3361.045075973116 a.u.

QM(B2)/MM Energy = -3363.170907000000 a.u.

QM(B3)/MM Energy = -3362.648012601409 a.u.

1 C 36.4914951 28.3307934 28.7691975

2 H 35.8382969 27.5065771 29.0780819

3 H 37.5182555 27.9379951 28.7641441

4 C 36.3601655 29.4400325 29.7593631

5 N 37.3547225 30.3443627 30.0729306

6 H 38.2646282 30.3974572 29.5980767

7 C 36.8869873 31.1892030 31.0162426

8 H 37.4563183 32.0129571 31.4411324

9 N 35.6405400 30.8619622 31.3444021

10 C 35.2980868 29.7834128 30.5593104

11 H 34.2986969 29.3592886 30.5868408

12 C 35.6621544 34.9246533 27.7616137

13 H 35.0310757 35.5222144 27.0819552

14 H 36.2659665 35.6463596 28.3315903

15 C 34.8254945 34.1254269 28.7098526

16 N 33.6793599 33.4255169 28.3730032

17 H 33.2435063 33.3531434 27.4477826

18 C 33.2046496 32.8224844 29.4817201

19 H 32.3288180 32.1784222 29.5032659

20 N 33.9697453 33.0990086 30.5268676

21 C 34.9766996 33.9168987 30.0594077

22 H 35.7410463 34.2973223 30.7285887

23 Cl 32.2349093 30.7516754 31.7765117

24 Fe 34.1677398 31.9476271 32.4234154

25 O 33.8932764 31.6224263 34.1903465

26 O 34.9984927 33.9609924 35.0362885

27 C 35.4595828 34.2581736 33.9211602

28 O 35.3133002 33.5477076 32.8540265

29 C 36.3165627 35.4971155 33.7348842

30 C 37.7659255 35.2208657 34.1914990

31 C 38.0356382 35.6968240 35.6320527

32 O 38.5577766 34.8867211 36.4437203

33 O 37.7572354 36.8974497 35.8611341

34 H 36.2811211 35.7866210 32.6747696

35 H 35.9203366 36.3263926 34.3384343

36 H 38.0355509 34.1586834 34.0796316

37 H 38.4545824 35.8019918 33.5543811

38 N 32.7981100 26.2279815 31.7159853

39 C 33.9840747 26.9193882 32.3074985

40 H 33.8705029 27.9778950 32.0355216

41 C 33.9960631 26.8253373 33.8353949
42 H 33.9441308 25.7699498 34.1501915
43 H 35.0030688 27.1749914 34.1289240
44 C 32.9622282 27.6416158 34.5373941
45 H 32.7443608 28.6457054 34.1537711
46 H 34.2572612 32.3517527 34.7458880
47 C 32.5064605 27.2254066 35.8963605
48 H 31.8727227 26.3180011 35.8297292
49 H 33.3763951 26.8957294 36.4998199
50 C 31.7887259 28.2850620 36.7177095
51 H 31.6418389 27.9370982 37.7486155
52 H 32.3515530 29.2295103 36.7526425
53 N 30.4245797 28.6141305 36.1947555
54 H 29.8943359 29.1400651 36.9171595
55 H 29.8789317 27.7566663 35.9551199
56 H 30.4240046 29.1934616 35.3315634
57 C 35.3022885 26.4160357 31.6684361
58 O 36.3509315 26.8944799 32.1666356
59 O 35.2408838 25.5804111 30.7375188
60 H 31.9463212 26.3605716 32.3053473
61 H 32.9087093 25.1958130 31.6319287
62 H 32.5851976 26.6219407 30.7799855
63 H 36.2610399 28.6000285 27.7384356
64 H 36.3230727 34.3546666 27.1086441

Chimera14[1]-Iso1-IM

QM(B1)/MM Energy = -3361.048510389913 a.u.

QM(B2)/MM Energy = -3363.173037000000 a.u.

QM(B3)/MM Energy = -3362.649009854529 a.u.

1 C 36.4928307 28.3624770 28.8229143
2 H 35.8429209 27.5376365 29.1376020
3 H 37.5214068 27.9733354 28.8232028
4 C 36.3561696 29.4829775 29.8011936
5 N 37.3506508 30.3931098 30.1009807
6 H 38.2603797 30.4384893 29.6259996
7 C 36.8790316 31.2560375 31.0257688
8 H 37.4314269 32.0994336 31.4312044
9 N 35.6305369 30.9370556 31.3594387
10 C 35.2932348 29.8409592 30.5943342
11 H 34.2935451 29.4185151 30.6252311
12 C 35.6730501 34.9670109 27.6189426
13 H 35.0585631 35.5391399 26.9036598
14 H 36.2709812 35.7076355 28.1695568
15 C 34.8210592 34.2169422 28.5905248
16 N 33.6838659 33.4846979 28.2909881
17 H 33.2533454 33.3472586 27.3703137
18 C 33.2120847 32.9394670 29.4280255
19 H 32.3488859 32.2808368 29.4853685
20 N 33.9748969 33.2848175 30.4570462
21 C 34.9740958 34.0867726 29.9463598
22 H 35.7492381 34.4927668 30.5849958
23 Cl 32.3539773 30.7962466 31.7797856
24 Fe 34.1808903 32.2284039 32.3134530
25 O 33.6420310 32.5020541 34.0173348
26 O 35.2663850 34.5632729 34.4883297
27 C 35.9467136 34.4372179 33.4575203
28 O 35.7906169 33.4973503 32.5886276
29 C 37.0730159 35.4186201 33.1784590
30 C 38.2728128 35.1564850 34.0981081
31 C 38.0994607 35.7256571 35.5221776
32 O 38.3825404 34.9746356 36.4970002
33 O 37.7628897 36.9306592 35.5865971
34 H 37.3714037 35.3388737 32.1217017
35 H 36.7194852 36.4419180 33.3795822
36 H 38.5222576 34.0853068 34.1401841

37 H 39.1429658 35.6862058 33.6737549
38 N 32.8200963 26.2317622 31.7292229
39 C 33.9970655 26.9417885 32.3167367
40 H 33.8759763 27.9959806 32.0309435
41 C 34.0092049 26.8666967 33.8461121
42 H 34.0126500 25.8137992 34.1756591
43 H 34.9979125 27.2699928 34.1352913
44 C 32.9294688 27.6337135 34.5338130
45 H 32.5925081 28.5773399 34.0890228
46 H 34.1424140 33.2664670 34.4044742
47 C 32.4597252 27.1998390 35.8821591
48 H 31.8104939 26.3046081 35.7925735
49 H 33.3192700 26.8449375 36.4833030
50 C 31.7477887 28.2504850 36.7209319
51 H 31.5696328 27.8719097 37.7362033
52 H 32.3292694 29.1804927 36.8036809
53 N 30.4050574 28.6256699 36.1767965
54 H 29.8758331 29.1677769 36.8881913
55 H 29.8404767 27.7841838 35.9257148
56 H 30.4359825 29.2077901 35.3156812
57 C 35.3222132 26.4395899 31.6928555
58 O 36.3628706 26.9559701 32.1669015
59 O 35.2724520 25.5633938 30.7980658
60 H 31.9638746 26.3557861 32.3138757
61 H 32.9443726 25.2011581 31.6465532
62 H 32.6035109 26.6205590 30.7915284
63 H 36.2641341 28.6145423 27.7874299
64 H 36.3420948 34.3618028 27.0072486

Chimera14^{1}-Iso1-TS-Cl

QM(B1)/MM Energy = -3360.997270460245 a.u.

QM(B2)/MM Energy = -3363.110863000000 a.u.

QM(B3)/MM Energy = -3362.591364025647 a.u.

1 C 36.4991136 28.3541230 28.8158185
2 H 35.8418618 27.5369648 29.1356616
3 H 37.5247885 27.9577167 28.8229782
4 C 36.3670573 29.4713986 29.7947852
5 N 37.3664228 30.3710487 30.1126281
6 H 38.2755430 30.4311365 29.6376533
7 C 36.8969160 31.2010728 31.0736850
8 H 37.4521746 32.0220135 31.5165774
9 N 35.6612190 30.8636417 31.4082554
10 C 35.3134250 29.8058554 30.6117440
11 H 34.3248072 29.3628078 30.6520758
12 C 35.6065333 34.8487031 27.8232616
13 H 34.9692612 35.4529269 27.1546512
14 H 36.1987283 35.5677108 28.4094222
15 C 34.7807785 34.0080539 28.7550832
16 N 33.6201957 33.3328195 28.4026805
17 H 33.1862300 33.2945462 27.4771924
18 C 33.1376318 32.7095944 29.4978236
19 H 32.2344881 32.1056047 29.4778776
20 N 33.9165514 32.9320555 30.5461886
21 C 34.9323701 33.7513101 30.0976195
22 H 35.7203706 34.0933098 30.7549368
23 Cl 33.3048351 30.0811567 33.4694559
24 Fe 34.1823629 32.1055116 32.4817696
25 O 33.0287339 32.8252350 33.7090366
26 O 34.9511751 34.7511987 34.3441985
27 C 35.7950889 34.3496920 33.5380353
28 O 35.8085873 33.1800788 32.9718674
29 C 36.9671602 35.2430704 33.1364195
30 C 38.2061238 35.0489263 34.0274297
31 C 38.0704847 35.7015236 35.4219565
32 O 38.3133878 34.9874394 36.4381698

33 O 37.7847563 36.9202630 35.4259818
34 H 37.2529584 35.0595132 32.0826523
35 H 36.6675195 36.2938057 33.2410059
36 H 38.4474155 33.9787395 34.1245237
37 H 39.0726533 35.5517437 33.5591382
38 N 32.8811095 26.3358968 31.8411650
39 C 34.1028826 27.0159289 32.3793193
40 H 34.0128781 28.0617573 32.0638949
41 C 34.2397666 26.9998765 33.9137843
42 H 33.9849008 25.9861442 34.2754403
43 H 35.3161400 27.1345111 34.0507647
44 C 33.5349756 28.0115451 34.7973504
45 H 34.2195023 28.6834559 35.3462420
46 H 33.3571366 33.7228140 33.8764169
47 C 32.4949767 27.4064424 35.6975429
48 H 31.8098973 26.7273882 35.1657056
49 H 33.1014415 26.7289338 36.3354889
50 C 31.7227310 28.2929446 36.6645702
51 H 31.4825314 27.7347537 37.5800890
52 H 32.2749138 29.1984628 36.9588603
53 N 30.4101055 28.7329681 36.1087623
54 H 29.8783604 29.2947791 36.8040005
55 H 29.8236417 27.9104105 35.8470711
56 H 30.4865437 29.3041839 35.2458508
57 C 35.3803534 26.4458829 31.7049834
58 O 36.4529035 26.9119081 32.1432906
59 O 35.2669263 25.5895228 30.8031942
60 H 32.0298368 26.4877321 32.4224663
61 H 32.9685104 25.3013215 31.7498530
62 H 32.6615322 26.7195960 30.9024337
63 H 36.2741225 28.6068302 27.7796790
64 H 36.2837671 34.3201246 27.1524216

Chimera14[1]-Iso1-PC-Cl
QM(B1)/MM Energy = -3361.060635051925 a.u.
QM(B2)/MM Energy = -3363.195876000000 a.u.
QM(B3)/MM Energy = -3362.663606366627 a.u.

1 C 36.4686491 28.2991020 28.7764622
2 H 35.8285554 27.4589773 29.0685993
3 H 37.5026777 27.9248022 28.7834189
4 C 36.3010308 29.4001155 29.7711878
5 N 37.2629720 30.3441400 30.0616541
6 H 38.1708829 30.4180545 29.5893043
7 C 36.7582342 31.1928598 30.9893357
8 H 37.2867502 32.0484916 31.4019999
9 N 35.5273821 30.8341287 31.3303984
10 C 35.2314452 29.7244486 30.5764710
11 H 34.2591007 29.2445401 30.6284474
12 C 35.6924356 34.9388836 27.6550107
13 H 35.0789026 35.5313195 26.9556134
14 H 36.2966442 35.6634958 28.2202476
15 C 34.8372053 34.1681847 28.6079649
16 N 33.6764802 33.4886090 28.2862922
17 H 33.2437857 33.3938383 27.3624635
18 C 33.1935633 32.9179795 29.4143062
19 H 32.2945761 32.3060544 29.4528299
20 N 33.9693516 33.1964633 30.4447452
21 C 34.9912729 33.9760223 29.9606393
22 H 35.7785519 34.3435246 30.6118808
23 Cl 33.0293535 29.1851631 33.8891531
24 Fe 34.0859247 32.1438537 32.3201972
25 O 32.2484060 31.7074824 32.5432942
26 O 34.7404787 34.6128108 34.4347009
27 C 35.5996829 34.3266412 33.6003695
28 O 35.6504301 33.2058190 32.9527921

29 C 36.7314458 35.3016151 33.2724000
30 C 37.9934526 35.0227236 34.1050843
31 C 37.9596594 35.6715685 35.5037384
32 O 38.2320064 34.9499821 36.5051823
33 O 37.7167959 36.9004141 35.5278027
34 H 36.9715709 35.2191105 32.2003137
35 H 36.4054433 36.3303953 33.4828669
36 H 38.1883196 33.9420768 34.1836734
37 H 38.8554169 35.4817358 33.5899353
38 N 32.8507223 26.1510670 31.5257744
39 C 33.9837463 26.7487347 32.3001165
40 H 33.9145114 27.8283496 32.1265824
41 C 33.8006104 26.4655960 33.8035937
42 H 32.9444435 25.7879742 33.9116214
43 H 34.6712661 25.9226856 34.1944388
44 C 33.5635752 27.6643684 34.7611958
45 H 34.5163447 27.9752044 35.2120489
46 H 31.7619796 32.5461571 32.5223085
47 C 32.5635089 27.2881667 35.8690561
48 H 31.7856371 26.6265135 35.4555287
49 H 33.1159185 26.6670032 36.5915439
50 C 31.8828064 28.4138954 36.6491105
51 H 31.6177850 28.0686138 37.6555622
52 H 32.5121929 29.3080288 36.7559005
53 N 30.5917660 28.8465142 36.0178092
54 H 30.0515359 29.4721449 36.6477445
55 H 29.9843138 28.0251560 35.8009786
56 H 30.7116267 29.3549739 35.1167786
57 C 35.3462666 26.3180112 31.7210721
58 O 36.3412899 26.8065730 32.3071252
59 O 35.3598514 25.5371114 30.7401189
60 H 31.9414215 26.3703512 31.9842482
61 H 32.8905533 25.1114996 31.4829618
62 H 32.8113932 26.5423601 30.5652764
63 H 36.2531386 28.5712830 27.7432440
64 H 36.3548630 34.3446317 27.0256286

Chimera14[1]-Iso2-TS

QM(B1)/MM Energy = -3361.038199669724 a.u.

QM(B2)/MM Energy = -3363.166214000000 a.u.

QM(B3)/MM Energy = -3362.643451561289 a.u.

1 C 36.5196686 28.3665797 28.8463903
2 H 35.8450479 27.5691050 29.1810122
3 H 37.5340566 27.9426789 28.8435814
4 C 36.4366155 29.5146593 29.7987272
5 N 37.4615638 30.4062200 30.0327632
6 H 38.3657882 30.3988647 29.5472736
7 C 37.0365557 31.3193245 30.9355917
8 H 37.6401309 32.1441411 31.3068935
9 N 35.7976774 31.0507939 31.3155783
10 C 35.4116109 29.9337989 30.6160643
11 H 34.4243462 29.4956166 30.7317991
12 C 35.6995936 34.9381140 27.7104745
13 H 35.0526023 35.5164115 27.0301664
14 H 36.3032480 35.6755188 28.2594869
15 C 34.8890529 34.1469405 28.6864539
16 N 33.7058990 33.4877737 28.4095195
17 H 33.2316828 33.4249134 27.5023620
18 C 33.2736097 32.8954916 29.5459145
19 H 32.3593147 32.3077946 29.6023265
20 N 34.1092174 33.1356411 30.5424028
21 C 35.1135368 33.9172572 30.0216234
22 H 35.9411575 34.2597788 30.6338821
23 Cl 34.0895176 30.3560644 33.8251296
24 Fe 34.3801634 32.2758620 32.5552201

25 O 33.0024232 33.2194956 33.2922630
26 O 34.9373129 35.0561064 34.1991981
27 C 35.8437328 34.6487059 33.4721574
28 O 35.8938508 33.4531619 32.9515954
29 C 37.0420582 35.5239243 33.1410780
30 C 38.2311341 35.2044291 34.0574182
31 C 38.0741195 35.8157037 35.4654646
32 O 38.2955165 35.0765741 36.4650031
33 O 37.7860796 37.0339435 35.4978715
34 H 37.3237914 35.3619698 32.0877011
35 H 36.7687922 36.5776594 33.2891994
36 H 38.4083885 34.1203002 34.1205377
37 H 39.1310550 35.6729160 33.6235316
38 N 32.8474196 26.1855731 31.6789075
39 C 34.0049769 26.8958283 32.3089703
40 H 33.8758066 27.9585320 32.0607708
41 C 33.9927800 26.7825186 33.8333186
42 H 34.1316751 25.7311836 34.1442513
43 H 34.9041326 27.3162293 34.1608295
44 C 32.7948737 27.3810042 34.4891444
45 H 32.1952548 28.1069965 33.9337679
46 H 33.3559921 34.0342097 33.7095841
47 C 32.4573271 27.0646774 35.9061126
48 H 31.7653841 26.1989972 35.9542053
49 H 33.3608132 26.7317247 36.4461874
50 C 31.8561285 28.2058009 36.7185938
51 H 31.7433763 27.9045037 37.7688414
52 H 32.4778317 29.1117313 36.6793021
53 N 30.4817946 28.5958005 36.2522239
54 H 29.9808977 29.0703344 37.0305980
55 H 29.9180009 27.7652776 35.9642214
56 H 30.4714936 29.2386880 35.4374687
57 C 35.3395028 26.4256112 31.6875196
58 O 36.3687859 26.9670214 32.1519706
59 O 35.3069689 25.5386433 30.7980654
60 H 31.9671432 26.3237002 32.2226433
61 H 32.9695455 25.1539458 31.6151578
62 H 32.6874334 26.5676412 30.7299291
63 H 36.2948670 28.6111617 27.8082616
64 H 36.3581523 34.3604393 27.0618917

Chimera14[1]-Iso2-IM

QM(B1)/MM Energy = -3361.043396483082 a.u.

QM(B2)/MM Energy = -3363.173589000000 a.u.

QM(B3)/MM Energy = -3362.649631948544 a.u.

1 C 36.5002529 28.2898657 28.7416626
2 H 35.8601197 27.4550757 29.0484368
3 H 37.5334699 27.9152404 28.7450119
4 C 36.3414308 29.4048463 29.7206354
5 N 37.3236665 30.3192222 30.0368470
6 H 38.2449490 30.3648163 29.5845064
7 C 36.8218382 31.1923813 30.9386177
8 H 37.3723496 32.0284881 31.3622540
9 N 35.5681869 30.8735811 31.2330498
10 C 35.2562185 29.7691490 30.4822104
11 H 34.2749450 29.3083289 30.5192494
12 C 35.6753842 34.9110046 27.6910000
13 H 35.0405286 35.4944015 27.0033410
14 H 36.2735493 35.6444366 28.2513457
15 C 34.8497953 34.1166006 28.6512788
16 N 33.6983819 33.4141498 28.3421915
17 H 33.2479523 33.3329682 27.4244517
18 C 33.2443890 32.8275188 29.4717921
19 H 32.3580050 32.1976491 29.5163377
20 N 34.0311138 33.1150686 30.4940022

21 C 35.0286583 33.9179614 29.9993318
22 H 35.8151537 34.3018820 30.6400529
23 Cl 34.3578266 30.4438692 34.1480327
24 Fe 34.1888438 31.9811982 32.4506998
25 O 32.3164090 31.9028837 32.3691483
26 O 34.7441744 34.7659572 34.4667631
27 C 35.5301080 34.4567706 33.5852086
28 O 35.4601036 33.3432229 32.8994706
29 C 36.7258236 35.3301317 33.2216119
30 C 37.9557109 34.9900648 34.0804523
31 C 37.9702440 35.7044864 35.4492874
32 O 38.2677223 35.0209217 36.4692784
33 O 37.7453481 36.9358203 35.4249648
34 H 36.9649428 35.1957371 32.1553058
35 H 36.4725841 36.3854328 33.3997854
36 H 38.0682104 33.9029377 34.2117718
37 H 38.8533734 35.3547201 33.5511932
38 N 32.8533247 26.1949636 31.6838590
39 C 34.0144657 26.9033758 32.3078184
40 H 33.8918138 27.9639576 32.0494282
41 C 33.9950200 26.8098491 33.8336866
42 H 34.1117967 25.7619347 34.1615183
43 H 34.9128286 27.3341207 34.1595008
44 C 32.8025730 27.4456493 34.4618948
45 H 32.2759252 28.2221830 33.8997294
46 H 31.9284947 32.7226298 32.0267669
47 C 32.4256795 27.1728843 35.8786564
48 H 31.7121780 26.3247405 35.9365314
49 H 33.3065459 26.8336337 36.4520276
50 C 31.8354524 28.3608096 36.6314161
51 H 31.6504315 28.0995146 37.6809846
52 H 32.4997729 29.2362327 36.5974693
53 N 30.5126081 28.7951277 36.0664133
54 H 29.9906889 29.3675900 36.7587496
55 H 29.9156499 27.9748493 35.8189048
56 H 30.6007193 29.3672690 35.2027153
57 C 35.3495932 26.4284667 31.6902865
58 O 36.3788637 26.9651323 32.1618769
59 O 35.3160466 25.5478494 30.7953241
60 H 31.9731127 26.3500759 32.2239103
61 H 32.9650209 25.1610701 31.6350639
62 H 32.6895224 26.5626620 30.7287219
63 H 36.2724489 28.5587398 27.7102174
64 H 36.3410192 34.3331273 27.0498644

Chimera14[1]-Iso2-TS-Cl

QM(B1)/MM Energy = -3361.036332532953 a.u.

QM(B2)/MM Energy = -3363.165485000000 a.u.

QM(B3)/MM Energy = -3362.648276882485 a.u.

1 C 36.5064753 28.2728318 28.7115246
2 H 35.8650488 27.4354165 29.0094130
3 H 37.5387871 27.8951611 28.7088112
4 C 36.3510407 29.3740783 29.7068422
5 N 37.3262748 30.2932456 30.0294170
6 H 38.2430142 30.3566778 29.5714933
7 C 36.8217221 31.1396878 30.9590045
8 H 37.3698639 31.9699417 31.3974128
9 N 35.5785686 30.7996389 31.2656412
10 C 35.2732142 29.7105205 30.4913540
11 H 34.2959563 29.2413743 30.5225074
12 C 35.6529006 34.8674237 27.7485665
13 H 35.0151676 35.4669867 27.0774661
14 H 36.2486175 35.5886572 28.3278025
15 C 34.8257158 34.0461446 28.6868292
16 N 33.6545275 33.3847949 28.3613297

17 H 33.2053866 33.3349137 27.4410023
18 C 33.1910243 32.7724380 29.4751884
19 H 32.2851684 32.1700800 29.5049815
20 N 33.9903579 33.0014483 30.5007615
21 C 35.0055034 33.7945334 30.0268013
22 H 35.8055068 34.1355910 30.6756572
23 Cl 34.1083745 29.8888141 33.8633237
24 Fe 34.0896908 31.8719321 32.4668671
25 O 32.2086746 31.8956869 32.4842788
26 O 34.6436862 34.5758600 34.5324831
27 C 35.4577385 34.2623297 33.6739885
28 O 35.4525198 33.1201197 33.0411917
29 C 36.6116933 35.1846071 33.2884526
30 C 37.8833770 34.8889632 34.1033370
31 C 37.9390826 35.6294281 35.4569605
32 O 38.2540368 34.9625761 36.4837687
33 O 37.7222122 36.8619053 35.4164788
34 H 36.8222805 35.0646926 32.2147592
35 H 36.3226258 36.2288616 33.4752162
36 H 38.0226455 33.8070037 34.2492789
37 H 38.7522138 35.2601038 33.5321594
38 N 32.8478149 26.1446207 31.5735002
39 C 33.9931424 26.8469142 32.2342023
40 H 33.8981598 27.9060678 31.9650790
41 C 33.9178480 26.7603565 33.7566489
42 H 33.7238261 25.7166717 34.0645123
43 H 34.9203532 27.0040927 34.1366838
44 C 32.9191696 27.6712633 34.3951247
45 H 32.0922457 28.0383987 33.7811772
46 H 31.8609716 32.7514262 32.1924551
47 C 32.6954310 27.5540015 35.8654645
48 H 32.1433720 26.6057961 36.0516401
49 H 33.6585101 27.4263254 36.3895330
50 C 31.9557466 28.7003601 36.5441829
51 H 31.8431725 28.5022736 37.6172502
52 H 32.4925538 29.6500680 36.4125929
53 N 30.5702994 28.9036104 36.0040739
54 H 30.0004598 29.4652137 36.6671590
55 H 30.0593464 28.0070643 35.8378509
56 H 30.5667946 29.4202277 35.1016225
57 C 35.3431063 26.3588414 31.6569151
58 O 36.3633437 26.8509976 32.1925584
59 O 35.3232715 25.5227677 30.7209991
60 H 31.9541916 26.3022209 32.0859177
61 H 32.9569882 25.1099369 31.5364692
62 H 32.7170949 26.5120827 30.6126625
63 H 36.2781918 28.5505587 27.6825340
64 H 36.3214309 34.3137861 27.0893095

Chimera14^{1}-Iso2-PC-Cl
QM(B1)/MM Energy = -3361.077333970246 a.u.
QM(B2)/MM Energy = -3363.213087000000 a.u.
QM(B3)/MM Energy = -3362.680277587607 a.u.

1 C 36.4597768 28.2891082 28.7297697
2 H 35.8135065 27.4529047 29.0193288
3 H 37.4902303 27.9056111 28.7305778
4 C 36.3069968 29.3841327 29.7331096
5 N 37.2886358 30.2977271 30.0526024
6 H 38.2010479 30.3613317 29.5871601
7 C 36.7937870 31.1411847 30.9914408
8 H 37.3380761 31.9764045 31.4256654
9 N 35.5513080 30.8063730 31.3103117
10 C 35.2359943 29.7224521 30.5295294
11 H 34.2490519 29.2715615 30.5555614
12 C 35.6761090 34.9219034 27.6741570

13 H	35.0567130	35.5153228	26.9805621
14 H	36.2775191	35.6466907	28.2424576
15 C	34.8274936	34.1395712	28.6245564
16 N	33.6709676	33.4534903	28.3003826
17 H	33.2380173	33.3622394	27.3761518
18 C	33.1928776	32.8753801	29.4271728
19 H	32.2999696	32.2541632	29.4627044
20 N	33.9659955	33.1556396	30.4592346
21 C	34.9826752	33.9433020	29.9769193
22 H	35.7681792	34.3119554	30.6296711
23 Cl	34.0398475	29.2288749	34.6052876
24 Fe	34.1133123	32.0769898	32.3343871
25 O	32.2877867	31.5751111	32.4989219
26 O	34.7541881	34.5564248	34.4590624
27 C	35.6062986	34.2817555	33.6141799
28 O	35.6576541	33.1663896	32.9571105
29 C	36.7285879	35.2664034	33.2814177
30 C	37.9972192	34.9984530	34.1078270
31 C	37.9676941	35.6522530	35.5040750
32 O	38.2505215	34.9353909	36.5061547
33 O	37.7171022	36.8797284	35.5261450
34 H	36.9644854	35.1863066	32.2083399
35 H	36.3945049	36.2922974	33.4937113
36 H	38.1995456	33.9194302	34.1894188
37 H	38.8535108	35.4606812	33.5859121
38 N	32.8255073	26.1997163	31.5139398
39 C	33.9635087	26.8349450	32.2510405
40 H	33.9131854	27.9109058	32.0377383
41 C	33.8033310	26.6196122	33.7590731
42 H	33.2885028	25.6634147	33.9269581
43 H	34.7959889	26.5447815	34.2202112
44 C	33.0091855	27.7075406	34.4810059
45 H	32.1476400	28.0298057	33.8795182
46 H	31.7697004	32.3729889	32.3126850
47 C	32.5714142	27.2588708	35.8725321
48 H	31.9289148	26.3707935	35.7409571
49 H	33.4545522	26.9158716	36.4360679
50 C	31.8589610	28.2842006	36.7405273
51 H	31.6485390	27.8523418	37.7280575
52 H	32.4662771	29.1876427	36.8868833
53 N	30.5387949	28.7259284	36.1843933
54 H	30.0129962	29.2474006	36.9152564
55 H	29.9510945	27.9138207	35.8919898
56 H	30.6133754	29.3453219	35.3513541
57 C	35.3220497	26.3524095	31.6819615
58 O	36.3323562	26.8046116	32.2689753
59 O	35.3104106	25.5645181	30.7070241
60 H	31.9236210	26.3538093	32.0113315
61 H	32.9207462	25.1646869	31.4505814
62 H	32.7311168	26.6033535	30.5619622
63 H	36.2419467	28.5700186	27.6993783
64 H	36.3413292	34.3383595	27.0377408

Chimera14[1]-Iso2-TS-OH

QM(B1)/MM Energy = -3361.000652247530 a.u.

QM(B2)/MM Energy = -3363.125769000000 a.u.

QM(B3)/MM Energy = -3362.608338957824 a.u.

1 C	36.5039111	28.2605662	28.6717498
2 H	35.8326031	27.4523323	28.9877583
3 H	37.5148052	27.8303570	28.6428766
4 C	36.4396461	29.3638704	29.6770602
5 N	37.4949973	30.1720500	30.0424495
6 H	38.4058319	30.1938018	29.5681606
7 C	37.0822192	30.9936018	31.0363785
8 H	37.7219148	31.7333909	31.5138830

9 N 35.8165321 30.7480092 31.3411488

10 C 35.4057201 29.7538980 30.4905240

11 H 34.3827088 29.4001061 30.4852971

12 C 35.4875708 34.6709744 27.9355251

13 H 34.8411227 35.3198484 27.3196888

14 H 36.0487279 35.3462018 28.5997796

15 C 34.6598966 33.7396862 28.7720110

16 N 33.5040651 33.0982014 28.3621265

17 H 33.0725099 33.1295210 27.4328810

18 C 33.0227405 32.3914944 29.4138412

19 H 32.1079584 31.8046436 29.3530981

20 N 33.7863064 32.5361215 30.4810195

21 C 34.8004700 33.3826813 30.0923413

22 H 35.5577102 33.7074966 30.7966898

23 Cl 35.0663956 30.8602719 34.7211448

24 Fe 34.2932878 31.5739224 32.6377113

25 O 32.8679752 30.2262199 32.7452351

26 O 34.8031635 34.1482514 35.0708272

27 C 35.1444566 34.1480021 33.8989041

28 O 34.5601828 33.4535367 32.9603404

29 C 36.3494381 34.9736041 33.4449188

30 C 37.6211926 34.5637171 34.2072619

31 C 37.8896992 35.3566488 35.5068953

32 O 38.3687862 34.7101350 36.4833834

33 O 37.6957734 36.5929584 35.4640333

34 H 36.4849176 34.8725008 32.3573785

35 H 36.1657051 36.0386550 33.6636567

36 H 37.6305342 33.4842396 34.4271978

37 H 38.4986233 34.7719497 33.5675354

38 N 32.8615880 26.0805410 31.4772721

39 C 33.9806215 26.9597578 31.9513981

40 H 33.8654272 27.8815476 31.3736938

41 C 33.9146044 27.3397703 33.4226102

42 H 33.9464729 26.4275034 34.0554874

43 H 34.8582415 27.8678530 33.6350387

44 C 32.7879371 28.1962840 33.9071691

45 H 31.8183417 28.1671230 33.4095330

46 H 32.4425661 29.9184119 31.9303658

47 C 32.8209435 28.4079603 35.3893199

48 H 32.6087309 27.4174603 35.8527832

49 H 33.8492963 28.6642637 35.6843522

50 C 31.9161065 29.4486619 36.0213999

51 H 32.2074134 29.6020063 37.0679545

52 H 32.0136227 30.4084819 35.5004271

53 N 30.4584319 29.0666296 36.0336271

54 H 30.0581179 29.2288875 36.9816047

55 H 30.2441390 28.0804749 35.7645409

56 H 29.9199710 29.6591918 35.3775914

57 C 35.3443413 26.3494559 31.5350867

58 O 36.3577954 26.8158806 32.1014551

59 O 35.3328364 25.4508515 30.6584342

60 H 31.9507149 26.3155908 31.9247783

61 H 32.9867318 25.0612090 31.6530852

62 H 32.7549912 26.2105608 30.4571388

63 H 36.2655570 28.5390072 27.6452387

64 H 36.1877533 34.2261959 27.2284298

Chimera14[1]-Iso2-PC-OH

QM(B1)/MM Energy = -3361.108747108261 a.u.

QM(B2)/MM Energy = -3363.232413000000 a.u.

QM(B3)/MM Energy = -3362.697165456666 a.u.

1 C 36.4824243 28.3614480 28.9177241

2 H 35.8185700 27.5511666 29.2411889

3 H 37.5033247 27.9526152 28.9195734

4 C 36.3769672 29.4993484 29.8808142

5 N 37.3939883 30.4011981 30.1110221

6 H 38.2889692 30.4145972 29.6085778

7 C 36.9727539 31.2961507 31.0326562

8 H 37.5597573 32.1351158 31.3988829

9 N 35.7400408 31.0079666 31.4300808

10 C 35.3540587 29.8965174 30.7161286

11 H 34.3611373 29.4665029 30.8258560

12 C 35.7494950 34.9998607 27.6116648

13 H 35.1111034 35.5523790 26.9025568

14 H 36.3647810 35.7529408 28.1241952

15 C 34.9354503 34.2687549 28.6266723

16 N 33.7582112 33.5861531 28.3861771

17 H 33.2777288 33.4792941 27.4869227

18 C 33.3373515 33.0430186 29.5514828

19 H 32.4346103 32.4440914 29.6549665

20 N 34.1740430 33.3413024 30.5291133

21 C 35.1685818 34.1074685 29.9703847

22 H 35.9965309 34.4838897 30.5641623

23 Cl 32.3640024 31.7964512 33.0311406

24 Fe 34.4679511 32.4434529 32.4284731

25 O 32.2180276 28.3425205 33.4814696

26 O 35.0214679 34.9390549 34.2773216

27 C 35.9432378 34.6421745 33.5202154

28 O 36.0508521 33.4925481 32.9199765

29 C 37.0898132 35.6054584 33.2437344

30 C 38.2836233 35.3062115 34.1604948

31 C 38.0530796 35.8084038 35.5998092

32 O 38.2312352 35.0013977 36.5529221

33 O 37.7496751 37.0200160 35.7071809

34 H 37.3937504 35.5127561 32.1882410

35 H 36.7585909 36.6349117 33.4387504

36 H 38.5259994 34.2327708 34.1595312

37 H 39.1606006 35.8569174 33.7783453

38 N 32.8811885 26.0710776 31.7129229

39 C 34.0266600 26.8100185 32.3325907

40 H 33.8805259 27.8450163 32.0053046

41 C 34.0088142 26.8291032 33.8665413

42 H 34.1939213 25.8319659 34.2901498

43 H 34.8753546 27.4489007 34.1376462

44 C 32.7169508 27.4402883 34.4585762

45 H 31.9952318 26.6253598 34.6360354

46 H 31.2541300 28.3418859 33.4850017

47 C 32.9615819 28.1658545 35.8069045

48 H 32.9990874 27.4314932 36.6306862

49 H 33.9483773 28.6585847 35.7834479

50 C 31.9694779 29.2889666 36.0980446

51 H 32.2869644 29.8730527 36.9727021

52 H 31.9168502 29.9628813 35.2338462

53 N 30.5691084 28.8354381 36.4019400

54 H 30.4894079 28.5608630 37.4027500

55 H 30.2038865 28.0443695 35.8267574

56 H 29.9026436 29.6240627 36.2770183

57 C 35.3713185 26.3383615 31.7436616

58 O 36.3881931 26.9118459 32.2006188

59 O 35.3609296 25.4387342 30.8678492

60 H 31.9775132 26.3074517 32.1719460

61 H 32.9536828 25.0333529 31.7323250

62 H 32.7839745 26.3806820 30.7295413

63 H 36.2635136 28.6068466 27.8785298

64 H 36.3964983 34.3723791 26.9987042

Chimera14^{2}-RC

QM(B1)/MM Energy = -3360.847125364460 a.u.

QM(B2)/MM Energy = -3362.975036000000 a.u.

QM(B3)/MM Energy = -3362.451741747370 a.u.

1 C	30.8467456	26.9685262	28.9517168
2 H	30.1882335	27.5519445	28.2967397
3 H	30.3618642	25.9922683	29.0965247
4 C	30.9942249	27.6896628	30.2506197
5 N	31.2034389	27.0793206	31.4680612
6 H	31.3137632	26.0675947	31.6035541
7 C	31.3072017	28.0346801	32.4169812
8 H	31.4719714	27.8312262	33.4727685
9 N	31.1560345	29.2332278	31.8683187
10 C	30.9656328	29.0364441	30.5221790
11 H	30.8423394	29.8684731	29.8340861
12 C	36.3819747	28.5665407	32.6476925
13 H	37.3884022	28.9548604	32.4205439
14 H	36.3497838	28.4135246	33.7367609
15 C	35.3198049	29.5395262	32.2466076
16 N	35.3263945	30.3076810	31.0935483
17 H	36.0738987	30.3654329	30.3918802
18 C	34.1840421	31.0230005	31.0485064
19 H	33.9150767	31.7072963	30.2469192
20 N	33.4429044	30.7687850	32.1160787
21 C	34.1406506	29.8551965	32.8729724
22 H	33.7553063	29.4957980	33.8215912
23 Cl	31.4034088	32.5737716	30.7180925
24 Fe	31.4283859	31.2039571	32.5796456
25 O	29.8687818	31.4395266	32.9308958
26 O	30.8461341	32.3775524	35.8294358
27 C	31.6577948	31.5083065	35.5539608
28 O	32.0890017	31.2287995	34.3456284
29 C	32.3089680	30.6079596	36.5938578
30 C	31.6589234	29.2046541	36.6085648
31 C	30.5920389	29.0713038	37.7292906
32 O	29.4677706	28.6137204	37.4059082
33 O	30.9846110	29.4137946	38.8640745
34 H	33.3815518	30.5464367	36.3473241
35 H	32.1909219	31.0473256	37.5941793
36 H	31.2173900	28.9530611	35.6305065
37 H	32.4340357	28.4488677	36.8270049
38 N	27.9962422	30.9808364	27.4027296
39 C	27.9560567	30.6578840	28.8690689
40 H	28.9649091	30.8829269	29.2444761
41 C	26.9551982	31.4949406	29.6756059
42 H	25.9205139	31.2272288	29.4060850
43 H	27.0981627	31.1799586	30.7213822
44 C	27.1751041	33.0035523	29.5467383
45 H	26.9279640	33.3255005	28.5208040
46 H	28.2486156	33.2209421	29.6943697
47 C	26.3670323	33.8721285	30.5254760
48 H	25.3112537	33.5609225	30.5491723
49 H	26.7616507	33.7452678	31.5465230
50 C	26.4960335	35.3224134	30.0731664
51 H	27.5341702	35.5251441	29.7774338
52 H	25.8666624	35.5293347	29.1967133
53 N	26.1849152	36.3689680	31.0948986
54 H	26.4535321	37.2826293	30.6802164
55 H	26.7664587	36.2565969	31.9524340
56 H	25.1915739	36.4482754	31.3981410
57 C	27.7034826	29.1366113	29.0108439
58 O	27.7571723	28.6538344	30.1587995
59 O	27.4280020	28.5123819	27.9477910
60 H	27.9066301	31.9847171	27.1471004
61 H	27.2803663	30.4536220	26.8596540
62 H	28.9274379	30.6924709	27.0444747
63 H	31.7895445	26.7736812	28.4406074
64 H	36.3267166	27.5980501	32.1506616

Chimera14^{2}-TS1

QM(B1)/MM Energy = -3360.802774805590 a.u.

QM(B2)/MM Energy = -3362.930982000000 a.u.

QM(B3)/MM Energy = -3362.413447328902 a.u.

1 C	30.8600412	26.8876475	28.7683571
2 H	30.2226595	27.4541104	28.0791386
3 H	30.3819401	25.9074367	28.9056324
4 C	30.9300324	27.6347151	30.0568940
5 N	31.0979850	27.0661026	31.3009900
6 H	31.2440792	26.0643017	31.4732043
7 C	31.0994759	28.0490549	32.2260201
8 H	31.2140862	27.8844012	33.2951166
9 N	30.9194304	29.2242301	31.6357770
10 C	30.8287567	28.9833441	30.2881135
11 H	30.7272775	29.7878089	29.5661391
12 C	36.1681343	28.6480306	32.6841731
13 H	37.1669800	29.0732660	32.4901973
14 H	36.1082102	28.4925461	33.7723402
15 C	35.0922389	29.5933406	32.2455887
16 N	35.1663033	30.4060798	31.1267206
17 H	35.9532715	30.4800922	30.4708598
18 C	34.0176459	31.1150162	31.0408885
19 H	33.7973116	31.8277928	30.2491238
20 N	33.2047055	30.8167638	32.0427418
21 C	33.8649125	29.8760166	32.8002808
22 H	33.4198380	29.4536381	33.6962666
23 Cl	31.3096599	32.6997205	30.4923751
24 Fe	30.9429133	31.2222246	32.2994646
25 O	29.1832900	31.4909570	32.2764626
26 O	31.0618894	32.5194294	35.8335190
27 C	31.4640297	31.4571414	35.3826800
28 O	31.2672837	31.0503093	34.1516489
29 C	32.2851114	30.4791503	36.2191436
30 C	31.6488861	29.0852011	36.3701134
31 C	30.6739469	29.0152448	37.5794050
32 O	29.4970669	28.6378003	37.3429551
33 O	31.1747084	29.3249251	38.6810035
34 H	33.2719487	30.4117283	35.7285982
35 H	32.4278669	30.9064699	37.2213278
36 H	31.1303476	28.7844194	35.4462533
37 H	32.4472283	28.3481657	36.5685014
38 N	28.0598673	30.9586368	27.3694494
39 C	27.9798951	30.6382303	28.8320088
40 H	28.9896775	30.7984263	29.2304824
41 C	26.9981614	31.5531602	29.5882697
42 H	26.3852088	32.1034704	28.8552032
43 H	26.2885265	30.9432070	30.1661463
44 C	27.6404549	32.5808787	30.5086434
45 H	28.5028406	33.0826969	30.0397788
46 H	28.2671580	31.9822438	31.3978433
47 C	26.6553031	33.5884815	31.1116134
48 H	25.6531535	33.1377028	31.2034188
49 H	26.9630994	33.8440472	32.1323241
50 C	26.5924785	34.8845258	30.2834058
51 H	27.5692658	35.0812796	29.8202020
52 H	25.8449966	34.8306527	29.4800080
53 N	26.3019692	36.1118813	31.0906057
54 H	26.5063419	36.9450811	30.4969702
55 H	26.9410637	36.1871868	31.9109933
56 H	25.3284302	36.2273256	31.4447034
57 C	27.6522968	29.1286947	28.9720652
58 O	27.6412335	28.6598303	30.1273361
59 O	27.3982939	28.5078281	27.9050925
60 H	27.9796126	31.9637681	27.1144139
61 H	27.3410111	30.4399910	26.8203095
62 H	28.9911825	30.6571764	27.0182455

63 H 31.8290504 26.7033903 28.3045279

64 H 36.1740742 27.6766624 32.1897284

Chimera14^2-IM1

QM(B1)/MM Energy = -3360.839961165994 a.u.

QM(B2)/MM Energy = -3362.968742000000 a.u.

QM(B3)/MM Energy = -3362.445329978713 a.u.

1 C 30.8390259 26.9231600 28.8762205

2 H 30.1856598 27.4972621 28.2083913

3 H 30.3594656 25.9445757 29.0213993

4 C 30.9621400 27.6567621 30.1697616

5 N 31.1625000 27.0639127 31.3975428

6 H 31.2845071 26.0554050 31.5472269

7 C 31.2367646 28.0298006 32.3364724

8 H 31.3888519 27.8411281 33.3969864

9 N 31.0730609 29.2207366 31.7729393

10 C 30.9113336 29.0046833 30.4252461

11 H 30.7984960 29.8250096 29.7214472

12 C 36.2951446 28.5988467 32.6842843

13 H 37.3040302 28.9936925 32.4790011

14 H 36.2462806 28.4365466 33.7717042

15 C 35.2398503 29.5760715 32.2711599

16 N 35.2878940 30.3629221 31.1322949

17 H 36.0513201 30.4154593 30.4475123

18 C 34.1539573 31.0929128 31.0694876

19 H 33.9166941 31.7887038 30.2675412

20 N 33.3759906 30.8335075 32.1088907

21 C 34.0435897 29.8978079 32.8666569

22 H 33.6239125 29.5123615 33.7909935

23 Cl 31.3795270 32.6359729 30.5382916

24 Fe 31.2021247 31.2185024 32.4505992

25 O 29.3622216 31.5813969 32.4749254

26 O 31.0200064 32.4319411 36.0431898

27 C 31.5942314 31.4649784 35.5655690

28 O 31.5816419 31.1554655 34.2915780

29 C 32.4056163 30.4870607 36.4040825

30 C 31.7467538 29.0964446 36.4934234

31 C 30.7059702 29.0271662 37.6461607

32 O 29.5454238 28.6441492 37.3516168

33 O 31.1504936 29.3458061 38.7688381

34 H 33.4078696 30.4225948 35.9465192

35 H 32.5039561 30.8819959 37.4243631

36 H 31.2763735 28.8156533 35.5371528

37 H 32.5246106 28.3473361 36.7246555

38 N 27.9637433 30.9981021 27.4223844

39 C 27.9460174 30.6146111 28.8706984

40 H 28.9624687 30.8101195 29.2397406

41 C 26.9709612 31.4203428 29.7353767

42 H 25.9258066 31.2454316 29.4222435

43 H 27.0507138 30.9564985 30.7398406

44 C 27.2732301 32.8793416 29.8198656

45 H 28.2781041 33.2205940 29.5378659

46 H 29.1141556 31.9935645 31.6326680

47 C 26.4229979 33.8289829 30.6061172

48 H 25.3690622 33.5063145 30.6093472

49 H 26.7448986 33.8117747 31.6692338

50 C 26.5686914 35.2484348 30.0585867

51 H 27.6061818 35.4197607 29.7424319

52 H 25.9279926 35.4146973 29.1830845

53 N 26.2834995 36.3395639 31.0404723

54 H 26.5394519 37.2362060 30.5794025

55 H 26.8902582 36.2650075 31.8844788

56 H 25.2984052 36.4328457 31.3672968

57 C 27.6795785 29.0929189 28.9710671

58 O 27.7253733 28.5892528 30.1112473

59 O 27.4034443 28.4954122 27.8959737

60 H 27.8245221 32.0073972 27.2082927

61 H 27.2687016 30.4602882 26.8611109

62 H 28.9056421 30.7698978 27.0472077

63 H 31.7922329 26.7357225 28.3819223

64 H 36.2595769 27.6333021 32.1797789

Chimera14^{2}-TS-Cl

QM(B1)/MM Energy = -3360.811677956483 a.u.

QM(B2)/MM Energy = -3362.940625000000 a.u.

QM(B3)/MM Energy = -3362.425134054495 a.u.

1 C 30.8432411 26.9618754 28.8645302

2 H 30.2014245 27.5428919 28.1899840

3 H 30.3562866 25.9842687 28.9912693

4 C 30.9326071 27.6781760 30.1721962

5 N 31.0418696 27.0630795 31.4006373

6 H 31.1700017 26.0541577 31.5406429

7 C 31.0327405 28.0155637 32.3595284

8 H 31.1056202 27.8057966 33.4251074

9 N 30.9048358 29.2171880 31.8117718

10 C 30.8600149 29.0204624 30.4526791

11 H 30.7945674 29.8530208 29.7580577

12 C 36.0274899 28.8396852 32.5690195

13 H 37.0250919 29.2763933 32.3908773

14 H 35.9285841 28.7447868 33.6614908

15 C 34.9518875 29.7552685 32.0607801

16 N 34.9997015 30.5009926 30.8921834

17 H 35.7806956 30.5433016 30.2261734

18 C 33.8572824 31.2280480 30.8105502

19 H 33.6349294 31.8998318 29.9827803

20 N 33.0779744 31.0071639 31.8573597

21 C 33.7556624 30.0994192 32.6423145

22 H 33.3476736 29.7568688 33.5877764

23 Cl 30.1580001 32.6699631 30.5311793

24 Fe 30.8937305 31.2521624 32.5390884

25 O 29.0699357 31.4400554 32.9938146

26 O 30.8953463 32.4162000 36.1800899

27 C 31.5310894 31.5610675 35.5721213

28 O 31.6615608 31.5114975 34.2766496

29 C 32.2596362 30.4390481 36.3073764

30 C 31.4519024 29.1249757 36.3087125

31 C 30.5127013 28.9862717 37.5422031

32 O 29.3461163 28.5721286 37.3183246

33 O 31.0326193 29.2539122 38.6452058

34 H 33.2407933 30.3010596 35.8232543

35 H 32.4140105 30.7260693 37.3567781

36 H 30.8652422 29.0124164 35.3823165

37 H 32.1536447 28.2718466 36.3616882

38 N 28.0374614 30.8643280 27.2404914

39 C 28.1082545 30.5344132 28.7002726

40 H 29.1673050 30.6309546 28.9657899

41 C 27.3320970 31.4819401 29.6042560

42 H 26.2557922 31.4842186 29.3385122

43 H 27.3664642 31.0221232 30.6073099

44 C 27.8062687 32.8979058 29.6544395

45 H 28.2452358 33.3288866 28.7507064

46 H 28.5782912 31.5981596 32.1784964

47 C 27.1131518 33.8360768 30.6034700

48 H 26.1074292 33.4440697 30.8422217

49 H 27.6654311 33.8457919 31.5587251

50 C 27.0181545 35.2457441 30.0297366

51 H 27.9941588 35.5465819 29.6250068

52 H 26.2867759 35.3008771 29.2109205

53 N 26.6601752 36.3049465 31.0227470

54 H 26.8017074 37.2193143 30.5465512

55 H 27.3053223 36.3013344 31.8407556
56 H 25.6802116 36.3125222 31.3789476
57 C 27.6978485 29.0541233 28.8935736
58 O 27.6815795 28.6176761 30.0629456
59 O 27.4061343 28.4205798 27.8447911
60 H 28.0181480 31.8750370 26.9957076
61 H 27.2400159 30.4022812 26.7540453
62 H 28.9042345 30.4952066 26.8044964
63 H 31.7983846 26.7667759 28.3769809
64 H 36.0834183 27.8387247 32.1412142

Chimera14^{2}-PC-Cl

QM(B1)/MM Energy = -3360.873486847769 a.u.

QM(B2)/MM Energy = -3363.004149000000 a.u.

QM(B3)/MM Energy = -3362.470895764175 a.u.

1 C 30.8288948 26.9894499 29.0545622
2 H 30.1928383 27.6273077 28.4268236
3 H 30.3151365 26.0196690 29.1360892
4 C 30.9741487 27.6228379 30.4008181
5 N 31.2240825 26.9266234 31.5636046
6 H 31.3451724 25.9107983 31.6243717
7 C 31.2889678 27.8077985 32.5864398
8 H 31.4645925 27.5182483 33.6207657
9 N 31.0734167 29.0403153 32.1508131
10 C 30.8779232 28.9379532 30.7921329
11 H 30.6749891 29.8147339 30.1839775
12 C 36.3131066 28.5863543 32.7029549
13 H 37.3096049 28.9985569 32.4731915
14 H 36.2945111 28.4199291 33.7905428
15 C 35.2320153 29.5510606 32.3294415
16 N 35.2291726 30.3621645 31.2080593
17 H 35.9691764 30.4312082 30.4987404
18 C 34.0847595 31.0870107 31.2151774
19 H 33.8318503 31.8070587 30.4384958
20 N 33.3489408 30.7963269 32.2765627
21 C 34.0568213 29.8462703 32.9778586
22 H 33.6843263 29.4227859 33.9074254
23 Cl 28.9884713 33.6103728 29.1966092
24 Fe 31.2587338 31.0986222 32.8396875
25 O 29.8205246 31.8156182 31.8448171
26 O 30.8184439 32.4064878 36.7133164
27 C 31.4191236 31.6653844 35.9322348
28 O 31.3389896 31.7643916 34.6464608
29 C 32.2966719 30.5295011 36.4525011
30 C 31.5605606 29.1789120 36.3809433
31 C 30.5801647 28.9843802 37.5724702
32 O 29.4077421 28.6174878 37.2975140
33 O 31.0747034 29.1830399 38.7001478
34 H 33.2240983 30.5110209 35.8558903
35 H 32.5474886 30.7079530 37.5069936
36 H 31.0213411 29.0752851 35.4248102
37 H 32.3010972 28.3589977 36.4440054
38 N 28.0459225 30.9790509 27.3082912
39 C 28.0362032 30.6699596 28.7776578
40 H 29.0717520 30.8235257 29.1072442
41 C 27.1493982 31.5741208 29.6452941
42 H 26.0861904 31.2901263 29.5632090
43 H 27.4751954 31.3761102 30.6781623
44 C 27.2220222 33.0761524 29.3710842
45 H 26.8094092 33.3157970 28.3826251
46 H 29.9836148 32.3843512 31.0820711
47 C 26.5494136 33.9404449 30.4402063
48 H 25.5418540 33.5331422 30.6278383
49 H 27.1212603 33.8389655 31.3748364
50 C 26.4758776 35.3971125 29.9857059

51 H 27.3815885 35.6575801 29.4248159

52 H 25.6211184 35.5791570 29.3225224

53 N 26.4030718 36.4044245 31.0867725

54 H 26.6297912 37.3252516 30.6616781

55 H 27.1249283 36.2465032 31.8202077

56 H 25.4712584 36.5109371 31.5418456

57 C 27.7057087 29.1623691 28.9398117

58 O 27.6724308 28.7020266 30.0954872

59 O 27.4813867 28.5270418 27.8714858

60 H 28.0968668 31.9840869 27.0473397

61 H 27.2481919 30.5554532 26.7889446

62 H 28.9025325 30.5462229 26.9160959

63 H 31.7636853 26.7840089 28.5329992

64 H 36.2807659 27.6207214 32.1984013

Chimera14^{2}-TS-OH

QM(B1)/MM Energy = -3360.815312985455 a.u.

QM(B2)/MM Energy = -3362.940852000000 a.u.

QM(B3)/MM Energy = -3362.423378232682 a.u.

1 C 30.8660283 26.8939873 28.7347146

2 H 30.2222679 27.4330725 28.0295207

3 H 30.3978529 25.9138066 28.9017494

4 C 30.9275270 27.6808497 29.9997049

5 N 31.0414295 27.1528248 31.2668609

6 H 31.1844434 26.1571244 31.4743099

7 C 31.0174617 28.1719545 32.1559847

8 H 31.0980923 28.0485541 33.2340224

9 N 30.8727361 29.3297348 31.5217773

10 C 30.8409458 29.0381333 30.1823664

11 H 30.7901472 29.8179745 29.4289763

12 C 36.1512910 28.6556101 32.6934775

13 H 37.1580624 29.0712244 32.5195595

14 H 36.0735410 28.4922240 33.7795191

15 C 35.0971180 29.6221586 32.2477990

16 N 35.2162902 30.4534580 31.1473970

17 H 36.0100320 30.5096630 30.4985953

18 C 34.0931575 31.2001262 31.0548689

19 H 33.9095315 31.9303866 30.2698796

20 N 33.2503789 30.9092766 32.0354055

21 C 33.8662649 29.9297715 32.7831381

22 H 33.3888762 29.4995186 33.6583512

23 Cl 31.5074561 32.8601163 30.3161364

24 Fe 31.0060457 31.3370308 32.1722705

25 O 29.1816923 32.2105138 31.9350903

26 O 30.9492742 32.4746310 35.7154702

27 C 31.3254171 31.3947937 35.2710560

28 O 31.0561458 30.9517151 34.0714792

29 C 32.1935326 30.4616942 36.1149728

30 C 31.6048559 29.0570246 36.3363121

31 C 30.6653254 29.0047160 37.5728265

32 O 29.4781791 28.6348531 37.3716793

33 O 31.1940741 29.3204576 38.6595749

34 H 33.1676734 30.4019239 35.5988394

35 H 32.3611088 30.9287129 37.0955090

36 H 31.0702305 28.7088943 35.4385534

37 H 32.4291695 28.3501231 36.5383152

38 N 28.0193787 30.9700562 27.5159500

39 C 28.0046537 30.5551633 28.9573776

40 H 29.0263258 30.6906009 29.3292620

41 C 27.0801159 31.3609468 29.8683016

42 H 26.0626360 31.4333885 29.4414329

43 H 26.9641508 30.7552496 30.7831766

44 C 27.5642872 32.7101989 30.2629840

45 H 28.4551922 33.1059389 29.7648804

46 H 29.5283672 33.0123180 31.5150329

47 C 26.6244389 33.6947836 30.8923037
48 H 25.5946287 33.3000239 30.8887570
49 H 26.8962847 33.8088526 31.9536176
50 C 26.6968940 35.0624855 30.1978049
51 H 27.7310005 35.2735045 29.8931051
52 H 26.0719667 35.0907090 29.2952481
53 N 26.3059314 36.2221222 31.0561830
54 H 26.5278967 37.0898144 30.5224183
55 H 26.8809139 36.2669657 31.9258874
56 H 25.3069596 36.2919720 31.3455664
57 C 27.6626382 29.0457244 29.0242156
58 O 27.6755225 28.5294113 30.1599010
59 O 27.3645137 28.4805896 27.9409226
60 H 27.8776657 31.9777079 27.2986750
61 H 27.3296119 30.4278805 26.9512783
62 H 28.9602216 30.7500108 27.1268293
63 H 31.8364024 26.7109306 28.2732695
64 H 36.1578603 27.6880610 32.1916082

Chimera14^{2}-PC-OH

QM(B1)/MM Energy = -3360.917577090083 a.u.

QM(B2)/MM Energy = -3363.042733000000 a.u.

QM(B3)/MM Energy = -3362.507944096181 a.u.

1 C 30.8593457 27.0626138 28.9709161
2 H 30.2161782 27.6620643 28.3117819
3 H 30.3547024 26.0952502 29.1076929
4 C 31.0159265 27.7717372 30.2770077
5 N 31.2294346 27.1410485 31.4830939
6 H 31.3277321 26.1267357 31.6064319
7 C 31.3440571 28.0839364 32.4440068
8 H 31.5135769 27.8584472 33.4949895
9 N 31.1917204 29.2967315 31.9247674
10 C 30.9945460 29.1144315 30.5746148
11 H 30.8751778 29.9575106 29.8985662
12 C 36.5366104 28.4528601 32.7295010
13 H 37.5496556 28.8122730 32.4869652
14 H 36.5301164 28.2524335 33.8102350
15 C 35.5056722 29.4837618 32.4155267
16 N 35.5274383 30.3291031 31.3228606
17 H 36.2458392 30.3809330 30.5912683
18 C 34.4164163 31.0943878 31.3534403
19 H 34.1581220 31.8331812 30.5970028
20 N 33.6789030 30.7959548 32.4130160
21 C 34.3465844 29.7970032 33.0827661
22 H 33.9601333 29.3522372 33.9969065
23 Cl 31.5551116 32.7424548 30.8092110
24 Fe 31.6256347 31.2617084 32.6535097
25 O 28.4718304 32.9113732 30.8749645
26 O 30.5940341 32.4415957 36.3725630
27 C 31.1870023 31.6405040 35.6575045
28 O 30.8854251 31.4313883 34.4039427
29 C 32.3170666 30.7855142 36.2221976
30 C 31.8882877 29.3185339 36.4164344
31 C 30.9695669 29.1491643 37.6603443
32 O 29.7905494 28.7637205 37.4465717
33 O 31.5078583 29.4145354 38.7567392
34 H 33.1769539 30.8584541 35.5344251
35 H 32.6133601 31.1908143 37.1997509
36 H 31.3732310 28.9458472 35.5158690
37 H 32.7897660 28.7018116 36.5838742
38 N 28.0491893 30.9025554 27.4826357
39 C 27.9518583 30.6289694 28.9555768
40 H 28.9409290 30.8366166 29.3853274
41 C 26.9231256 31.5119120 29.6673531
42 H 25.9756785 31.5090197 29.1122292

43 H 26.7324601 31.0546780 30.6496377
44 C 27.4279294 32.9407709 29.9140868
45 H 27.8089540 33.3505973 28.9576081
46 H 29.3709228 32.9252780 30.5007797
47 C 26.3556938 33.9075209 30.4479949
48 H 25.3708270 33.6663895 30.0330567
49 H 26.2960988 33.7673318 31.5389563
50 C 26.7568024 35.3383919 30.0950288
51 H 27.8521663 35.4262495 30.0866117
52 H 26.3863491 35.6222088 29.1001350
53 N 26.3088834 36.4061894 31.0443535
54 H 26.5601821 37.3150474 30.6064372
55 H 26.8433202 36.3607130 31.9383033
56 H 25.2949728 36.4475434 31.2796061
57 C 27.6662423 29.1164277 29.1312889
58 O 27.7546811 28.6544777 30.2861770
59 O 27.3425180 28.4898724 28.0877567
60 H 27.9444800 31.8921999 27.1795383
61 H 27.3546691 30.3333585 26.9532045
62 H 28.9899796 30.6101836 27.1538480
63 H 31.8010103 26.8468927 28.4661396
64 H 36.4205467 27.5148609 32.1865887

Chimera14^{2}-Iso1-TS

QM(B1)/MM Energy = -3360.829571105328 a.u.

QM(B2)/MM Energy = -3362.957982000000 a.u.

QM(B3)/MM Energy = -3362.434068516189 a.u.

1 C 30.8534694 26.9285993 28.8501848
2 H 30.1953739 27.4810783 28.1689769
3 H 30.3822847 25.9502444 29.0213642
4 C 30.9668502 27.7003179 30.1218799
5 N 31.1543800 27.1494532 31.3733801
6 H 31.2942571 26.1478920 31.5551390
7 C 31.2034807 28.1463242 32.2791066
8 H 31.3468697 28.0076283 33.3481426
9 N 31.0391887 29.3198272 31.6705818
10 C 30.9019756 29.0547181 30.3278541
11 H 30.7960441 29.8528795 29.5991691
12 C 36.2951450 28.6242281 32.7006771
13 H 37.3074143 29.0081975 32.4913535
14 H 36.2512032 28.4564570 33.7874066
15 C 35.2530993 29.6235114 32.3061516
16 N 35.3117509 30.4385324 31.1862643
17 H 36.0664082 30.4797415 30.4900929
18 C 34.2005432 31.1998016 31.1490307
19 H 33.9636075 31.9044350 30.3547393
20 N 33.4235563 30.9350576 32.1929347
21 C 34.0715456 29.9577899 32.9203223
22 H 33.6378875 29.5545015 33.8288711
23 Cl 31.3766787 32.4140485 30.2661294
24 Fe 31.2939186 31.2542345 32.4577859
25 O 30.0617136 32.5680242 32.8675462
26 O 30.8990971 32.0320710 35.8945776
27 C 31.5251392 31.0644846 35.4903213
28 O 31.4565940 30.5829998 34.2658809
29 C 32.4573091 30.2623938 36.3885078
30 C 31.8858800 28.8638176 36.6803277
31 C 30.7795545 28.9552671 37.7642135
32 O 29.6116405 28.6270193 37.4321750
33 O 31.1768243 29.3648875 38.8745764
34 H 33.4389878 30.1965792 35.8888680
35 H 32.5808692 30.7921304 37.3432796
36 H 31.4930437 28.4012875 35.7601812
37 H 32.6903580 28.2216174 37.0785823
38 N 27.9516327 31.0247915 27.4744215

39 C 27.9360407 30.6726090 28.9319428

40 H 28.9505539 30.8833211 29.2983480

41 C 26.9582120 31.5026528 29.7710948

42 H 25.9157119 31.3280396 29.4493938

43 H 27.0178641 31.0613917 30.7872152

44 C 27.2730976 32.9597280 29.8275273

45 H 28.2938017 33.2734796 29.5725793

46 H 29.9647952 33.1127912 32.0714050

47 C 26.4430970 33.9214268 30.6195917

48 H 25.3860364 33.6094109 30.6439669

49 H 26.7873771 33.9072896 31.6755934

50 C 26.5895651 35.3360895 30.0617889

51 H 27.6194335 35.4969580 29.7164993

52 H 25.9272454 35.5080314 29.2034335

53 N 26.3401716 36.4323442 31.0494418

54 H 26.5802691 37.3262620 30.5762229

55 H 26.9769129 36.3610358 31.8713304

56 H 25.3639319 36.5232503 31.4029468

57 C 27.6619806 29.1540945 29.0721117

58 O 27.7314588 28.6713924 30.2203346

59 O 27.3513352 28.5361483 28.0166029

60 H 27.8046630 32.0282278 27.2389866

61 H 27.2589767 30.4718418 26.9265264

62 H 28.8936826 30.7991908 27.0937913

63 H 31.8065508 26.7391061 28.3564283

64 H 36.2467886 27.6607693 32.1932562

Chimera14^2-Iso1-IM

QM(B1)/MM Energy = -3360.835310748868 a.u.

QM(B2)/MM Energy = -3362.966673000000 a.u.

QM(B3)/MM Energy = -3362.442323875412 a.u.

1 C 30.8485262 27.0029722 29.0011885

2 H 30.1692053 27.5501899 28.3360275

3 H 30.3830347 26.0246664 29.1907678

4 C 31.0024794 27.7817610 30.2664277

5 N 31.2226196 27.2374705 31.5142034

6 H 31.3619013 26.2369888 31.6960594

7 C 31.2944835 28.2485424 32.4102761

8 H 31.4494440 28.1070244 33.4774961

9 N 31.1175366 29.4203316 31.8061960

10 C 30.9446400 29.1386813 30.4718323

11 H 30.8065797 29.9272103 29.7392673

12 C 36.3858135 28.5711032 32.7098378

13 H 37.3936676 28.9508859 32.4746857

14 H 36.3701667 28.3947606 33.7956525

15 C 35.3386105 29.5773510 32.3539773

16 N 35.3599065 30.3938068 31.2353351

17 H 36.0890440 30.4296044 30.5115781

18 C 34.2551274 31.1656515 31.2439687

19 H 33.9964193 31.8794391 30.4638568

20 N 33.5233654 30.9021130 32.3182019

21 C 34.1838451 29.9162000 33.0157248

22 H 33.7909861 29.5048080 33.9407601

23 Cl 31.0391519 32.3783523 30.5241275

24 Fe 31.5141439 31.4634326 32.6586439

25 O 31.6363727 33.1783009 33.3948182

26 O 30.3396143 31.8898402 36.1222048

27 C 31.0893768 31.1011746 35.5721028

28 O 30.9569654 30.6895680 34.3246560

29 C 32.2252645 30.4284987 36.3268879

30 C 31.8425101 28.9778991 36.6735164

31 C 30.7537774 28.9833895 37.7794813

32 O 29.5929851 28.6300017 37.4467682

33 O 31.1507693 29.3757872 38.8957960

34 H 33.1412740 30.4751190 35.7154348

35 H	32.3963337	30.9741969	37.2658919
36 H	31.4783739	28.4468513	35.7782572
37 H	32.7294746	28.4506939	37.0645611
38 N	27.9413891	31.0145248	27.4634319
39 C	27.9263966	30.6545318	28.9198027
40 H	28.9376024	30.8737951	29.2897245
41 C	26.9393280	31.4712410	29.7606526
42 H	25.8980136	31.2842955	29.4415570
43 H	27.0079151	31.0328718	30.7771117
44 C	27.2436250	32.9305958	29.8102671
45 H	28.2624199	33.2441794	29.5518512
46 H	31.9963454	33.8206995	32.7645368
47 C	26.4376739	33.8941005	30.6243064
48 H	25.3784892	33.5916772	30.6741053
49 H	26.8101843	33.8793257	31.6716755
50 C	26.5832552	35.3058978	30.0578636
51 H	27.5998871	35.4480810	29.6689018
52 H	25.8862490	35.4870961	29.2295820
53 N	26.3959943	36.4108532	31.0500550
54 H	26.6215211	37.2985346	30.5576796
55 H	27.0743041	36.3429851	31.8368345
56 H	25.4363652	36.5162660	31.4431014
57 C	27.6797817	29.1324195	29.0469937
58 O	27.7415271	28.6436607	30.1926989
59 O	27.4014100	28.5146130	27.9814680
60 H	27.7863350	32.0177513	27.2337476
61 H	27.2572837	30.4573283	26.9090160
62 H	28.8884194	30.8014602	27.0910895
63 H	31.7821274	26.8091079	28.4731047
64 H	36.3116388	27.6136699	32.1941957

Chimera14^{2}-Iso1-TS-Cl

QM(B1)/MM Energy = -3360.818738678899 a.u.

QM(B2)/MM Energy = -3362.949910000000 a.u.

QM(B3)/MM Energy = -3362.431258933327 a.u.

1 C	30.8425624	27.0599501	28.9987052
2 H	30.1731903	27.6089622	28.3242772
3 H	30.3645072	26.0872109	29.1863879
4 C	30.9849801	27.8368408	30.2679858
5 N	31.1742368	27.2746378	31.5126240
6 H	31.3111133	26.2722348	31.6844801
7 C	31.2117182	28.2728458	32.4269029
8 H	31.3399310	28.1140395	33.4954534
9 N	31.0403059	29.4514095	31.8391729
10 C	30.9135592	29.1918401	30.4963112
11 H	30.7775387	29.9921874	29.7757167
12 C	36.2342744	28.6916392	32.6714088
13 H	37.2401211	29.0911006	32.4601739
14 H	36.1880778	28.5437885	33.7611594
15 C	35.1799744	29.6730300	32.2590741
16 N	35.2365068	30.4961282	31.1469602
17 H	35.9890895	30.5338663	30.4479563
18 C	34.1308514	31.2761087	31.1382094
19 H	33.9274645	32.0174353	30.3667690
20 N	33.3621319	31.0094522	32.1835344
21 C	34.0052630	30.0139744	32.8854085
22 H	33.5928251	29.5975763	33.7997712
23 Cl	29.6385981	32.3808415	30.9886926
24 Fe	31.2884489	31.4972789	32.7263568
25 O	31.4944957	33.2579310	33.3048893
26 O	30.2837988	31.9701650	36.2961018
27 C	30.9235331	31.1661959	35.6307686
28 O	30.6208828	30.7922614	34.4115470
29 C	32.1258928	30.4512291	36.2367757
30 C	31.7759419	28.9837149	36.5381370

31 C 30.7512611 28.9296685 37.7010796
32 O 29.5695113 28.6064251 37.4081978
33 O 31.2097885 29.2494766 38.8168856
34 H 32.9794024 30.5340916 35.5434103
35 H 32.3929283 30.9473139 37.1805992
36 H 31.3550822 28.4957615 35.6437075
37 H 32.6870479 28.4427144 36.8481655
38 N 27.9889713 30.8787643 27.3227841
39 C 27.9871833 30.5374477 28.7823630
40 H 29.0130004 30.7085290 29.1290726
41 C 27.0749607 31.4125036 29.6366458
42 H 26.0373189 31.3901655 29.2523172
43 H 27.0322457 30.9323227 30.6270083
44 C 27.5262724 32.8281314 29.7940962
45 H 28.0739965 33.2773353 28.9587039
46 H 32.0170500 33.8615891 32.7586169
47 C 26.7365925 33.7716186 30.6633848
48 H 25.6911596 33.4208202 30.7424854
49 H 27.1449482 33.7399608 31.6879544
50 C 26.7957274 35.1890847 30.0980872
51 H 27.8146492 35.3964033 29.7493034
52 H 26.1186007 35.3087417 29.2411179
53 N 26.4925290 36.2972364 31.0579173
54 H 26.6676127 37.1850098 30.5428645
55 H 27.1521540 36.3105068 31.8641187
56 H 25.5178394 36.3560049 31.4233999
57 C 27.6848929 29.0313378 28.9367727
58 O 27.6887226 28.5715635 30.0966165
59 O 27.4467656 28.3985962 27.8718542
60 H 27.9433027 31.8896308 27.0824726
61 H 27.2317321 30.3977692 26.7925297
62 H 28.8882014 30.5442547 26.9261561
63 H 31.7773755 26.8495890 28.4791478
64 H 36.2114107 27.7158407 32.1862680

Chimera14^{2}-Iso1-PC-Cl
QM(B1)/MM Energy = -3360.872518780227 a.u.
QM(B2)/MM Energy = -3363.003791000000 a.u.
QM(B3)/MM Energy = -3362.471063351884 a.u.

1 C 30.8510306 26.9568340 28.9218100
2 H 30.2261722 27.5911244 28.2772029
3 H 30.3348829 25.9897202 29.0093663
4 C 30.9897428 27.6058491 30.2600613
5 N 31.2061292 26.9203729 31.4344167
6 H 31.2940895 25.9027432 31.5154659
7 C 31.3006656 27.8158213 32.4415932
8 H 31.4630510 27.5381993 33.4814142
9 N 31.1324238 29.0467240 31.9822985
10 C 30.9453011 28.9303003 30.6258235
11 H 30.7836609 29.8004757 29.9960538
12 C 36.3082858 28.5901271 32.7158379
13 H 37.3050320 29.0022779 32.4866705
14 H 36.2925959 28.4160213 33.8022976
15 C 35.2307488 29.5631076 32.3536918
16 N 35.2424442 30.3967864 31.2493533
17 H 35.9853306 30.4677476 30.5431236
18 C 34.1057919 31.1352085 31.2673166
19 H 33.8640444 31.8732738 30.5041074
20 N 33.3610092 30.8307201 32.3192168
21 C 34.0549115 29.8567387 33.0030651
22 H 33.6699102 29.4154243 33.9193367
23 Cl 28.9492983 33.6608292 29.1633900
24 Fe 31.2329423 31.0512920 32.7657684
25 O 29.9207777 31.9391700 31.7616968
26 O 30.7731526 32.3880076 36.5422780

27 C 31.3291551 31.5146889 35.8753972
28 O 30.9899120 31.1927026 34.6700656
29 C 32.4641157 30.6820544 36.4671885
30 C 32.0398607 29.2114831 36.6261562
31 C 31.0087982 29.0750878 37.7773047
32 O 29.8426544 28.7092646 37.4746927
33 O 31.4544288 29.3679580 38.9079867
34 H 33.3414633 30.7729693 35.8027242
35 H 32.7250134 31.0833482 37.4559255
36 H 31.6145507 28.8337949 35.6819782
37 H 32.9232449 28.6017470 36.8883189
38 N 28.0320072 30.9187422 27.3976496
39 C 28.0350898 30.6784629 28.8801594
40 H 29.0691961 30.8527426 29.2026449
41 C 27.1423123 31.6109135 29.7100954
42 H 26.0813678 31.3145149 29.6367824
43 H 27.4675950 31.4513867 30.7495063
44 C 27.1949598 33.1030998 29.3861007
45 H 26.7647975 33.3066221 28.3976157
46 H 30.0913467 32.4748568 30.9786156
47 C 26.5255202 33.9847726 30.4423000
48 H 25.5232395 33.5727675 30.6465817
49 H 27.1058703 33.9043799 31.3729769
50 C 26.4365967 35.4342344 29.9711067
51 H 27.3288552 35.6911531 29.3880874
52 H 25.5668954 35.6079319 29.3257886
53 N 26.3854930 36.4500217 31.0664753
54 H 26.6144678 37.3667907 30.6341088
55 H 27.1155982 36.2900427 31.7911677
56 H 25.4605733 36.5618246 31.5343064
57 C 27.6916669 29.1805041 29.1134824
58 O 27.7433437 28.7516060 30.2813559
59 O 27.3682120 28.5309627 28.0816458
60 H 28.0809691 31.9110491 27.0908961
61 H 27.2292212 30.4679970 26.9119340
62 H 28.8795481 30.4606036 27.0125596
63 H 31.7998292 26.7442031 28.4292286
64 H 36.2746001 27.6277927 32.2051081

Chimera14^2-Iso2-TS

QM(B1)/MM Energy = -3360.833347669434 a.u.
QM(B2)/MM Energy = -3362.963958000000 a.u.
QM(B3)/MM Energy = -3362.441908509401 a.u.

1 C 30.8417094 27.0753203 29.0675077
2 H 30.1609941 27.6283577 28.4085581
3 H 30.3654135 26.1031092 29.2637863
4 C 31.0227570 27.8479445 30.3342379
5 N 31.2792695 27.2753106 31.5616171
6 H 31.4103741 26.2695024 31.7196032
7 C 31.3834062 28.2644588 32.4781505
8 H 31.5696733 28.0950088 33.5361446
9 N 31.1911555 29.4491662 31.9097042
10 C 30.9715785 29.2028621 30.5752063
11 H 30.7808953 30.0071526 29.8705866
12 C 36.4353834 28.5259395 32.7255349
13 H 37.4427887 28.9030141 32.4863033
14 H 36.4236767 28.3409455 33.8096922
15 C 35.3875830 29.5338904 32.3812123
16 N 35.4183934 30.3740466 31.2845308
17 H 36.1513844 30.4248628 30.5656228
18 C 34.3043551 31.1371854 31.3018549
19 H 34.0767061 31.8849368 30.5430754
20 N 33.5569425 30.8382009 32.3535115
21 C 34.2180297 29.8438610 33.0332768
22 H 33.8245627 29.4080492 33.9478482

23 Cl 29.8090874 32.3302050 31.4192951

24 Fe 31.5201789 31.5019339 32.7757763

25 O 32.2085925 33.1242925 33.2892267

26 O 30.4674332 32.1148740 36.2812595

27 C 31.1370388 31.2917561 35.6808181

28 O 30.8783104 30.8854780 34.4505313

29 C 32.3160390 30.5844424 36.3274780

30 C 31.9772430 29.1071509 36.5980446

31 C 30.9356803 29.0207871 37.7458745

32 O 29.7645935 28.6812484 37.4347952

33 O 31.3778205 29.3399469 38.8697530

34 H 33.1860822 30.6933972 35.6579161

35 H 32.5412323 31.0720036 37.2864157

36 H 31.5784993 28.6283119 35.6892703

37 H 32.8899326 28.5733533 36.9140994

38 N 27.9519401 30.9657387 27.4022743

39 C 27.8954101 30.6480806 28.8675288

40 H 28.8815254 30.9145457 29.2707699

41 C 26.8484054 31.4584467 29.6367198

42 H 25.8275605 31.1748888 29.3181734

43 H 26.9292405 31.1083262 30.6831915

44 C 27.0521314 32.9358102 29.5711653

45 H 27.9324236 33.3204743 29.0443197

46 H 32.9930684 33.5524391 32.9182460

47 C 26.3437995 33.8847742 30.4861149

48 H 25.2822392 33.6093909 30.6082722

49 H 26.7881497 33.8073077 31.4986889

50 C 26.4941970 35.3149745 29.9785597

51 H 27.5041825 35.4556798 29.5738060

52 H 25.7841808 35.5433845 29.1737165

53 N 26.3487898 36.3845087 31.0162640

54 H 26.5889855 37.2832967 30.5522590

55 H 27.0369646 36.2797328 31.7900418

56 H 25.3974422 36.4980378 31.4269667

57 C 27.6941642 29.1224582 29.0265962

58 O 27.7484522 28.6542804 30.1809662

59 O 27.4573027 28.4784229 27.9645003

60 H 27.8517567 31.9700833 27.1489336

61 H 27.2433029 30.4369622 26.8512708

62 H 28.8859605 30.6817337 27.0507366

63 H 31.7664948 26.8631004 28.5310359

64 H 36.3489068 27.5750284 32.1998382

Chimera14^{2}-Iso2-IM

QM(B1)/MM Energy = -3360.840590596986 a.u.

QM(B2)/MM Energy = -3362.970919000000 a.u.

QM(B3)/MM Energy = -3362.448162036391 a.u.

1 C 30.8384688 27.0461489 29.0675774

2 H 30.1779406 27.6434952 28.4263523

3 H 30.3436811 26.0733138 29.2053319

4 C 31.0092826 27.7478148 30.3762240

5 N 31.2489348 27.1130576 31.5766775

6 H 31.3672661 26.0995529 31.6875220

7 C 31.3540815 28.0511750 32.5441611

8 H 31.5289996 27.8264162 33.5939973

9 N 31.1766177 29.2618386 32.0320890

10 C 30.9644119 29.0874607 30.6845934

11 H 30.7821357 29.9245042 30.0156729

12 C 36.3791962 28.5537946 32.7320154

13 H 37.3756590 28.9528151 32.4813885

14 H 36.3845852 28.3702850 33.8166449

15 C 35.3043203 29.5394657 32.4021589

16 N 35.2916905 30.3711535 31.2953659

17 H 36.0185761 30.4387211 30.5709936

18 C 34.1628583 31.1146867 31.3357097

19 H 33.9021681 31.8590279 30.5843954
20 N 33.4473174 30.8092426 32.4081041
21 C 34.1473856 29.8369884 33.0823234
22 H 33.7765430 29.4130456 34.0123535
23 Cl 29.2360934 31.6587191 32.1007921
24 Fe 31.3463012 31.2764482 32.9334978
25 O 31.8683784 33.0615669 32.9852386
26 O 30.6653241 32.3055226 36.1158220
27 C 31.2694440 31.2651360 35.9035990
28 O 31.3449618 30.6730756 34.7312120
29 C 32.0233421 30.5104339 36.9901898
30 C 31.5388291 29.0591400 37.1476116
31 C 30.2180775 29.0098020 37.9603225
32 O 29.1878820 28.5658378 37.3955198
33 O 30.3037863 29.4386932 39.1304289
34 H 33.0933504 30.5378101 36.7082401
35 H 31.8779193 31.0386181 37.9438469
36 H 31.4158301 28.5835238 36.1628711
37 H 32.2960943 28.4931207 37.7176740
38 N 28.0182018 31.0035601 27.3471497
39 C 27.9337645 30.6484529 28.8018938
40 H 28.9222576 30.8748936 29.2248597
41 C 26.8973761 31.4654733 29.5771898
42 H 25.8790508 31.2627789 29.2001888
43 H 26.9166808 31.0459214 30.6007158
44 C 27.1880806 32.9308003 29.6241093
45 H 28.1840316 33.2693020 29.3136297
46 H 32.7900928 33.1728707 33.2613575
47 C 26.3759806 33.8785614 30.4531351
48 H 25.3009854 33.6509382 30.3704199
49 H 26.6266595 33.7391274 31.5245371
50 C 26.6645096 35.3159217 30.0319586
51 H 27.7483731 35.4816976 29.9443616
52 H 26.2232225 35.5344400 29.0495590
53 N 26.1767180 36.3871701 30.9585201
54 H 26.4425035 37.2955947 30.5257681
55 H 26.6120501 36.4015431 31.9039492
56 H 25.1514737 36.4229966 31.1395987
57 C 27.7036113 29.1213012 28.9106504
58 O 27.7117855 28.6229872 30.0539304
59 O 27.4986660 28.5052555 27.8280300
60 H 27.9407682 32.0165337 27.1207439
61 H 27.3028502 30.5039233 26.7764950
62 H 28.9485411 30.7066042 27.0004148
63 H 31.7691903 26.8440303 28.5375089
64 H 36.3131103 27.5979190 32.2123986

Chimera14^2-Iso2-TS-Cl

QM(B1)/MM Energy = -3360.832750917750 a.u.

QM(B2)/MM Energy = -3362.961304000000 a.u.

QM(B3)/MM Energy = -3362.442034939636 a.u.

1 C 30.8465766 27.0651808 29.0001958
2 H 30.1842989 27.6268276 28.3292855
3 H 30.3648600 26.0900460 29.1661010
4 C 30.9811401 27.8207374 30.2830362
5 N 31.2030998 27.2428183 31.5155188
6 H 31.3574789 26.2401102 31.6707785
7 C 31.2370512 28.2265796 32.4455429
8 H 31.3829542 28.0529762 33.5094268
9 N 31.0307558 29.4101007 31.8800197
10 C 30.8846937 29.1699306 30.5356002
11 H 30.7260950 29.9773947 29.8274436
12 C 36.2665667 28.6575457 32.7011918
13 H 37.2698042 29.0606113 32.4847057
14 H 36.2303810 28.4954264 33.7892976

15 C	35.2041161	29.6366364	32.3061493
16 N	35.2515219	30.4597848	31.1938512
17 H	36.0067368	30.5092461	30.4983378
18 C	34.1323423	31.2204508	31.1790588
19 H	33.9193039	31.9624907	30.4105202
20 N	33.3632934	30.9371537	32.2185751
21 C	34.0207081	29.9589071	32.9290469
22 H	33.6056317	29.5435211	33.8442327
23 Cl	29.3666622	32.2268545	31.3530751
24 Fe	31.2708494	31.4629360	32.7399232
25 O	31.7743251	33.2247310	33.1188839
26 O	30.4015888	32.2725959	36.0644838
27 C	30.9824697	31.2656697	35.6778884
28 O	30.8617180	30.7310655	34.4920661
29 C	31.9268475	30.4959546	36.5993084
30 C	31.4310816	29.0640177	36.8581781
31 C	30.1865757	29.0946497	37.7781418
32 O	29.0617475	28.8608476	37.2492243
33 O	30.4014201	29.3900745	38.9670986
34 H	32.9246775	30.4837957	36.1227571
35 H	31.9939174	31.0338977	37.5564337
36 H	31.1944503	28.5695641	35.9027804
37 H	32.2247306	28.4934345	37.3700422
38 N	28.0387414	30.9306357	27.2910743
39 C	27.9904926	30.5610265	28.7425872
40 H	29.0031053	30.7293398	29.1281981
41 C	27.0354813	31.4085568	29.5812102
42 H	26.0280835	31.4218720	29.1271115
43 H	26.9179977	30.8796296	30.5396704
44 C	27.5115248	32.8041023	29.8473256
45 H	28.2179603	33.2369239	29.1295846
46 H	32.6590584	33.2650299	33.5094228
47 C	26.6020249	33.7795384	30.5487106
48 H	25.5780024	33.6575388	30.1466764
49 H	26.5283068	33.5315853	31.6218738
50 C	27.0842421	35.2097692	30.3198307
51 H	28.1433339	35.3121260	30.5964321
52 H	26.9923716	35.4673624	29.2554486
53 N	26.3587535	36.2895876	31.0652920
54 H	26.5815888	37.1843604	30.5791575
55 H	26.6628000	36.4270309	32.0525726
56 H	25.3210609	36.2350575	31.1115723
57 C	27.6963904	29.0467209	28.8449074
58 O	27.6594204	28.5587481	29.9928010
59 O	27.5005992	28.4367811	27.7606704
60 H	27.9957837	31.9456425	27.0667548
61 H	27.2952775	30.4580693	26.7328449
62 H	28.9501855	30.6061671	26.9162140
63 H	31.7858400	26.8625802	28.4856044
64 H	36.2378814	27.6888424	32.2023350

Chimera14^{2}-Iso2-PC-Cl

QM(B1)/MM Energy = -3360.881930866828 a.u.

QM(B2)/MM Energy = -3363.016020000000 a.u.

QM(B3)/MM Energy = -3362.482074370118 a.u.

1 C	30.8503549	27.0093865	28.9340822
2 H	30.2044228	27.5992059	28.2704993
3 H	30.3583759	26.0341498	29.0651905
4 C	30.9790898	27.7249879	30.2392437
5 N	31.2290073	27.1098640	31.4485182
6 H	31.3757556	26.1017518	31.5747793
7 C	31.2935539	28.0596285	32.4068347
8 H	31.4758929	27.8580010	33.4602916
9 N	31.0757213	29.2607184	31.8829240
10 C	30.8873528	29.0651503	30.5346219

11 H	30.7091609	29.8910979	29.8508044
12 C	36.3998752	28.5640170	32.6998116
13 H	37.4129866	28.9381918	32.4776897
14 H	36.3701198	28.3879348	33.7854143
15 C	35.3586986	29.5719676	32.3311198
16 N	35.3850259	30.3725188	31.2020026
17 H	36.1224086	30.4135129	30.4884966
18 C	34.2707044	31.1372165	31.1958101
19 H	34.0462236	31.8611765	30.4136197
20 N	33.5281243	30.8831270	32.2613474
21 C	34.1978336	29.9178356	32.9803757
22 H	33.7950188	29.5406495	33.9179972
23 Cl	29.0991153	33.3801859	29.8169456
24 Fe	31.4777898	31.1607927	32.7670234
25 O	30.7154447	32.8489971	33.0029226
26 O	30.8792277	32.3659249	35.7988002
27 C	31.3788174	31.2232775	35.7602047
28 O	31.5067181	30.5032248	34.7100471
29 C	31.9646686	30.6075562	37.0330998
30 C	31.5319709	29.1522694	37.2765799
31 C	30.1404083	29.1071151	37.9457509
32 O	29.1264871	28.8904694	37.2242198
33 O	30.1258104	29.3330378	39.1713040
34 H	33.0639919	30.6592899	36.9001422
35 H	31.6866940	31.2332431	37.8949436
36 H	31.5377187	28.6061284	36.3215509
37 H	32.2459271	28.6739833	37.9679043
38 N	28.0138710	30.9640731	27.2882332
39 C	27.9697813	30.6648798	28.7563958
40 H	28.9925011	30.8404641	29.1118559
41 C	27.0367870	31.5678142	29.5713800
42 H	25.9848458	31.3994303	29.2907457
43 H	27.1395722	31.2338973	30.6153531
44 C	27.3217650	33.0694878	29.4799215
45 H	27.1767974	33.4205176	28.4479444
46 H	30.7820506	32.9088146	33.9798695
47 C	26.4676639	33.9376873	30.4153628
48 H	25.4135925	33.6482464	30.2841154
49 H	26.7403835	33.7276186	31.4596046
50 C	26.6734363	35.4065761	30.0579228
51 H	27.7458985	35.6201296	29.9622239
52 H	26.2073455	35.6442102	29.0910126
53 N	26.1579556	36.4255213	31.0231289
54 H	26.3971413	37.3537507	30.6182079
55 H	26.6043165	36.4277230	31.9634397
56 H	25.1330565	36.4345914	31.2106645
57 C	27.6578447	29.1560103	28.9253403
58 O	27.6867426	28.6977642	30.0846709
59 O	27.3881167	28.5214223	27.8706519
60 H	28.0136935	31.9685965	27.0191597
61 H	27.2503557	30.4933991	26.7572711
62 H	28.9082231	30.5833537	26.9247153
63 H	31.7982427	26.8091863	28.4345867
64 H	36.3295298	27.6068802	32.1830836

Chimera14^{2}-Iso2-TS-OH

QM(B1)/MM Energy = -3360.799734232482 a.u.

QM(B2)/MM Energy = -3362.925150000000 a.u.

QM(B3)/MM Energy = -3362.407012761394 a.u.

1 C	30.8612687	26.9769310	28.8158321
2 H	30.2145177	27.5329483	28.1251185
3 H	30.3875431	25.9954191	28.9589437
4 C	30.9170371	27.7228079	30.1090322
5 N	30.8940767	27.1432823	31.3601764
6 H	31.0094976	26.1373133	31.5351704

7 C 30.8266917 28.1316289 32.2844996

8 H 30.7962269 27.9610387 33.3590555

9 N 30.7975645 29.3180050 31.6908191

10 C 30.8787076 29.0742339 30.3441872

11 H 30.9184077 29.8827419 29.6216394

12 C 35.9680841 28.8976060 32.5552706

13 H 36.9639353 29.3452219 32.3930650

14 H 35.8507267 28.8066539 33.6463604

15 C 34.8954508 29.8051030 32.0228401

16 N 34.9829143 30.5602634 30.8630512

17 H 35.7829656 30.6007826 30.2199001

18 C 33.8476976 31.2937913 30.7515017

19 H 33.6684770 31.9832718 29.9279054

20 N 33.0313651 31.0653752 31.7691411

21 C 33.6809354 30.1482414 32.5691569

22 H 33.2418240 29.8129776 33.5040236

23 Cl 28.5295084 31.5300242 33.2782010

24 Fe 30.8173506 31.3540854 32.3847457

25 O 29.9777176 32.6638885 31.0363379

26 O 30.6369211 32.8590525 35.6888416

27 C 31.1952472 31.8076263 35.3766556

28 O 31.5739897 31.5062258 34.1689338

29 C 31.4247088 30.7269729 36.4252292

30 C 30.3546703 29.6282953 36.2845200

31 C 29.8345070 29.0221153 37.6144011

32 O 28.8178591 28.2840596 37.4824798

33 O 30.4379462 29.3013754 38.6696949

34 H 32.4409636 30.3104288 36.3181289

35 H 31.3318225 31.1699270 37.4266507

36 H 29.4723104 30.0222295 35.7566156

37 H 30.7206330 28.7899901 35.6627611

38 N 28.0773129 30.8992879 27.3584470

39 C 28.1825813 30.5226796 28.8092248

40 H 29.2509173 30.5710180 29.0406787

41 C 27.4699099 31.4568613 29.7726846

42 H 26.3827430 31.4859869 29.5601500

43 H 27.5529373 30.9988216 30.7760520

44 C 27.9759623 32.8566705 29.8517451

45 H 28.6436036 33.2363438 29.0736609

46 H 29.3328524 32.9264296 31.7166951

47 C 27.0803154 33.8331402 30.5613986

48 H 26.0484718 33.6322337 30.2080852

49 H 27.0526980 33.5933904 31.6383802

50 C 27.4293008 35.2868091 30.2840918

51 H 28.4618714 35.5195657 30.5764393

52 H 27.3331885 35.4905644 29.2089151

53 N 26.5558564 36.2889727 30.9742224

54 H 26.7017647 37.1950553 30.4808174

55 H 26.7913508 36.4815206 31.9722991

56 H 25.5317586 36.1212369 30.9725728

57 C 27.7272861 29.0488760 28.9643336

58 O 27.6983273 28.5808544 30.1198790

59 O 27.4169978 28.4540076 27.8976088

60 H 28.0364221 31.9140887 27.1338804

61 H 27.2855296 30.4284725 26.8713610

62 H 28.9407343 30.5581282 26.8944819

63 H 31.8236913 26.7915564 28.3389190

64 H 36.0466565 27.8952938 32.1342437

Chimera14^{2}-Iso2-PC-OH

QM(B1)/MM Energy = -3360.908532960206 a.u.

QM(B2)/MM Energy = -3363.033379000000 a.u.

QM(B3)/MM Energy = -3362.498081510453 a.u.

1 C 30.8439031 27.1217192 29.0698052

2 H 30.2226100 27.7857990 28.4525467

3 H	30.3062492	26.1642525	29.1395082
4 C	31.0144547	27.7255384	30.4280694
5 N	31.2169098	26.9884752	31.5759958
6 H	31.3007819	25.9667672	31.6112111
7 C	31.3289614	27.8354343	32.6202415
8 H	31.4846305	27.5082419	33.6463582
9 N	31.1918675	29.0898458	32.2135760
10 C	30.9950190	29.0347643	30.8505602
11 H	30.8437252	29.9313912	30.2540336
12 C	36.4270140	28.5623839	32.6837048
13 H	37.4285700	28.9480559	32.4319152
14 H	36.4279089	28.3913173	33.7703827
15 C	35.3598506	29.5524657	32.3428610
16 N	35.3103843	30.3357775	31.2021614
17 H	36.0231373	30.3906471	30.4637471
18 C	34.1782602	31.0747082	31.2415631
19 H	33.8960235	31.7829112	30.4639049
20 N	33.4950536	30.8183979	32.3460894
21 C	34.2270367	29.8829860	33.0435123
22 H	33.9035277	29.5148044	34.0147370
23 Cl	29.3604023	31.7890315	32.7292975
24 Fe	31.5202339	31.0072819	33.1416350
25 O	29.5692712	33.0445103	29.8473931
26 O	30.8271417	32.8573272	36.2256498
27 C	31.4734693	31.8409457	35.9750608
28 O	32.1040379	31.6297946	34.8548035
29 C	31.5039469	30.6927062	36.9646502
30 C	30.4366349	29.6597924	36.5616160
31 C	29.8155395	28.8847260	37.7491706
32 O	28.7737800	28.2272555	37.4801604
33 O	30.3786985	28.9900750	38.8539143
34 H	32.5032463	30.2263275	36.9884563
35 H	31.2584605	31.0606370	37.9687113
36 H	29.6026157	30.1611735	36.0447351
37 H	30.8393258	28.9261714	35.8386227
38 N	28.1016564	30.9951091	27.2652576
39 C	28.1377211	30.7657318	28.7489474
40 H	29.2005230	30.8168354	29.0185567
41 C	27.4377753	31.8615909	29.5628373
42 H	26.3948966	31.9989149	29.2340993
43 H	27.4079600	31.4905806	30.5993020
44 C	28.1954909	33.2137091	29.5276169
45 H	28.1793684	33.6090591	28.4973766
46 H	29.6050598	32.6459215	30.7412385
47 C	27.6298107	34.3113086	30.4522387
48 H	27.7490530	33.9746563	31.4935619
49 H	28.3035392	35.1729355	30.3205327
50 C	26.1926206	34.7704099	30.2267093
51 H	25.9509506	34.8523413	29.1574614
52 H	25.4543405	34.0989558	30.6869850
53 N	25.9930576	36.1343100	30.8141221
54 H	26.4495871	36.8624484	30.2218365
55 H	26.4050481	36.2671996	31.7624378
56 H	24.9977549	36.4027514	30.9581529
57 C	27.6805893	29.3008430	29.0039837
58 O	27.7367044	28.8728675	30.1730559
59 O	27.3197996	28.6491298	27.9857960
60 H	28.0322336	31.9838999	26.9530729
61 H	27.3381547	30.4520277	26.8088016
62 H	28.9975244	30.6458880	26.8787892
63 H	31.7714195	26.8966187	28.5433925
64 H	36.3527843	27.6010684	32.1753451

5. References

(1) Neugebauer, M. E.; Kissman, E. N.; Marchand, J. A.; Pelton, J. G.; Sambold, N. A.; Millar, D. C.; Chang, M. C. Y. Reaction Pathway Engineering Converts a Radical Hydroxylase into a Halogenase. *Nat. Chem. Biol.* **2022**, *18* (2), 171–179. https://doi.org/10.1038/s41589-021-00944-x.
